# Integration and Fixation Preferences of Human and Mouse Endogenous Retroviruses Uncovered with Functional Data Analysis

**DOI:** 10.1371/journal.pcbi.1004956

**Published:** 2016-06-16

**Authors:** Rebeca Campos-Sánchez, Marzia A. Cremona, Alessia Pini, Francesca Chiaromonte, Kateryna D. Makova

**Affiliations:** 1 Genetics Graduate Program, The Huck Institutes of the Life Sciences, Penn State University, University Park, Pennsylvania, United States of America; 2 MOX—Modeling and Scientific Computing, Department of Mathematics, Politecnico di Milano, Milano, Italy; 3 Department of Statistics, Penn State University, University Park, Pennsylvania, United States of America; 4 Center for Medical Genomics, The Huck Institutes of the Life Sciences, Penn State University, University Park, Pennsylvania, United States of America; 5 Department of Biology, Penn State University, University Park, Pennsylvania, United States of America; Temple University, UNITED STATES

## Abstract

Endogenous retroviruses (ERVs), the remnants of retroviral infections in the germ line, occupy ~8% and ~10% of the human and mouse genomes, respectively, and affect their structure, evolution, and function. Yet we still have a limited understanding of how the genomic landscape influences integration and fixation of ERVs. Here we conducted a genome-wide study of the most recently active ERVs in the human and mouse genome. We investigated 826 fixed and 1,065 *in vitro* HERV-Ks in human, and 1,624 fixed and 242 polymorphic ETns, as well as 3,964 fixed and 1,986 polymorphic IAPs, in mouse. We quantitated >40 human and mouse genomic features (e.g., non-B DNA structure, recombination rates, and histone modifications) in ±32 kb of these ERVs’ integration sites and in control regions, and analyzed them using Functional Data Analysis (FDA) methodology. In one of the first applications of FDA in genomics, we identified genomic scales and locations at which these features display their influence, and how they work in concert, to provide signals essential for integration and fixation of ERVs. The investigation of ERVs of different evolutionary ages (young *in vitro* and polymorphic ERVs, older fixed ERVs) allowed us to disentangle integration vs. fixation preferences. As a result of these analyses, we built a comprehensive model explaining the uneven distribution of ERVs along the genome. We found that ERVs integrate in late-replicating AT-rich regions with abundant microsatellites, mirror repeats, and repressive histone marks. Regions favoring fixation are depleted of genes and evolutionarily conserved elements, and have low recombination rates, reflecting the effects of purifying selection and ectopic recombination removing ERVs from the genome. In addition to providing these biological insights, our study demonstrates the power of exploiting multiple scales and localization with FDA. These powerful techniques are expected to be applicable to many other genomic investigations.

## Author Summary

Approximately half of the human genome is composed of repetitive elements. Yet we do not completely understand why certain elements insert in particular genomic locations, and what determines which elements are retained and which are eliminated from the genome. To address these questions we studied endogenous retroviruses, one type of repetitive elements which occupy ~10% of the human and mouse genomes, together with genomic features characterizing various biological processes (e.g., recombination and transcription) in the neighborhoods of these elements. Using statistical techniques, we identified enrichment of genomic features in the vicinity of endogenous retroviruses of different evolutionary ages. Features overrepresented adjacent to young endogenous retroviruses are expected to have facilitated their insertion in the genome. Features overrepresented adjacent to older endogenous retroviruses are expected to have facilitated both their insertion and their chances of being sustained in the genome. Our analyses allowed us to explain the uneven distribution of endogenous retroviruses along the genome, and thus to better understand the interaction of different biological processes in shaping the evolution of genome architecture.

## Introduction

Endogenous Retroviruses (ERVs) are Class I Transposable Elements (TEs) considered to be remnants of germ-line retrovirus infections inherited by the next generations [1]. As all Class I TEs, ERVs transpose via an RNA intermediate, i.e. they “retrotranspose”. Because they possess Long Terminal Repeats (LTRs), they are also known as LTR-retrotransposons. Depending on the similarity of their gene content to that of certain retroviruses, ERVs are classified as Gammaretrovirus-, Betaretrovirus-, and Spumaretrovirus-like [1–3]. Full-length ERVs possess three retroviral coding genes (i.e. *gag*, *pol*, and *env*) and LTR flanking sequences [4]]. In most cases, the internal genes are deleted by recombination of LTRs, converting ERVs into solo-LTRs [5,6].

Most ERVs have accumulated numerous mutations that render them inactive [7]. However, some rare examples of young ERVs that have coding capacity, are expressed and are transpositionally active, have been described in mammals, e.g. in koala [8], mouse [3], cat [9], sheep [10], and mule deer [7]. Active ERVs are transposition-competent and have integrated recently; hence for them, polymorphic events–in terms of presence/absence–are observed at the population level but the allele frequencies of integrations are low. For instance, CrERVγ is an endogenous gammaretrovirus that was recently detected in mule deer [7]. This ERV has been invading the germ line of mule deer since its speciation from white-tailed deer approximately 1.1 million years ago (MYA), and the copies found display polymorphisms in the wild mule deer population. In total, in this species, there are on average 100 full-length copies of the CrERVγ per haploid genome [7]. However, if solo-LTR elements are included, this estimate increases two-to-three-fold [7].

ERVs occupy ~8% of the reference human genome (they are called HERVs for Human ERVs), and have been integrating in it starting more than 35 MYA [6,7,11,12]. However, only the HERV-K family has been active during the past 6 MY–since the divergence of human and chimpanzee [13]. Moreover, among 113 human-specific HERV-K elements only 15 are full-length and none is infectious [13], though about a dozen were found to be polymorphic in 100 individuals from diverse populations indicating retrotransposition activity in the recent past [13,14]. In cell lines, however, two HERV-K named *Phoenix* [15] and HERV-K_CON_ [16] were reconstructed to be infectious, producing retroviral particles and causing *in vitro* integrations. Expression of HERV mRNA varies among tissues. Importantly, significant expression levels were detected in testis as well as placenta [17,18]. Some analyses are available for HERV-Ks embryonic expression [19,20].

Approximately 10% of the reference mouse genome is derived from LTR elements, including ERVs [21]. In mouse oocytes, approximately 13% of transcripts were reported to be derived from MaLRs (a type of LTR elements) as detected from ESTs [22]. Also, mice have highly active ERVs causing up to 10–12% of spontaneous germ-line insertional mutations–most of which are due to activity of IAP (Intracisternal A Particle) and MusD/ETn (or ETns in short; Early Transposon family) elements [23]. IAPs and ETns are both non-infectious betaretroviruses. In the mouse genome, full-length IAPs contain retroviral genes needed for retrotransposition; however there are also partially deleted copies (ERVs missing genes or other sequences). ETns consist of non-coding sequences and are aided by MusD proteins to retrotranspose [3]. Insertional polymorphisms have been detected for both IAPs and ETns in multiple mouse strains; additionally, some insertions arose prior to the divergence of these strains [24,25]. In the rodent lineage, out of seventeen species studied, three (*Mus*, *Spermophilus*, and *Cavia*) possess 80% of all IAP loci found in these species [26]. These elements are absent from monkeys and apes [26]. Mouse IAPs and ETns are known to transpose in different mouse strains causing mutations in the germ line; both polymorphic (in terms of presence/absence) and fixed elements are known for each mouse strain [25].

The exaptation of ERVs–i.e. the recruitment of their sequences to perform a new function as regulatory or coding sequences–has influenced the evolution of genomes in multiple ways. Some enhancers and promoters derived from ERVs assume new roles in gene regulation; e.g., the alternative promoter of the *CYP19 gene–*an enzyme important for estrogen biosynthesis–leads to its high expression levels in the primate placenta [3,27]. Another interesting example of ERV exaptation associated with the evolution of placenta is syncytin, a gene derived from the *env* gene of HERV-W [28]. Other ERV genes were exapted to function as proteases, RNA-dependent DNA polymerase with RNAse H, and integrases, as well as structural proteins, in diverse organisms [29]. Importantly, while ERVs have been relevant to genome evolution, they have also been implicated in the development of multiple diseases by disrupting genes, modifying regulatory sequences or altering gene expression. Though causal links have not been definitely established, the diseases that have been associated with ERV retrotransposition or expression include multiple sclerosis, cancer and psoriasis in human [3,11,29,30]; and obesity, diabetes, and cancer in mouse [24].

Notwithstanding the role ERVs play in the architecture, evolution, and function of genomes, our knowledge of how the genomic landscape influences their integration and fixation is still limited. Gene density and GC content have been shown to be negative predictors of ERV density–for not only full-length elements but also solo-LTRs [31–33]. In contrast, reconstructed HERV-Ks integrate in regions with high numbers of gene transcription units [34]. Similarly, *in vitro* IAP integrations occurred preferentially in actively transcribed domains of HeLa cells [35]. Interestingly, human and mouse ERVs that are located in introns are mostly present in antisense orientation avoiding gene expression disruption [32,36,37]. Other important genomic characteristics of ERVs and their genomic neighborhoods are high levels of methylation and epigenetic modifications used by the genome to limit transposition [3,38]. ETns, however, show decreased methylation when located in the vicinity of transcription start sites and expressed genes [39]. Chromosome location is another relevant feature of ERV distribution, as illustrated by the description of 100 previously unknown HERV-Ks in the centromeres of 15 chromosomes [40]. It has been suggested [34] that the accumulation of ERVs is the net result of two processes–integration, which can be biased towards certain genomic landscapes, and purifying selection, which removes ERVs disrupting the function of important elements, e.g. of genes. Disentangling these two processes can be challenging and requires the investigation of ERVs that integrated in the genome at different times.

Several approaches have been used to elucidate the relationships between genomic features and distribution of TEs. Most studies of the associations between genomic features and TE density, Integration Site (IntS) preferences, or neighboring sequences characteristics were performed employing statistical methods such as ROC curves [41], non-parametric tests [42], Fisher exact tests [43], maximum likelihood techniques [33], MANOVA [44], and multiple regressions [45,46]. The main limitation of many past studies was the low data resolution determined by available technologies. However, resolution has recently improved, e.g., with the release of ENCODE and ModENCODE consortia data [47]. The application of innovative statistical approaches though has not kept pace with the improvement in data. Statistical methodology should address the fact that many features of the genome act jointly in defining its biological functionality. Being able to consider multiple genomic features simultaneously, e.g., with multiple regression analyses [45,46], is essential to obtain meaningful biological conclusions. Moreover, with the availability of higher resolution data, it becomes paramount to use statistical techniques capable of detecting and differentiating effects at different scales and locations, e.g., one genomic feature may be generally enriched or depleted in the broad flanks of a TE, while another may show enrichment or depletion at a specific location in close proximity of the element’s IntS.

To perform more powerful and effective analyses, one can view genomic features as “curves” composed of measurements in consecutive genomic intervals. In this framework, Functional Data Analysis (FDA) techniques can be exploited to extract signals from these curves, taking advantage of the ordered nature of the measurements and considering different scales and locations, i.e. sizes and positions of genomic intervals (see [48] and [49] for a comprehensive introduction to FDA). This class of techniques includes curve smoothing and registration methods, functional principal component analysis, functional hypothesis testing, functional regression, and functional clustering [50]. In the last decade FDA has been utilized in an increasing number of biomedical applications [51,52], particularly in cardiovascular research [53–55] and kinesiology [56]. Although still limited in number, some applications of FDA also exist in the context of genetics and genomics, e.g., in genetic association studies [57–59], epistasis analysis [60], and ChIP-seq peak shape clustering [61].

Here, applying FDA methodology, we address three questions about the biology of ERVs. First, what genomic features are significant for ERV integration and fixation? Second, at what genomic scales and locations are these features influential? Third, and finally, how do genomic features work in concert to provide signals essential for integration and fixation of ERVs? Using genome-wide data, we applied the recently developed Interval Testing Procedure (ITP) [62] to determine the influence of flanking sequence features on integration and fixation of mouse (polymorphic and fixed ETns and IAPs) and human (fixed and *in vitro* HERV-Ks) ERVs. As a result, we detected diverse genomic features that affect integration and fixation of these elements (e.g. gene content, replication timing, AT count, and LINE content), and did so differentiating effects at various scales and locations in the flanking regions. Finally, we employed multiple Functional Logistic Regression (FLR) models to capture the combined effects of a restricted set of features resulting in a compact group of genome features that define the genomic landscape of integration and/or fixation preferences for ERVs. Importantly, the functional testing procedures and regression techniques we extended, employed and demonstrated in this study can be broadly applied in genomics.

## Results

### Elements and controls

In this study we analyzed *in vitro*, polymorphic, and fixed ERVs. The distributions of *in vitro* and polymorphic ERVs are only marginally influenced by selection and thus provide a more accurate view of integration preferences. Fixed ERVs, in contrast, carry information about both integration and fixation. We interrogated the genomic neighborhoods (32-kb flanking sequences upstream and 32-kb flanking sequences downstream of each element, so there is no overlap among flanking regions to maximize the number of ERVs in the study) of one human and two mouse ERV families. In mouse, we considered 1,866 ETns (242 polymorphic and 1,624 fixed) and 5,950 IAPs (1,986 polymorphic and 3,964 fixed) detected genome-wide by Zhang and colleagues [25]; elements were considered to be fixed if they were shared among four mice strains, and polymorphic if they were present in the C57BL/6J strain but not in the other three strains (see Methods). As control regions, we considered 1,379 continuous 64-kb regions of the mouse genome that did not overlap with the flanking sequences of ERVs (see Methods, Table 1). In human, we considered 826 fixed HERV-Ks (Table 1) annotated by Subramanian and colleagues [63]. We also extracted the genomic locations of 1,065 *in vitro* HERV-K integrations in human embryonic kidney and fibrosarcoma cell lines [34] (Table 1). A total of 1,690 control regions were generated similarly to those in mouse (see Methods). Human and mouse ERVs in our analyses ranged from solo-LTRs (~60 bp) to full-length elements (~9 kb) (Table A in S1 Text). The number of ERVs present on each chromosome correlated with chromosome size (Fig A in S1 Text). Human chromosome 19 was an outlier with an overrepresentation of fixed HERV-Ks (Fig A in S1 Text).

**Table 1:** Number of ERV elements and control regions used in this study.

Genome	Datasets	Elements in the reference	Elements used	Sample size after filtering	Reference
Mouse	Polymorphic ETn	248	242	217	[25]
	Fixed ETn	1868	1624	1296	[25]
	Polymorphic IAP	2224	1986	1788	[25]
	Fixed IAP	5064	3964	3255	[25]
	Control regions		1379	1142	
Human	*In vitro* HERV-K	1565	1065	1005	[34]
	Fixed HERV-K	1036	826	826	[63]
	Control regions		1690	1543	

### Genomic features

**Fig 1:**
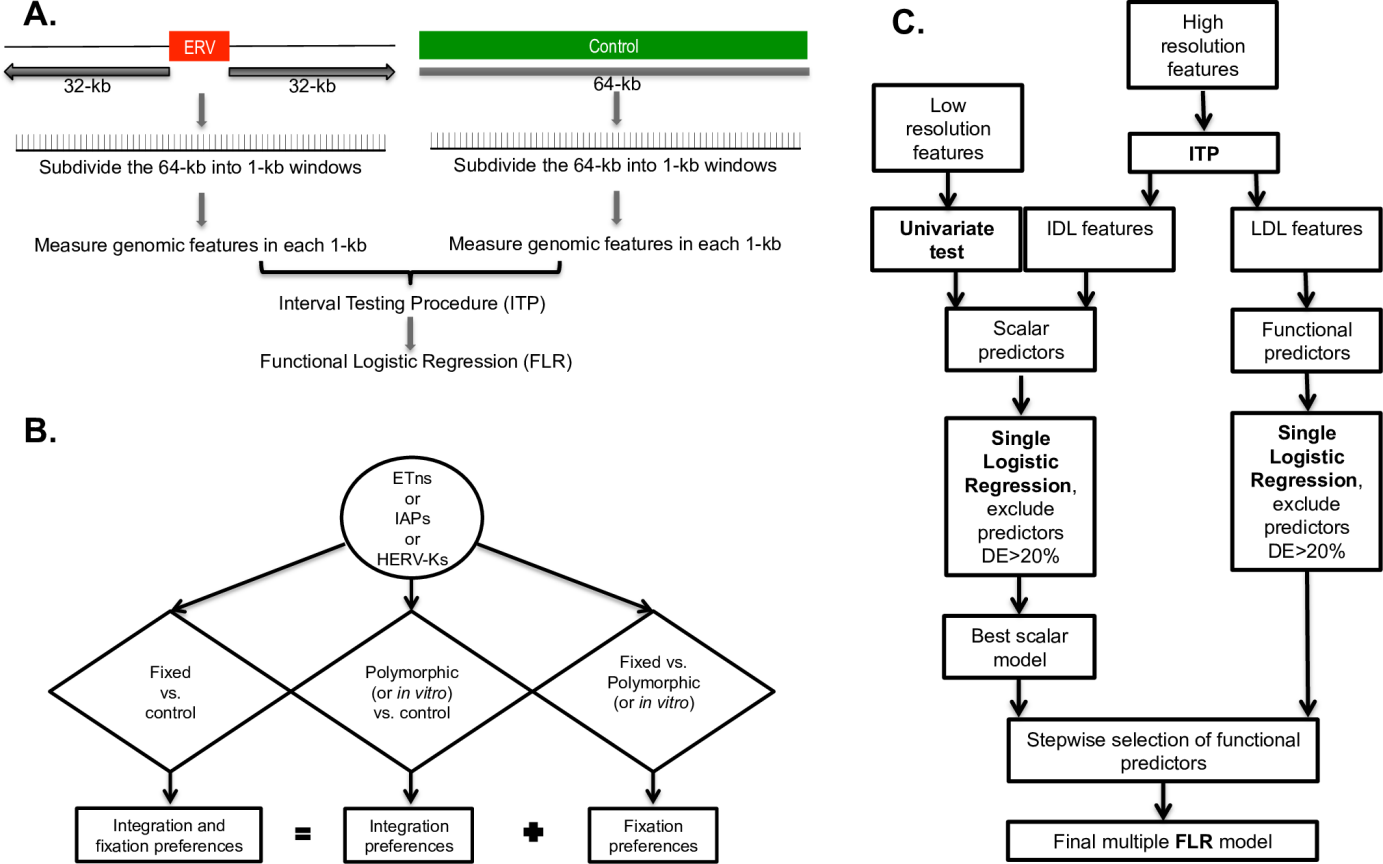
Workflow of the methodology employed to compare the flanking regions of ERVs versus control regions. The comparison between the flanking regions of two different ERV types utilizes an analogous pipeline. (A) Generation of windows and data. (B) Schematic of the nine comparisons implemented in our study. (C) Schematic of the statistical analysis, including Functional Data Analysis techniques. FLR: Functional Logistic Regression, ITP: interval testing procedure, IDL: invariant differential landscape, LDL: localized differential landscape.

We selected a diverse set of genomic features (Table 2) that could be implicated in ERV integration or fixation as reported by previous ERV [31–33] and non-ERV TE studies [45,46]. In total, we considered 41 and 43 genomic features in mouse and human ERV flanking regions, respectively (derived from 43 datasets in mouse and 44 datasets in human). These features reflected DNA conformation (e.g., G-quadruplex), DNA sequence (e.g., A/T content), position on the chromosome (e.g., distance to the closest centromere and telomere), recombination (e.g., local recombination rates), replication (e.g., replication timing), gene regulation and expression (e.g., histone marks and DNase I hypersensitive sites), as well as selection (e.g., exons and most conserved elements). Where possible, we specifically utilized features studied in embryonic stem cells (ESCs) or in sperm cells as they most closely proxy characteristics of germ-line and embryonic cells [64]. Four *low-resolution features* (replication timing, recombination rates, distance to telomere, and distance to centromere) were represented by a single value for each 64-kb region. For each *high-resolution feature*, we measured either its content (fraction of the genomic window covered by the feature), its count or its weighted average (WA, only for methylation and expression features) in each of the 64 1-kb windows constituting the flanks of each ERV and each control region (Fig 1A, see Methods). We applied hierarchical clustering to screen out high-resolution genomic features that present strong correlations with each other (Figs B and C in S1 Text). For example, for human, exon content was highly correlated with gene expression in ESCs and thus we removed the latter from the analysis. As a result, a total of 35 mouse and 36 human high-resolution genomic features (derived from 35 datasets in mouse and 37 datasets in human) were retained for further analysis (Figs D and E in S1 Text).

**Table 2:** Complete list of genomic features analyzed in this study. H and L marks indicate features analyzed in mouse, human or both using high- and low-resolution datasets, respectively. The nature of the measures used is explained in more detail in the Methods.

Category	Name	Studied in mouse	Studied in human	Measure	Reference
DNA conformation	A-phased repeat	H	H	Content	[65]
	Direct repeat	H	H	Content	[65]
	G-quadruplex repeat	H	H	Content	[65]
	Inverted repeat	H	H	Content	[65]
	Mirror repeat	H	H	Content	[65]
	Z-DNA repeat	H	H	Content	[65]
	Mononucleotide STR	H	H	Content	Genome-wide screening
	Dinucleotide STR	H	H	Content	Genome-wide screening
	Trinucleotide STR	H	H	Content	Genome-wide screening
	Tetranucleotide STR	H	H	Content	Genome-wide screening
DNA sequence	SINE	H	H	Content	UCSC Genome Browser
	LINE	H	H	Content	UCSC Genome Browser
	L1 target sites	H	H	Count	Genome-wide screening
	AT nucleotides	H	H	Count	Genome-wide screening
	CG nucleotides	H	H	Count	Genome-wide screening
Position on the chromosome	Distance to centromere	L	L	Distance in bp	Genome-wide screening
	Distance to telomere	L	L	Distance in bp	Genome-wide screening
Recombination	Recombination hotspot motif (13-mer instability)		H	Count	Genome-wide screening [66]
	Recombination hotspots	H	H	Content	[67–69]
	Recombination rate	H, L	L	Weighted average	[67,69–71]
Replication	ESC replication timing	L	L	Weighted average	DECODEdcc, UCSC Genome Browser
	Replication origins		H	Content	[72]
Methylation	Methylated CpG	H	H	Weighted average	[73–74]
	Methylated CHG	H	H	Weighted average	[73–74]
	Methylated CHH	H	H	Weighted average	[73–74]
	Not methylated CpG	H		Content	[74]
	Not methylated CHG	H		Content	[74]
	Not methylated CHH	H		Content	[74]
	Sperm hypomethylation		H	Content	[75]
Gene expression	Testis gene expression	2 H	H	Weighted average	[76–77]
	ESC gene expression	H	H	Weighted average	DECODEdcc, [77]
	H1-hESC exon expression		H	Weighted average	DECODEdcc
	H1-hESC transcript expression		H	Weighted average	DECODEdcc
	Transcription start sites	H	2 H	Content	ENCODE, [78]
Chromatin openness/ modifications	Dnase I hypersensitive sites	H	H	Content	UCSC Genome Browser
	H3K27ac—enhancers	H	H	Content	ENCODE *
	H3K27me3—repressed chromatin	H	H	Content	ENCODE *
	H3K36me3—transcribed chromatin	H	H	Content	ENCODE *
	H3K4me1—enhancers	H	H	Content	ENCODE *
	H3K4me3—promoters	H	H	Content	ENCODE *
	H3K9ac—transcription activation	H	H	Content	ENCODE *
	H3K9me3—repressed chromatin	H	H	Content	ENCODE *
Selection	CpG islands	H	H	Content	UCSC Genome Browser
	Exon	H	H	Content	UCSC Genome Browser
	Intron	H	H	Content	UCSC Genome Browser
	Most conserved elements	H	H	Content	UCSC Genome Browser
Total number features		41	43		

* Mouse ES Bruce4 C57BL6, human H1-ESC.

### Analysis overview

To identify genomic features significantly affecting the ERV distributions in the human and mouse genomes, we contrasted flanking regions of fixed ERVs (either mouse ETn and IAP, or human HERV-K) vs. control regions; such a comparison is expected to reflect both integration and fixation preferences (Fig 1B). In an attempt to disentangle genomic features affecting ERV integration from those affecting their fixation, we conducted additional comparisons; namely, we contrasted flanking regions of polymorphic mouse ERVs (ETn and IAP) vs. mouse control regions, and flanking regions of *in vitro* HERV-K vs. human control regions. In these comparisons integration preferences are expected to be substantially more prominent than fixation preferences because selection had substantially less time to act on polymorphic or *in vitro* ERVs than on fixed ERVs. Finally, to pinpoint genomic features significant for ERV fixation, we contrasted flanking regions of fixed vs. polymorphic mouse elements (ETns and IAPs), and of fixed vs. *in vitro* HERV-Ks. In a way, the analysis of fixed ERVs vs. controls can be viewed as “cumulating” that of polymorphic or *in vitro* ERVs vs. controls, and that of fixed vs. polymorphic or *in vitro* ERVs. In total, we conducted nine comparisons (Fig 1B), each using four different statistical techniques as described below (Fig 1C). Admittedly, polymorphic integrations are affected by selection to a greater degree than *in vitro* ones, however we are not in possession of both of these data types for the species in our study; only polymorphic data are available for mouse ERVs and only *in vitro* data are available for human ERVs.

**Fig 2:**
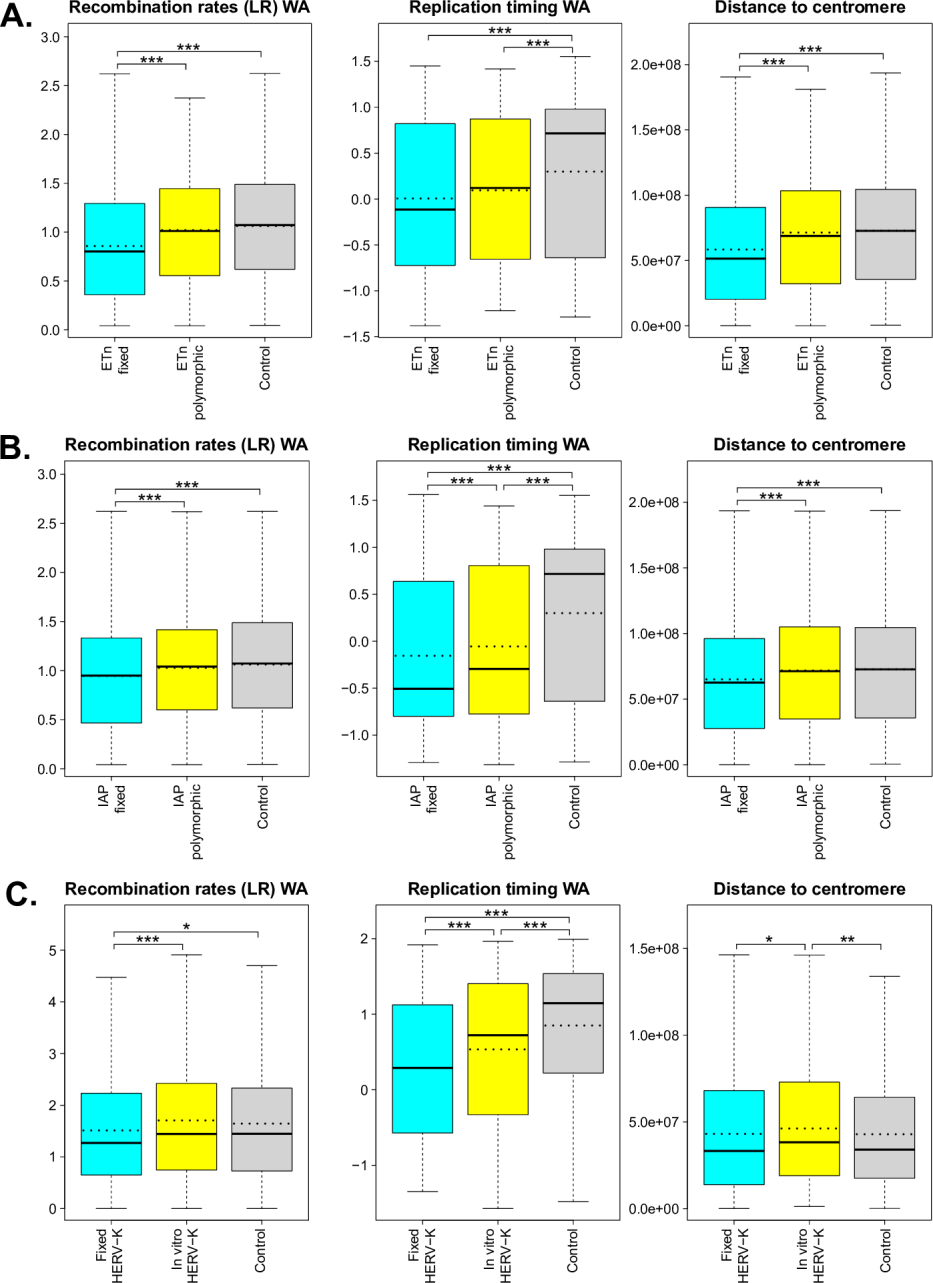
Boxplots of significant low-resolution features (i.e. recombination rates, replication timing, and distance to centromere) measured for the flanking regions of fixed and polymorphic (or *in vitro*) ERVs, and for control regions. The ERVs considered are: (A) ETns, (B) IAPs, and (C) HERV-Ks. Dotted lines represent means (solid lines in the boxplots are medians). Univariate permutation tests for the mean differences are summarized with asterisks above the corresponding comparisons (p-value <0.001 ‘***’, p-value <0.01 ‘**’, and p-value <0.05 ‘*’). Distance to telomere was also tested but was not found to have significant mean differences. WA—weighted average. More details can be found in Table C in S1 Text.

**Fig 3:**
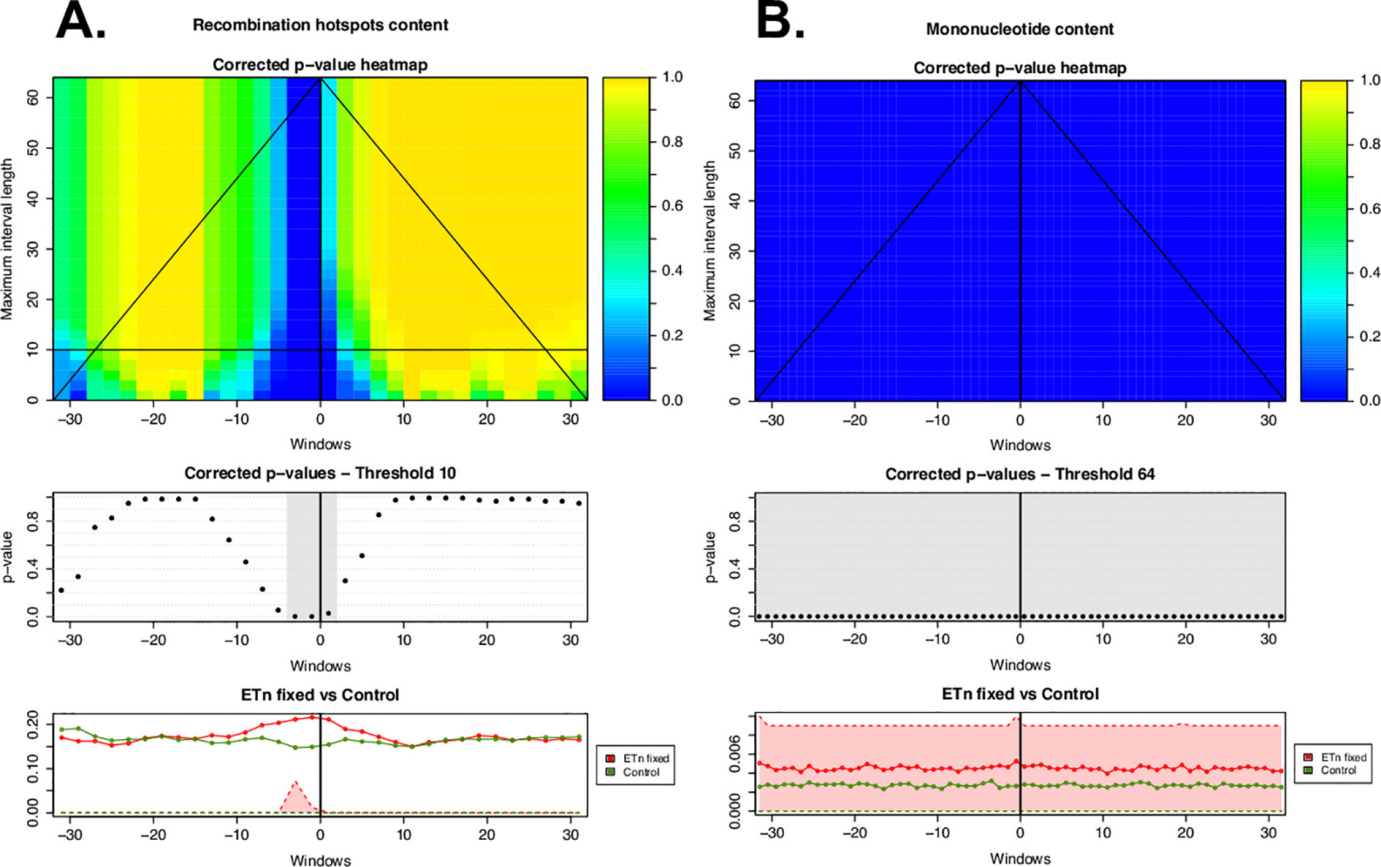
**ITP results using the mean difference as test statistics for (A) recombination hotspots (localized differential landscape–LDL) and (B) mononucleotide microsatellites (invariant differential landscape–IDL) in the flanking regions of fixed ETn vs. controls.** The heatmap in the top panel shows the p-values for each component (i.e. window; horizontal axis) corrected controlling the family-wise error rate on all possible maximum interval lengths (vertical axis). Blue corresponds to low p-values, hence significant differences between the distributions underlying the flanking regions of ERVs and the controls. The middle panel shows corrected p-values at the chosen maximum interval length threshold, with gray highlighting significant components (corrected p-values<0.05). The lower panels show the average of the genome feature under consideration over the flanking regions of all fixed ETns (red line) and controls (green line). First and third quartiles (25% and 75% quantiles) are shaded in the respective colors–red for ETns and green for controls. The shades for control are invisible because they are zeros for control. The heatmap suggests the scales (vertical axis) and the locations (horizontal axis) at which the feature is significant to characterize ERVs genomic landscape.

**Fig 4:**
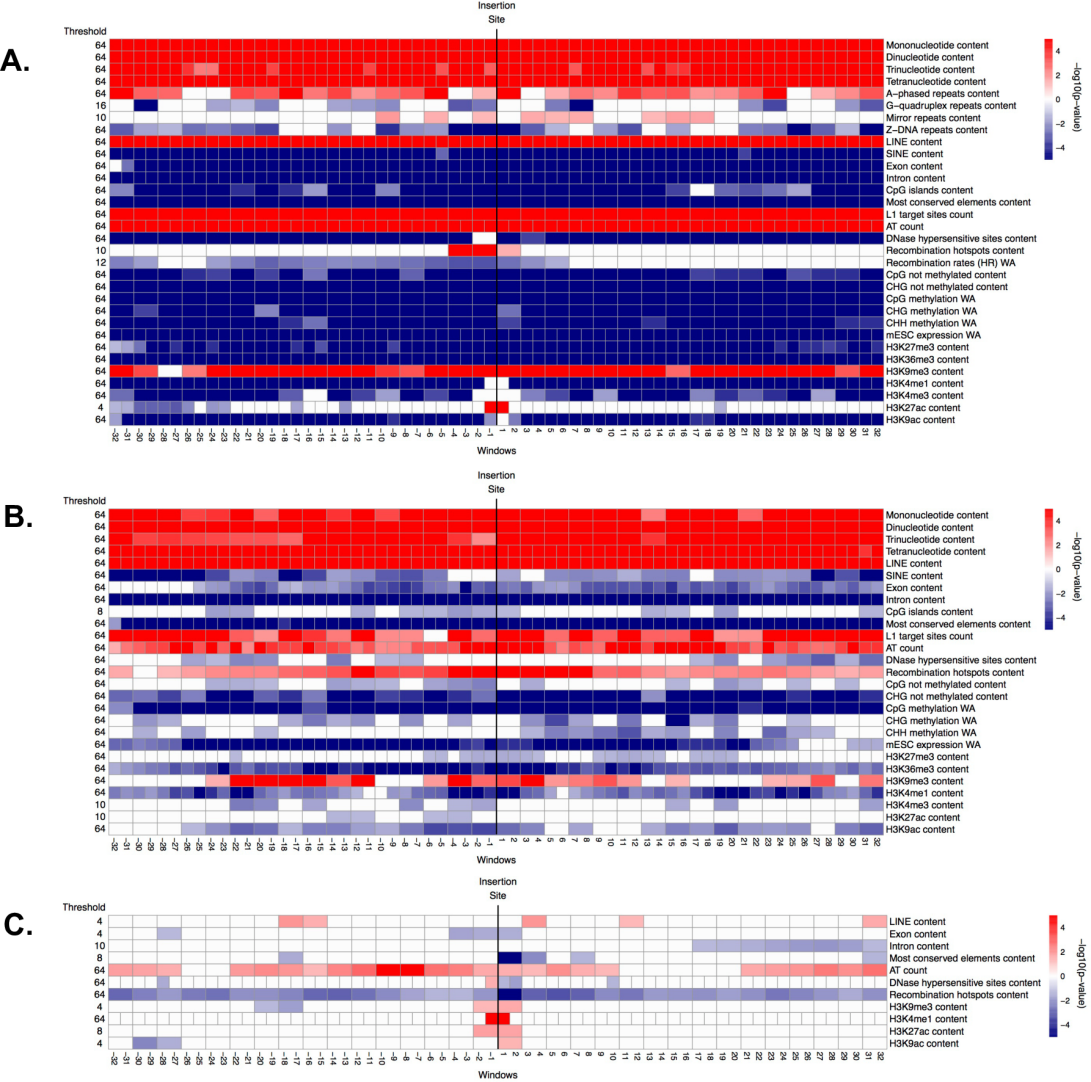
**Significance (i.e. -log10(corrected p-value)) of genomic features in windows along the flanking regions, obtained from the ITP using the mean difference as test statistics: (A) fixed ETns vs. controls, (B) polymorphic ETns vs. controls, and (C) fixed vs. polymorphic ETns.** In each panel, the horizontal axis represents the 64 1-kb windows. The vertical black line between window -1 kb and 1 kb marks the integration site. The thresholds reported on the left represent the maximum scale at which each feature is significant, ranging from 64 kb (coarsest) to 1 kb (finest). Each row corresponds to one feature and each cell represents one or two contiguous windows, depending on the number of nodes employed in the B-splines (we consider one value for every 1-kb window when using the raw data, and one value every two 1-kb windows when using the piecewise constant smoothed version of the data). White cells: not significant (p-value >0.05), red cells: significant with higher mean in the flanking regions of ETns vs. controls (or in the flanking regions of fixed vs. polymorphic ETns), blue cells: significant with lower mean in the flanking regions of ETns vs. controls (or in the flanking regions of fixed vs. polymorphic ETns). Color intensity is proportional to significance (more intense colors correspond to lower corrected p-values).

**Fig 5:**
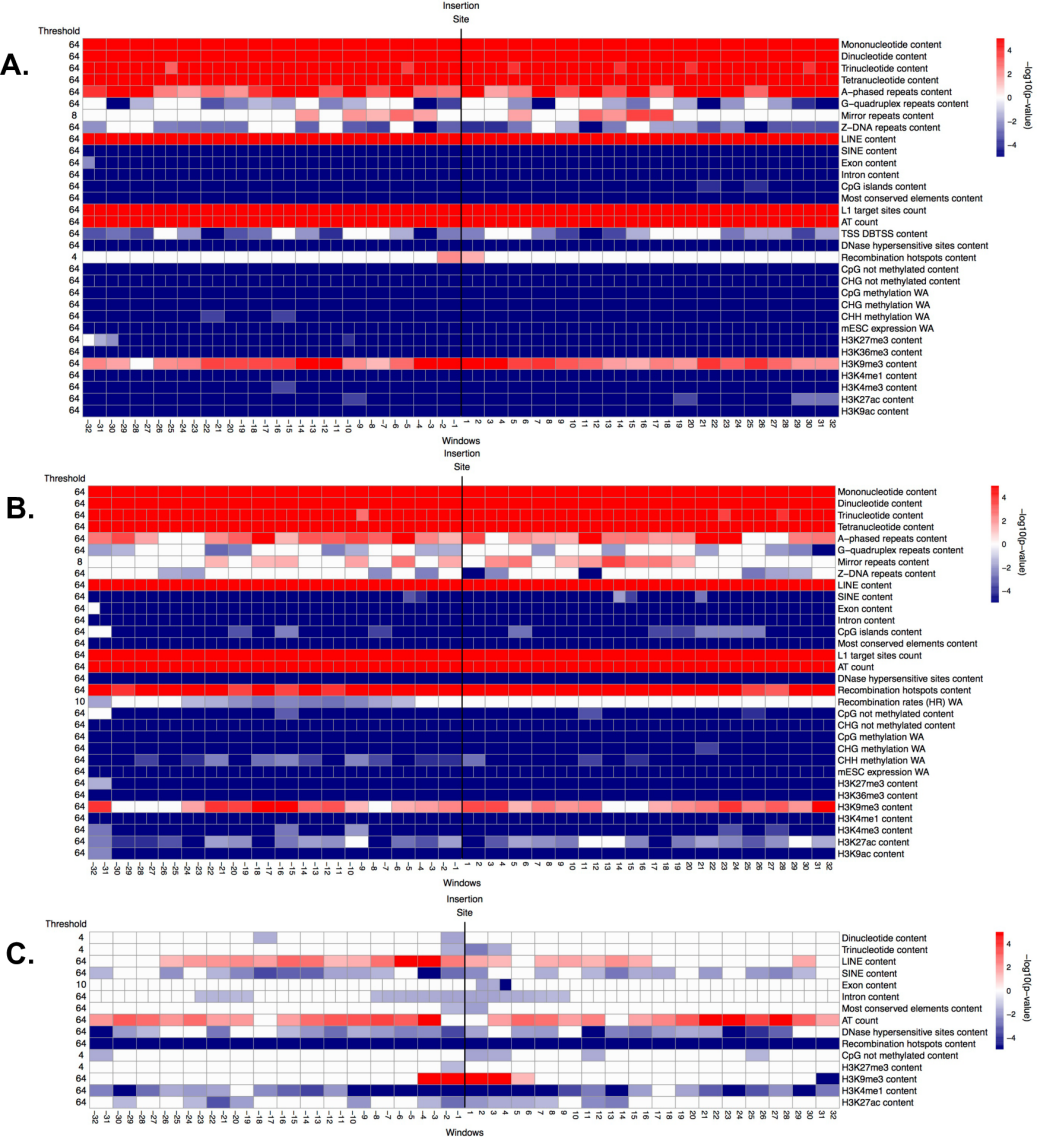
**Significance (i.e. -log10(corrected p-value)) of genomic features in windows along the flanking regions, obtained from the ITP using the mean difference as test statistics: (A) fixed IAPs vs. controls, (B) polymorphic IAPs vs. controls, and (C) fixed vs. polymorphic IAPs.** See explanations for Fig 4.

**Fig 6:**
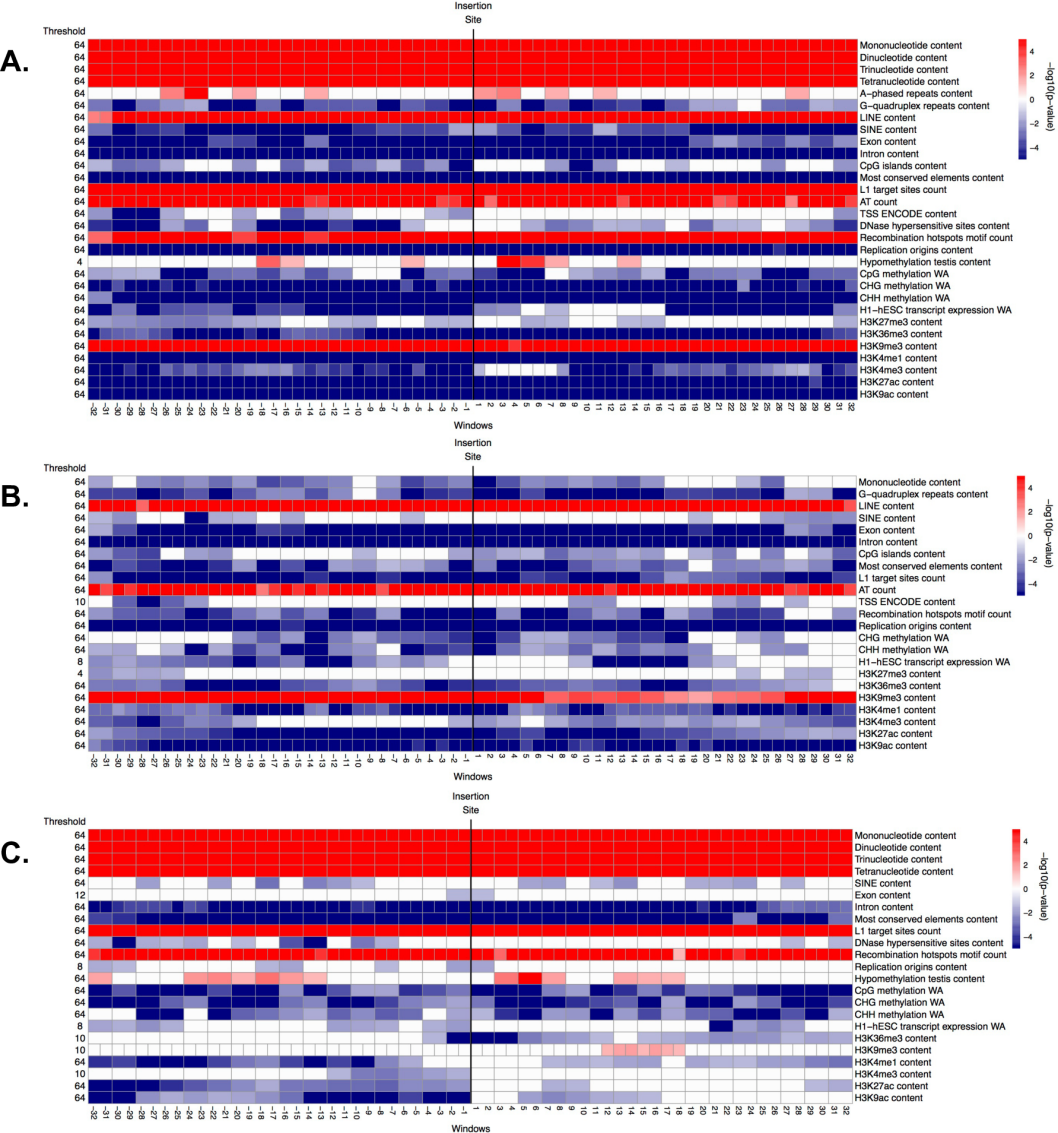
**Significance (i.e. -log10(corrected p-value)) of genomic features in windows along the flanking regions, obtained from the ITP using the mean difference as test statistics: (A) fixed HERV-Ks vs. controls, (B) *in vitro* HERV-Ks vs. controls, and (C) fixed vs. *in vitro* HERV-Ks.** See explanations for Fig 4.

*First*, we tested whether ERV presence was significantly affected by low-resolution features using a univariate permutation test (see Methods, Table B in S1 Text; Fig 2); this is appropriate because these features are represented by a single value for each 64-kb region. *Second*, for the high-resolution genomic features, we employed the two-population Interval Testing Procedure (ITP) for functional data [62] to assess whether each feature, when considered alone, had significantly different content (or count, or WA) in a comparison, e.g. in ERV flanking regions vs. controls (see Methods for details). This technique is particularly suitable for our analysis because it considers the data as a curve over the 64 1-kb windows comprising each region, instead of taking one value for the region (e.g., an average over the 64 windows). ITP combines inference on the whole curve with component-wise inference (i.e. inference on measurements comprising the curve). Thus, it allows us to select relevant genomic features detecting both the scale and the location at which each feature acts (see Methods for more details). From this analysis we expect to detect genomic features that: (1) show significant enrichment/depletion locally, especially in windows close to the IntS of ERVs or further away from it–we call these *localized differential landscape* (LDL) features (e.g. Fig 3A); (2) show a uniform level of significant enrichment/depletion throughout all 64 1-kb windows–we call these *invariant differential landscape* (IDL) features (e.g. Fig 3B); or (3) are not significant over the whole 64-kb region examined. In order to capture different nuances of the data, we performed ITP using three test statistics (mean difference, median difference, and variance ratio; Figs 4–6 and F-T in S1 Text), however, below we focus on results concerning mean differences.

**Fig 7:**
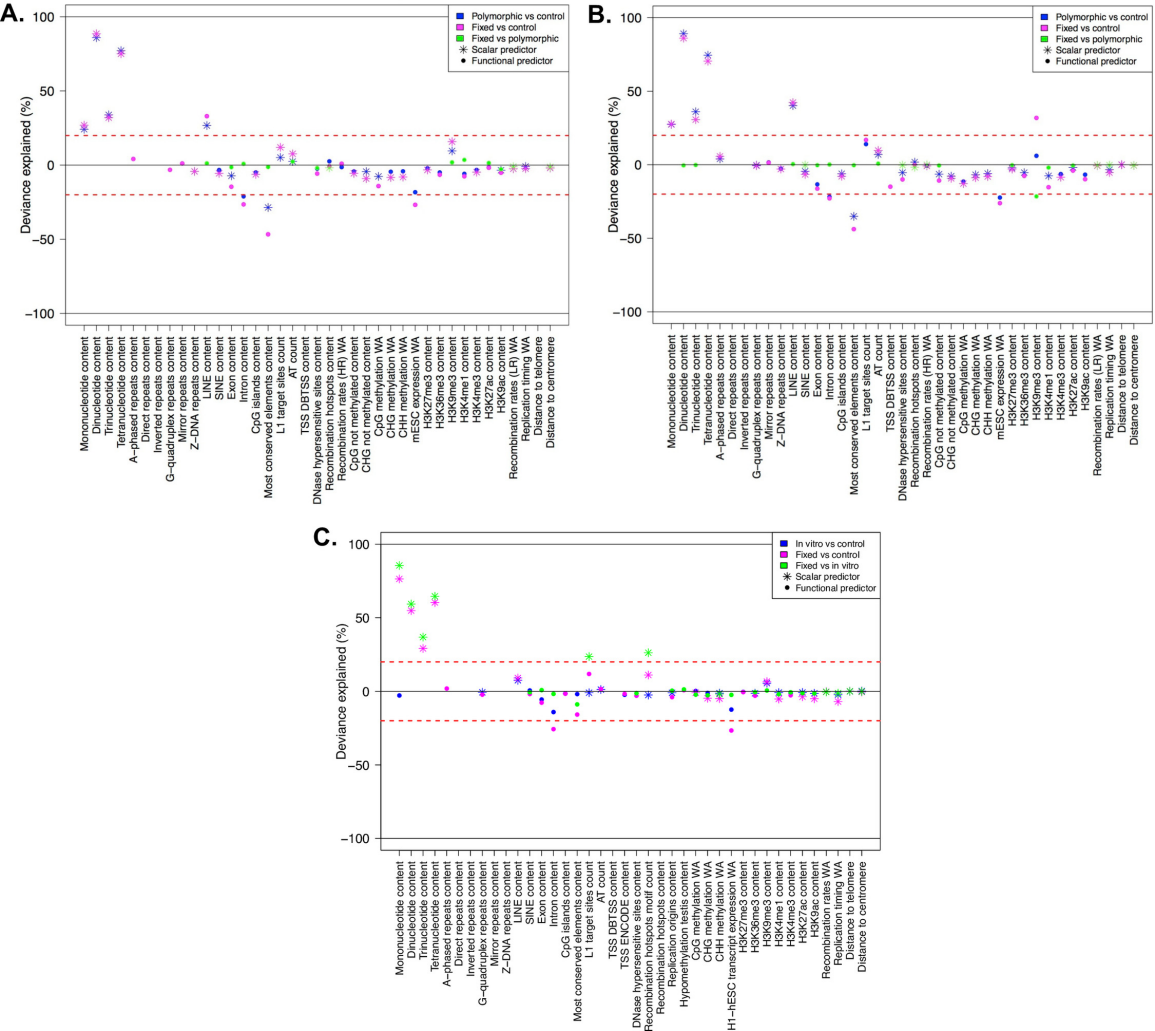
**Deviance explained by individual predictors in single logistic regression fits, concerning (A) ETns, (B) IAPs, and (C) HERV-Ks.** Each panel contains color-coded results from three comparisons–magenta: fixed vs. controls, blue: polymorphic (or *in vitro*) vs controls, green: fixed vs. polymorphic (or *in vitro*). Only features that resulted significant in the ITP are considered here as scalar or functional predictors (indicated with stars and points, respectively), depending on whether they were IDL or LDL features. The deviance explained (between 0 and 100%) is reported as positive or negative depending on the sign of the estimated predictor coefficient (the predominant sign in the case of functional predictors). The red lines at ±20% represent the threshold above (below) which “dominant” predictors were omitted from the subsequent multiple FLR modeling.

*Third*, to determine the individual explanatory power of major predictors, we fitted single Functional Logistic Regressions (FLRs; Figs 1C and 7, Table C in S1 Text) for each feature found to be significant in ITP. This analysis allowed us to summarize and better quantify the results obtained by univariate permutation test and ITP (Fig 7). Moreover, it allowed us to identify features that, by themselves, explained a percent of deviance in excess of 20%. These are clearly very relevant predictors (Table C in S1 Text) but we did *not* include them in our final multiple FLR models (see below) as they would hide the concurrent effects of other potentially relevant predictors.

*Fourth*, we examined the joint effects of the remaining significant predictors (as determined by ITP and univariate permutation test) via multiple Functional Logistic Regression (Fig 1C and Tables 3–11). The multiple FLR models expressed the likelihood of being in the neighborhood of an ERV vs. control (or of a fixed vs. a polymorphic mouse ERV, or of a fixed vs. *in vitro* HERV-K) as a joint function of several predictors. In particular, IDL features and low-resolution features that proved significant in univariate permutation tests were treated as *scalar predictors* represented by their averages across the 64 windows constituting each region. In contrast, LDL features were treated as *functional predictors* with curves evaluated at customized scales and intervals to capture the specific behavior of each LDL feature, e.g. around the IntS, as suggested by the ITP. Importantly, the modified ITP we employed (see Methods) gave us detailed information on the best scale and location, i.e. on the subregions on which to study the curve, for each of these functional predictors.

**Table 3:** Multiple FLR models for fixed ETn vs. control. The "Predictor" column reports predictors included in the logistic regression. The "Coefficient" column reports coefficient estimates (a positive coefficient means that an increase in the feature increases the likelihood of, e.g., fixed vs. control; a negative coefficient means an increase in the feature decreases such likelihood). The "p-value" column reports p-values for the coefficients. They both are in bold if p-value<0.05. For functional predictors, several rows are listed corresponding to the intervals where the feature was considered—as indicated in the "Range of windows" column. The "RCDE" column reports the relative contribution to the deviance explained for each feature. "DE" at the bottom of each panel is the total deviance explained by the model.

Predictor	Range of windows	Coefficient	p-value	RCDE (%)
Z-DNA repeats content	scalar	**-1.3E+00**	**1.0E-10**	2.6
SINE content	scalar	**-4.9E-01**	**5.6E-04**	0.7
L1 target sites count	scalar	**4.4E+01**	**2.0E-16**	23.5
AT count	scalar	**-7.4E-02**	**2.0E-16**	17.8
Recombination rates LR WA	scalar	**-8.7E-01**	**9.5E-05**	0.9
CHG not methylated content	scalar	**2.0E+01**	**2.0E-09**	2.2
H3K27me3 content	scalar	**-3.0E-01**	**2.0E-16**	4.7
H3K9me3 content	scalar	**5.7E-01**	**2.0E-16**	20.5
CpG methylation WA	(-30,-10)	**-2.0E-01**	**2.3E-12**	10.8
	(-10,10)	**-2.0E-01**	**4.1E-12**	
	(10,30)	**-2.0E-01**	**1.3E-12**	
Exon content	(-28,-20)	**-1.3E-02**	**3.7E-02**	9.7
	(-20,-12)	-3.0E-03	6.3E-01	
	(-12,-4)	1.7E-03	8.1E-01	
	(-4,4)	**-5.2E-02**	**1.6E-11**	
	(4,12)	-9.7E-03	1.5E-01	
	(12,20)	-1.1E-02	9.3E-02	
	(20,28)	**-1.4E-02**	**1.9E-02**	
H3K4me1 content	(-28,-20)	**-1.9E-02**	**4.6E-04**	5.1
	(-20,-12)	-8.5E-03	1.3E-01	
	(-12,-4)	**-1.6E-02**	**3.4E-03**	
	(-4,4)	**2.6E-02**	**7.2E-07**	
	(4,12)	**-1.7E-02**	**2.8E-03**	
	(12,20)	-3.4E-03	5.2E-01	
	(20,28)	**-1.4E-02**	**5.7E-03**	
Mirror repeats content	(-32,-16)	**3.5E-02**	**1.2E-02**	3.1
	(-16,0)	**5.3E-02**	**7.5E-05**	
	(0,16)	**5.7E-02**	**3.1E-05**	
	(16,32)	**3.3E-02**	**1.3E-02**	
**DE (%)**	**51.1**			

**Table 4:** Multiple FLR models for polymorphic ETn vs. control. See explanations for Table 3.

Predictor	Range of windows	Coefficient	p-value	RCDE (%)
Exon content	scalar	**-3.0E-01**	**2.5E-02**	0.9
L1 target sites count	scalar	**5.1E+01**	**2.0E-16**	34.3
AT count	scalar	**-6.4E-02**	**2.0E-16**	21.9
CHG not methylated content	scalar	**2.2E+01**	**1.4E-05**	3.2
CpG methylation WA	scalar	**-1.1E+01**	**8.5E-14**	10.9
H3K9me3 content	scalar	**4.3E-01**	**1.9E-14**	10.8
mESC expression WA	(-30,-10)	**-9.2E-03**	**2.5E-02**	29.2
	(-10,10)	**-2.5E-02**	**3.9E-06**	
	(10,30)	-2.5E-03	5.4E-01	
H3K27me3 content	(-30,-10)	**-3.3E-02**	**2.7E-03**	10.9
	(-10,10)	**-2.7E-02**	**3.8E-03**	
	(10,30)	**-1.9E-02**	**2.0E-02**	
Recombination hotspots content	(-30,-10)	4.7E-03	1.3E-01	2.4
	(-10,10)	**6.8E-03**	**4.6E-02**	
	(10,30)	-2.4E-03	4.6E-01	
**DE (%)**	**48.4**			

**Table 5:** Multiple FLR models for fixed vs. polymorphic ETn. See explanations for Table 3.

Predictor	Range of windows	Coefficient	p-value	RCDE (%)
Recombination hotspots content	scalar	**-4.2E-01**	**5.2E-05**	8.7
Distance to centromere	scalar	**-5.7E-09**	**2.3E-03**	4.9
H3K4me1 content	(-28,-20)	1.6E-02	8.9E-02	25.3
	(-20,-12)	**-2.3E-02**	**2.6E-02**	
	(-12,-4)	**-3.3E-02**	**7.8E-04**	
	(-4,4)	**4.9E-02**	**1.9E-06**	
	(4,12)	**-2.3E-02**	**1.4E-02**	
	(12,20)	1.3E-02	2.0E-01	
	(20,28)	1.3E-02	2.0E-01	
H3K9ac content	(-28,-20)	-1.4E-02	3.7E-01	14.9
	(-20,-12)	3.1E-02	9.7E-02	
	(-12,-4)	1.5E-02	4.2E-01	
	(-4,4)	**5.7E-02**	**6.9E-03**	
	(4,12)	**-4.3E-02**	**2.8E-03**	
	(12,20)	**4.5E-02**	**6.8E-03**	
	(20,28)	-1.5E-02	2.9E-01	
H3K9me3 content	(-28,-20)	1.3E-02	4.4E-01	16.4
	(-20,-12)	**-3.6E-02**	**1.8E-02**	
	(-12,-4)	-7.2E-03	6.8E-01	
	(-4,4)	**4.7E-02**	**9.1E-05**	
	(4,12)	-1.8E-02	2.6E-01	
	(12,20)	2.0E-02	2.5E-01	
	(20,28)	4.0E-02	6.7E-02	
DNase hypersensitive sites content	(-30,-26)	**-9.3E-02**	**2.0E-02**	15.1
	(-26,-22)	-1.2E-02	7.8E-01	
	(-22,-18)	6.7E-03	8.8E-01	
	(-18,-14)	-4.2E-02	3.7E-01	
	(-14,-10)	3.6E-02	4.1E-01	
	(-10,-6)	1.0E-03	9.8E-01	
	(-6,-2)	-5.4E-02	1.9E-01	
	(-2,2)	-2.2E-02	5.8E-01	
	(2,6)	-7.1E-02	8.3E-02	
	(6,10)	-1.2E-02	7.9E-01	
	(10,14)	**-9.5E-02**	**3.2E-02**	
	(14,18)	-1.1E-02	8.2E-01	
	(18,22)	-2.3E-02	6.1E-01	
	(22,26)	8.7E-03	8.5E-01	
	(26,30)	-3.4E-03	9.3E-01	
H3K27ac content	(-28,-20)	1.4E-02	4.2E-01	8.1
	(-20,-12)	1.4E-02	4.0E-01	
	(-12,-4)	2.2E-02	1.6E-01	
	(-4,4)	2.0E-02	1.8E-01	
	(4,12)	3.0E-02	6.2E-02	
	(12,20)	**-3.1E-02**	**3.3E-02**	
	(20,28)	1.9E-03	9.0E-01	
**DE (%)**	**15.0**			

**Table 6:** Multiple FLR models for fixed IAP vs. control. See explanations for Table 3.

Predictor	Range of windows	Coefficient	p-value	RCDE (%)
CpG methylation WA	scalar	**-1.1E+00**	**1.2E-03**	0.6
H3K27me3 content	scalar	**-2.6E-01**	**2.0E-16**	5.5
H3K4me3 content	scalar	**3.5E-01**	**1.2E-03**	0.6
TSS DBTSS content	(-32,-24)	**-1.9E-02**	**3.1E-04**	11.1
	(-24,-16)	**-1.5E-02**	**2.3E-03**	
	(-16,-8)	-2.9E-03	5.4E-01	
	(-8,0)	**-1.8E-02**	**2.3E-04**	
	(0,8)	**-1.4E-02**	**3.1E-03**	
	(8,16)	**-2.1E-02**	**1.1E-05**	
	(16,24)	**-1.1E-02**	**2.7E-02**	
	(24,32)	**-1.5E-02**	**5.7E-04**	
Exon content	(-28,-20)	2.4E-03	5.0E-01	7.6
	(-20,-12)	**-1.0E-02**	**5.8E-03**	
	(-12,-4)	1.0E-03	8.0E-01	
	(-4,4)	**-3.2E-02**	**3.4E-14**	
	(4,12)	-3.4E-03	4.0E-01	
	(12,20)	-5.2E-03	1.9E-01	
	(20,28)	**-6.8E-03**	**4.7E-02**	
H3K4me1 content	(-28,-20)	**-7.0E-03**	**1.6E-02**	9.4
	(-20,-12)	4.2E-04	8.9E-01	
	(-12,-4)	-4.4E-03	1.7E-01	
	(-4,4)	**-3.3E-02**	**2.0E-16**	
	(4,12)	-3.8E-03	2.4E-01	
	(12,20)	-2.4E-05	9.9E-01	
	(20,28)	**-5.9E-03**	**4.7E-02**	
L1 target sites count	(-30,-10)	**5.4E-02**	**7.1E-06**	8.6
	(-10,10)	**5.3E-02**	**2.2E-05**	
	(10,30)	**5.1E-02**	**1.0E-05**	
DNase hypersensitive sites content	(-28,-20)	4.0E-03	5.0E-01	5.1
	(-20,-12)	**2.1E-02**	**1.1E-03**	
	(-12,-4)	**2.2E-02**	**6.6E-04**	
	(-4,4)	6.8E-03	2.7E-01	
	(4,12)	**1.9E-02**	**3.8E-03**	
	(12,20)	**1.9E-02**	**2.3E-03**	
	(20,28)	**1.6E-02**	**1.0E-02**	
H3K36me3 content	(-30,-10)	6.8E-04	7.3E-01	3.5
	(-10,10)	**-1.6E-02**	**2.2E-11**	
	(10,30)	**5.4E-03**	**8.8E-03**	
**DE (%)**	**33.3**			

**Table 7:** Multiple FLR models for polymorphic IAP vs. control. See explanations for Table 3.

Predictor	Range of windows	Coefficient	p-value	RCDE (%)
Recombination hotspots content	scalar	**3.2E-01**	**8.3E-07**	2.1
CHG methylation WA	scalar	**7.6E+00**	**4.5E-05**	1.4
Replication timing WA	scalar	**8.0E-01**	**2.0E-16**	6.6
H3K27me3 content	scalar	**-1.2E-01**	**8.8E-07**	2.1
H3K36me3 content	scalar	**-9.4E-02**	**1.9E-05**	1.5
H3K4me1 content	scalar	**-1.5E-01**	**7.0E-03**	0.6
L1 target sites count	(-30,-10)	**6.3E-02**	**1.6E-07**	13.5
	(-10,10)	**5.0E-02**	**5.4E-05**	
	(10,30)	**4.3E-02**	**1.7E-04**	
H3K9me3 content	(-28,-20)	**6.8E-02**	**7.6E-05**	16.1
	(-20,-12)	**1.4E-01**	**7.7E-08**	
	(-12,-4)	**7.1E-02**	**2.0E-04**	
	(-4,4)	**7.8E-02**	**2.0E-05**	
	(4,12)	**5.1E-02**	**8.6E-03**	
	(12,20)	**4.9E-02**	**1.9E-03**	
	(20,28)	**1.0E-01**	**7.2E-06**	
Exon content	(-28,-20)	**-9.8E-03**	**3.7E-02**	8.9
	(-20,-12)	-5.6E-03	2.6E-01	
	(-12,-4)	3.2E-03	5.4E-01	
	(-4,4)	**-3.6E-02**	**3.2E-10**	
	(4,12)	-6.4E-04	9.0E-01	
	(12,20)	-8.2E-03	1.2E-01	
	(20,28)	-6.0E-03	1.9E-01	
CpG methylation WA	(-28,-20)	**-1.2E-01**	**5.3E-04**	6.1
	(-20,-12)	3.0E-02	4.0E-01	
	(-12,-4)	**-7.6E-02**	**2.9E-02**	
	(-4,4)	-1.3E-02	7.0E-01	
	(4,12)	**-1.1E-01**	**2.0E-03**	
	(12,20)	**-1.1E-01**	**1.7E-03**	
	(20,28)	**-8.7E-02**	**1.2E-02**	
**DE (%)**	**30.4**			

**Table 8:** Multiple FLR models for fixed vs. polymorphic IAP. See explanations for Table 3.

Predictor	Range of windows	Coefficient	p-value	RCDE (%)
Recombination hotspots content	scalar	**-4.1E-01**	**2.0E-16**	42.2
Recombination rates LR WA	scalar	**-1.9E+00**	**1.3E-03**	4.2
H3K4me1 content	(-28,-20)	-6.7E-04	7.5E-01	47.6
	(-20,-12)	1.6E-03	4.6E-01	
	(-12,-4)	4.9E-04	8.2E-01	
	(-4,4)	**-2.4E-02**	**2.0E-16**	
	(4,12)	9.2E-04	6.6E-01	
	(12,20)	1.2E-03	5.6E-01	
	(20,28)	-2.1E-03	2.8E-01	
**DE (%)**	**3.8**			

**Table 9:** Multiple FLR models for fixed HERV-K vs. control. See explanations for Table 3.

Predictor	Range of windows	Coefficient	p-value	RCDE (%)
LINE content	scalar	**3.6E+00**	**2.0E-16**	13.33
Recombination hotspots motif count	scalar	**3.0E+00**	**2.0E-16**	9.68
Replication timing WA	scalar	**-8.3E-01**	**4.1E-07**	1.12
CHG methylation WA	scalar	**-7.8E+00**	**1.5E-04**	0.64
H3K9me3 content	scalar	**1.9E-01**	**1.9E-07**	1.17
H3K4me1 content	scalar	**-3.6E-01**	**1.9E-04**	0.58
H3K9ac content	scalar	**-3.1E-01**	**9.1E-04**	0.46
L1 target sites count	(-30,-10)	**3.2E-01**	**2.0E-16**	27.09
	(-10,10)	**2.6E-01**	**1.6E-14**	
	(10,30)	**3.4E-01**	**2.0E-16**	
AT count	(-30,-10)	**-1.9E-03**	**8.5E-13**	12.61
	(-10,10)	**-1.5E-03**	**5.4E-08**	
	(10,30)	**-2.1E-03**	**5.5E-16**	
G-quadruplex repeats content	(-32,-16)	**-9.7E-02**	**1.8E-02**	1.89
	(-16,0)	**-1.5E-01**	**2.4E-04**	
	(0,16)	**-1.1E-01**	**5.2E-03**	
	(16,32)	-3.0E-02	4.5E-01	
Hypomethylation testis content	(-32,-16)	**3.7E-02**	**2.6E-06**	5.37
	(-16,0)	**3.9E-02**	**2.4E-07**	
	(0,16)	**3.3E-02**	**5.0E-05**	
	(16,32)	**1.8E-02**	**1.8E-02**	
CpG islands content	(-32,0)	**-5.7E-02**	**2.9E-13**	5.22
	(0,32)	**-3.1E-02**	**7.2E-06**	
**DE (%)**	**79.0**			

**Table 10:** Multiple FLR models for *in vitro* HERV-K vs. control. See explanations for Table 3.

Predictor	Range of windows	Coefficient	p-value	RCDE (%)
L1 target sites count	scalar	**-2.8E+00**	**2.0E-16**	11.0
AT count	scalar	**1.6E-02**	**5.9E-15**	7.7
Recombination hotspots motif count	scalar	**-2.9E-01**	**4.9E-04**	1.5
CHH methylation WA	scalar	**-3.4E+00**	**5.0E-03**	0.9
H3K9me3 content	scalar	**1.3E-01**	**5.3E-14**	6.8
Distance to telomere	scalar	**-4.5E-09**	**3.0E-02**	0.6
Intron content	(-32,-16)	**-4.6E-02**	**1.9E-05**	29.2
	(-16,0)	**-3.7E-02**	**4.0E-03**	
	(0,16)	**-3.8E-02**	**3.9E-03**	
	(16,32)	**-3.5E-02**	**8.0E-04**	
CpG methylation WA	(-32,-16)	**1.6E-01**	**6.0E-03**	4.6
	(-16,0)	8.8E-02	1.2E-01	
	(0,16)	**1.4E-01**	**2.0E-02**	
	(16,32)	**1.5E-01**	**4.5E-03**	
**DE (%)**	**24.4**			

**Table 11:** Multiple FLR models for fixed vs. *in vitro* HERV-K. See explanations for Table 3.

Predictor	Range of windows	Coefficient	p-value	RCDE (%)
Replication timing WA	scalar	**-1.7E-01**	**1.0E-02**	2.2
CHH methylation WA	scalar	**-3.9E+00**	**3.5E-05**	5.8
Most conserved elements content	(-30,-10)	**-1.2E-02**	**2.3E-04**	56.1
	(-10,10)	**-2.6E-02**	**9.2E-13**	
	(10,30)	-2.7E-03	4.0E-01	
Exon content	(-28,-20)	1.1E-02	9.4E-02	10.3
	(-20,-12)	**1.6E-02**	**1.8E-02**	
	(-12,-4)	4.7E-03	5.0E-01	
	(-4,4)	-1.5E-02	5.4E-02	
	(4,12)	3.8E-03	5.9E-01	
	(12,20)	5.2E-03	4.5E-01	
	(20,28)	**1.3E-02**	**3.4E-02**	
H3K4me1 content	(-28,-20)	**-1.6E-02**	**2.9E-02**	9.1
	(-20,-12)	4.0E-04	9.6E-01	
	(-12,-4)	**-2.3E-02**	**5.3E-03**	
	(-4,4)	**2.5E-02**	**1.2E-03**	
	(4,12)	-6.6E-03	3.7E-01	
	(12,20)	-5.5E-03	4.8E-01	
	(20,28)	-3.7E-03	5.9E-01	
**DE (%)**	**12.1**			

### ETns

To identify genomic features affecting the distribution of ETns in the mouse genome–as a result of both integration and fixation preferences of these elements–we contrasted flanking sequences of fixed ETns vs. control regions. Univariate permutation tests applied to the low-resolution features (Fig 2A and Table B in S1 Text) indicated that the flanking regions of fixed ETns have lower recombination rates and later replication timing, and are closer to centromeres. ITP indicated (Fig 4A and Fig F in S1 Text) that all four microsatellites types, LINEs, L1 target sites, AT count, and the H3K9me3 histone mark are overrepresented, while SINEs, exons, introns, CpG islands, most conserved elements, all features associated with CpG methylation, ESC expression, and two histone marks (H3K27me3 and H3K36me3) are underrepresented, throughout the *whole* fixed ETn flanking regions. ITP also identified features with interesting localized behaviors: recombination hotspot content and the H3K27ac histone mark are overrepresented immediately next to the IntS, while DNase I hypersensitive sites and three histone marks (H3K4me1, H3K4me3, and H3K9ac) are underrepresented everywhere except for immediately next to the IntS. We found that also Z-DNA repeats and G-quadruplex repeats are underrepresented, with a yet more complex local behavior. Next, single FLRs revealed that all four microsatellites types, LINEs, introns, most conserved elements, and ESC expression have very strong effects (relative contribution to the deviance explained, RCDE ≥26%; Fig 7A and Table C in S1 Text). We therefore excluded these predictors from the final multiple FLR model, which explained 51.1% of the deviance in discriminating fixed ETns from controls (Table 3). The two strongest scalar predictors in such a model (i.e. H3K9me3 and L1 target sites, RCDE 20.5% and 23.5%, respectively) had positive effects, while AT count and H3K27me3 had negative effects (RCDE 17.8% and 4.7%, respectively; see Discussion for the explanation of the negative effect of AT count in multiple FLRs). The two strongest functional predictors–CpG methylation and exon content (RCDE of ~10%)–had negative effects. Interestingly, the H3K4me1 mark (RCDE 5.1%) had a strong positive effect strictly localized at the IntS (-4 to 4 kb) and a negative effect away from the IntS, while mirror repeats (RCDE 3.1%) had a positive effect on the whole region.

To highlight integration preferences, we contrasted flanking sequences of polymorphic ETns vs. control regions. In this analysis (Fig 4B and Fig G in S1 Text) we found many similarities but also a number of noteworthy differences with respect to the analysis of fixed ETns vs. controls (Fig 4A and Fig F in S1 Text). For instance, similar to fixed ETns, the flanking sequences of polymorphic ETns appeared to replicate later than controls (Table B in S1 Text and Fig 2A) suggesting that this feature might be important for ETn integration. The underrepresentation of exons, CpG islands, and several histone marks was weaker for polymorphic ETns vs. controls than for fixed ETns vs. controls suggesting that ETns can integrate but tend not to become fixed in such environments (Fig 4B and 4A). Moreover, the content of DNase I hypersensitive sites did not differ significantly from controls in a relatively large region surrounding the IntS for polymorphic ETns (-6 kb to +16 kb; Fig 4B) but only at 1 kb upstream from the IntS for fixed ETns (Fig 4A). Single FLRs displayed very similar explained deviances for microsatellites and LINEs in the two comparisons, while the explained deviances for intron content and most conserved elements content were lower for polymorphic ETns vs. controls than for fixed ETns vs. controls (Fig 7A, Tables 4 and 3)–confirming that the former are subject to weaker selection effects. The final multiple FLR model for polymorphic ETns vs. controls explained 48.4% of the deviance (Table 4) and was similar to the model for fixed ETns vs. controls (Table 3).

Next, to highlight fixation preferences, we contrasted flanking regions of fixed vs. polymorphic ETns. This analysis, too, revealed some similarities and some important differences relative to that of fixed ETn vs. controls. Fixed ETns were located in regions with lower recombination rates and closer to centromeres compared to polymorphic ETns (Table B in S1 Text and Fig 2A) suggesting that these features are important for fixation. The ITP tests contrasting fixed vs. polymorphic ETns (Fig 4C and Fig H in S1 Text) did not identify microsatellites as features differentiating fixation and integration propensities. However, they did reveal a more localized underrepresentation of exons and most conserved elements around the IntS (Fig 4C)–as compared to the ITP tests contrasting fixed ETns vs. controls (Fig 4A). Recombination hotspots, which were overrepresented in polymorphic ETns vs. controls (Fig 4B) and strongly overrepresented near the IntS in fixed ETns vs. controls (Fig 4A), were *underrepresented* in fixed vs. polymorphic ETns (Fig 4C). Also, several histone marks (H3K9me3, H3k4me1, and H3K9ac) were overrepresented near the IntS in fixed vs. polymorphic ETns (Fig 4C), but did not show a significant difference near the IntS in fixed ETn vs. controls (Fig 4A). Interestingly, DNase I hypersensitive sites were overrepresented -1 kb upstream and underrepresented up to 2 kb downstream from the IntS (Fig 4C). Single FLRs did not identify features which, individually, had great strength in characterizing fixed vs. polymorphic ETns (all explained deviances <3.5%) (Fig 7A and Table C in S1 Text). However, taken together in the context of multiple FLR, eight features explained 15% of the deviance in discriminating fixed vs. polymorphic ETns (Table 5) and reiterated most of our observations from the ITP tests (Fig 4C).

### IAPs

Interestingly, the genomic features significant in distinguishing between the flanking sequences of fixed IAP and control regions were very similar to those identified in the analogous comparison for ETns. For instance, just as fixed ETns (Table B in S1 Text and Fig 2A), fixed IAPs were found in regions with lower recombination, later replication, and smaller distance to the centromere than controls (Table B in S1 Text and Fig 2B). Most predictors found to be significant by the ITP tests were also shared between the fixed IAPs vs. controls and the fixed ETns vs. controls comparisons (Figs 5A and 4A, respectively). Several noteworthy exceptions included a uniform underrepresentation of H3K27ac throughout the 64 kb region flanking fixed IAPs (Fig 5A and Fig I in S1 Text), while this histone mark was *overrepresented* near the IntS of fixed ETns (Fig 4A and Fig H in S1 Text). Transcription start sites were underrepresented for fixed IAPs, while they were not significant for fixed ETns. Also, three histone marks (H3K4me1, H3K4me3, and K3K9ac), as well as DNase I hypersensitive sites, were uniformly underrepresented throughout the 64 kb region flanking fixed IAPs, but not significantly different from controls immediately next to the IntS for ETns (Fig 5A, Fig I in S1 Text, Fig 4A, and Fig H in S1 Text). Major individual predictors, as identified by single FLRs, were also remarkably similar between fixed IAPs (Table C in S1 Text and Fig 7B) and fixed ETns (Table C in S1 Text and Fig 7A)–with only one extra predictor for fixed IAPs; H3K9me3 content. The multiple FLR model had a 33.3% deviance explained (Table 6) and again showed many similarities to the analogous model for fixed ETns (Table 3).

Next, following a logic similar to the one used above for ETns, we attempted to separate integration vs. fixation preferences for IAPs. Just as was observed for ETns, genomic features significant in distinguishing the flanking sequences of polymorphic IAPs from control regions (Fig 5B and Fig J in S1 Text) were very similar to those distinguishing the flanking sequences of fixed IAP from control regions (Fig 5A and Fig I in S1 Text). The underrepresentation of Z-DNA repeats was more localized (close to the IntS), while recombination hotspot content was more uniformly overrepresented in polymorphic IAPs vs. controls than in fixed IAPs vs. controls. Moreover, transcription start sites showed no significant differences and the H3K27ac histone mark was only weakly underrepresented in polymorphic IAPs vs. controls, while these features were more prominent in fixed IAPs vs. controls suggesting their importance for fixation rather than integration. Single FLRs identified the same group of major predictors for polymorphic IAPs vs. controls as were identified for polymorphic ETns vs. controls, with the addition of the functional predictor ESC expression (explained deviance 22%)(Fig 7B and Table C in S1 Text). We excluded these features from the final multiple FLR model, which explained 30.4% of the deviance in discriminating polymorphic IAPs vs. controls (Table 7) and was similar to the analogous model for ETns (Table 4).

When comparing fixed and polymorphic IAPs (Fig 5C and Fig K in S1 Text), the ITP identified only three features that had lower means throughout the fixed IAP integration regions–SINEs, DNase I hypersensitive sites, and recombination hotspots. However, striking differences were observed for 12 genomic features with localized landscape. For instance, fixed IAPs revealed strong signatures of depressed means within ±4 kb from the IntS for features such as dinucleotide and trinucleotide microsatellites, most conserved elements, unmethylated CpGs, and the histone mark H3K27me3. Introns and two histone marks (i.e. H3K4me1 and H3K27ac) were also underrepresented in a larger area around the IntS. Moreover, we observed overrepresentation of LINEs and H3K9me3 (the mark of heterochromatic regions) surrounding the IntS in fixed vs. polymorphic IAPs. Exon content was underrepresented in a few windows downstream of the IntS, while AT count was overrepresented in most windows of both flanks except at the IntS. Single FLRs for fixed vs. polymorphic IAPs identified H3K9me3 as a very strong functional predictor (explained deviance of 21.5%, Fig 7B and Table C in S1 Text). All other features, singularly, could explain only a very low portion of the deviance (<2%), and even their concurrent effect was low, producing a multiple FLR model with an explained deviance of 3.8% (Table 8).

### HERV-Ks

Flanking sequences of fixed HERV-Ks were characterized by lower recombination rates and later replication timing than controls (Table B in S1 Text and Fig 2C). Based on the ITP (Fig 6A), microsatellites (of all four types), LINEs, recombination hotspots, L1 target sites, AT count, and H3K9me3 marks were overrepresented, while G-quadruplex repeats, SINEs, replication origins, cytosine methylation level features (i.e. CpG, CHG, and CHH), and four histone marks (H3K36me3, H3K4me1, H3K27ac, and H3K9ac) were underrepresented, throughout these flanking sequences. Exons, introns, and most conserved elements were underrepresented as well, especially near the IntS. Single FLR fits revealed that all four microsatellites types (scalar predictors), introns, and H1-hESC transcript expression, considered as functional predictors, each individually explained 26–76% of the deviance. These features were excluded from the final multiple FLR model (Fig 7C and Table C in S1 Text), which explained 79% of the deviance (Table 9). The model included LINEs and recombination hotspots as positive scalar predictors (RCDE of 13% and 9.7%, respectively). In terms of functional predictors, L1 target sites was the strongest predictor with a positive effect in the whole region from -30 to 30 kb, and stronger away from the IntS (RCDE 27.1%). In addition, AT count had a negative effect stronger away from the IntS (RCDE 12.6%), hypomethylation in testis and CpG islands had positive and negative effects, respectively, for the whole integration region (both RCDE of ~5%), and G-quadruplex repeats had a negative effect near and upstream of the IntS (RCDE 1.9%).

To highlight HERV-Ks’ integration preferences, we contrasted flanking sequences of *in vitro* HERV-Ks vs. control regions. The former replicated later and were more distant from the centromere than the latter (Table B in S1 Text and Fig 2C). The ITP indicated (Fig 6B) that G-quadruplex repeats, L1 target sites, recombination hotspots, replication origins, CHH methylation, and four histone marks associated with active transcription or promoters (H3K36me3, H3K4me1, H3K27ac, and H3K9ac) were underrepresented, while LINEs, AT count, and the H3K9me3 mark were overrepresented, throughout the flanking sequences compared to control regions. Additionally, the ITP indicated that there were fewer CHG methylated sites near the IntS of *in vitro* HERV-Ks than in control regions. Other genomic features had significant differences further away from the IntS (e.g., SINEs, TSS ENCODE, H1-hESC transcript expression, and H3K27me3), or showed more complex localized behaviors (Fig 6B and Fig M in S1 Text). The multiple FLR model for *in vitro* HERV-Ks vs controls explained 24.4% of the deviance (Table 10).

To focus on HERV-K fixation preferences, we contrasted flanking sequences of fixed vs. *in vitro* HERV-Ks. Recombination rates, replication timing, and distance to centromere were significantly different, with lower means for fixed compared to *in vitro* HERV-Ks (Table B in S1 Text and Fig 2C). The ITP (Fig 6C and Fig N in S1 Text) indicated that microsatellites, L1 target sites, and recombination hotspots were overrepresented throughout the flanking sequences of fixed vs. *in vitro* HERV-Ks. Introns and most conserved elements were underrepresented throughout the whole region too, but the difference was stronger near the IntS. Exons were underrepresented ±2 kb next to the IntS. Moreover, three histone marks (H3K4me3, H3K27ac, and H3K9ac) were underrepresented upstream of the IntS, while the H3K9me3 mark was overrepresented downstream of the IntS. CpG and CHG methylated sites, as well as H3K36me3, were generally underrepresented but had more complex localization patterns. Single FLRs identified six positive scalar predictors–all four microsatellites types, recombination hotspots, and L1 target sites–each of which, individually, explained as much as 85% of the deviance (Fig 7C and Table C in S1 Text).

## Discussion

Here, to investigate patterns in the distributions of ERV elements, we studied a number of genomic features in the flanking sequences of human and mouse ERVs with FDA, a class of statistical techniques still relatively underused in the field of genomics. In particular, we contrasted features between the flanking regions of fixed ERVs vs. controls, polymorphic (or *in vitro*) ERVs vs. controls, and fixed vs. polymorphic ERVs. The first type of contrast reflects both fixation and integration preferences with the latter somewhat eroded by selection, the second type highlights integration preferences with minimal influence of selection–particularly in the case of *in vitro* ERVs, while the third type captures fixation preferences. We observed that various genomic features are overrepresented or underrepresented in some but not other contrasts (Fig 7). Moreover, while some features present significant differences over the whole flanking regions considered, others present localized differences–especially close to the IntS. Below we discuss our findings, relating them to biological processes proxied by the various genomic features under analysis (Table 2).

### DNA conformation

We explored the associations between ERVs and a diverse set of non-B DNA conformation predictors inferred from the primary DNA sequence of the human and mouse genomes. *In vivo*, such conformations are formed transiently during recombination, repair, transcription, and replication, frequently causing genomic instability [79] and were found to be associated with the presence of DNA transposons [44,46]. We observed that mirror repeats and A-phased repeats are overrepresented in the flanking regions of fixed ETns, as well as of fixed and polymorphic IAPs, as compared with control regions (A-phased repeats are also overrepresented in the vicinity of fixed HERV-Ks). The overrepresentation of these repeats in the flanking regions of both fixed and polymorphic IAPs suggests their role in ERV integration–the lack of significant overrepresentation for polymorphic ETns perhaps being due to a more limited statistical power, given the smaller sample size (Table 1). A subset of mirror repeats–triplex repeats–are thought to bind mismatch and nucleotide excision repair proteins [79], therefore we propose that these protein complexes might be recognized by the integrase machinery and trigger ERV integration. This hypothesis needs to be evaluated experimentally. Mirror repeats have also been associated with low gene expression levels [79]. In agreement with this we found ERVs enriched in regions with low levels of transcription (see below). A-phased repeats cause double helix bends that have been implicated in nucleosome assembly and expansion of trinucleotide microsatellites [65,80] and might be important for the recognition of IntSs by the ERV integrase, as suggested by retroviral studies [81–84]. Moreover, unlike mirror repeats that do not have base composition bias, A-phased repeats are adenine-rich [65], resonating with the effect of A/T nucleotides (see below).

G-quadruplex and Z-DNA repeats displayed negative associations with the ERVs. G-quadruplex repeats are underrepresented in fixed vs. control and *in vitro* (or polymorphic) vs. control contrasts for both HERV-Ks and IAPs and thus likely inhibit ERV integration. Z-DNA repeats might inhibit ERV integration as well, because they are underrepresented in the flanking regions of both fixed and polymorphic IAPs. Importantly, these two types of repeats appear to be inhibitive of ERV integration beyond their GC-rich composition [65] because in several of our models they appear as significant predictors of ERV distributions together with AT-content (Tables 3 and 9). Both G-quadruplex and Z-DNA repeats are enriched in promoters and in the 5’ and 3’ gene termini [79], and therefore we cannot exclude the possibility that purifying selection removes ERVs from such regions.

We observed a strong overrepresentation of all four types of microsatellites in the fixed and polymorphic mouse ERVs compared to controls, suggesting the importance of microsatellites for ERV integration. Many microsatellites form non-canonical DNA structures–e.g., (AG)_n_ repeats form triplexes, (AT)_n_ form four-stranded cruciforms, while (CA)_n_ and (GC)_n_ form Z-DNA [85,86]–which lead to genome instability [87] and may be used by the integrases to recognize potential IntSs. In the human genome, we found an enrichment of microsatellites of all four types for fixed HERV-Ks compared with controls, and for fixed vs. *in vitro* HERV-Ks. Therefore, microsatellites might not be directly relevant to HERV-K integration, and instead be more relevant for their fixation (caution should be exercised though when comparing the results from *in vitro* vs. control and polymorphic vs. control contrasts, as the latter are more influenced by selection). The flanking regions of *in vitro* HERV-Ks actually had an underrepresentation of mononucleotide microsatellites, potentially because such microsatellites are frequently present as (A/T)_n_ repeats located at the 3’ ends of retrotransposed genes–where HERV-K integrations might be selected against. Additionally, (A/T)_n_ repeats are found in *Alu*s that are also underrepresented in the *in vitro* HERV-K flanking regions (see below).

### Nucleotide composition and the presence of other TEs

Corroborating previous studies [24,32], we observed that both mouse and human ERVs integrate and become fixed in AT-rich genomic regions. Indeed, AT-content was a significant predictor in eight out of nine ITP contrasts (except for *in vitro* HERV-K). Moreover, L1 target sites [88] are overrepresented in the flanking regions of polymorphic and fixed mouse ERVs as compared to controls, suggesting that these sequences correlate with mouse ERV integration. L1 target sites are also overrepresented in the flanking regions of fixed HERV-Ks vs. controls, and of fixed vs. *in vitro* HERV-Ks, but are *underrepresented* in the flanking sequences of *in vitro* HERV-Ks vs. controls, thus suggesting that these sequences correlate with HERV-K fixation. The enrichment of L1 target sites in the vicinity of ERVs can be explained by the high AT-content of these sequences, and thus it might simply reflect the AT-richness of the genomic regions in which ERVs integrate or are fixed, or perhaps it also indicates the enrichment of LINEs in these flanking regions (see below). Both AT nucleotides and L1 target sites have positive effects as single predictors, however, in the context of multiple FLR, when the two predictors are considered jointly, we observed a negative effect of AT nucleotides. Indeed, given the high correlation of the two predictors (Figs B and C in S1 Text), the positive effect shown by AT nucleotides when considered on their own is covered by a statistically dominant effect of L1 target sites in the joint analysis.

Mouse and human ERVs tend to be present in genomic regions rich in LINEs, but depleted of SINEs. This trend is significant for the fixed vs. control and polymorphic (or *in vitro*) vs. control contrasts, but not for the fixed vs. polymorphic (or *in vitro*) contrasts, arguing for a link with ERV integration. An association of ERVs with LINEs might reflect a preference towards integration in AT-rich sequences for both types of TEs. SINEs, in contrast, accumulate in GC-rich regions of the genome [12,89]. Additionally, a strong positive relationship between LINEs and ERVs, as evidenced by ITP and FLR, could also be explained by H3K9me3 histone marks known to be located in regions rich in these TEs [90] (see below). An important consideration in our analysis is that control regions are almost completely depleted of ERVs (see Methods), as we excluded even older ERVs than the ones we studied here from our control regions; this might underestimate the influence of such ERVs in integration preferences of the studied ERVs.

### Replication and recombination

In our study, ERV flanking regions had a late replicating profile. More specifically, the flanking regions of fixed ERVs replicated later than those of polymorphic ERVs, and the flanking regions of polymorphic ERVs replicated later than control regions. This was true for all three types of ERVs studied. Moreover, the flanking regions of both fixed and *in vitro* HERV-Ks presented a low content of replication origins–also a signature of late replication [91]. We hypothesize that the ERV integrase machinery targets late replicating regions because they are AT-rich [92] or that ERV integration might be coordinated with DNA replication, similar to Tf1 retrotransposon integration at stalled replication forks [93] and as proposed for DNA transposons [46].

Recombination appears to be important for both integration and fixation of ERVs. The flanking sequences of fixed ERVs have lower recombination rates than those of polymorphic (or *in vitro*) ERVs and of controls (Fig 2), suggesting a signature of fixation preference. This observation is in concordance with the hypothesis that ERVs are removed from highly recombining regions via ectopic recombination [6,31,94]. Alternatively, drift might fix ERVs in low recombining regions of the genome where selection is weaker. Katzourakis and colleagues [31] did not find a correlation between HERV fixation and recombination rates, but the discrepancy between our results and theirs might be due to the different HERV families investigated in the two studies.

We also observed that polymorphic and fixed mouse ERVs are located in genomic neighborhoods with higher content of recombination hotspots than controls, suggesting a role of recombination hotspots in mouse ERV integration. The overrepresentation of hotspots right next to the IntS for polymorphic ETns further supports this observation. In human, Myers and colleagues [68] detected overrepresentation of two retrovirus-like elements (THE1A and THE1B) in regions enriched with recombination hotspots. Moreover, for DNA transposons, it has been proposed that recombination hotspots are required by the transposition mechanism [46], and perhaps a similar interaction is essential for ERVs. In contrast, the comparison of fixed vs. polymorphic mouse ERVs indicated an underrepresentation of recombination hotspots suggesting that their high concentration *prevents* fixation. HERV-Ks presented an almost opposite pattern: recombination hotspots were overrepresented when contrasting fixed HERV-Ks vs. controls and fixed vs. *in vitro* HERV-Ks, and were underrepresented when contrasting *in vitro* HERV-Ks vs. controls, arguing for an association between recombination hotspots and HERV-K fixation but not integration. Note that we found experimentally validated recombination hotspots [67] to be significant in mouse, and predicted ones [66] to be significant in human, potentially explaining some differences in the results (experimentally validated recombination hotspots in human [68,69]) were not significant in our models).

### Location on the chromosome

We found that fixed mouse ERVs are located closer to centromeres than polymorphic mouse ERVs, and that the latter are located closer to centromeres than control regions. The preferential location of ERVs next to centromeres might be explained by their integration in AT-rich regions and by their fixation in regions with low recombination rates (see above). Indeed, recombination rates and GC-content, which are highly correlated with each other, are markedly decreased near centromeres [95]. In contrast, only small differences in the distance to centromere were observed among fixed HERV-Ks, *in vitro* HERV-Ks, and control regions.

### Epigenomic influences

Both methylated and unmethylated CpG sites were underrepresented in the flanking regions of fixed and polymorphic mouse ERVs compared to controls, reflecting integration of these elements in AT-rich areas of the genome. Interestingly, hypomethylated CpGs representing contiguous domains of low methylation measured in sperm (mean size of 1.8 kb) [75], are overrepresented in the flanking regions of fixed HERV-Ks compared to controls and to the flanking regions of *in vitro* HERV-Ks, suggesting a link with HERV-K fixation. Hypomethylated CpGs overlap with promoters and other regulatory regions [75] and thus selection might not tolerate ERVs in such areas of the genome.

All three ERV families studied tend to occur in regions with low DNase I hypersensitive sites content, i.e. in areas with closed chromatin. However, our results are conflicting as to whether this is an integration or fixation preference. Indeed, the signal for HERV-Ks is generally weak, in contrast to Brady and colleagues [34] who reported integration close to DNase I cleavage sites for *in vitro* HERV-K (i.e. HERV-K_con_). There is no significant signal near the IntS in the polymorphic ETns vs. controls contrast, and the signal in the fixed vs. polymorphic ETns comparison is also weak (Fig 4). For IAPs, the signal is stronger in the fixed and polymorphic vs. control contrasts than in the fixed vs. polymorphic contrast, arguing for a link with integration. Integration in areas with closed chromatin was previously observed for several retroviruses (e.g., MLV and HIV), and it was proposed that the nucleosomal DNA is targeted for integration by retroviral integrases [81,96].

### Histone marks

As reported previously for retroviruses [97], histone marks are important predictors of ERV distributions. Overall, our results corroborate previous findings (e.g., [98]) suggesting that ERVs integrate in areas of repressed chromatin. Consistent with previous studies [38,90,99–101], we observed an underrepresentation of histone marks associated with transcribed chromatin (H3K36me3 and H3K9ac), promoters (H3K4me3), and enhancers (H3K4me1 and H3K27ac; [102]) in the flanking regions of fixed and polymorphic ERVs. In agreement with this observation, we observed an overrepresentation of the H3K9me3 mark specific to repressed chromatin [102,103] in the flanking regions of both fixed and polymorphic elements. Surprisingly, and at odds with other studies (e.g., investigating youngest ETns and IAPs [90], and ERV-Ls [104]), we observed an underrepresentation of the H3K27me3 mark associated with repressed chromatin in the flanking regions of ERVs. What can explain the opposite results we obtained for H3K9me3 and H3K27me3, two marks of repressed chromatin which are one enriched and the other one depleted in the flanking regions of ERVs? While both marks signal repressed chromatin in early embryonic development, they are found in different regions of the genome [105]. On the one hand, the H3K9me3 mark is associated with heterochromatin formation due to the presence of tandem repeats [105], and thus its enrichment in the flanking regions of ERVs supports the strong association we found between ERVs and microsatellites. On the other hand, the H3K27me3 mark is abundant in gene-rich regions [105], and thus its depletion in the flanking regions of ERVs might reflect purifying selection acting against ERV integration in or around genes. Also, our results might be specific to the ESCs–as they agree with those of Hiratani and colleagues [106] who also studied ESCs. Some differences between our study and that of Brady and colleagues [34] might be explained by the use of histone modification data from different cells (Brady and colleagues utilized the data generated for CD4+ cells [99]). Finally, some of the differences among studies may be due to differences in the protocols used to construct control data sets.

Our results concerning ERVs fixation preferences (from the fixed vs. polymorphic, or *in vitro*, contrasts) with respect to histone marks suggest that ETns are fixed in areas rich in enhancer marks (H3K4me1 and H3K27ac) and this signal is localized at the IntS, while IAPs show an opposite trend (depletion of these marks) that is less localized. ETns bind transcription factors [107] and therefore could act as enhancers attracting H3K27ac, however we would like to see our observation of an association between ETns and enhancer marks validated in subsequent studies including histone marks from different ESC.

### Selection

In our analysis, exons, introns, and most conserved elements were underrepresented in the flanking regions of fixed and polymorphic (or *in vitro*) ERVs vs. controls, as well as in the flanking regions of fixed vs. polymorphic (or *in vitro*) ERVs. This may be evidence of purifying selection acting against integration and fixation of ERVs in areas of the genome rich in genes and most conserved elements. The signal was frequently weaker in the polymorphic (or *in vitro*) vs. control contrast, and localized close to the IntS in the fixed vs. polymorphic contrast. Our observation that mouse polymorphic ERVs are more prevalent in gene-rich regions than mouse fixed ERVs corroborated the findings of Zhang and colleagues [25]. Our results for fixed ERVs are also in agreement with the results of Medstrand and colleagues [32] who observed that older ERVs are underrepresented within 5 kb from genes. A selective purge from areas of the genome that are rich in genes and most conserved elements appears to be a characteristic of most TEs [24,32,36,45,46]. *Alu*s, preferentially integrating within genes [42,45], appear to be an exception. We also observed that ERVs are more prevalent in areas with low levels of transcription–likely reflecting both an integration preference for repressed chromatin and a fixation preference for gene-poor neighborhoods. This result contrasts observations made by Brady and colleagues [34]. These differences are probably due to the strict selection of control regions in our analyses and the different methods of analysis.

### The model for integration and fixation of ERVs

Some of the genomic features analyzed here were included in previous studies of ERV distributions, and our results are largely consistent with such studies [97,108–110]. However, our analysis included the most comprehensive list of genomic features to date, with many features not considered in previous studies (e.g., replication, most conserved elements, diverse DNA conformation features, L1 target sites, and recombination hotspots for human and mouse). Moreover, our use of FDA techniques allowed us to effectively investigate localization and scale at which the effects of these features unfold.

**Fig 8:**
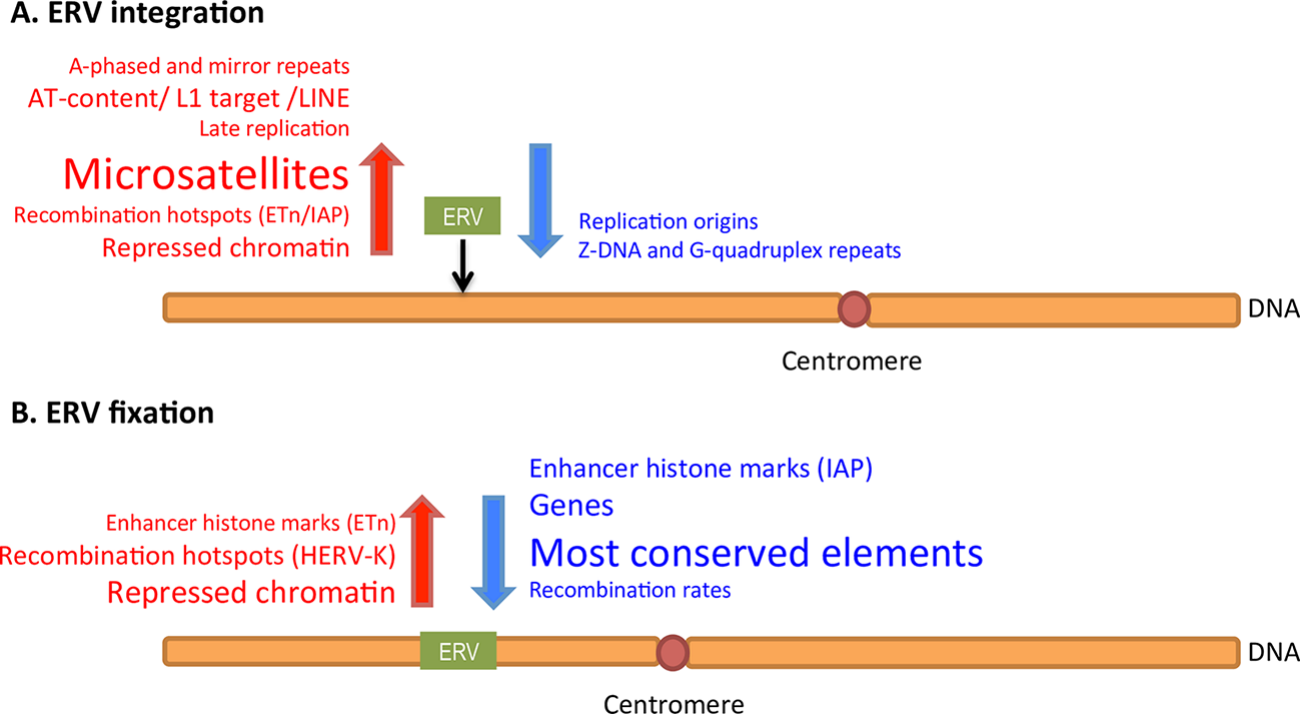
**Models of ERV (A) integration, and (B) fixation preferences based on our analysis of genomic features in the regions flanking the integration sites of mouse and human elements.** Word size is proportional to average deviance explained across various single FLR models. As indicated by the arrows, red and blue represent positive and negative effects, respectively.

As a result, we are in a position to propose a comprehensive model for the integration and fixation preferences of the mouse and human ERVs considered in our study (Fig 8). ERVs integrate in regions of the genome with high AT-content, enriched in A-phased repeats (as well as mirror repeats for mouse ERVs) and microsatellites–the former possessing and the latter frequently presenting non-canonical DNA structure. This highlights the potential importance of unusual DNA bendability in ERV integration, in agreement with previous studies [96,111]. Interestingly, some non-canonical DNA structures–e.g., G-quadruplexes and Z-DNA–appear to inhibit ERV integration (with Z-DNA avoidance strictly localized at the IntS site for polymorphic IAPs). ERV integration regions are rich in LINEs, replicate late, and have low density of replication origins. Replication hotspots might assist integration of mouse ERVs (with the signal localized close to the IntS for ETns). Our results on histone marks and DNase I hypersensitive sites suggest that integration occurs in gene-poor regions with closed chromatin–mimicking the behavior of retroviruses, whose integrases were proposed to target nucleosomal DNA [81,96]. Our observations also indicate that ETns and IAPs differ locally in their sensitivity to certain histone marks associated with promoters or expression and open chromatin, with the flanking regions of IAPs completely depleted of these features. However, for ETns these features are not significant next to the IntS.

The ERVs in our study are preferentially fixed in areas of the genome where both genes and most conserved elements are scarce (the signal is frequently stronger next to the IntS). There is also a strong preference of ERVs to be fixed in areas with low recombination rates, likely because in such areas they have a lower probability of being removed via ectopic recombination. The initial integration occurs preferentially relatively close to the centromere, likely because of increased AT-richness, and fixed ERVs are located even closer to the centromere, likely because of reduced recombination rates. ETns also show a fixation preference for areas rich in enhancer histone marks (alternatively they might induce the formation of these marks), and this signal is localized at the IntS.

### The importance of contrasts

Utilizing data on the distributions of fixed, polymorphic, and *in vitro* ERVs, and comparing their flanking regions with parts of the genome devoid of ERVs (controls) and with each other, we were able to separate, at least partially, the integration and fixation preferences of these elements. Ideally, one would like to utilize all three groups of flanking regions plus controls to study each element family. However, we were only in possession of data on polymorphic elements in the mouse genome, and of data on *in vitro* elements in the human genome. Because of this, observed patterns may not be entirely comparable; in particular, polymorphic mouse ERVs may experience more purifying selection than *in vitro* human ERVs. Moreover, one drawback of using *in vitro* integrations is that these are produced in cell lines using ERV constructs.

### Conclusions

The complexity of our study of ERV biology reflects the intricacy of the genome and of the mechanisms affecting the integration and fixation of these elements. We found evidence of the existence of genomic features associated with both insertion sites and the ability of elements to be fixed in the genome. Our analysis leveraged the availability of data on polymorphic ERVs and *in vitro* integrations, which we could contrast with fixed elements; we suggest applying a similar strategy to future studies of TE distribution. We expect further refinements of our model as more genomic data become available, e.g., nuclear lamina interactions and Hi-C profiles data [112] providing additional information on chromatin conformations that may influence DNA accessibility for new integrations. Finally, the FDA statistical methodology we utilized, in particular the ITP and FLR techniques, can be employed to analyze a variety of genomic data. These techniques are very versatile; the ITP does not require assumptions on the distributions underlying the data, and both the ITP and the FLR allow one to effectively characterize location and scale of a phenomenon, linking detected effects to specific intervals. We thus expect them to substantially contribute to numerous applications in genomics research.

## Materials and Methods

### Selection of ERVs and data manipulation

We retrieved ETn and IAP elements fixed (1,868 and 5,064, respectively) in mouse strain C57BL/6J, and those polymorphic (248 and 2,224, respectively) for C57BL/6J with respect to strains A/J, DBA/2J and 129X1/SvJ (reference genome mm9) [25] (Table 1). We assume ERVs shared among four mice strains to be fixed, even though these elements are transpositionally active and some of them might be absent in mouse strains not analyzed here. In addition, we chose 1,036 full-length and solo-LTR HERV-K elements from the human genome (hg19) [63] (Table 1). This data set includes all known polymorphic elements (a total of five at the time of writing this manuscript) [14] but, because they are also expected to be at least a few hundred years old [113], we analyzed them together and refer to them all as fixed HERV-Ks. All elements were manually re-annotated, to avoid errors from automatic annotations in the genomes. The data sets were filtered to include only the elements in the 60 bp to 11 kb length range (Table A in S1 Text). We also obtained the IntS of *in vitro* HERV-K integrations in human embryonic kidney (293T, female) and fibrosarcoma (HT1080, male) cell lines from [34] (Table 1). Out of the 1,565 integrations reported in this study, we located 1,208 by alignment of the IntS sequence to hg19 using BLAT [114]. Only matches with 100% identity over the total length of the IntS sequence were considered. In addition, IntS sequences that matched multiple locations (a total of 31 cases with integration site sequences <100 bp) were discarded because we could not identify their integration sites definitively.

For each element, we considered flanking sequences spanning 32-kb upstream and 32-kb downstream of the element, so that we could maximize the number of ERVs analyzed without overlapping of the flanking regions. This 64-kb region was then divided into 64 1-kb windows on which to quantify genomic features (Fig 1A). We eliminated elements for which the 32-kb flanking sequences overlapped by over 320-bp (1%) with genome gaps (i.e. sequences with Ns) or by over 1-bp with other elements’ flanking regions. Similarly, we generated 64-kb control regions, selecting areas that overlapped by at most 2% (1%) with mouse (human) LTR elements (as annotated in the corresponding genome at the UCSC Table browser [115]), or with the 32-kb flanking sequences of our ERVs (Table 1). After this filtering, we were left with 1,866 ETns (242 polymorphic and 1,624 fixed), 5,950 IAPs (1,986 polymorphic and 3,964 fixed), 826 fixed HERV-Ks and 1,065 *in vitro* HERV-Ks, which represent approximately four fifths of the elements analyzed in the original publications, respectively (Table 1). For ERVs that were annotated in the reverse strand (i.e. negative orientation with respect to the chromosome orientation), we inverted the 64-kb regions to consistently evaluate the effects of the genomic features with respect to the ERVs. Most of the functional datasets used in this study lack data for the Y chromosome, therefore we excluded the ERVs on this chromosome from our analyses.

### Selection of genomic features

We selected a set of genomic features associated with ERVs by prior studies, or found to be significant in other TE experiments. The data were obtained from the UCSC Table Browser [115], ENCODE [116], and previous publications (Table 2); when necessary the lift-over tool [117] was used to convert genome locations to mm9 and hg19. In total, we quantified 38 mouse and 39 human high-resolution genomic features, derived from to 39 mouse and 40 human datasets (Table 2) in 1-kb windows along the flanking sequences of our elements and our control regions (Fig 1A). Genome-wide microsatellite features were extracted from mouse and human genomes requiring a minimum of 9, 5, 4 and 4 motif repeats to define mono-, di-, tri-, and tetra-nucleotide microsatellites, respectively [118]. Mono-, di-, tri-, and tetranucleotide microsatellites were analyzed separately due to their unique genome distribution and mutation rates [118,119]. High-resolution recombination rates (in mouse), as well as methylation levels and expression levels, were quantified as weighted averages (average of rates or levels weighted by the total number of base pairs in a window) in each 1-kb window. All other features were quantified as coverages (fraction of the genomic window covered by the feature) in each 1-kb window, except L1 target sites, AT and CG nucleotides for which we used counts.

In order to reduce multicollinearity in downstream analyses, we applied a hierarchical clustering with Spearman’s rank correlation (distance = 1-|correlation|) and complete linkage [87], and selected one feature from each cluster above a 80% threshold in mouse and in human (Figs B and C in S1 Text). This reduced the set of features under investigation to 35 and 36 (35 and 37 datasets) in mouse and human, respectively (Figs D and E in S1 Text).

In addition to these high-resolution features, we selected two features that were available at low resolution in human and mouse (recombination rates and replication timing) and we considered their average value as a single value for each 64-kb region. Replication timing was measured as the log2 of early/late S-phase populations of cells in culture [120]; therefore regions that replicate earlier present high replication timing “values”. Moreover, distances to telomere and centromere (measured considering the element or the center of the control region) were included in the study.

### Statistical methodology overview

For each high-resolution genomic feature, we considered curves described by the whole 64-kb signal as the object of our study, embedding the problem in the framework of Functional Data Analysis (FDA) [48,49]. Importantly, FDA allowed us to naturally incorporate in the analysis the consecutive ordering of the measurements of each feature along the genome. The analysis was divided in two main phases (see Fig 1A). First, we considered each individual genomic feature separately, in order to assess whether it had significant discriminatory power in a given comparison (e.g. when contrasting ERV flanking sequences vs. controls; Fig 1B). We performed this phase using an extended version of the Interval Testing Procedure for functional data (ITP) recently introduced by [62]. ITP is able not only to assess whether differences exist between the distributions of the curves in, e.g., ERV flanking sequences vs. control regions, but also to anchor statistically significant differences to specific locations (1-kb windows) or sub-intervals in the regions, and to specific scales (e.g., a specific 5-kb sub-interval near the integration site, comprising a difference that is registered as significant up to a scale of 30-kb). Also, in any given comparison (Fig 1B), low-resolution features (recombination rates, replication timing, distance to telomere and distance to centromere) were tested using the simple univariate non parametric test employed by the first step of the ITP on each basis component (see below for more on the ITP). In the second phase we evaluated individual features fitting single Functional Logistic Regressions (FLR), and dealt with multiple predictors simultaneously by means of multiple FLR models (see Fig 1C) [48,49]. Combining the results obtained with these FDA methods we were able to perform an extensive, genome-wide evaluation of the effects of genomic landscape on integration and fixation of the various ERV families considered in this study (Fig 1C).

### Comparisons among data sets

In order to capture the influence of different genomic features on integration and/or fixation of ERVs, we applied the ITP and FLR to nine pair-wise comparisons (Fig 1B): (1) ETn polymorphic vs. control, (2) ETn fixed vs. controls, (3) ETn fixed vs. polymorphic, (4) IAP polymorphic vs. controls, (5) IAP fixed vs. controls, (6) IAP fixed vs. polymorphic, (7) *in vitro* HERV-Ks vs. controls, (8) fixed HERV-Ks vs. controls and (9) fixed vs. *in vitro* HERV-Ks. Only non-overlapping regions were employed in these comparisons, leading to the sample sizes indicated in Table 1.

### Interval Testing Procedure for functional data (ITP)

Let X_F,R_(w) w = 1,…,64 be the signal (measured as count, coverage or weighted average) related to the genomic feature F in the 64-kb region R–this will be one of two types of regions; e.g., the flanking sequences of an ERV element or a control region. We considered this signal as 64 equidistant pointwise measurements of the curve x_F,R_(t) within the interval [-31.5; 31.5] surrounding the IntS of an ERV, or the center of a control region (see Figs D and E in S1 Text). In order to determine which are the features that best distinguish between the two types of regions, we employed a functional hypothesis test on the differences between the curves distributions in the two groups. Many FDA methods exist to deal with this inferential problem globally, considering the distribution of the whole curve at the same time [121], or locally, considering each component of the curve separately. Since we were interested both in global and local significance, we used the ITP [62], which allowed us to simultaneously test for global differences between the two distributions and to impute observed differences to specific subregions. Because of its non-parametric (permutational) nature, this test does not require assumptions on the distributions underlying the data; this too made it highly attractive for our application, since many of the genomic features under consideration have signals that appear to follow complex, non-regular distributions. Importantly, we also introduced some extensions to the original ITP of [62]. In its extended version, the procedure allows us to (i) use multiple test statistics–capturing distinct aspects of the curves distributions–in assessing differences, and (ii) evaluate multiple scales by changing the maximum length of the intervals on which the global test is performed. Below we give a brief description of the ITP approach (full detail can be found in [62]).

Let x_1,i_(t) i = 1,…,n_1_ and x_2,i_(t) i = 1,…,n_2_ be two random samples from two independent stochastic curves. In our application, these are the curves x_F,R_(t) related to a feature F, in the two groups that we want to compare (e.g., flanking sequences of ERV vs. control regions). We consider the problem of testing the null hypothesis that the distributions of the two stochastic curves are equal, versus the alternative hypothesis that the two distributions differ. The first step of the ITP consists in decomposing the observed curves on a suitable reduced basis ${\left\{\phi_{k}\right\}}_{k=1}^{K}$ [48,49], i.e. represent each curve x_g,i_(t) as the set ${\{c_{g,i}^{\left(k\right)}\}}_{k=1}^{K}$ of coefficients obtained in the expansion

$$x_{g,i}\left(t\right)=\sum_{k=1}^{K}\;c_{g,i}^{\left(k\right)}\;\phi_{k}(t).$$

Then, in the second step, a univariate permutation test [122] on each basis component *c*^*(k)*^ is performed to assess whether its distribution is significantly different for the two stochastic curves (or underlying “population” of curves). Under the null hypothesis that the distributions are the same, all permutations of the n_1_+n_2_ “observed” coefficient values $c_{1,1}^{\left(k\right)},\cdots ,c_{1,n_{1}}^{\left(k\right)},c_{2,1}^{\left(k\right)},\cdots ,c_{2,n_{2}}^{\left(k\right)}$ have the same chance to occur. Hence, we can compute the empirical null distribution of a test statistics considering the values it assumes on all the different permutations; the p-value is generated dividing the number of permutations with a test statistics more extreme than the one observed on the data by the total number of permutations. The third step is to perform analogous multivariate permutation tests on each possible set of contiguous components (i.e. on the interval components *c*^*(1)*^*-c*^*(2)*^, *c*^*(2)*^*-c*^*(3)*^, …, *c*^*(1)*^-*c*^*(2)*^-*c*^*(3)*^,…). Here we employ the Nonparametric Combination Procedure developed by Pesarin and Salmaso [121], that allow us to implement a multivariate test by combining univariate test statistics obtained with synchronized permutations. In detail, the same permutations of the n_1_+n_2_ values $c_{1,1}^{\left(k\right)},\cdots ,c_{1,n_{1}}^{\left(k\right)},c_{2,1}^{\left(k\right)},\cdots ,c_{2,n_{2}}^{\left(k\right)}$ are applied to all components k = 1,…, K. Finally, in order to control the familywise error rate over sets of contiguous components, the fourth step computes an adjusted p-value for each component as the maximum among the p-values of all tests whose null hypothesis includes the component under consideration. Such a strategy exploits the ordered nature of the basis components creating a multiple testing correction that is coherent with the structure of data, and allows us to impute observed differences to specific subregions.

We chose to represent the curves using B-splines of order 1 (piecewise constant basis) with 65 or 33 nodes in the interval [-31.5; 31.5] and thus retain all the variability of the genomic features observed at 1-kb resolution (Figs D and E in S1 Text). In this way, we were able to test directly the raw data (65 nodes choice, one value every 1-kb) as well as a piecewise constant smoothed version of the raw data (33 nodes, one value every two 1-kb windows) without introducing substantial biases in the ITP. Notably, even if we are considering order 1 B-splines to represent the curves, the functional test statistics of the ITP (e.g. the mean curves) as computed on the data are rather smooth and their distributions do not show marked discontinuities (Figs F-N in S1 Text). The test p-values were computed considering 10,000 random permutations.

In its extended implementation, the ITP can be performed using different test statistics, each highlighting a particular aspect of the curves distributions. In particular, we considered three test statistics: 1) the sample mean difference, 2) the sample median difference, and 3) the sample variance ratio. In addition to introducing different test statistics, we modified the ITP so that differences between the distributions of the two stochastic curves are evaluated at all possible resolutions; that is, we corrected the p-values controlling the familywise error rate on intervals of all possible maximum length. In this way, we were able not only to evaluate the importance of a genomic feature in characterizing ERVs (globally or at specific locations) when considering the whole 64-kb flanking region, but also to establish if the feature of interest is able to differentiate between events and controls only at a smaller scale.

All genomic features found significant (p-value<0.05), considering at least one test statistics in the ITP, were included in the Functional Logistic Regression (FLR) analysis phase (see Fig 1C and below). In particular, features that showed significant differences between the two groups independently of scales and locations (invariant differential landscape features–IDL) were represented through their mean values across the 64 1-kb windows and treated as scalar predictors in the FLR. On the other hand, features that were significant only at a particular scale and/or for specific locations, considering at least one test statistics (localized differential landscape features–LDL) were treated as functional predictors. Genomic features that were significant on the whole 64-kb region but showed stronger differences (e.g., in means) in a particular location were also treated as functional predictors.

To test for significant differences (e.g., between ERVs flanking sequences and controls, or other comparisons) in recombination rate, replication timing, distance from telomere and distance from centromere (low-resolution features), we employed an univariate version of the ITP described above, where a single value is considered for each region. Similarly to the ITP, we tested the null hypothesis that the feature under study has the same distribution in elements and controls (or other comparisons), versus the alternative hypothesis that the two distributions differ, by means of a univariate permutation test. We considered again three test statistics: 1) the sample mean difference, 2) the sample median difference, and 3) the sample variance ratio. For each, we computed the test p-value estimating the distribution of the test statistics under the null hypothesis with 10,000 random permutations. The features that resulted significant (p-value<0.05) with respect to at least one test statistics were included in the FLR as scalar predictors (see Fig 1C and below).

### Functional Logistic Regression (FLR)

The second phase of our analysis consisted in fitting single and multiple FLR models [48,49] using as potential predictors the genomic features selected with the ITP (Fig 1C). The goal of single FLR fits was to identify predictors with very strong predictive power in each comparison. After setting aside these major predictors, whose strength may obscure the role of other features, the goal of multiple FLR was to relate the discrimination (e.g., between the flanking sequences of ERVs and control regions) with all remaining features simultaneously.

The details are as follows. For each comparison, we generated a binary response Y encoding events and controls as “1” and “0”, respectively, and considered the functional model

$$\text{logit}\left(E[Y|Z_{{\text{F}}_{1}},\cdots ,Z_{{\text{F}}_{r}},{\text{x}}_{{\text{F}}_{r+1}}\left(t\right),\cdots ,{\text{x}}_{{\text{F}}_{r+s}}\left(t\right)]\right)=\text{ln}\left(\frac{p}{1-p}\right)=\beta_{0}+\sum_{i=1}^{r}\beta_{{\text{F}}_{i}}\;{\text{Z}}_{{\text{F}}_{i}}+\sum_{i=r+1}^{r+s}\frac{1}{\sqrt{\left|{\text{I}}_{{\text{F}}_{i}}\right|}}\int_{{\text{I}}_{{\text{F}}_{i}}}\beta_{{\text{F}}_{i}}\left(t\right){\text{x}}_{{\text{F}}_{i}}\left(t\right)dt$$

where *p* represents the probability of an event conditionally to the observed features. Here ${\text{Z}}_{{\text{F}}_{i}}$ represent the scalar predictors, i.e. the features that emerged as important for all scales and locations (IDL features) and the low-resolution features selected through the univariate test, while ${\text{x}}_{{\text{F}}_{i}}\left(t\right)$ represent the functional predictors (LDL features) with support intervals ${\text{I}}_{{\text{F}}_{i}}$, respectively. In detail, the signal X_F,R_(w) w = 1,…,64 in region R for a genomic feature F that was significant independently of scales and locations in the ITP, was summarized by its sample mean over the 64 windows

$${\text{Z}}_{\text{F},\text{R}}=\frac{1}{64}\sum_{w=1}^{64}\;{\text{X}}_{\text{F},\text{R}}\left(w\right)$$

and then included in the FLR model as scalar predictor. Conversely, the curve x_F,R_(t) with pointwise evaluations X_F,R_(w) w = 1,…,64 corresponding to a genomic feature F that was significant exclusively in the interval I_F_ was included in the model as functional predictor in the selected interval. For each functional predictor, we expanded predictor curve and related coefficient function on the same reduced basis ${\left\{\phi_{F,k}\right\}}_{k=1}^{K_{F}}$, i.e.

$${\text{x}}_{\text{F},\text{R}}\left(\text{t}\right)=\sum_{k=1}^{K_{F}}c_{F,R}^{\left(k\right)}\;\phi_{F,k}\left(t\right)$$

$$\beta_{F}\left(t\right)=\sum_{k=1}^{K_{F}}b_{F}^{\left(k\right)}\;\phi_{F,k}\left(t\right).$$

This basis (with its specific support) was chosen separately for each functional predictor, in order to minimize the dimension *K*_*F*_ while capturing the scale that emerged as significant in the ITP.

To arrive to a meaningful FLR model (Fig 1C) we first considered each scalar predictor Z_F_ alone and fitted the single logistic regression model:

$$\text{logit}(E[Y|{\text{Z}}_{\text{F}}])=\text{ln}\;\left(\frac{p_{\text{F}}}{1-p_{\text{F}}}\right)=\beta_{0}+\beta_{\text{F}}{\text{Z}}_{\text{F}}$$

where *p*_*F*_ represents the probability of an event conditionally to the observed feature F. Whenever the distribution of the scalar predictor was skewed, we regularized it with a shifted logarithmic transformation and fit the model with the transformed data. More specifically, we took logs after adding a positive shift parameter *s* chosen to simultaneously maximize the p-values of the Shapiro-Wilk normality test on the transformed scalar signal in both events and controls. We computed the proportion of deviance explained (DE) by the model, or pseudo R-squared, as $\text{DE}=R_{pseudo}^{2}=\frac{D_{null}-D_{model}}{D_{null}}$ where *D*_*null*_ is the null deviance and *D*_*model*_ the model residual deviance [123]. This measure revealed the discriminatory strength of each individual scalar predictor (e.g., to distinguish between flanking sequences of ERVs and control regions). Similarly, we fitted a single functional logistic regression model for each individual functional predictor x_F_(t):

$$\text{logit}(E[Y|{\text{x}}_{\text{F}}\left(t\right)])=\text{ln}\left(\frac{p_{\text{F}}}{1-p_{\text{F}}}\right)=\beta_{0}+\frac{1}{\sqrt{\left|{\text{I}}_{\text{F}}\right|}}\int_{{\text{I}}_{\text{F}}}\beta_{\text{F}}\left(t\right){\text{x}}_{\text{F}}\left(t\right)dt$$

where *p*_*F*_ represents again the probability of an event conditionally to the observed feature F. Similar to scalar predictors, functional predictors were log-transformed as needed, and their strength was measured by means of the DE. Scalar or functional predictors that were very strong (DE > 20%) were noted and interpreted (see Results and Discussion), but *not* included in the multiple FLR–as their inclusion would obscure subtler contributions and co-significance of other, weaker predictors.

Next, we chose the best subset among the remaining scalar predictors using a multiple logistic regression model with LASSO regularization after predictors standardization [124]. The optimal regularization coefficient was chosen as the one maximizing the mean 10-fold cross validation misclassification rate. After identifying the best subset of scalar predictors, say ${\left\{Z_{F_{i}}\right\}}_{i=1}^{r}$, we fitted the standard additive multiple logistic model (without LASSO penalization)

$$\text{logit}\left(E[Y|{\text{Z}}_{{\text{F}}_{1}},\cdots ,{\text{Z}}_{{\text{F}}_{r}}]\right)=\text{ln}\;\left(\frac{p_{\text{scalar}}}{1-p_{\text{scalar}}}\right)=\beta_{0}+\sum_{i=1}^{r}\beta_{{\text{F}}_{i}}\;{\text{Z}}_{{\text{F}}_{i}}.$$

We then augmented this scalar model attempting to add the remaining functional predictors one at a time in a stepwise forward fashion (i.e. adding each time the functional predictor inducing the biggest gain in terms of DE), as long as the DE increased more than 1% and the AIC decreased. Finally, we excluded scalar predictors that may have been rendered non-significant by the introduction of the functional predictors in a stepwise backward fashion–leading to our final multiple FLR model for the comparison under consideration.

The importance of each predictor (scalar or functional) in the final multiple FLR model was measured by its relative contribution to the deviance explained (RCDE), defined as $\text{RCDE}=\frac{\left(D_{null}-D_{model})-(D_{null}-D_{red\;model}\right)}{\left(D_{null}-D_{model}\right)}$ where *D*_*null*_ is the null deviance, *D*_*model*_ the multiple FLR model residual deviance, and *D*_*red model*_ the residual deviance of the multiple FLR model obtained by removing the predictor of interest [87].

### Tools and packages

All data manipulations were performed with in-house scripts and Galaxy tools (e.g. lift-over, make windows, assign weighted-average, and feature coverage). Statistical analyses were performed in the R environment using the packages fda.usc [125], car [126], glmnet [127], pheatmap [128] and a modified version of the functions in fdatest [129].

## Supporting Information

S1 Text Supplemental tables and plots extending results for ITP and FLR. (PDF)

## References

[1] Blomberg J, Benachenhou F, Blikstad V, Sperber G, Mayer J. Classification and nomenclature of endogenous retroviral sequences (ERVs): Problems and recommendations. Gene. 2009;448: 115–123. 10.1016/j.gene.2009.06.007 19540319

[2] Jern P, Sperber GO, Blomberg J. Use of Endogenous Retroviral Sequences (ERVs) and structural markers for retroviral phylogenetic inference and taxonomy. Retrovirology. 2005;2: 50 16092962 10.1186/1742-4690-2-50 PMC1224870

[3] Maksakova IA, Romanish MT, Gagnier L, Dunn CA, de Lagemaat LNV, Mager DL. Retroviral elements and their hosts: Insertional mutagenesis in the mouse germ line. PLoS Genet. 2006;2: e2 16440055 10.1371/journal.pgen.0020002 PMC1331978

[4] Cowley M, Oakey RJ. Transposable elements re-wire and fine-tune the transcriptome. PLoS Genet. 2013;9: e1003234 10.1371/journal.pgen.1003234 23358118 PMC3554611

[5] Sverdlov ED. Perpetually mobile footprints of ancient infections in human genome. FEBS Lett. 1998;428: 1–6. 9645463 10.1016/s0014-5793(98)00478-5

[6] Belshaw R, Watson J, Katzourakis A, Howe A, Woolven-Allen J, Burt A, et al Rate of recombinational deletion among human endogenous retroviruses. J Virol. 2007;81: 9437–9442. 17581995 10.1128/JVI.02216-06 PMC1951428

[7] Elleder D, Kim O, Padhi A, Bankert JG, Simeonov I, Schuster SC, et al Polymorphic Integrations of an Endogenous Gammaretrovirus in the Mule Deer Genome. J Virol. 2012;86: 2787–2796. 10.1128/JVI.06859-11 22190723 PMC3302240

[8] Tarlinton RE, Meers J, Young PR. Retroviral invasion of the koala genome. Nature. 2006;442: 79–81. 16823453 10.1038/nature04841

[9] Roca AL, Pecon-Slattery J, O'Brien SJ. Genomically intact endogenous feline leukemia viruses of recent origin. J Virol. 2004 ed. 2004;78: 4370–4375. 15047851 10.1128/JVI.78.8.4370-4375.2004 PMC374283

[10] Chessa B, Pereira F, Arnaud F, Amorim A, Goyache F, Mainland I, et al Revealing the history of sheep domestication using retrovirus integrations. Science. 2009 ed. 2009;324: 532–536. 10.1126/science.1170587 19390051 PMC3145132

[11] Bannert N, Kurth R. The evolutionary dynamics of human endogenous retroviral families. Annu Rev Genomics Hum Genet. Annual Reviews; 2006;7: 149–173. 10.1146/annurev.genom.7.080505.115700 16722807

[12] Lander ES, Linton LM, Birren B, Nusbaum C, Zody MC, Baldwin J, et al Initial sequencing and analysis of the human genome. Nature. 2001;409: 860–921. 11237011 10.1038/35057062

[13] Belshaw R, Katzourakis A, Paces J, Burt A, Tristem M. High copy number in human endogenous retrovirus families is associated with copying mechanisms in addition to reinfection. Mol Biol Evol. 2005;22: 814–817. 15659556 10.1093/molbev/msi088

[14] Shin W, Lee J, Son S-Y, Ahn K, Kim H-S, Han K. Human-Specific HERV-K Insertion Causes Genomic Variations in the Human Genome. Plos One. 2013;8: e60605 10.1371/journal.pone.0060605 23593260 PMC3625200

[15] Dewannieux M, Harper F, Richaud A, Letzelter C, Ribet D, Pierron G, et al Identification of an infectious progenitor for the multiple-copy HERV-K human endogenous retroelements. Genome Research. 2006;16: 1548–1556. 17077319 10.1101/gr.5565706 PMC1665638

[16] Lee YN, Bieniasz PD. Reconstitution of an Infectious Human Endogenous Retrovirus. PLoS Pathog. 2007;3: e10 17257061 10.1371/journal.ppat.0030010 PMC1781480

[17] Taruscio D, Mantovani A. Factors regulating endogenous retroviral sequences in human and mouse. Cytogenet Genome Res. 2004;105: 351–362. 15237223 10.1159/000078208

[18] de Parseval N, Lazar V, Casella JF, Benit L, Heidmann T. Survey of Human Genes of Retroviral Origin: Identification and Transcriptome of the Genes with Coding Capacity for Complete Envelope Proteins. J Virol. 2003;77: 10414–10422. 12970426 10.1128/JVI.77.19.10414-10422.2003 PMC228468

[19] Grow EJ, Flynn RA, Chavez SL, Bayless NL, Wossidlo M, Wesche DJ, et al Intrinsic retroviral reactivation in human preimplantation embryos and pluripotent cells. Nature. 2015;522: 221–225. 10.1038/nature14308 25896322 PMC4503379

[20] Göke J, Lu X, Chan Y-S, Ng H-H, Ly L-H, Sachs F, et al Dynamic Transcription of Distinct Classes of Endogenous Retroviral Elements Marks Specific Populations of Early Human Embryonic Cells. Stem Cell. Elsevier Inc; 2015;16: 135–141. 10.1016/j.stem.2015.01.005 25658370

[21] Mouse Genome Sequencing Consortium, Waterston RH, Lindblad-Toh K, Birney E, Rogers J, Abril JF, et al Initial sequencing and comparative analysis of the mouse genome. Nature. 2002;420: 520–562. 12466850 10.1038/nature01262

[22] Peaston AE, Evsikov AV, Graber JH, de Vries WN, Holbrook AE, Solter D, et al Retrotransposons regulate host genes in mouse oocytes and preimplantation embryos. Dev Cell. 2004;7: 597–606. 15469847 10.1016/j.devcel.2004.09.004

[23] Ribet D, Dewannieux M, Heidmann T. An active murine transposon family pair: retrotransposition of “master” MusD copies and ETn trans-mobilization. Genome Research. 2004;14: 2261–2267. 15479948 10.1101/gr.2924904 PMC525684

[24] Nellåker C, Keane TM, Yalcin B, Wong K, Agam A, Belgard TG, et al The genomic landscape shaped by selection on transposable elements across 18 mouse strains. Genome Biology. BioMed Central Ltd; 2012;13: R45 10.1186/gb-2012-13-6-r45 22703977 PMC3446317

[25] Zhang Y, Maksakova IA, Gagnier L, van de Lagemaat LN, Mager DL. Genome-wide assessments reveal extremely high levels of polymorphism of two active families of mouse endogenous retroviral elements. PLoS Genet. 2008;4: e1000007 10.1371/journal.pgen.1000007 18454193 PMC2265474

[26] Magiorkinis G, Gifford RJ, Katzourakis A, De Ranter J, Belshaw R. Env-less endogenous retroviruses are genomic superspreaders. Proceedings of the National Academy of Sciences. 2012;109: 7385–7390. 10.1073/pnas.1200913109 PMC3358877 22529376

[27] van de Lagemaat LN, Landry J-R, Mager DL, Medstrand P. Transposable elements in mammals promote regulatory variation and diversification of genes with specialized functions. Trends Genet. 2003;19: 530–536. 14550626 10.1016/j.tig.2003.08.004

[28] Dupressoir A, Lavialle C, Heidmann T. From ancestral infectious retroviruses to bona fide cellular genes: Role of the captured syncytins in placentation. Placenta. Elsevier Ltd; 2012;33: 663–671. 10.1016/j.placenta.2012.05.005 22695103

[29] Blikstad V, Benachenhou F, Sperber GO, Blomberg J. Evolution of human endogenous retroviral sequences: a conceptual account. Cell Mol Life Sci. 2008;65: 3348–3365. 10.1007/s00018-008-8495-2 18818874 PMC11131805

[30] Moyes D, Griffiths DJ, Venables PJ. Insertional polymorphisms: a new lease of life for endogenous retroviruses in human disease. Trends Genet. 2007;23: 326–333. 17524519 10.1016/j.tig.2007.05.004

[31] Katzourakis A, Pereira V, Tristem M. Effects of Recombination Rate on Human Endogenous Retrovirus Fixation and Persistence. J Virol. 2007;81: 10712–10717. 17634225 10.1128/JVI.00410-07 PMC2045447

[32] Medstrand P, van de Lagemaat LN, Mager DL. Retroelement distributions in the human genome: Variations associated with age and proximity to genes. Genome Res. 2002;12: 1483–1495. 12368240 10.1101/gr.388902 PMC187529

[33] Wright SI, Agrawal N, Bureau TE. Effects of recombination rate and gene density on transposable element distributions in Arabidopsis thaliana. Genome Research. 2003;13: 1897–1903. 12902382 10.1101/gr.1281503 PMC403781

[34] Brady T, Lee YN, Ronen K, Malani N, Berry CC, Bieniasz PD, et al Integration target site selection by a resurrected human endogenous retrovirus. Genes Dev. 2009;23: 633–642. 10.1101/gad.1762309 19270161 PMC2658518

[35] Dewannieux M, Dupressoir A, Harper F, Pierron G, Heidmann T. Identification of autonomous IAP LTR retrotransposons mobile in mammalian cells. Nature Genet. 2004;36: 534–539. 15107856 10.1038/ng1353

[36] Smit AFA. Interspersed repeats and other mementos of transposable elements in mammalian genomes. Curr Opin Genet Dev. 1999;9: 657–663. 10607616 10.1016/s0959-437x(99)00031-3

[37] Zhang Y, Romanish MT, Mager DL. Distributions of Transposable Elements Reveal Hazardous Zones in Mammalian Introns. Plos Comput Biol. 2011;7: e1002046 10.1371/journal.pcbi.1002046 21573203 PMC3088655

[38] Xie M, Hong C, Zhang B, Lowdon RF, Xing X, Li D, et al DNA hypomethylation within specific transposable element families associates with tissue-specific enhancer landscape. Nature Genetics. Nature Publishing Group; 2013;45: 836–841. 10.1038/ng.2649 23708189 PMC3695047

[39] Rebollo R, Romanish MT, Mager DL. Transposable Elements: An Abundant and Natural Source of Regulatory Sequences for Host Genes. Annu Rev Genet. 2012;46: 21–42. 10.1146/annurev-genet-110711-155621 22905872

[40] Contreras-Galindo R, Kaplan MH, He S, Contreras-Galindo AC, Gonzalez-Hernandez MJ, Kappes F, et al HIV infection reveals widespread expansion of novel centromeric human endogenous retroviruses. Genome Research. 2013;23: 1505–1513. 10.1101/gr.144303.112 23657884 PMC3759726

[41] Berry C, Hannenhalli S, Leipzig J, Bushman FD. Selection of target sites for mobile DNA integration in the human genome. Plos Comput Biol. 2006;2: 1450–1462. 10.1371/journal.pcbi.0020157 PMC1664696 17166054

[42] Wagstaff BJ, Hedges DJ, Derbes RS, Campos Sanchez R, Chiaromonte F, Makova KD, et al Rescuing Alu: recovery of new inserts shows LINE-1 preserves Alu activity through A-tail expansion. PLoS Genet. 2012 ed. 2012;8: e1002842 10.1371/journal.pgen.1002842 22912586 PMC3415434

[43] Gao X, Hou Y, Ebina H, Levin HL, Voytas DF. Chromodomains direct integration of retrotransposons to heterochromatin. Genome Res. 2008;18: 359–369. 10.1101/gr.7146408 18256242 PMC2259100

[44] Vigdal TJ, Kaufman CD, Izsvak Z, Voytas DF, Ivics Z. Common physical properties of DNA affecting target site selection of Sleeping Beauty and other Tc1/mariner transposable elements. J Mol Biol. 2002;323: 441–452. 12381300 10.1016/s0022-2836(02)00991-9

[45] Kvikstad EM, Makova KD. The (r)evolution of SINE versus LINE distributions in primate genomes: sex chromosomes are important. Genome Res. 2010;20: 600–613. 10.1101/gr.099044.109 20219940 PMC2860162

[46] Campos Sanchez R, Kapusta A, Feschotte C, Chiaromonte F, Makova KD. Genomic Landscape of Human, Bat, and Ex Vivo DNA Transposon Integrations. Mol Biol Evol. 2014;31: 1816–1832. 10.1093/molbev/msu138 24809961 PMC4069622

[47] ENCODE Project Consortium, Bernstein BE, Birney E, Dunham I, Green ED, Gunter C, et al An integrated encyclopedia of DNA elements in the human genome. Nature. 2012;489: 57–74. 10.1038/nature11247 22955616 PMC3439153

[48] Ramsay JO, Silverman BW. Functional data analysis. Springer series in Statistics; 2005.

[49] Ramsay JO, Silverman BW. Applied Functional Data Analysis: Methods and Case Studies. Springer Series in Statistics; 2002.

[50] Sørensen H, Goldsmith J, Sangalli LM. An introduction with medical applications to functional data analysis. Aalen OO, Borgan Ø, Kvaløy JT, editors. Statist Med. 2013;32: 5222–5240. 10.1002/sim.5989 24114808

[51] Escabias M. Functional Data Analysis in Biometrics and Biostatistics. J Biom Biostat. 2012;03.

[52] Ullah S, Finch CF. Applications of functional data analysis: A systematic review. BMC Med Res Methodol. 2013;13: 43 10.1186/1471-2288-13-43 23510439 PMC3626842

[53] Ratcliffe SJ, Heller GZ, Leader LR. Functional data analysis with application to periodically stimulated foetal heart rate data. II: Functional logistic regression. Statist Med. 2002;21: 1115–1127. 10.1002/sim.1068 11933037

[54] Sangalli L, Secchi P, Vantini S, Veneziani A. A Case Study in Exploratory Functional Data Analysis: Geometrical Features of the Internal Carotid Artery. Journal of the American Statistical Association. 2009;104: 37–48.

[55] Ieva F, Paganoni AM. Risk prediction for myocardial infarction via generalized functional regression models. Statistical Methods in Medical Research. 2013;0: 1–13. 10.1177/0962280213495988 23868543

[56] Hébert-Losier K, Pini A, Vantini S, Strandberg J, Abramowicz K, Schelin L, et al One-leg hop kinematics 20 years following anterior cruciate ligament rupture: Data revisited using functional data analysis. Clinical Biomechanics. Elsevier Ltd; 2015;: 1–9. 10.1016/j.clinbiomech.2015.08.010 26365484

[57] Reimherr M, Nicolae D. A functional data analysis approach for genetic association studies. Ann Appl Stat. 2014;8: 406–429.

[58] Luo L, Boerwinkle E, Xiong M. Association studies for next-generation sequencing. Genome Research. 2011;21: 1099–1108. 10.1101/gr.115998.110 21521787 PMC3129252

[59] Vsevolozhskaya OA, Zaykin DV, Greenwood MC, Wei C, Lu Q. Functional Analysis of Variance for Association Studies. Wei Z, editor. Plos One. 2014;9: e105074 10.1371/journal.pone.0105074 25244256 PMC4171465

[60] Zhang F, Boerwinkle E, Xiong M. Epistasis analysis for quantitative traits by functional regression model. Genome Research. 2014;24: 989–998. 10.1101/gr.161760.113 24803592 PMC4032862

[61] Cremona MA, Sangalli LM, Vantini S, Dellino GI, Pelicci PG, Secchi P, et al Peak shape clustering reveals biological insights. BMC Bioinformatics. 2015;16: 349 10.1186/s12859-015-0787-6 26511446 PMC4625869

[62] Pini A, Vantini S. The Interval Testing Procedure: a General Framework for Inference in Functional Data Analysis. Biometrics. 2015. 10.1111/biom.12476 26811864

[63] Subramanian RP, Wildschutte JH, Russo C, Coffin JM. Identification, characterization, and comparative genomic distribution of the HERV-K (HML-2) group of human endogenous retroviruses. Retrovirology. BioMed Central Ltd; 2011;8: 90 10.1186/1742-4690-8-90 22067224 PMC3228705

[64] Katzourakis A, Rambaut A, Pybus OG. The evolutionary dynamics of endogenous retroviruses. Trends in Microbiology. 2005;13: 463–468. 16109487 10.1016/j.tim.2005.08.004

[65] Cer RZ, Bruce KH, Mudunuri US, Yi M, Volfovsky N, Luke BT, et al Non-B DB: a database of predicted non-B DNA-forming motifs in mammalian genomes. Nucleic Acids Res. 2011;39: D383–D391. 10.1093/nar/gkq1170 21097885 PMC3013731

[66] Myers S, Freeman C, Auton A, Donnelly P, McVean G. A common sequence motif associated with recombination hot spots and genome instability in humans. Nature Genet. 2008;40: 1124–1129. 10.1038/ng.213 19165926

[67] Brunschwig H, Levi L, Ben-David E, Williams RW, Yakir B, Shifman S. Fine-Scale Maps of Recombination Rates and Hotspots in the Mouse Genome. Genetics. 2012;191: 757–764. 10.1534/genetics.112.141036 22562932 PMC3389972

[68] Myers S, Bottolo L, Freeman C, McVean G, Donnelly P. A fine-scale map of recombination rates and hotspots across the human genome. Science. 2005;310: 321–324. 16224025 10.1126/science.1117196

[69] Kong A, Thorleifsson G, Gudbjartsson DF, Masson G, Sigurdsson A, Jonasdottir A, et al Fine-scale recombination rate differences between sexes, populations and individuals. Nature. Nature Publishing Group; 2010;467: 1099–1103. 10.1038/nature09525 20981099

[70] Kong A, Gudbjartsson DF, Sainz J, Jonsdottir GM, Gudjonsson SA, Richardsson B, et al A high-resolution recombination map of the human genome. Nature Genet. 2002;31: 241–247. 12053178 10.1038/ng917

[71] Cox A, Ackert-Bicknell CL, Dumont BL, Ding Y, Bell JT, Brockmann GA, et al A New Standard Genetic Map for the Laboratory Mouse. Genetics. 2009;182: 1335–1344. 10.1534/genetics.109.105486 19535546 PMC2728870

[72] Besnard E, Babled A, Lapasset L, Milhavet O, Parrinello H, Dantec C, et al Unraveling cell type–specific and reprogrammable human replication origin signatures associated with G-quadruplex consensus motifs. Nature Publishing Group. Nature Publishing Group; 2012;19: 837–844. 10.1038/nsmb.2339 22751019

[73] Lister R, Pelizzola M, Dowen RH, Hawkins RD, Hon G, Tonti-Filippini J, et al Human DNA methylomes at base resolution show widespread epigenomic differences. Nature. 2009;462: 315–322. 10.1038/nature08514 19829295 PMC2857523

[74] Zhao L, Sun MA, Li Z, Bai X, Yu M, Wang M, et al The dynamics of DNA methylation fidelity during mouse embryonic stem cell self-renewal and differentiation. Genome Research. 2014;24: 1296–1307. 10.1101/gr.163147.113 24835587 PMC4120083

[75] Molaro A, Hodges E, Fang F, Song Q, McCombie WR, Hannon GJ, et al Sperm Methylation Profiles Reveal Features of Epigenetic Inheritance and Evolution in Primates. Cell. Elsevier Inc; 2011;146: 1029–1041. 10.1016/j.cell.2011.08.016 21925323 PMC3205962

[76] Brawand D, Soumillon M, Necsulea A, Julien P, Csárdi G, Harrigan P, et al The evolution of gene expression levels in mammalian organs. Nature. 2011 ed. 2011;478: 343–348. 10.1038/nature10532 22012392

[77] Shen Y, Yue F, McCleary DF, Ye Z, Edsall L, Kuan S, et al A map of the cis-regulatory sequences in the mouse genome. Nature. 2012;488: 116–120. 10.1038/nature11243 22763441 PMC4041622

[78] Suzuki A, Wakaguri H, Yamashita R, Kawano S, Tsuchihara K, Sugano S, et al DBTSS as an integrative platform for transcriptome, epigenome and genome sequence variation data. Nucleic Acids Res. 2015;43: D87–D91. 10.1093/nar/gku1080 25378318 PMC4383915

[79] Zhao J, Bacolla A, Wang G, Vasquez K. Non-B DNA structure-induced genetic instability and evolution. Cell Mol Life Sci. 2010;67: 43–105. 10.1007/s00018-009-0131-2 19727556 PMC3017512

[80] Rohs R, West SM, Sosinsky A, Liu P, Mann RS, Honig B. The role of DNA shape in protein-DNA recognition. Nature. 2009;461: 1248–1253. 10.1038/nature08473 19865164 PMC2793086

[81] Pruss D, Bushman FD, Wolffe AP. Human immunodeficiency virus integrase directs integration to sites of severe DNA distortion within the nucleosome core. P Natl Acad Sci USA. 1994;91: 5913–5917. 10.1073/pnas.91.13.5913 PMC44107 8016088

[82] Katz RA, Gravuer K, Skalka AM. A preferred target DNA structure for retroviral integrase in vitro. J Biol Chem. 1998;273: 24190–24195. 9727042 10.1074/jbc.273.37.24190

[83] Milot E, Belmaaza A, Rassart E, Chartrand P. Association of a host DNA structure with retroviral integration sites in chromosomal DNA. Virology. 1994;201: 408–412. 8184553 10.1006/viro.1994.1310

[84] Müller HP, Varmus HE. DNA bending creates favored sites for retroviral integration: an explanation for preferred insertion sites in nucleosomes. Embo J. 1994;13: 4704–4714. 7925312 10.1002/j.1460-2075.1994.tb06794.x PMC395405

[85] Sinden RR. DNA Structure and Function. San Diego: Academic Press; 1994.

[86] Hile SE, Yan G, Eckert KA. Somatic mutation rates and specificities at TC/AG and GT/CA microsatellite sequences in nontumorigenic human lymphoblastoid cells. Cancer Res. 2000;60: 1698–1703. 10749142

[87] Fungtammasan A, Walsh E, Chiaromonte F, Eckert KA, Makova KD. A genome-wide analysis of common fragile sites: what features determine chromosomal instability in the human genome? Genome Res. 2012 ed. 2012;22: 993–1005. 10.1101/gr.134395.111 22456607 PMC3371707

[88] Cost GJ, Feng QH, Jacquier A, Boeke JD. Human L1 element target-primed reverse transcription in vitro. Embo J. 2002;21: 5899–5910. 12411507 10.1093/emboj/cdf592 PMC131089

[89] Jurka J, Krnjajic M, Kapitonov VV, Stenger JE, Kokhanyy O. Active Alu elements are passed primarily through paternal germlines. Theor Popul Biol. 2002;61: 519–530. 12167372 10.1006/tpbi.2002.1602

[90] Liu S, Brind’Amour J, Karimi MM, Shirane K, Bogutz A, Lefebvre L, et al Setdb1is required for germline development and silencing of H3K9me3-marked endogenous retroviruses in primordial germ cells. Genes Dev. 2014;28: 2041–2055. 10.1101/gad.244848.114 25228647 PMC4173156

[91] Méchali M, Yoshida K, Coulombe P, Pasero P. Genetic and epigenetic determinants of DNA replication origins, position and activation. Curr Opin Genet Dev. Elsevier Ltd; 2013;23: 124–131. 10.1016/j.gde.2013.02.010 23541525

[92] Woodfine K, Fiegler H, Beare DM, Collins JE, McCann OT, Young BD, et al Replication timing of the human genome. Hum Mol Genet. 2004;13: 191–202. 14645202 10.1093/hmg/ddh016

[93] Jacobs JZ, Rosado-Lugo JD, Cranz-Mileva S, Ciccaglione KM, Tournier V, Zaratiegui M. Arrested replication forks guide retrotransposon integration. Science. 2015;349: 1549–1553. 10.1126/science.aaa3810 26404838 PMC4832573

[94] Duret L, Marais G, Biémont C. Transposons but Not Retrotransposons Are Located Preferentially in Regions of High Recombination Rate in Caenorhabditis elegans. Genetics. 2000;: 1–9. 10.1093/genetics/156.4.1661 PMC1461346 11102365

[95] Jensen-Seaman MI, Furey TS, Payseur BA, Lu Y, Roskin KM, Chen C-F, et al Comparative recombination rates in the rat, mouse, and human genomes. Genome Research. 2004;14: 528–538. 15059993 10.1101/gr.1970304 PMC383296

[96] Pryciak PM, Varmus HE. Nucleosomes, DNA-binding proteins, and DNA sequence modulate retroviral integration target site selection. Cell. 1992;69: 769–780. 1317268 10.1016/0092-8674(92)90289-o

[97] Kvaratskhelia M, Sharma A, Larue RC, Serrao E, Engelman A. Molecular mechanisms of retroviral integration site selection. Nucleic Acids Res. 2014;42: 10209–10225. 10.1093/nar/gku769 25147212 PMC4176367

[98] Grewal SIS, Jia S. Heterochromatin revisited. Nat Rev Genet. 2007;8: 35–46. 17173056 10.1038/nrg2008

[99] Barski A, Cuddapah S, Cui K, Roh T-Y, Schones DE, Wang Z, et al High-resolution profiling of histone methylations in the human genome. Cell. 2007;129: 823–837. 17512414 10.1016/j.cell.2007.05.009

[100] Creyghton MP, Cheng AW, Welstead GG, Kooistra T, Carey BW, Steine EJ, et al Histone H3K27ac separates active from poised enhancers and predicts developmental state. Proceedings of the National Academy of Sciences. 2010;107: 21931–21936. 10.1073/pnas.1016071107 PMC3003124 21106759

[101] Hezroni H, Sailaja BS, Meshorer E. Pluripotency-related, Valproic Acid (VPA)-induced Genome-wide Histone H3 Lysine 9 (H3K9) Acetylation Patterns in Embryonic Stem Cells. Journal of Biological Chemistry. 2011;286: 35977–35988. 10.1074/jbc.M111.266254 21849501 PMC3195619

[102] Makova KD, Hardison RC. The effects of chromatin organization on variation in mutation rates inthe genome. Nat Rev Genet. Nature Publishing Group; 2015;16: 213–223. 10.1038/nrg3890 25732611 PMC4500049

[103] Rebollo R, Karimi MM, Bilenky M, Gagnier L, Miceli-Royer K, Zhang Y, et al Retrotransposon-Induced Heterochromatin Spreading in the Mouse Revealed by Insertional Polymorphisms. Greally JM, editor. PLoS Genet. 2011;7: e1002301 10.1371/journal.pgen.1002301 21980304 PMC3183085

[104] Day DS, Luquette LJ, Park PJ, Kharchenko PV. Estimating enrichment of repetitive elements from high-throughput sequence data. Genome Biology. 2010;11: R69 10.1186/gb-2010-11-6-r69 20584328 PMC2911117

[105] Kim J, Kim H. Recruitment and biological consequences of histone modification of H3K27me3 and H3K9me3. ILAR J. 2012;53: 232–239. 10.1093/ilar.53.3-4.232 23744963 PMC3747788

[106] Hiratani I, Ryba T, Itoh M, Yokochi T, Schwaiger M, Chang C-W, et al Global Reorganization of Replication Domains During Embryonic Stem Cell Differentiation. Gasser SM, editor. Plos Biology. 2008;6: e245 10.1371/journal.pbio.0060245 PMC2561079 18842067

[107] Maksakova IA, Mager DL. Transcriptional Regulation of Early Transposon Elements, an Active Family of Mouse Long Terminal Repeat Retrotransposons. J Virol. 2005;79: 13865–13874. 16254322 10.1128/JVI.79.22.13865-13874.2005 PMC1280189

[108] Maskell DP, Renault L, Serrao E, Lesbats P, Matadeen R, Hare S, et al Structural basis for retroviral integration into nucleosomes. Nature. 2015;523: 366–369. 10.1038/nature14495 26061770 PMC4530500

[109] Bor YC, Bushman FD, Orgel LE. In vitro integration of human immunodeficiency virus type 1 cDNA into targets containing protein-induced bends. P Natl Acad Sci USA. 1995;92: 10334–10338. 10.1073/pnas.92.22.10334 PMC40791 7479779

[110] de Jong J, Akhtar W, Badhai J, Rust AG, Rad R, Hilkens J, et al Chromatin Landscapes of Retroviral and Transposon Integration Profiles. Roth MJ, editor. PLoS Genet. 2014;10: e1004250 10.1371/journal.pgen.1004250 24721906 PMC3983033

[111] Serrao E, Ballandras-Colas A, Cherepanov P, Maertens GN, Engelman AN. Key determinants of target DNA recognition by retroviral intasomes. Retrovirology. 2015;12: 1295. 10.1186/s12977-015-0167-3 PMC4422553 25924943

[112] Jin F, Li Y, Dixon JR, Selvaraj S, Ye Z, Lee AY, et al A high-resolution map of the three-dimensional chromatin interactome in human cells. Nature. Nature Publishing Group; 2013;503: 290 10.1038/nature12644 24141950 PMC3838900

[113] Belshaw R, Dawson ALA, Woolven-Allen J, Redding J, Burt A, Tristem M. Genomewide screening reveals high levels of insertional polymorphism in the human endogenous retrovirus family HERV-K(HML2): Implications for present-day activity. J Virol. 2005;79: 12507–12514. 16160178 10.1128/JVI.79.19.12507-12514.2005 PMC1211540

[114] Kent WJ. BLAT—The BLAST-Like Alignment Tool. Genome Research. 2002;12: 656–664. 11932250 10.1101/gr.229202 PMC187518

[115] Karolchik D, Baertsch R, Diekhans M, Furey TS, Hinrichs A, Lu YT, et al The UCSC Genome Browser Database. Nucleic Acids Res. 2003rd ed. 2003;31: 51–54. 12519945 10.1093/nar/gkg129 PMC165576

[116] Rosenbloom KR, Sloan CA, Malladi VS, Dreszer TR, Learned K, Kirkup VM, et al ENCODE data in the UCSC Genome Browser: year 5 update. Nucleic Acids Res. 2013;41: D56–63. 10.1093/nar/gks1172 23193274 PMC3531152

[117] Blankenberg D, Kuster Von G, Coraor N, Ananda G, Lazarus R, Mangan M, et al Galaxy: a web-based genome analysis tool for experimentalists. Curr Protoc Mol Biol. 2010 ed. 2010;Chapter 19: Unit 19 10 1–21. 10.1002/0471142727.mb1910s89 PMC4264107 20069535

[118] Ananda G, Walsh E, Jacob KD, Krasilnikova M, Eckert KA, Chiaromonte F, et al Distinct Mutational Behaviors Differentiate Short Tandem Repeats from Microsatellites in the Human Genome. Genome Biol Evol. 2013;5: 606–620. 10.1093/gbe/evs116 23241442 PMC3622297

[119] Ellegren H. Microsatellites: simple sequences with complex evolution. Nat Rev Genet. 2004;5: 435–445. 15153996 10.1038/nrg1348

[120] Ryba T, Hiratani I, Lu J, Itoh M, Kulik M, Zhang J, et al Evolutionarily conserved replication timing profiles predict long-range chromatin interactions and distinguish closely related cell types. Genome Res. 2010;20: 761–770. 10.1101/gr.099655.109 20430782 PMC2877573

[121] Secchi P, Stamm A, Vantini S. Inference for the mean of large p small n data: A finite-sample high-dimensional generalization of Hotelling’s theorem. Electron J Statist. 2013;7: 2005–2031.

[122] Pesarin F, Salmaso L. Permutation tests for complex data: theory, applications and software John Wiley & Sons; 2010.

[123] Efron B. Regression and ANOVA with zero-one data: Measures of residual variation. Journal of the American Statistical Association. 1978.

[124] Tibshirani R. Regression Shrinkage and Selection via the Lasso. Journal of the Royal Statistical Society. 1996;58: 267–288.

[125] Febrero-Bande M, Oviedo de la Fuente M. Statistical computing in functional data analysis: the R package fda.usc. Journal of Statistical Software. 2012;51: 1–28.23504300

[126] Fox J, Weisberg S. An {R} companion to applied regression [Internet]. Second. Thousand Oaks, CA: Sage; 2011 Available: http://socserv.socsci.mcmaster.ca/jfox/Books/Companion

[127] Friedman J, Hastie T, Simon N. glmnet: Lasso and Elastic-Net Regularized Generalized Linear Models [Internet]. 2nd ed. Available: http://www.jstatsoft.org/v33/i01/.

[128] Kolde R. pheatmap: Pretty Heatmaps. R software environment. 1st ed. 2015.

[129] Pini A, Vantini S. Package “fdatest.” R software environment. 2nd ed. 2015.

