# Supplementary material for: Integration and Fixation Preferences of Human and Mouse Endogenous Retroviruses Uncovered with Functional Data Analysis
Source: PLoS Comput Biol. 2016 Jun 16;12(6):e1004956. doi: 10.1371/journal.pcbi.1004956 (PMC4911145; doi:10.1371/journal.pcbi.1004956)
Supplement: S1 Text — (PDF) [file pcbi.1004956.s001.pdf]

Table A. Statistics of the sizes of the ERVs studied, including those in the sex chromosomes – X and Y

ERV	Number	Mean (bp)	Standard deviation (bp)	0%	25%	50%	75%	100%
ETn	1866	1519	2429	60	316	331	340	9776
IAP	5950	3009	2725	60	402	2722	5281	9877
HERV-K	872	1430	1704	751	965	974	1011	10368

Table B. Univariate permutation tests for low-resolution features. P-values obtained from univariate permutation tests with three different test statistics (sample mean difference, sample median difference, and sample variance ratio) and for three comparisons (fixed elements vs. controls, polymorphic (or *in vitro*) elements vs. controls, and fixed vs polymorphic (or *in vitro*) elements) in the study of ETns, IAPs, and HERV-Ks. Significant results (p-value<0.05) are in bold, and color coded as red when the statistics is higher in, e.g., fixed elements vs. controls, and in blue when it is lower.

ETn	Fixed vs. control			Polymorphic vs. control			Fixed vs. polymorphic		
	Mean	Median	Variance	Mean	Median	Variance	Mean	Median	Variance
**Recombination rates**	**0.00**	**0.00**	0.96	0.32	0.22	0.13	**0.00**	**0.00**	0.11
**Replication timing**	**0.00**	**0.00**	**0.00**	**0.00**	**0.00**	**0.01**	0.12	0.06	0.39
**Distance to telomere**	0.06	0.06	0.09	0.96	0.88	0.13	0.28	0.50	0.63
**Distance to centromere**	**0.00**	**0.00**	0.77	0.67	0.36	0.19	**0.00**	**0.00**	0.15

IAP	Fixed vs. control			Polymorphic vs. control			Fixed vs. polymorphic		
	Mean	Median	Variance	Mean	Median	Variance	Mean	Median	Variance
**Recombination rates**	**0.00**	**0.00**	0.58	0.11	0.35	0.96	**0.00**	**0.00**	0.58
**Replication timing**	**0.00**	**0.00**	**0.00**	**0.00**	**0.00**	**0.02**	**0.00**	**0.00**	**0.00**
**Distance to telomere**	0.71	0.98	**0.00**	0.57	0.27	**0.03**	0.78	0.16	0.23
**Distance to centromere**	**0.00**	**0.00**	0.53	0.57	0.42	0.51	**0.00**	**0.00**	0.12

HERV-K	Fixed vs. control			*In vitro* vs. control			Fixed vs. *in vitro*		
	Mean	Median	Variance	Mean	Median	Variance	Mean	Median	Variance
**Recombination rates**	**0.01**	**0.00**	0.26	0.22	0.98	0.32	**0.00**	**0.00**	0.06
**Replication timing**	**0.00**	**0.00**	**0.00**	**0.00**	**0.00**	**0.00**	**0.00**	**0.00**	0.08
**Distance to telomere**	0.53	**0.00**	**0.00**	0.84	0.17	**0.01**	0.48	**0.03**	0.08
**Distance to centromere**	0.89	0.60	**0.00**	**0.01**	**0.03**	0.12	**0.05**	**0.02**	**0.02**

Table C. Predictors that explained a deviance >20% in single logistic regression fits for the three comparisons implemented for each of type of elements – ETns, IAPs and HERV-Ks (these predictors were excluded from multiple FLR models).

The "Predictor" column reports predictors included in each single logistic regression fit. The "Coefficient" column reports coefficient estimates (a positive coefficient means that an increase in the feature increases the likelihood of, e.g., fixed vs. control; a negative coefficient means an increase in the feature decreases such likelihood). The "p-value" column reports p-values for the coefficients. They both are in bold if p-value<0.05. For functional predictors, several rows are listed corresponding to the intervals where the feature was considered – as indicated in the "Range of windows" column. The "DE" column reports the total deviance explained by each single logistic regression fit.

A. Fixed ETn vs control

Predictor	Range of windows	Coefficient	p-value	DE (%)
Mononucleotide content	Scalar	**4.5E+00**	**2.0E-16**	26.9
Dinucleotide content	Scalar	**1.0E+01**	**2.0E-16**	88.6
Trinucleotide content	Scalar	**4.8E+00**	**2.0E-16**	32.0
Tetranucleotide content	Scalar	**8.8E+00**	**2.0E-16**	75.1
LINE content	(-32,-24)	**1.6E-02**	**1.7E-03**	33.0
	(-24,-16)	**1.1E-02**	**3.9E-02**	
	(-16,-8)	**2.2E-02**	**4.5E-05**	
	(-8,0)	**2.9E-02**	**6.3E-08**	
	(0,8)	**2.6E-02**	**6.5E-07**	
	(8,16)	**1.7E-02**	**1.3E-03**	
	(16,24)	**1.1E-02**	**3.6E-02**	
	(24,32)	**2.2E-02**	**1.3E-05**	
Intron content	(-32,-16)	-1.3E-02	2.4E-01	26.4
	(-16,0)	**-8.2E-02**	**2.7E-09**	
	(0,16)	**-6.9E-02**	**1.2E-06**	
	(16,32)	**-4.5E-02**	**2.7E-05**	
Most conserved elements content	(-30,-20)	**-2.3E-02**	**1.3E-04**	46.6
	(-20,-10)	**-2.0E-02**	**1.2E-03**	
	(-10,0)	**-6.2E-02**	**2.0E-16**	
	(0,10)	**-5.6E-02**	**2.0E-16**	
	(10,20)	**-3.5E-02**	**3.0E-08**	
	(20,30)	**-2.9E-02**	**1.4E-06**	
mESC expression WA	(-30,-10)	-1.0E-03	1.9E-01	26.8
	(-10,10)	**-9.5E-03**	**2.0E-16**	
	(10,30)	**-2.7E-03**	**2.1E-04**	

B. Polymorphic ETn vs control

Predictor	Range of windows	Coefficient	p-value	DE (%)
Mononucleotide content	Scalar	**4.5E+00**	**2.0E-16**	24.2
Dinucleotide content	Scalar	**9.3E+00**	**2.0E-16**	85.9
Trinucleotide content	Scalar	**5.3E+00**	**2.0E-16**	33.9
Tetranucleotide content	Scalar	**9.3E+00**	**2.0E-16**	77.2
LINE content	Scalar	**8.3E-01**	**2.0E-16**	26.7
Intron content	(-28,-20)	-4.6E-02	2.6E-01	21.1
	(-20,-12)	-3.0E-02	5.8E-01	
	(-12,-4)	-5.5E-02	3.1E-01	
	(-4,4)	**-1.9E-01**	**2.7E-04**	
	(4,12)	-1.1E-02	8.4E-01	
	(12,20)	-7.0E-02	2.0E-01	
	(20,28)	7.1E-03	8.7E-01	
Most conserved elements content	Scalar	**-5.8E+00**	**2.0E-16**	28.5

C. Fixed ETn vs polymorphic

Predictor	Range of windows	Coefficient	p-value	DE (%)
----------------------------	------	------	------	------

D. Fixed IAP vs control

Predictor	Range of windows	Coefficient	p-value	DE (%)
Mononucleotide content	Scalar	**4.9E+00**	**2.0E-16**	27.7
Dinucleotide content	Scalar	**3.5E+01**	**2.0E-16**	86.1
Trinucleotide content	Scalar	**4.7E+00**	**2.0E-16**	30.7
Tetranucleotide content	Scalar	**7.9E+00**	**2.0E-16**	70.5
LINE content	Scalar	**1.6E+00**	**2.0E-16**	42.3
Intron content	(-30,-20)	4.6E-03	7.8E-01	22.8
	(-20,-10)	**-5.3E-02**	**1.5E-02**	
	(-10,0)	**-9.5E-02**	**4.3E-05**	
	(0,10)	**-9.5E-02**	**5.4E-05**	
	(10,20)	-1.2E-02	5.9E-01	
	(20,30)	**-4.7E-02**	**5.0E-03**	
Most conserved elements content	(-28,-20)	**-3.0E-02**	**1.0E-08**	43.8
	(-20,-12)	**-2.9E-02**	**3.4E-07**	
	(-12,-4)	**-3.8E-02**	**2.5E-11**	
	(-4,4)	**-8.3E-02**	**2.0E-16**	
	(4,12)	**-3.3E-02**	**6.0E-09**	
	(12,20)	**-3.2E-02**	**2.8E-08**	
	(20,28)	**-2.5E-02**	**3.7E-06**	
mESC expression WA	(-30,-10)	**-1.3E-03**	**4.6E-02**	26.1
	(-10,10)	**-9.4E-03**	**2.0E-16**	
	(10,30)	**-2.4E-03**	**1.2E-04**	
H3K9me3 content	(-28,-20)	**4.4E-02**	**5.9E-03**	31.9
	(-20,-12)	**1.3E-01**	**9.6E-07**	
	(-12,-4)	**4.3E-02**	**9.8E-03**	
	(-4,4)	**3.1E-01**	**2.0E-16**	
	(4,12)	**6.5E-02**	**2.0E-04**	
	(12,20)	2.2E-02	1.6E-01	
	(20,28)	**8.4E-02**	**4.2E-05**	

E. Polymorphic IAP vs control

Predictor	Range of windows	Coefficient	p-value	DE (%)
Mononucleotide content	Scalar	**4.6E+00**	**2.0E-16**	27.4
Dinucleotide content	Scalar	**3.8E+01**	**2.0E-16**	89.1
Trinucleotide content	Scalar	**5.1E+00**	**2.0E-16**	36.0
Tetranucleotide content	Scalar	**8.5E+00**	**2.0E-16**	74.5
LINE content	Scalar	**1.1E+00**	**2.0E-16**	40.2
Intron content	(-28,-20)	-2.3E-03	9.2E-01	21.8
	(-20,-12)	**-6.8E-02**	**2.0E-02**	
	(-12,-4)	-5.5E-02	6.1E-02	
	(-4,4)	**-1.2E-01**	**7.3E-05**	
	(4,12)	-3.9E-02	2.1E-01	
	(12,20)	-1.2E-02	7.0E-01	
	(20,28)	**-7.0E-02**	**2.5E-03**	
Most conserved elements content	Scalar	**-2.5E+00**	**2.0E-16**	35.1
mESC expression WA	(-30,-10)	**-2.4E-03**	**4.6E-03**	22.3
	(-10,10)	**-9.4E-03**	**2.0E-16**	
	(10,30)	**-2.7E-03**	**8.7E-04**	

F. Fixed IAP vs polymorphic

Predictor	Range of windows	Coefficient	p-value	DE (%)
H3K9me3 content	(-28,-20)	-8.7E-03	2.8E-01	21.5
	(-20,-12)	**-2.1E-02**	**2.4E-02**	
	(-12,-4)	**-2.8E-02**	**3.0E-03**	
	(-4,4)	**1.7E-01**	**2.0E-16**	
	(4,12)	-1.5E-02	7.8E-02	
	(12,20)	**-1.9E-02**	**2.5E-02**	
	(20,28)	6.0E-04	9.5E-01	

G. Fixed HERV-K vs control

Predictor	Range of windows	Coefficient	p-value	DE (%)
Mononucleotide content	Scalar	**9.4E+00**	**2.0E-16**	76.4
Dinucleotide content	Scalar	**6.8E+00**	**2.0E-16**	54.9
Trinucleotide content	Scalar	**5.5E+00**	**2.0E-16**	29.1
Tetranucleotide content	Scalar	**2.5E+00**	**2.0E-16**	60.4
Intron content	(-28,-20)	**-7.7E-02**	**1.6E-03**	25.7
	(-20,-12)	-5.9E-02	5.4E-02	
	(-12,-4)	-2.8E-04	9.9E-01	
	(-4,4)	**-1.8E-01**	**9.3E-10**	
	(4,12)	-2.4E-02	4.2E-01	
	(12,20)	-5.4E-02	6.3E-02	
	(20,28)	**-6.8E-02**	**3.0E-03**	
H1-hESC transcript expression WA	(-32,-16)	**-5.2E-03**	**1.5E-04**	26.6
	(-16,0)	**-7.5E-03**	**4.0E-06**	
	(0,16)	**-9.5E-03**	**4.4E-09**	
	(16,32)	**-4.5E-03**	**5.4E-04**	

H. *In vitro* HERV-K vs control

Predictor	Range of windows	Coefficient	p-value	DE (%)
----------------------------	------	------	------	------

I. Fixed vs *in vitro* HERV-K

Predictor	Range of windows	Coefficient	p-value	DE (%)
Mononucleotide content	Scalar	**1.1E+01**	**2.0E-16**	85.5
Dinucleotide content	Scalar	**7.3E+00**	**2.0E-16**	59.3
Trinucleotide content	Scalar	**6.3E+00**	**2.0E-16**	36.9
Tetranucleotide content	Scalar	**1.0E+01**	**2.0E-16**	64.6
L1 target sites count	Scalar	**4.2E+00**	**2.0E-16**	23.6
Recombination hotspots motif count	Scalar	**1.6E+00**	**2.0E-16**	26.2

Figure A. Scatterplot of chromosome size versus number of ERVs integrated. Correlation is quite high for all the elements, both in human and mouse. Notably, human chromosome 19 stands out as an outlier, showing a higher concentration of fixed HERV-K.

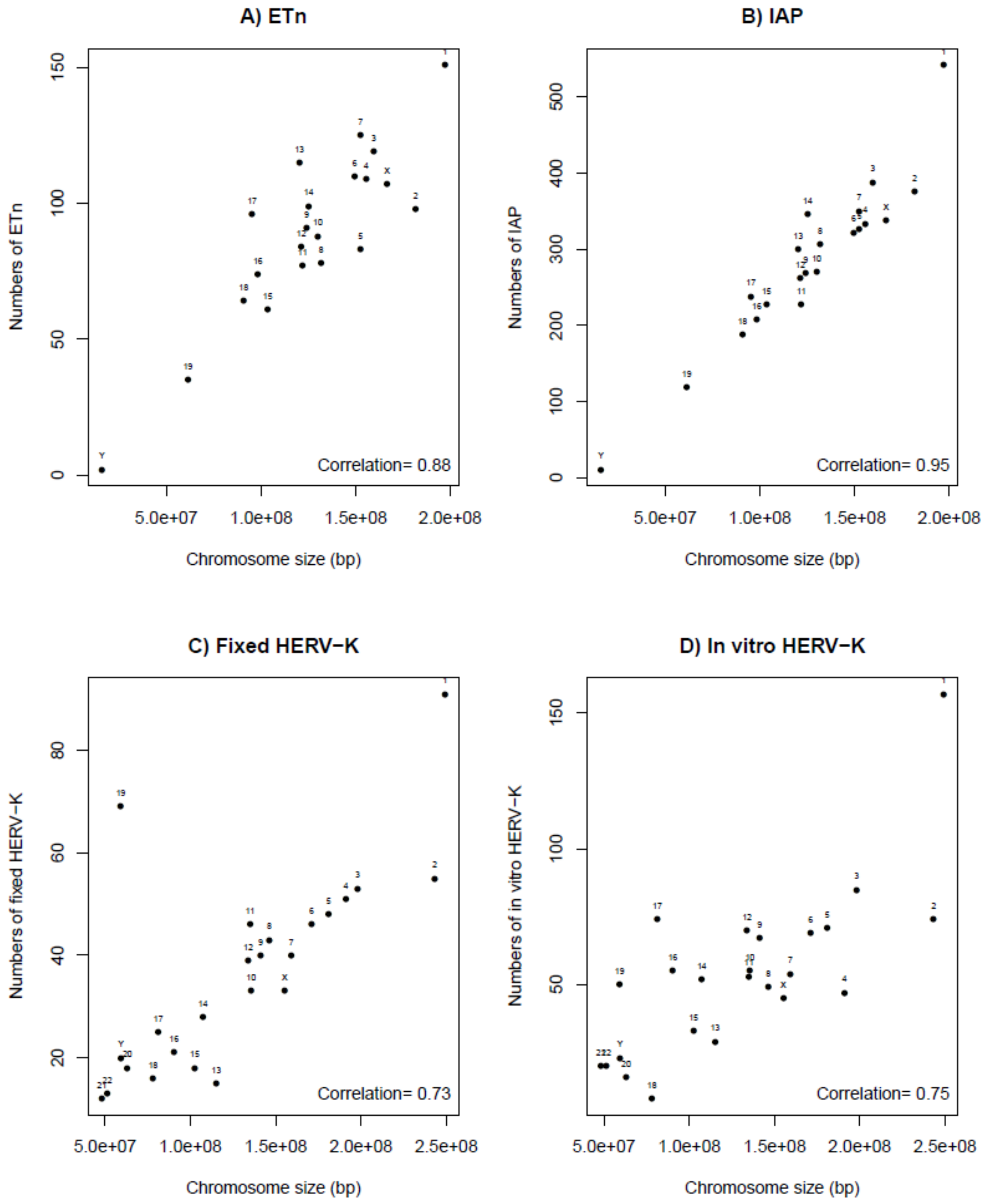

Figure B. Hierarchical clustering of features close to fixed ETn and control regions (polymorphic ETn, fixed and polymorphic IAP clustering is similar to fixed ETn). WA= weighted averages, bp= base pairs, TSS= transcription start sites. Underlined is the feature from each cluster that was used in the analyses.

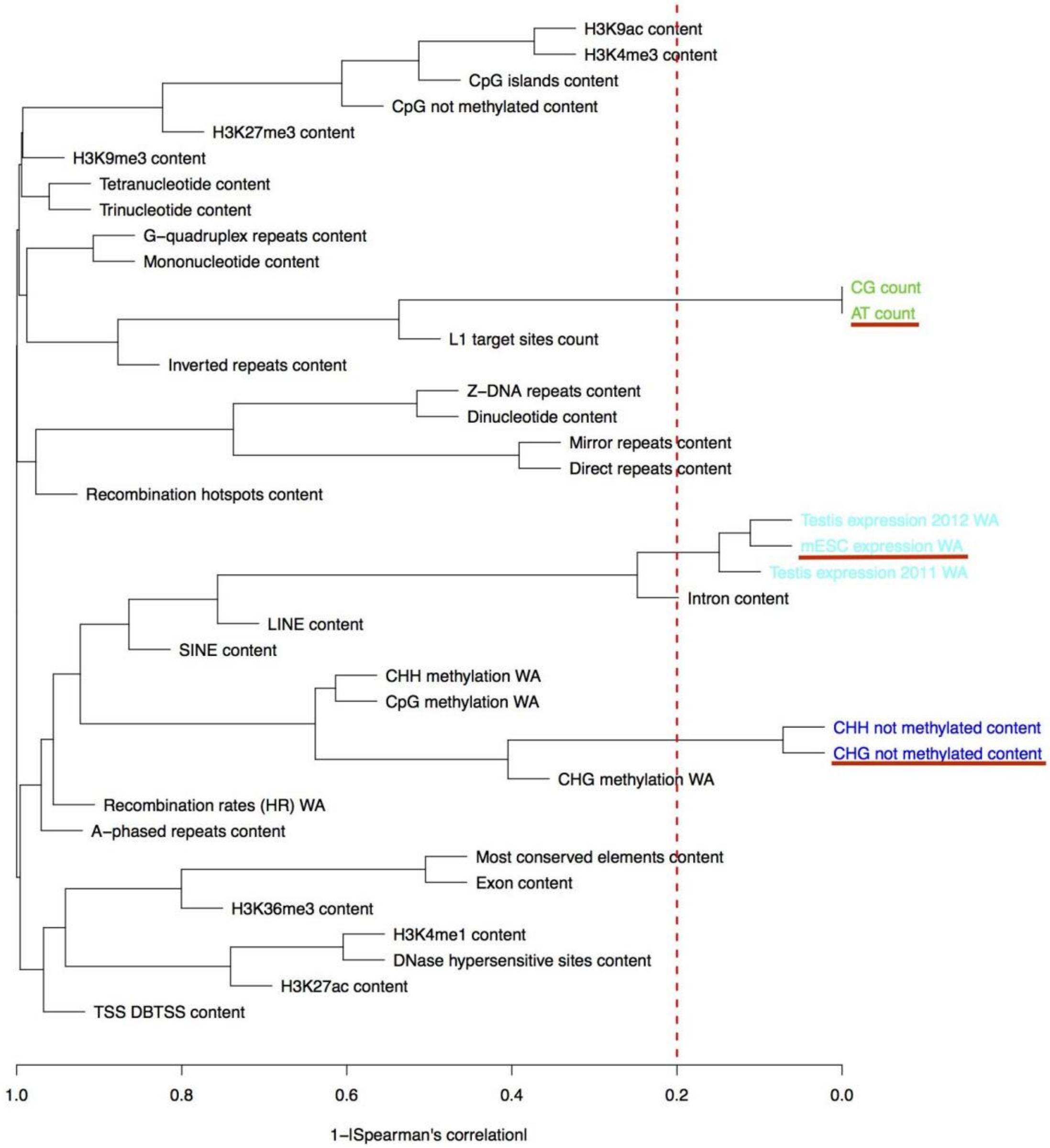

Figure C. Hierarchical clustering of features close to fixed HERV-K and controls regions (the clustering for *in vitro* HERV-Ks is similar to fixed HERV-K). WA= weighted averages, bp= base pairs, TSS= transcription start sites. Underlined is the feature from each cluster that was used in the analyses.

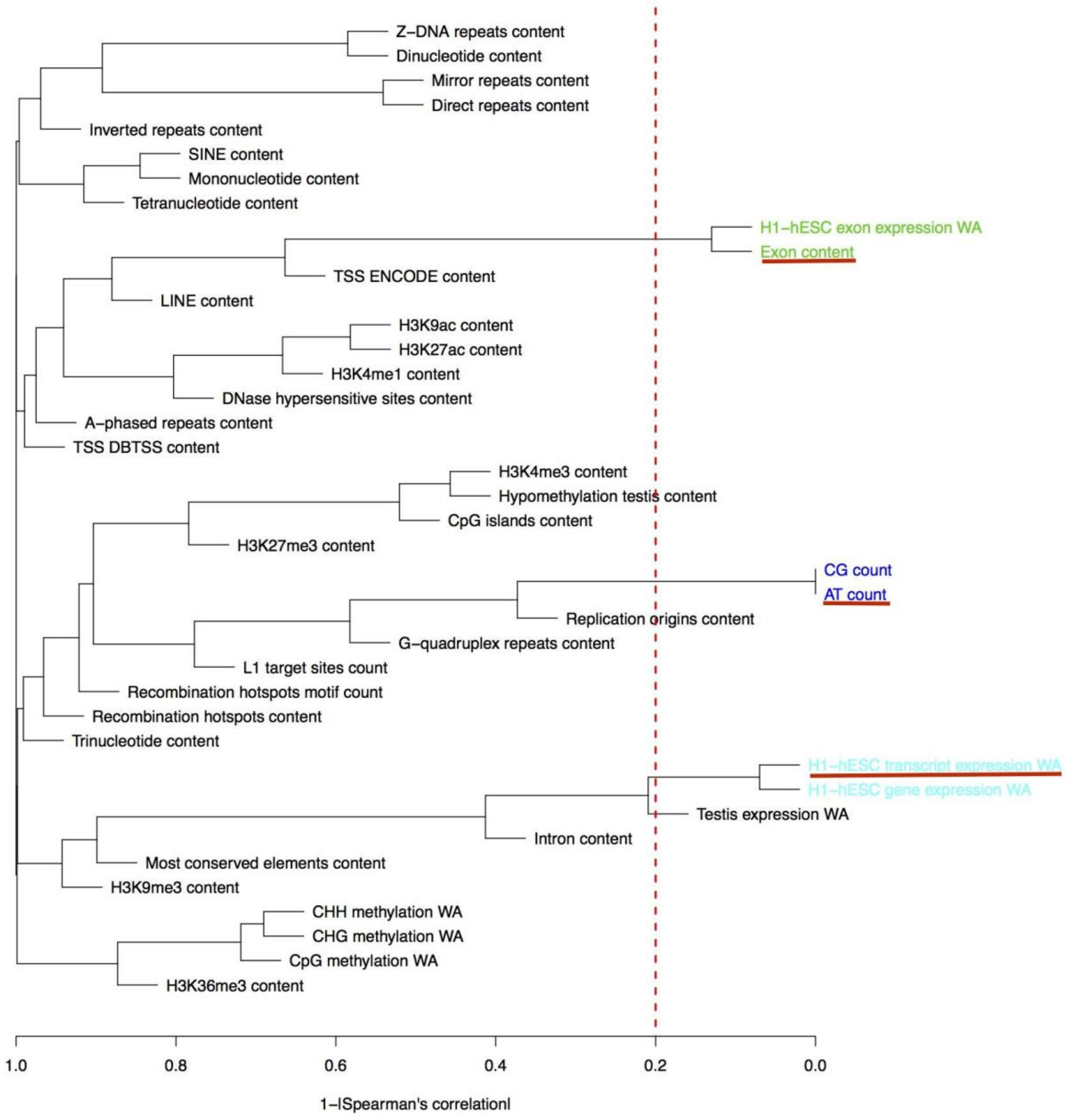

Figure D. Plot of features in one fixed HERV-K, one *in vitro* HERV-K and one human control randomly chosen regions. Colors indicate the different groups the regions belong to.

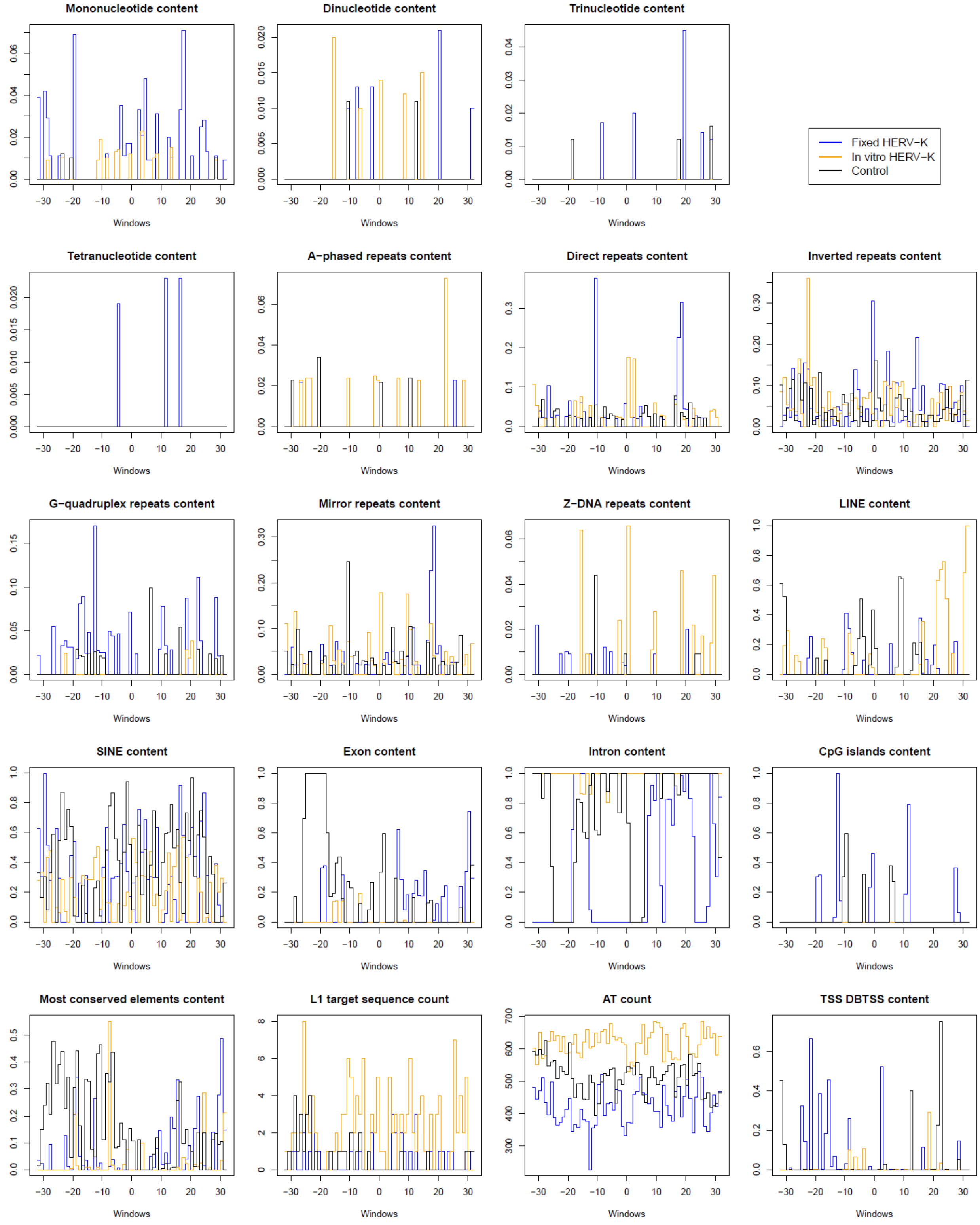

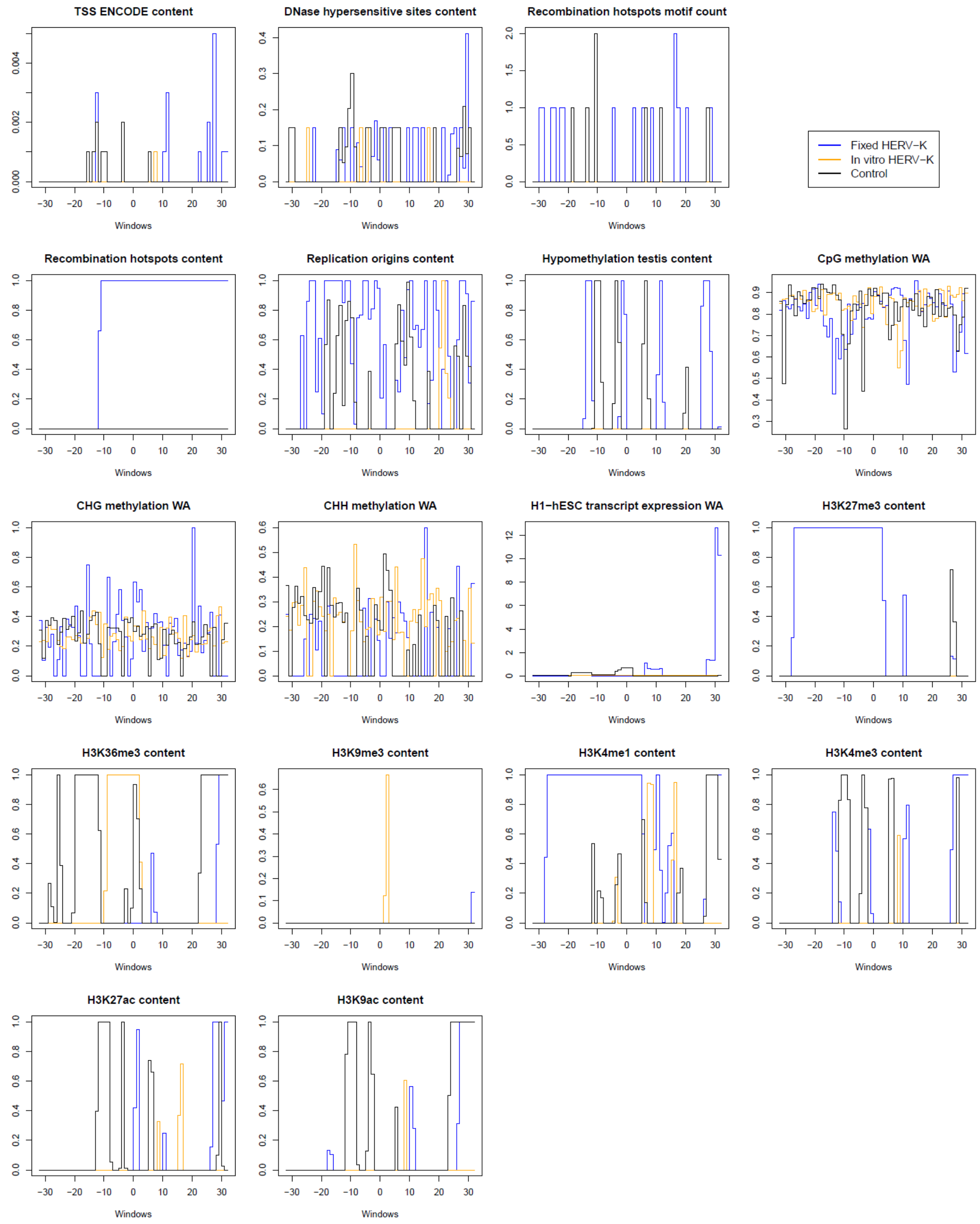

TSS ENCODE content
DNase hypersensitive sites content
Recombination hotspots motif count
Fixed HERV−K
In vitro HERV−K
Control
Recombination hotspots content
Replication origins content
Hypomethylation testis content
CpG methylation WA
CHG methylation WA
CHH methylation WA
H1−hESC transcript expression WA
H3K27me3 content
H3K36me3 content
H3K9me3 content
H3K4me1 content
H3K4me3 content
H3K27ac content
H3K9ac content
Windows

Figure E. Plot of features in one fixed ETn, one polymorphic ETn, one fixed IAP, one polymorphic IAP and one mouse control randomly chosen regions. Colors indicate the different groups the regions belong to.

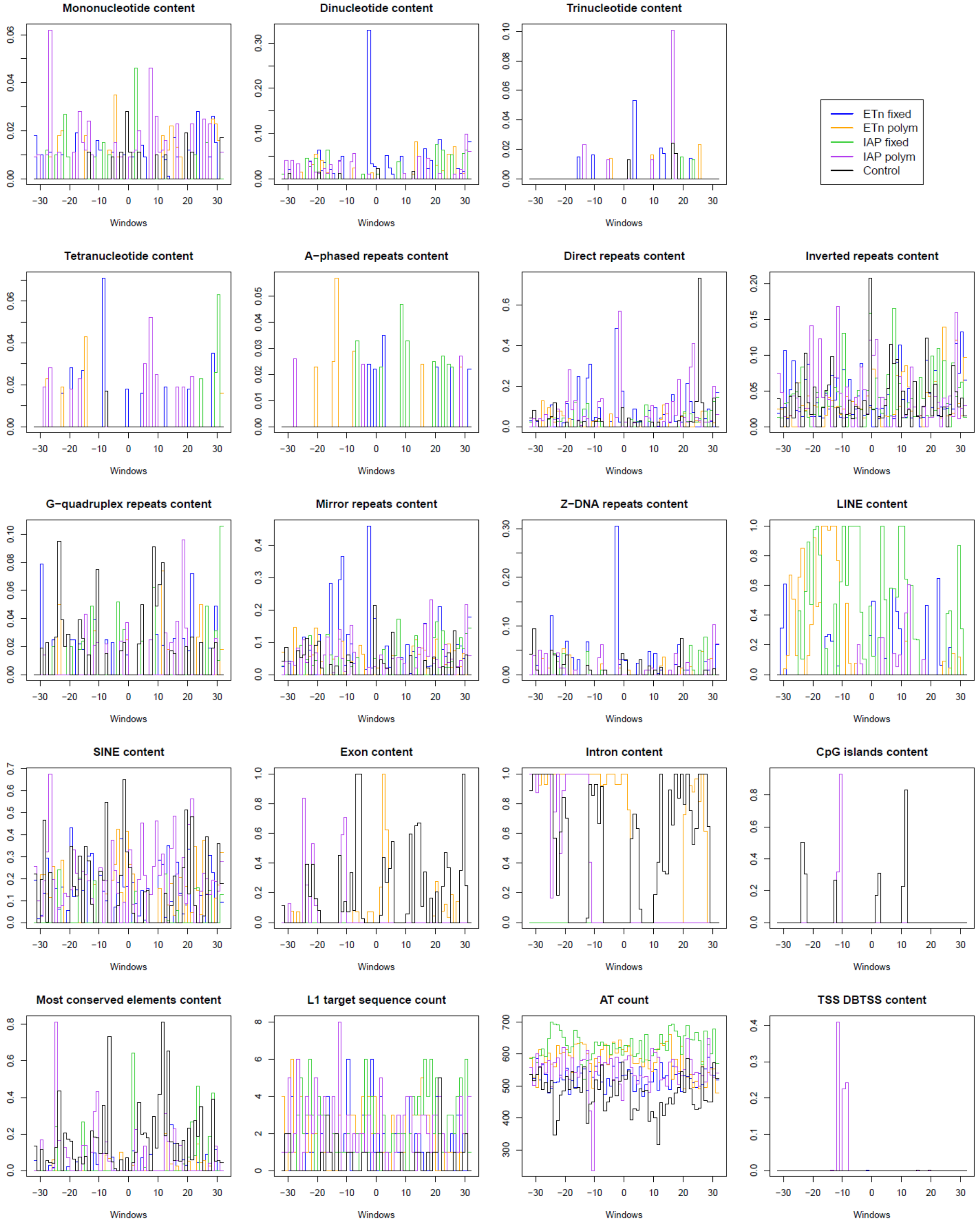

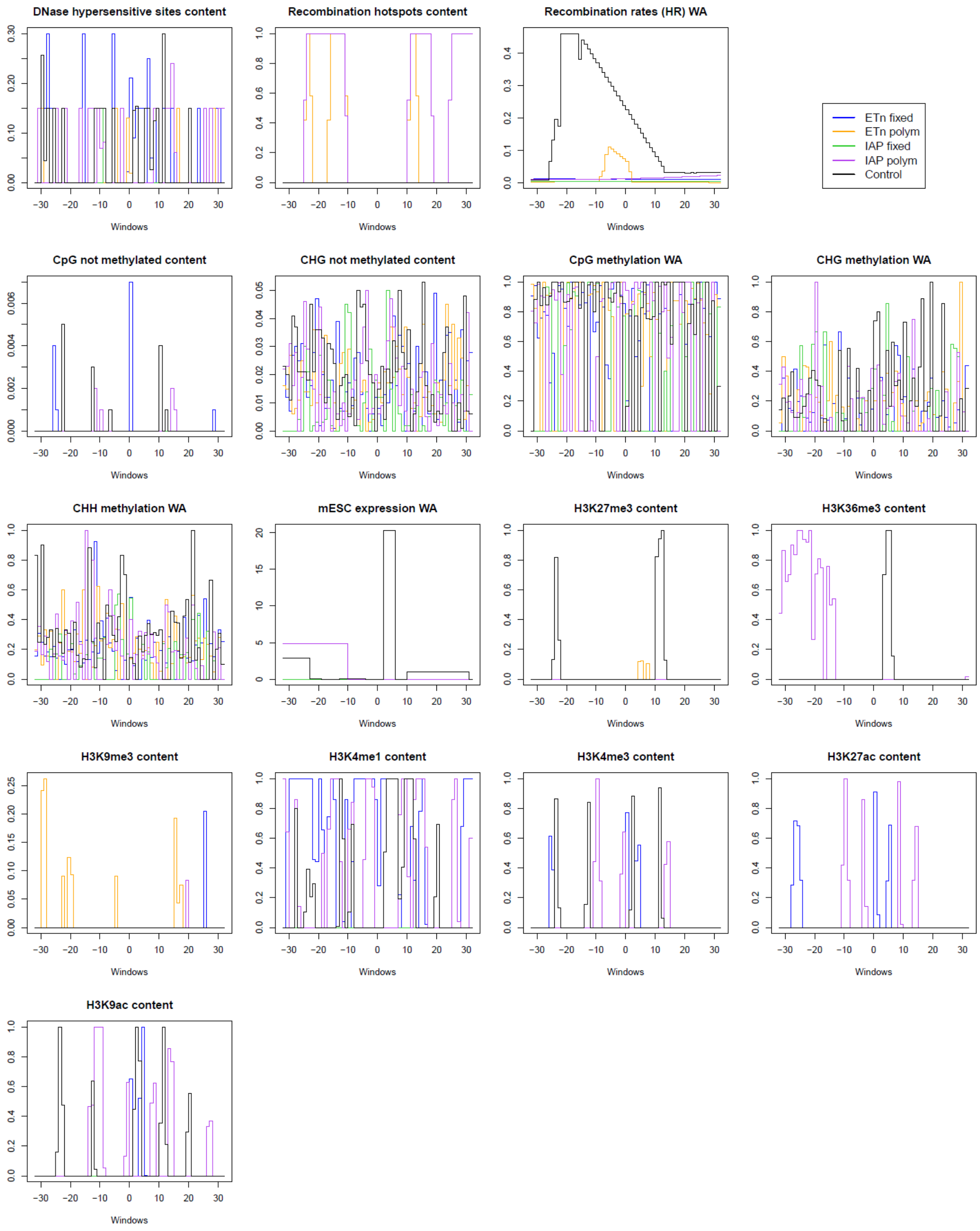
DNase hypersensitive sites content
Recombination hotspots content
Recombination rates (HR) WA
ETn fixed
ETn polym
IAP fixed
IAP polym
Control
CpG not methylated content
CHG not methylated content
CpG methylation WA
CHG methylation WA
CHH methylation WA
mESC expression WA
H3K27me3 content
H3K36me3 content
H3K9me3 content
H3K4me1 content
H3K4me3 content
H3K27ac content
H3K9ac content
Windows

Figure F. Significant genomic features for ITP of comparisons fixed ETn vs. control with test statistics: A) sample mean difference, B) sample median difference, C) sample variance ratio.

A)

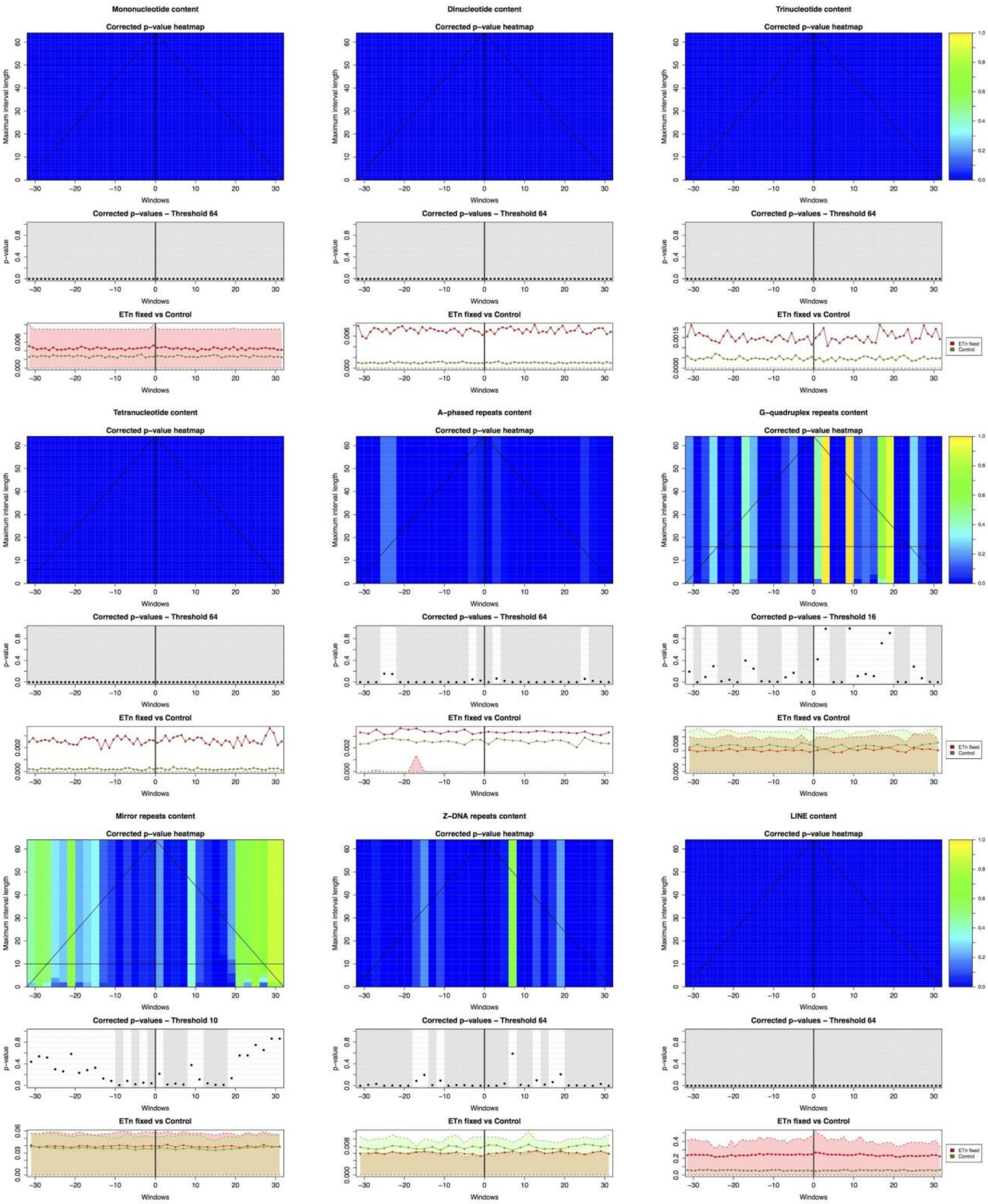

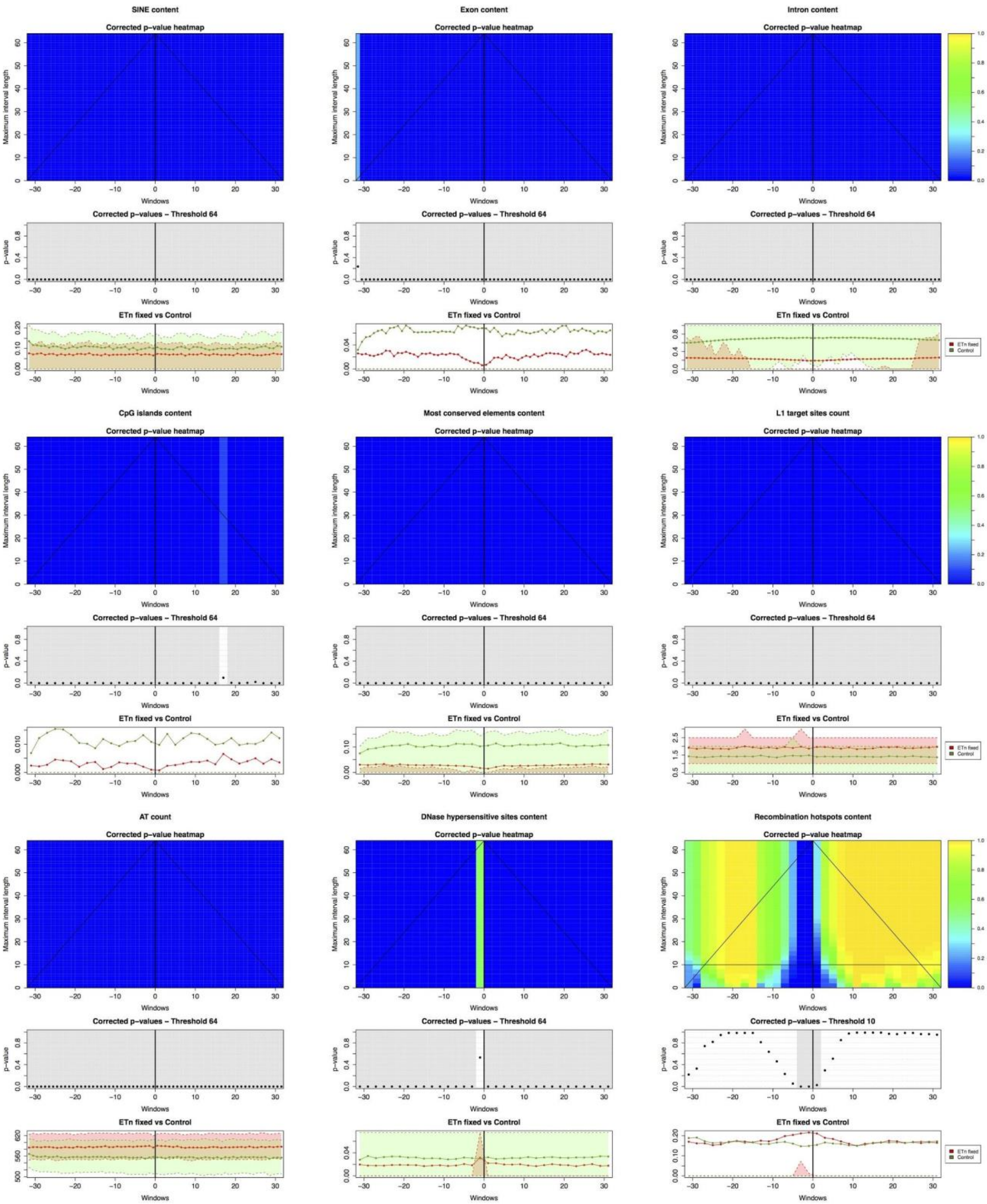

SINE content
Exon content
Intron content
CpG islands content
Most conserved elements content
L1 target sites count
AT count
DNase hypersensitive sites content
Recombination hotspots content
Corrected p–value heatmap
Corrected p–values – Threshold 64
Corrected p–values – Threshold 10
ETn fixed vs Control
Maximum interval length
p–value
Windows
ETn fixed
Control

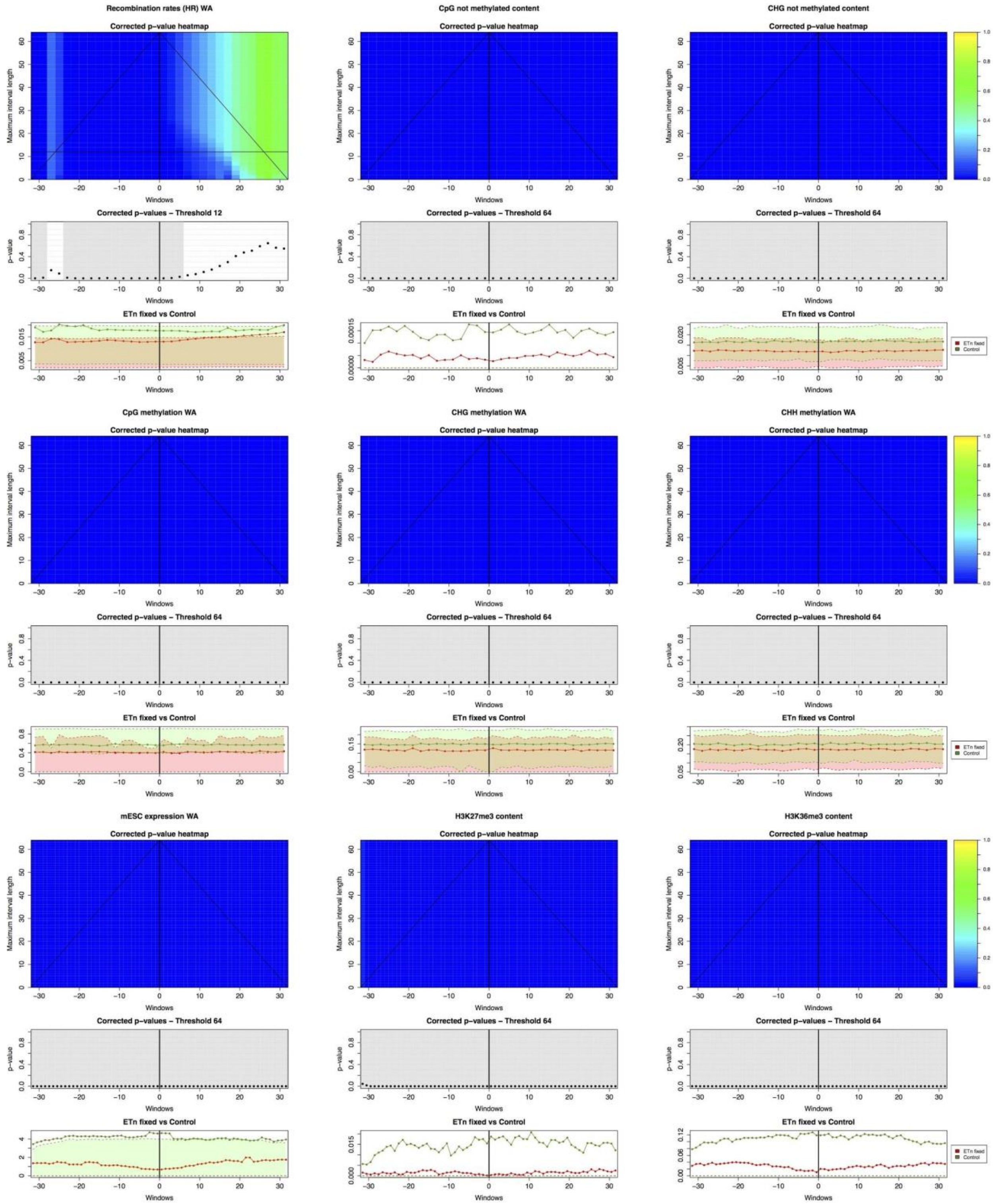

Recombination rates (HR) WA
CpG not methylated content
CHG not methylated content
CpG methylation WA
CHG methylation WA
CHH methylation WA
mESC expression WA
H3K27me3 content
H3K36me3 content
Corrected p−value heatmap
Maximum interval length
Windows
Corrected p−values − Threshold 12
Corrected p−values − Threshold 64
p−value
ETn fixed vs Control
ETn fixed
Control

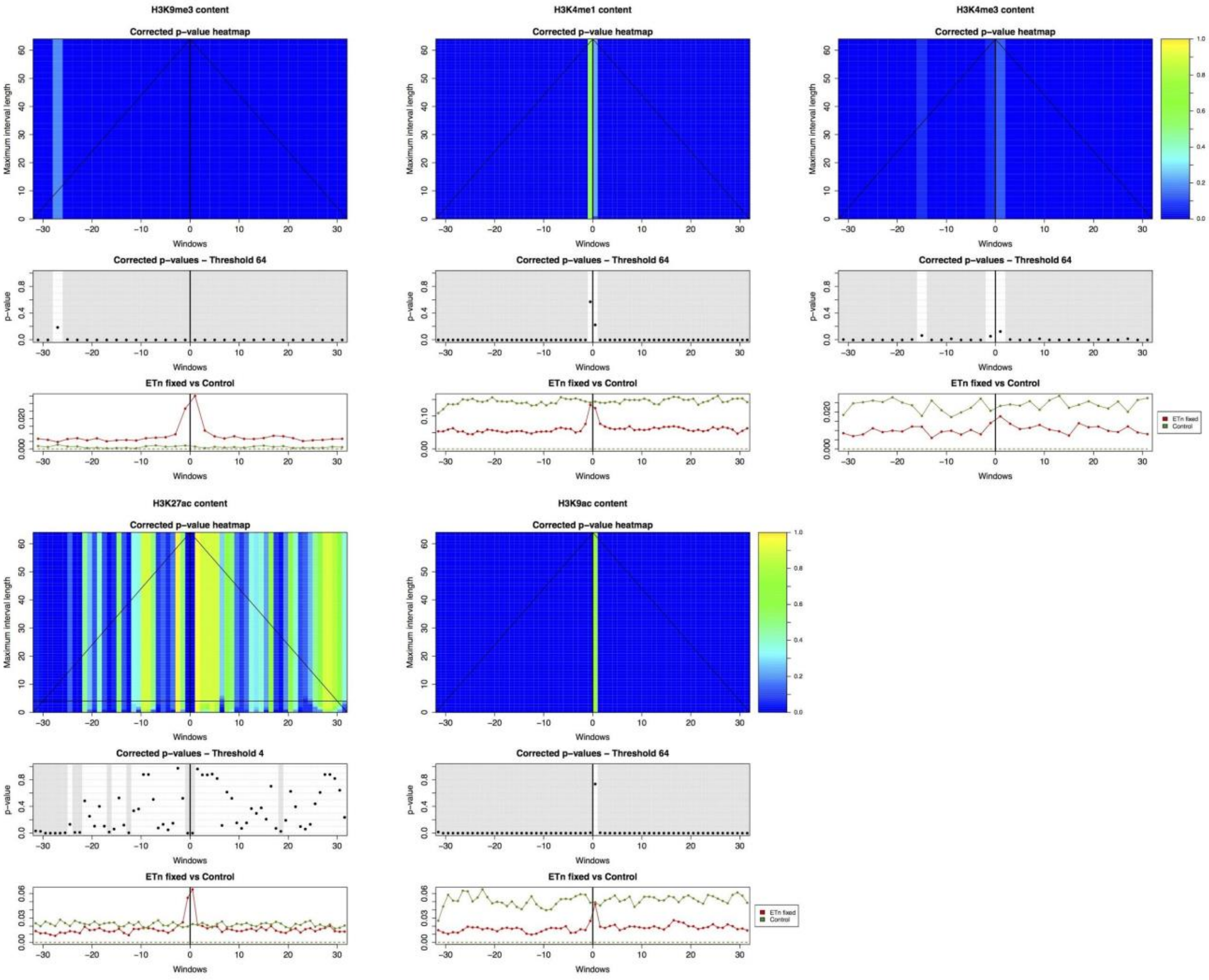
H3K9me3 content
Corrected p−value heatmap
Maximum interval length
Windows
Corrected p−values − Threshold 64
p−value
Windows
ETn fixed vs Control
Windows
H3K4me1 content
Corrected p−value heatmap
Maximum interval length
Windows
Corrected p−values − Threshold 64
p−value
Windows
ETn fixed vs Control
Windows
H3K4me3 content
Corrected p−value heatmap
Maximum interval length
Windows
Corrected p−values − Threshold 64
p−value
Windows
ETn fixed vs Control
Windows
ETn fixed
Control
H3K27ac content
Corrected p−value heatmap
Maximum interval length
Windows
Corrected p−values − Threshold 4
p−value
Windows
ETn fixed vs Control
Windows
H3K9ac content
Corrected p−value heatmap
Maximum interval length
Windows
Corrected p−values − Threshold 64
p−value
Windows
ETn fixed vs Control
Windows
ETn fixed
Control

B)

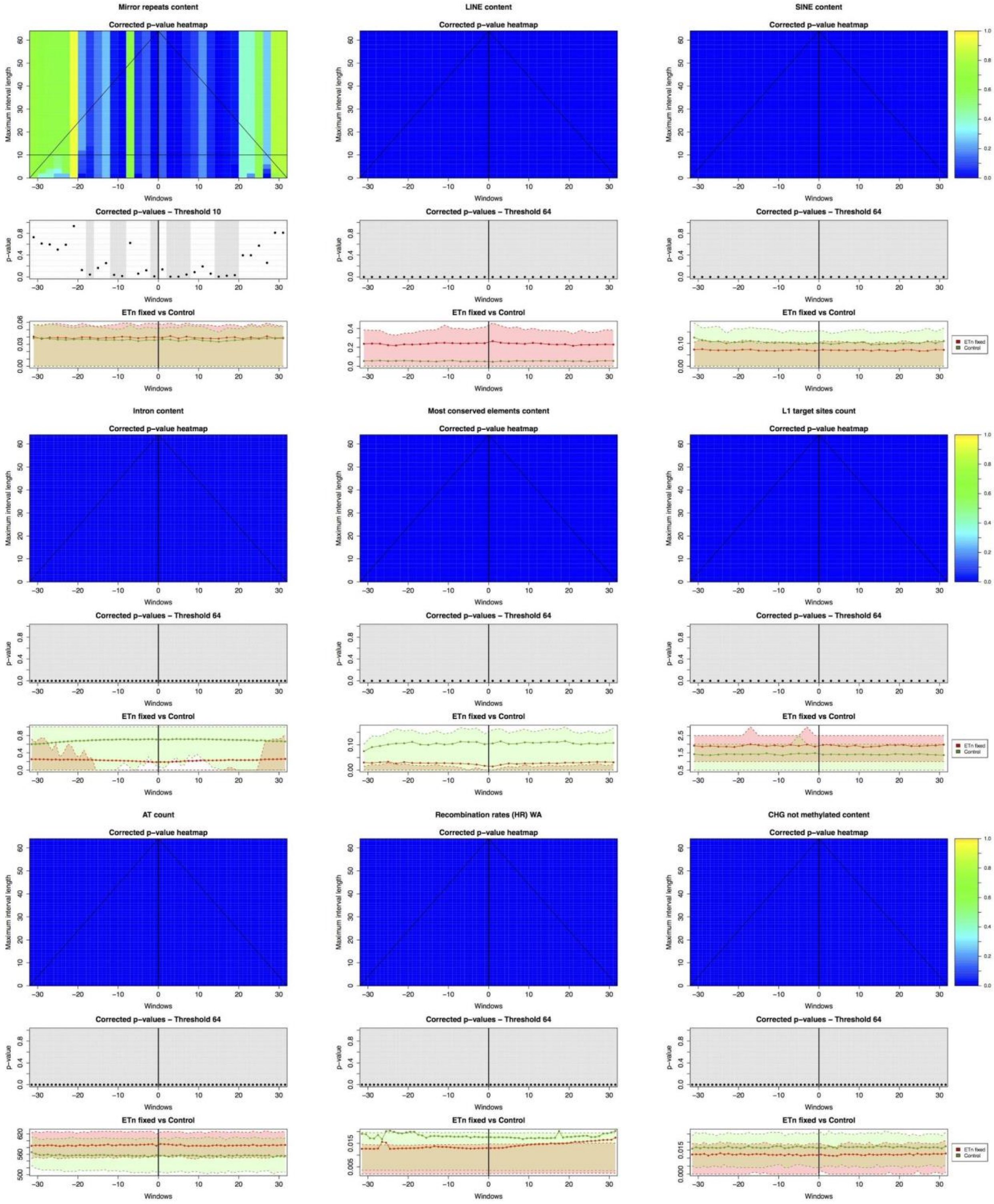

Mirror repeats content
LINE content
SINE content
Corrected p−value heatmap
Maximum interval length
Windows
Corrected p−values − Threshold 10
Corrected p−values − Threshold 64
p−value
ETn fixed vs Control
ETn fixed
Control
Intron content
Most conserved elements content
L1 target sites count
AT count
Recombination rates (HR) WA
CHG not methylated content

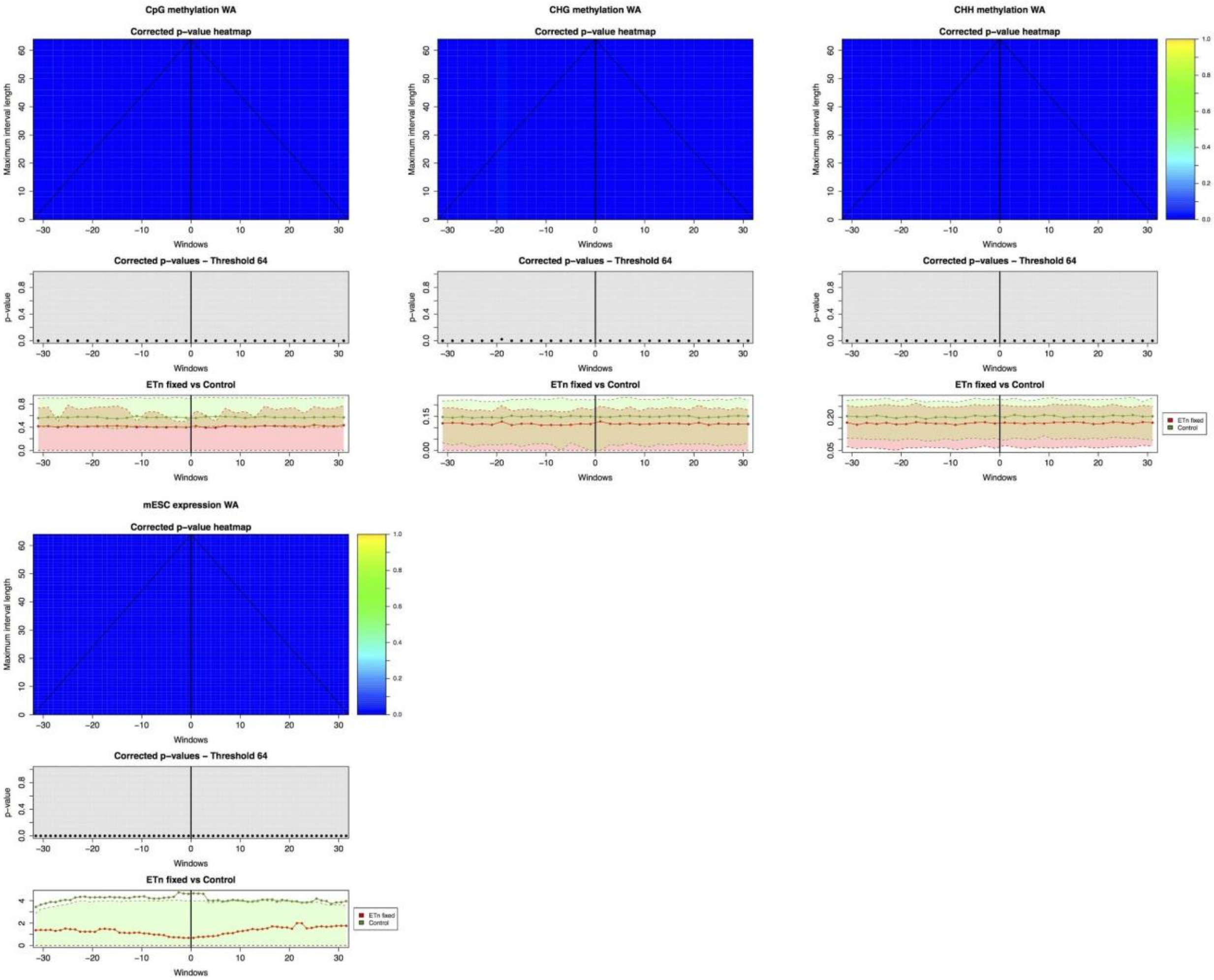
CpG methylation WA
CHG methylation WA
CHH methylation WA
mESC expression WA
Corrected p−value heatmap
Maximum interval length
Windows
Corrected p−values − Threshold 64
p−value
ETn fixed vs Control
ETn fixed
Control

C)

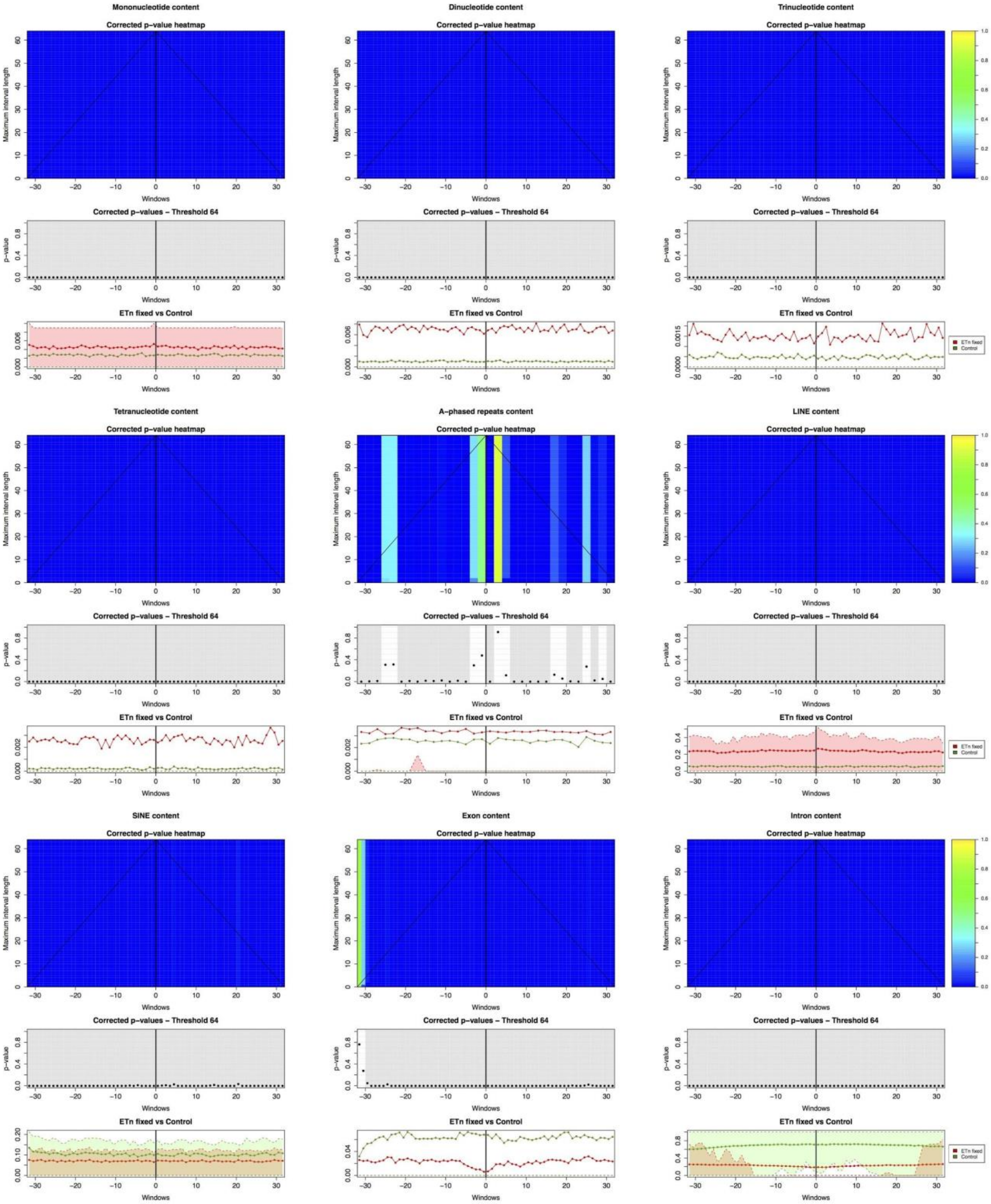
Mononucleotide content
Dinucleotide content
Trinucleotide content
Tetranucleotide content
A−phased repeats content
LINE content
SINE content
Exon content
Intron content
Corrected p−value heatmap
Maximum interval length
Windows
Corrected p−values − Threshold 64
p−value
ETn fixed vs Control
ETn fixed
Control

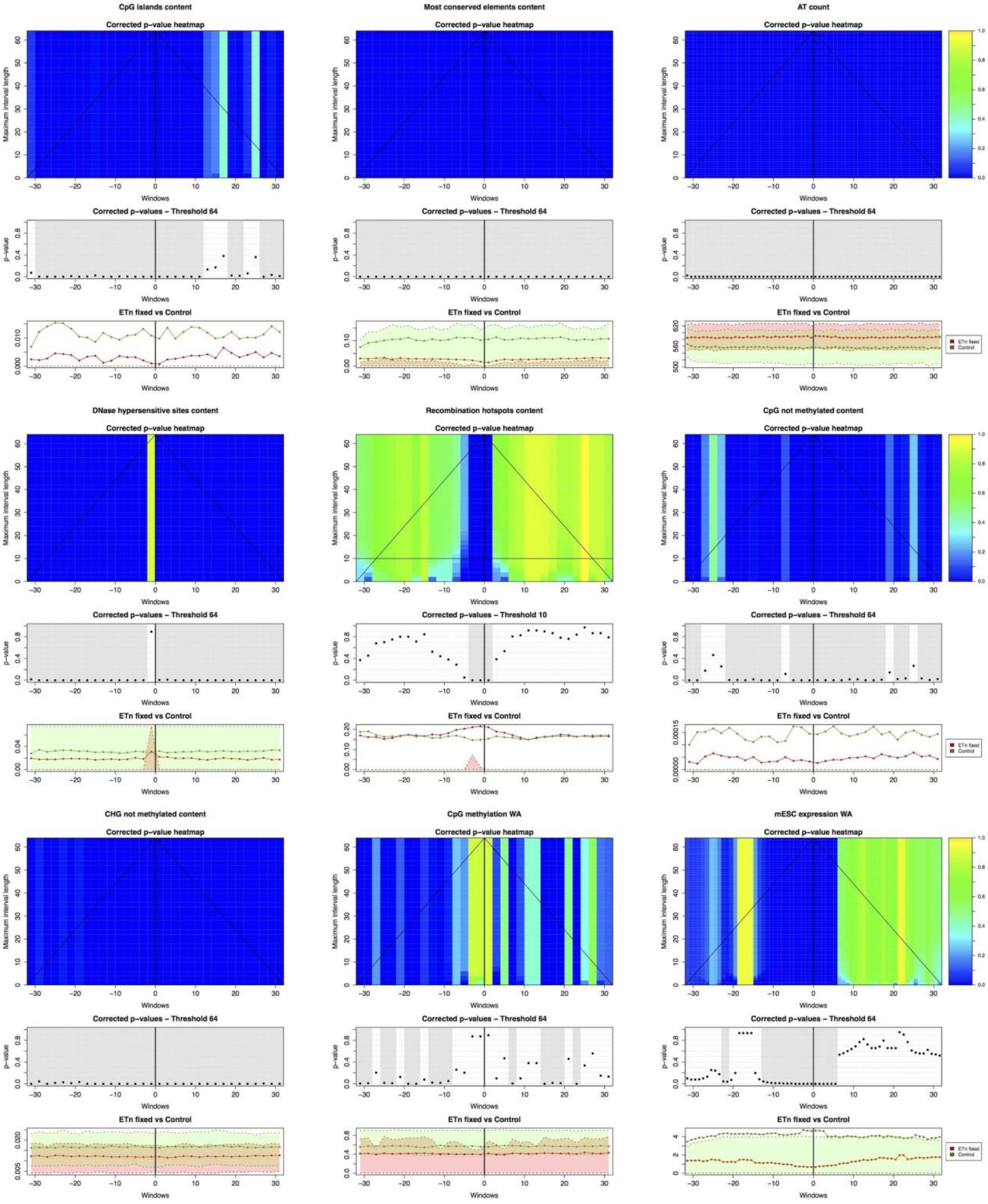
CpG islands content
Most conserved elements content
AT count
DNase hypersensitive sites content
Recombination hotspots content
CpG not methylated content
CHG not methylated content
CpG methylation WA
mESC expression WA
Corrected p-value heatmap
Maximum interval length
Windows
Corrected p-values - Threshold 64
Corrected p-values - Threshold 10
p-value
ETn fixed vs Control
ETn fixed
Control

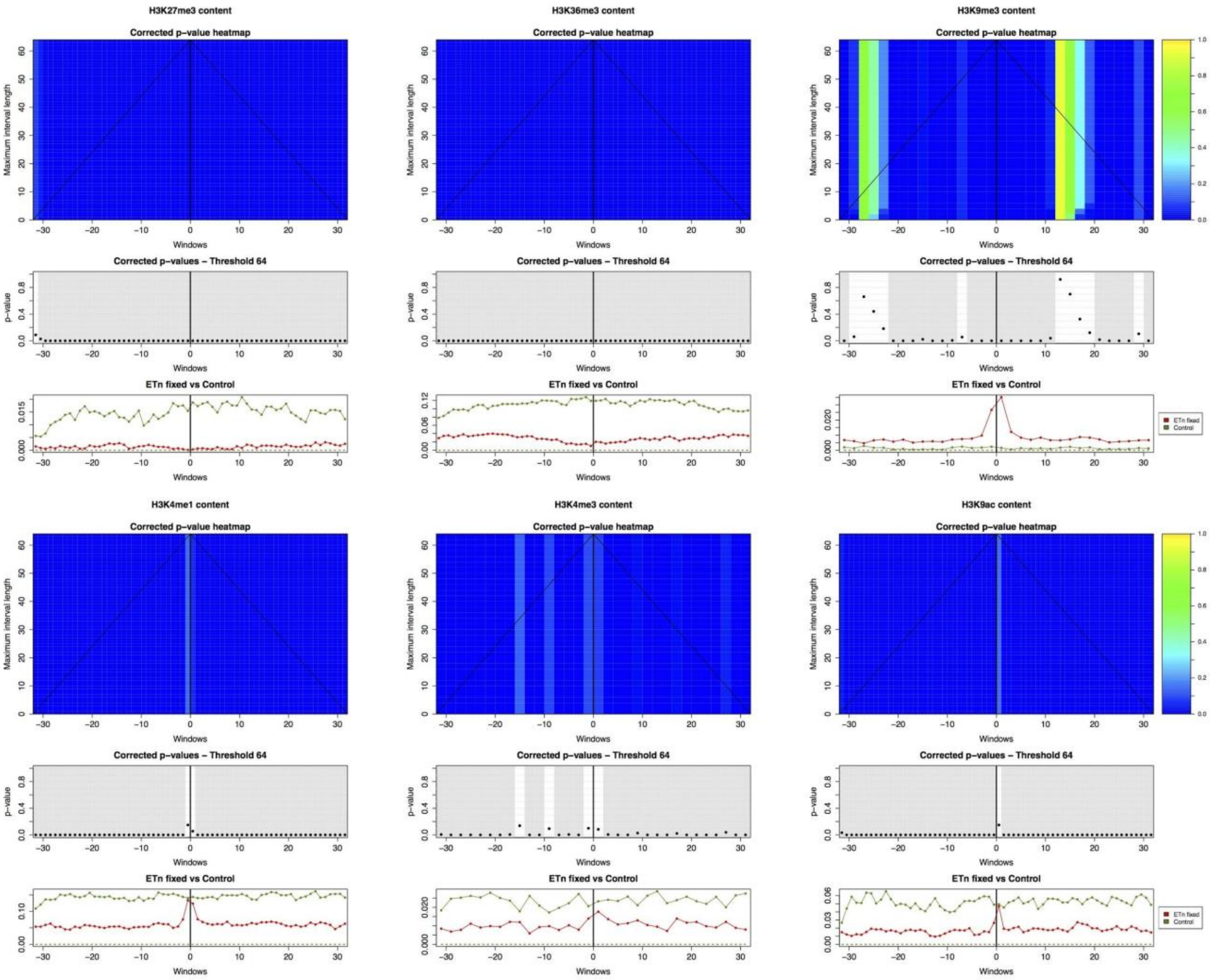

H3K27me3 content
H3K36me3 content
H3K9me3 content
Corrected p−value heatmap
Maximum interval length
Windows
Corrected p−values − Threshold 64
p−value
ETn fixed vs Control
ETn fixed
Control
H3K4me1 content
H3K4me3 content
H3K9ac content

Figure G. Significant genomic features for ITP of comparisons polymorphic ETn vs. control with test statistics: A) sample mean difference, B) sample median difference, C) sample variance ratio.

A)

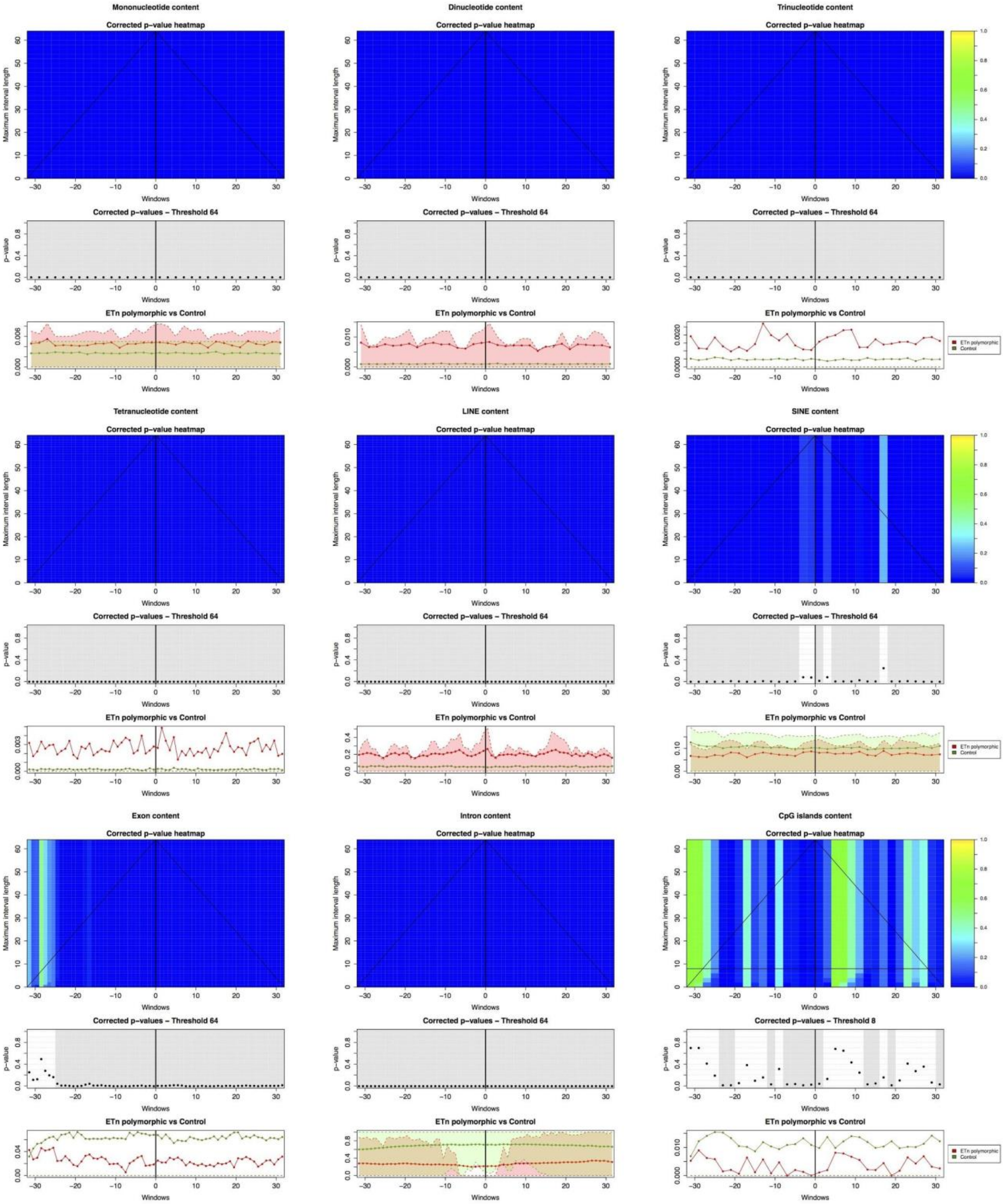

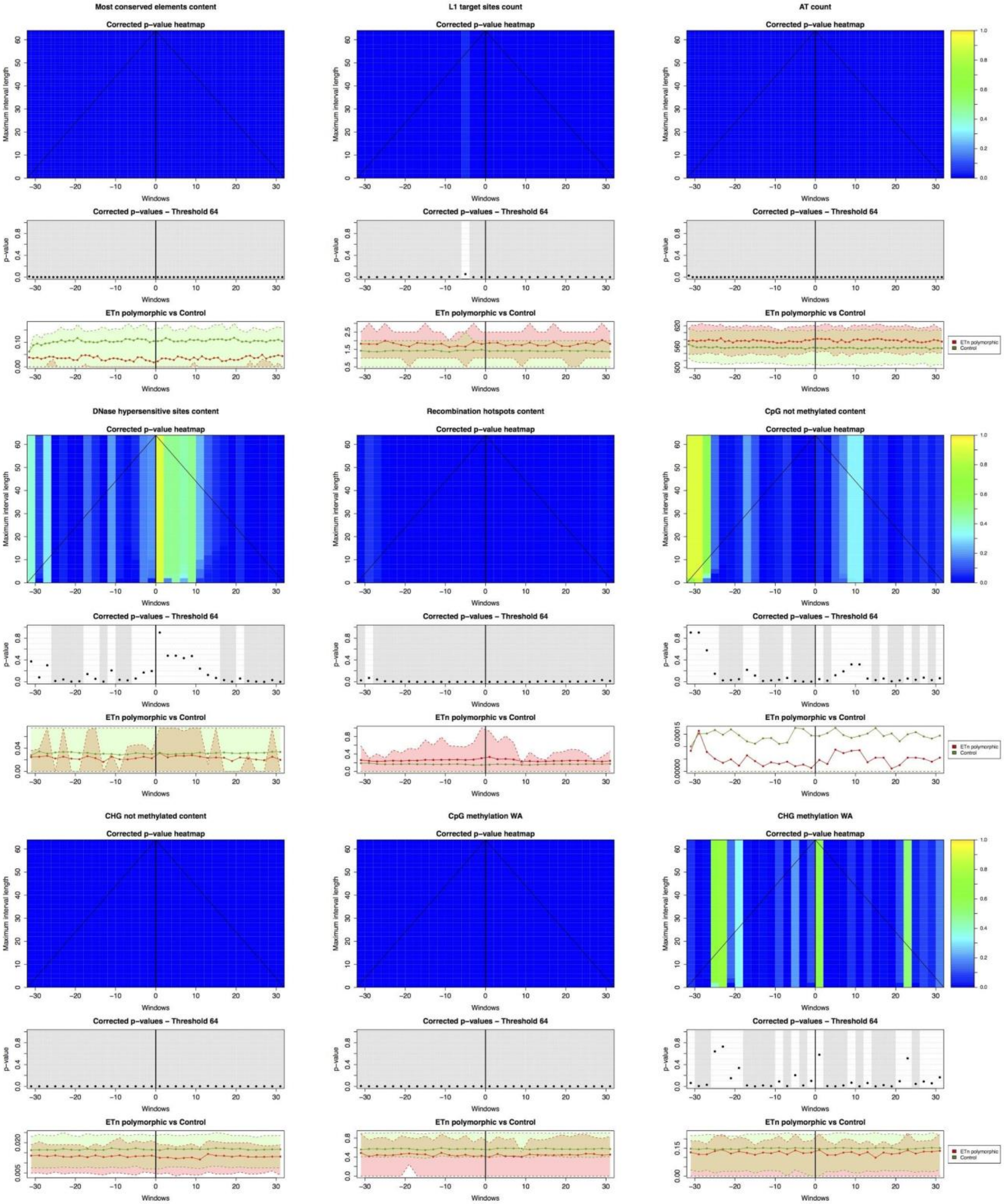
Most conserved elements content
L1 target sites count
AT count
DNase hypersensitive sites content
Recombination hotspots content
CpG not methylated content
CHG not methylated content
CpG methylation WA
CHG methylation WA
Corrected p−value heatmap
Maximum interval length
Windows
Corrected p−values − Threshold 64
p−value
ETn polymorphic vs Control
ETn polymorphic
Control

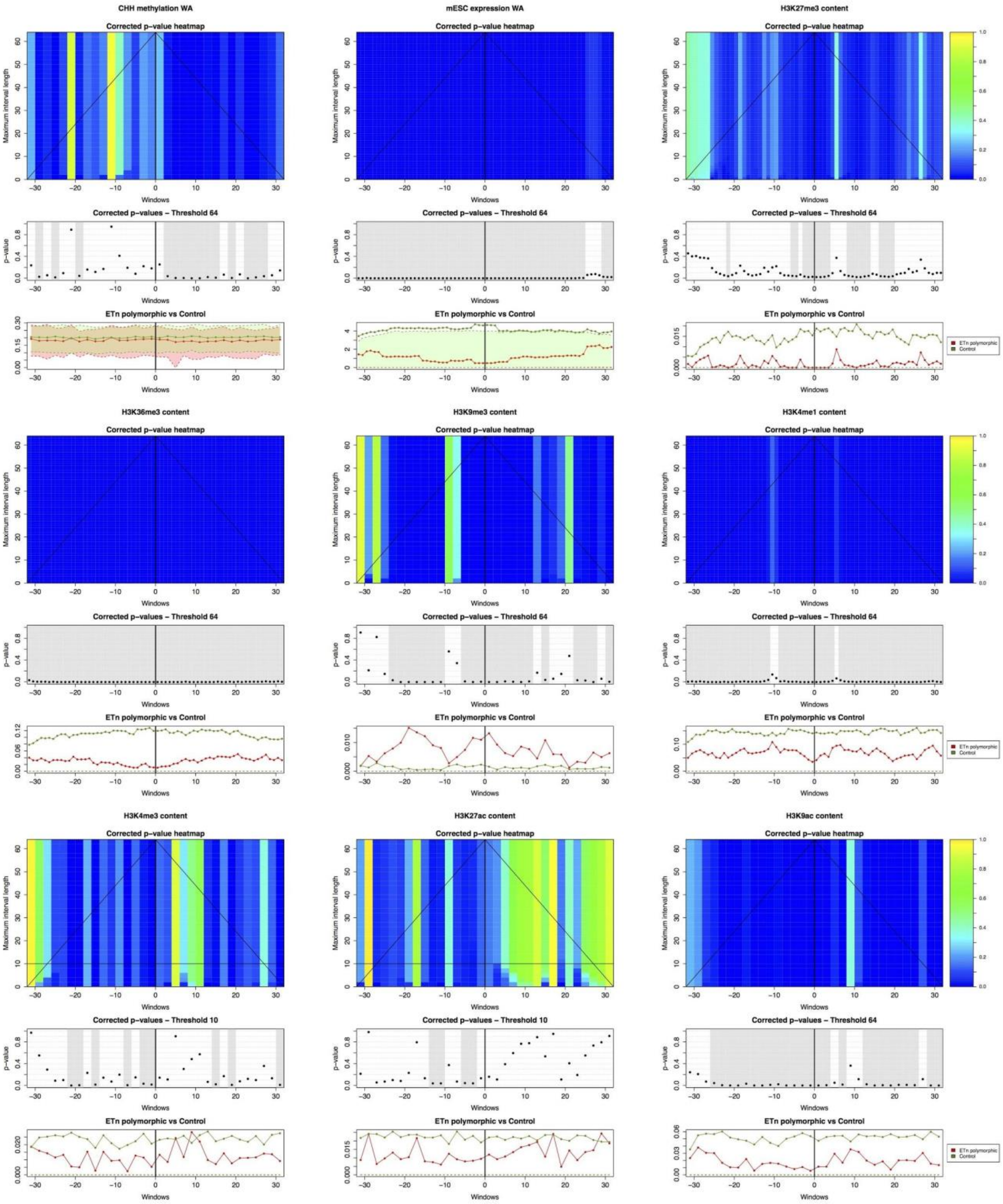
CHH methylation WA
Corrected p–value heatmap
Maximum interval length
Windows
Corrected p–values – Threshold 64
p–value
ETn polymorphic vs Control
mESC expression WA
Corrected p–value heatmap
Corrected p–values – Threshold 64
ETn polymorphic vs Control
H3K27me3 content
Corrected p–value heatmap
Corrected p–values – Threshold 64
ETn polymorphic vs Control
ETn polymorphic
Control
H3K36me3 content
Corrected p–value heatmap
Corrected p–values – Threshold 64
ETn polymorphic vs Control
H3K9me3 content
Corrected p–value heatmap
Corrected p–values – Threshold 64
ETn polymorphic vs Control
H3K4me1 content
Corrected p–value heatmap
Corrected p–values – Threshold 64
ETn polymorphic vs Control
ETn polymorphic
Control
H3K4me3 content
Corrected p–value heatmap
Corrected p–values – Threshold 10
ETn polymorphic vs Control
H3K27ac content
Corrected p–value heatmap
Corrected p–values – Threshold 10
ETn polymorphic vs Control
H3K9ac content
Corrected p–value heatmap
Corrected p–values – Threshold 64
ETn polymorphic vs Control
ETn polymorphic
Control

B)

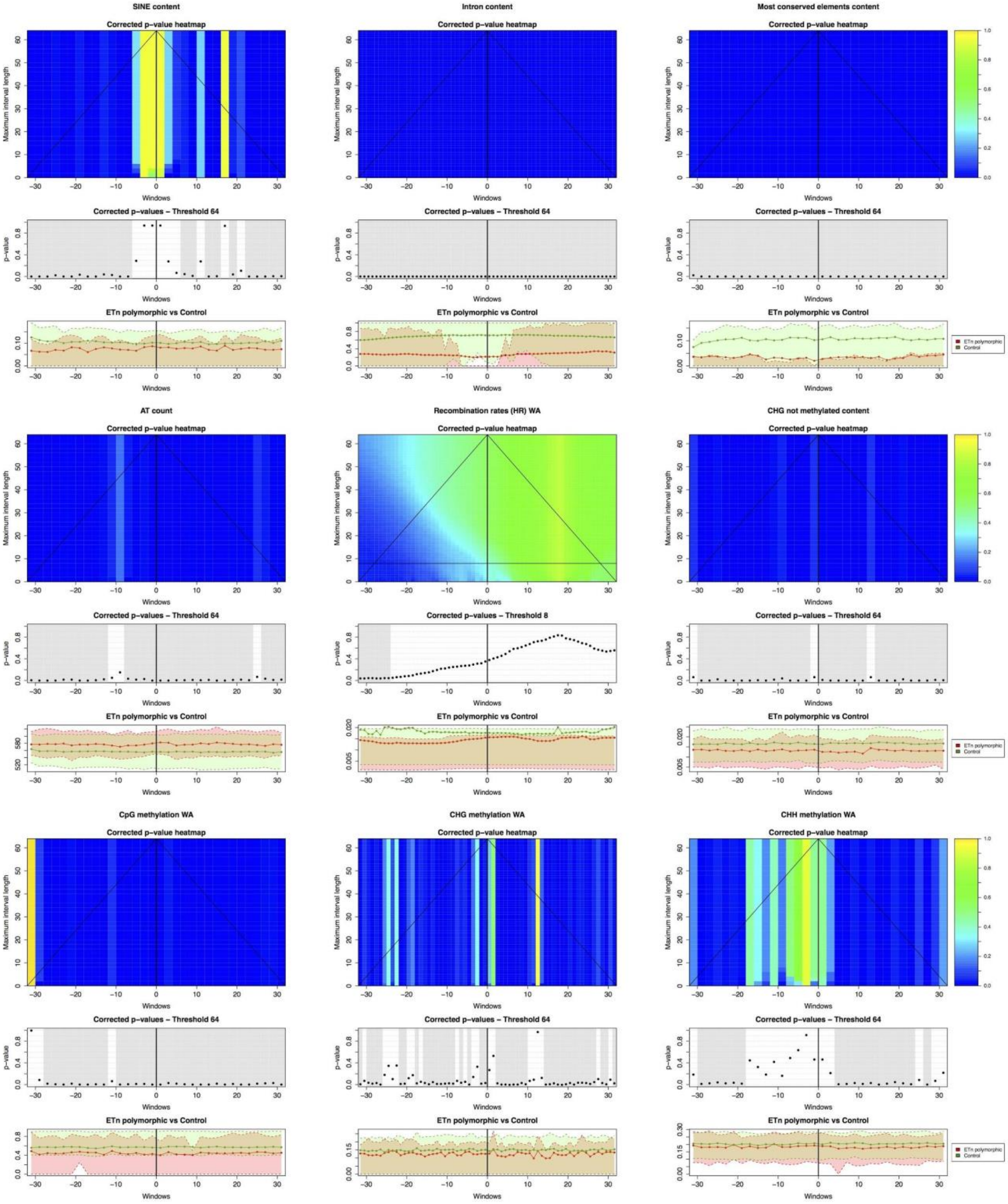
SINE content
Intron content
Most conserved elements content
AT count
Recombination rates (HR) WA
CHG not methylated content
CpG methylation WA
CHG methylation WA
CHH methylation WA
Corrected p–value heatmap
Maximum interval length
Windows
Corrected p–values – Threshold 64
Corrected p–values – Threshold 8
p–value
ETn polymorphic vs Control
ETn polymorphic
Control

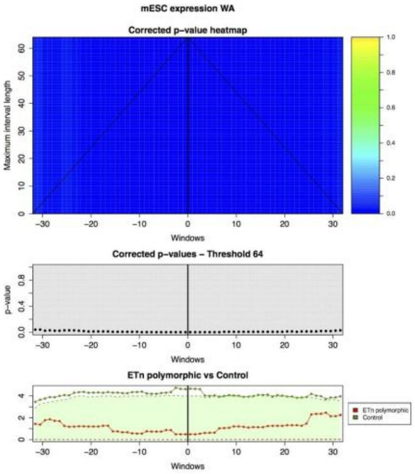
mESC expression WA
Corrected p–value heatmap
Maximum interval length
Windows
Corrected p–values – Threshold 64
p–value
Windows
ETn polymorphic vs Control
ETn polymorphic
Control
Windows

C)

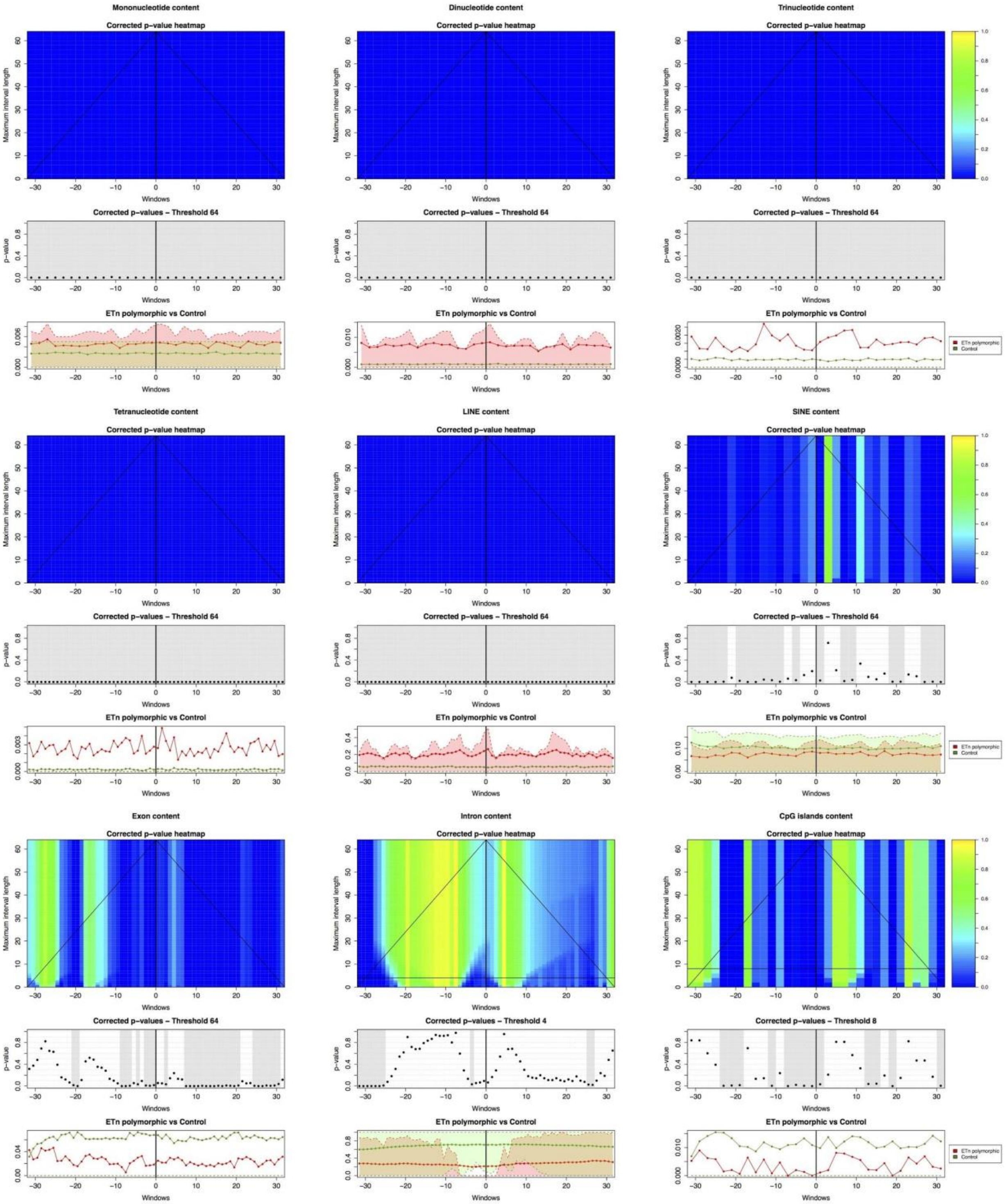
Mononucleotide content
Dinucleotide content
Trinucleotide content
Tetranucleotide content
LINE content
SINE content
Exon content
Intron content
CpG islands content
Corrected p-value heatmap
Maximum interval length
Windows
Corrected p-values - Threshold 64
Corrected p-values - Threshold 4
Corrected p-values - Threshold 8
p-value
ETn polymorphic vs Control
ETn polymorphic
Control

Most conserved elements content

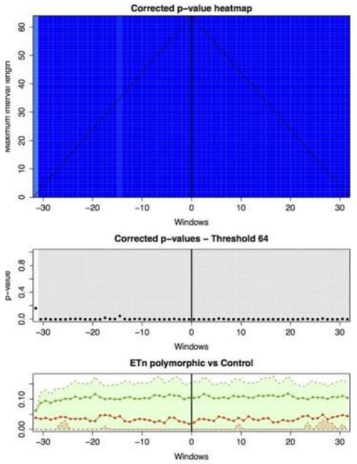

AT count

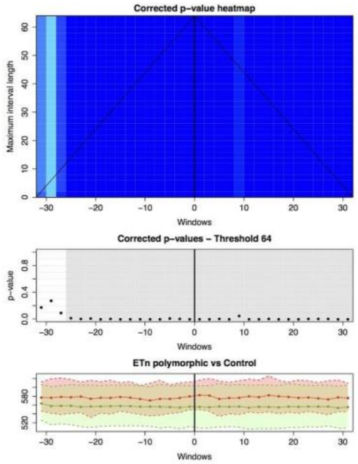

DNase hypersensitive sites content

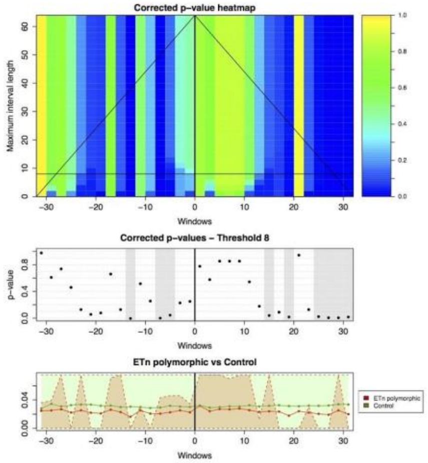

Recombination hotspots content

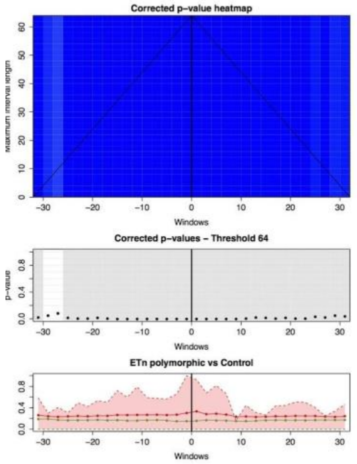

CpG not methylated content

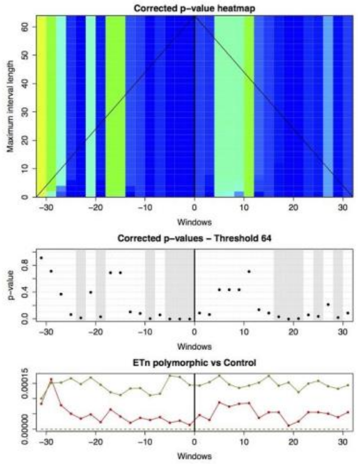

mESC expression WA

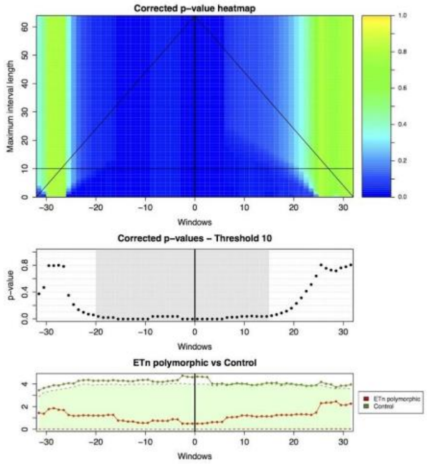

H3K27me3 content

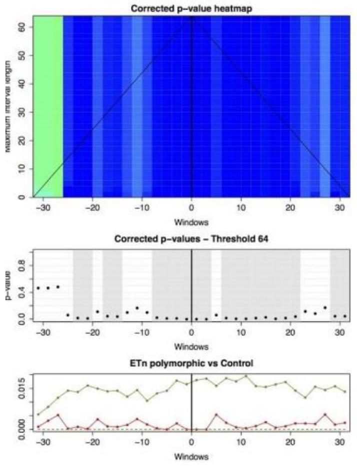

H3K36me3 content

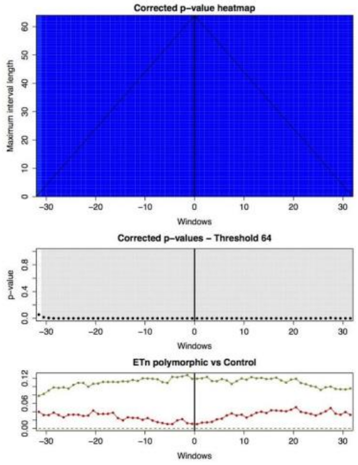

H3K4me3 content

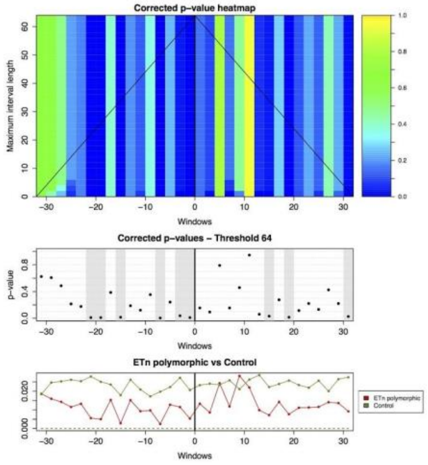

H3K9ac content

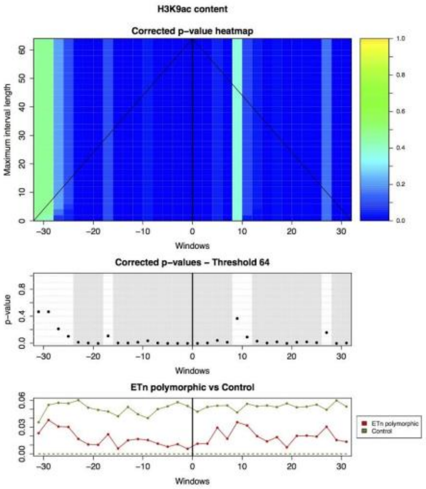

H3K9ac content
Corrected p−value heatmap
Maximum interval length
Windows
Corrected p−values − Threshold 64
p−value
Windows
ETn polymorphic vs Control
ETn polymorphic
Control
Windows

Figure H. Significant genomic features for ITP of comparisons fixed vs. polymorphic ETn with test statistics: A) sample mean difference, B) sample median difference, C) sample variance ratio.

A)

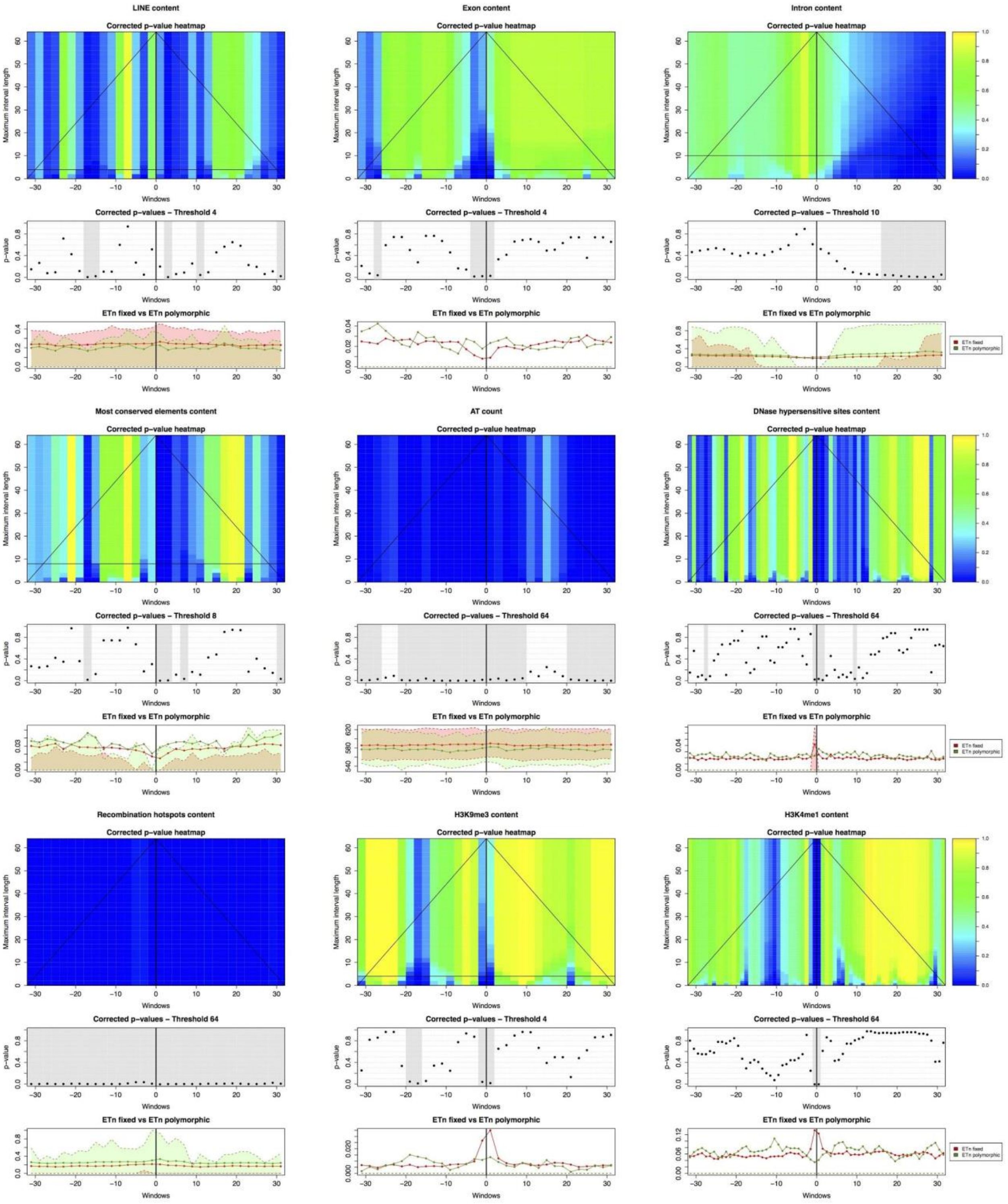

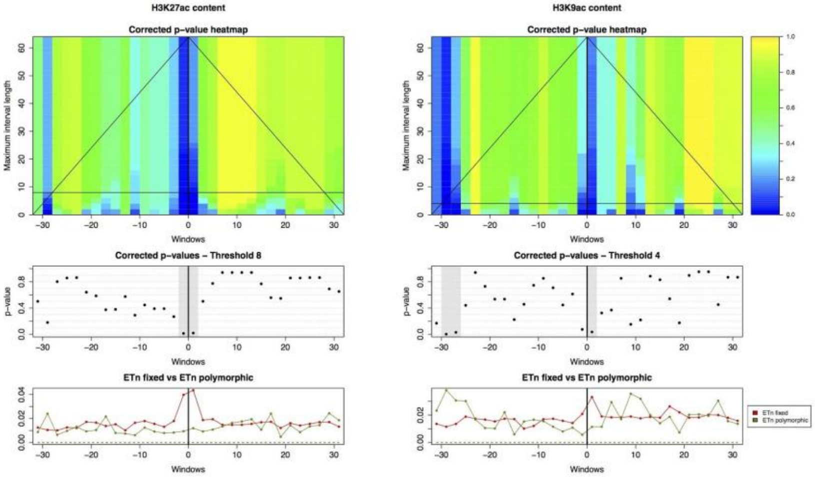
H3K27ac content
H3K9ac content
Corrected p-value heatmap
Maximum interval length
Windows
Corrected p-values - Threshold 8
Corrected p-values - Threshold 4
p-value
ETn fixed vs ETn polymorphic
ETn fixed
ETn polymorphic

B)

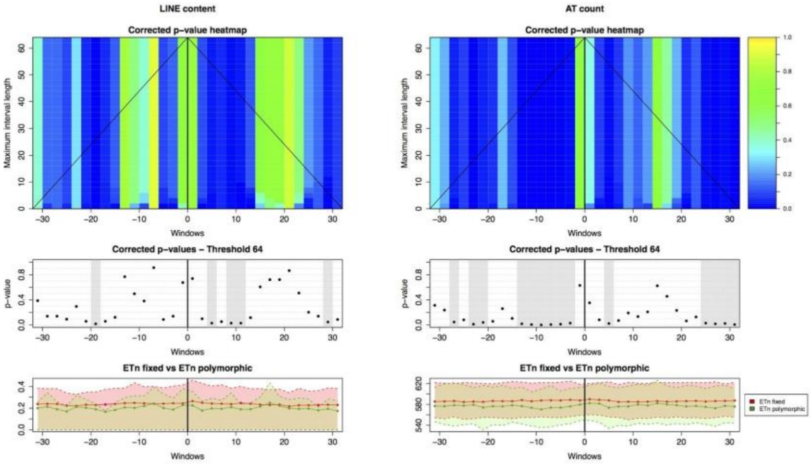
LINE content
AT count
Corrected p-value heatmap
Maximum interval length
Windows
Corrected p-values - Threshold 64
p-value
ETn fixed vs ETn polymorphic
ETn fixed
ETn polymorphic

C)

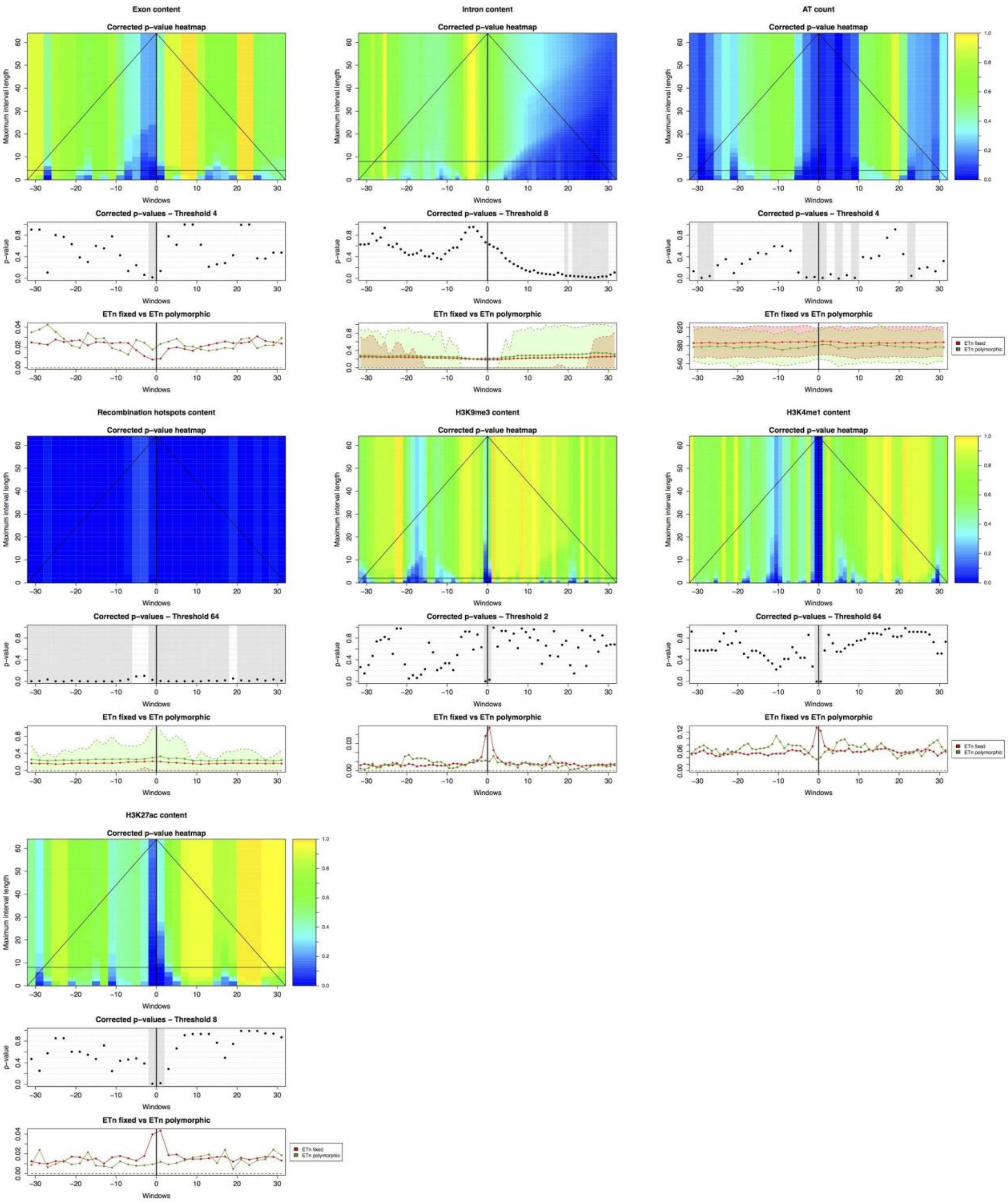
Exon content
Intron content
AT count
Corrected p−value heatmap
Corrected p−values – Threshold 4
Corrected p−values – Threshold 8
ETn fixed vs ETn polymorphic
Recombination hotspots content
H3K9me3 content
H3K4me1 content
Corrected p−values – Threshold 64
Corrected p−values – Threshold 2
H3K27ac content
Maximum interval length
Windows
p−value
ETn fixed
ETn polymorphic

Figure I. Significant genomic features for ITP of comparisons fixed IAP vs. control with test statistics: A) sample mean difference, B) sample median difference, C) sample variance ratio.

A)

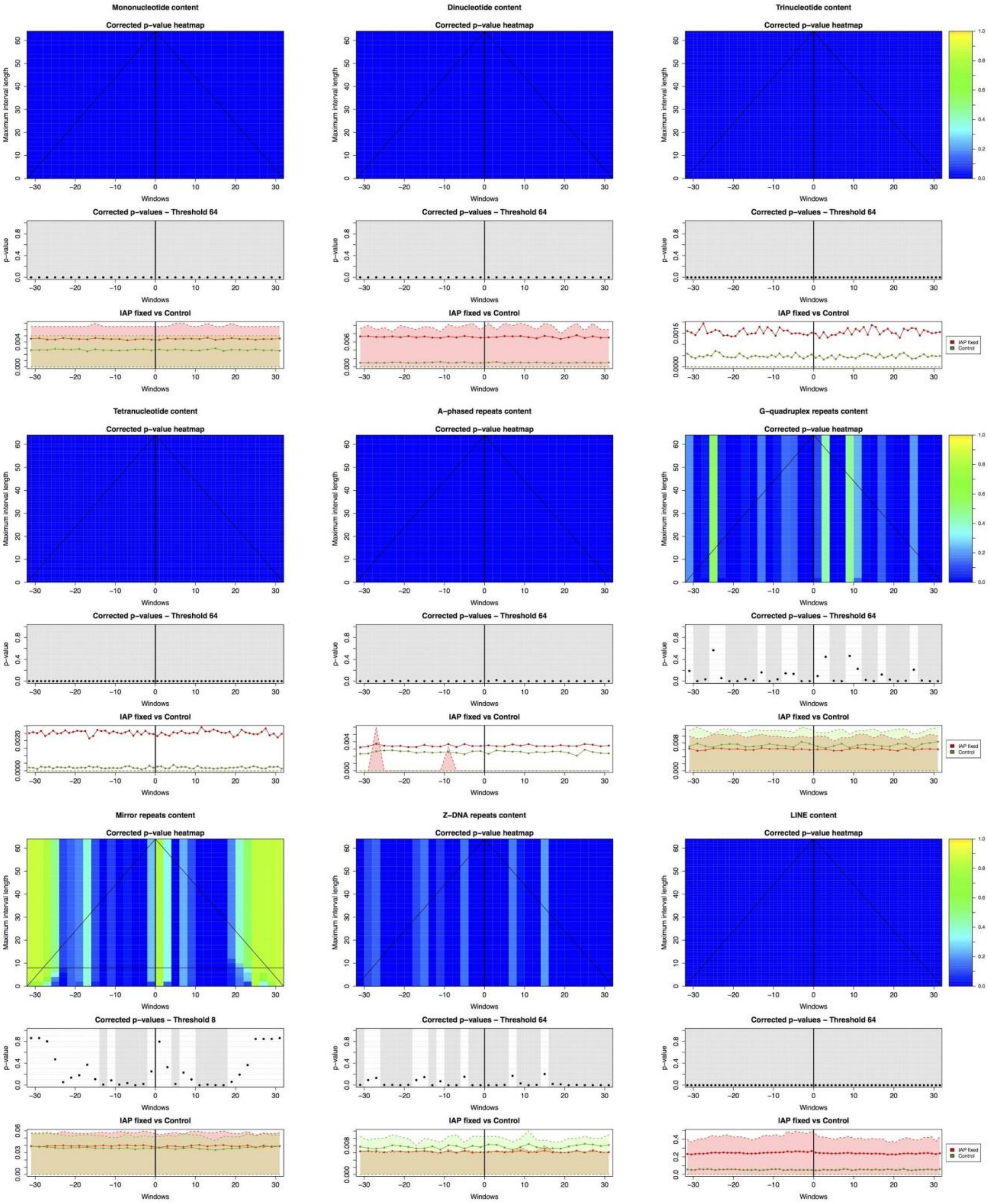

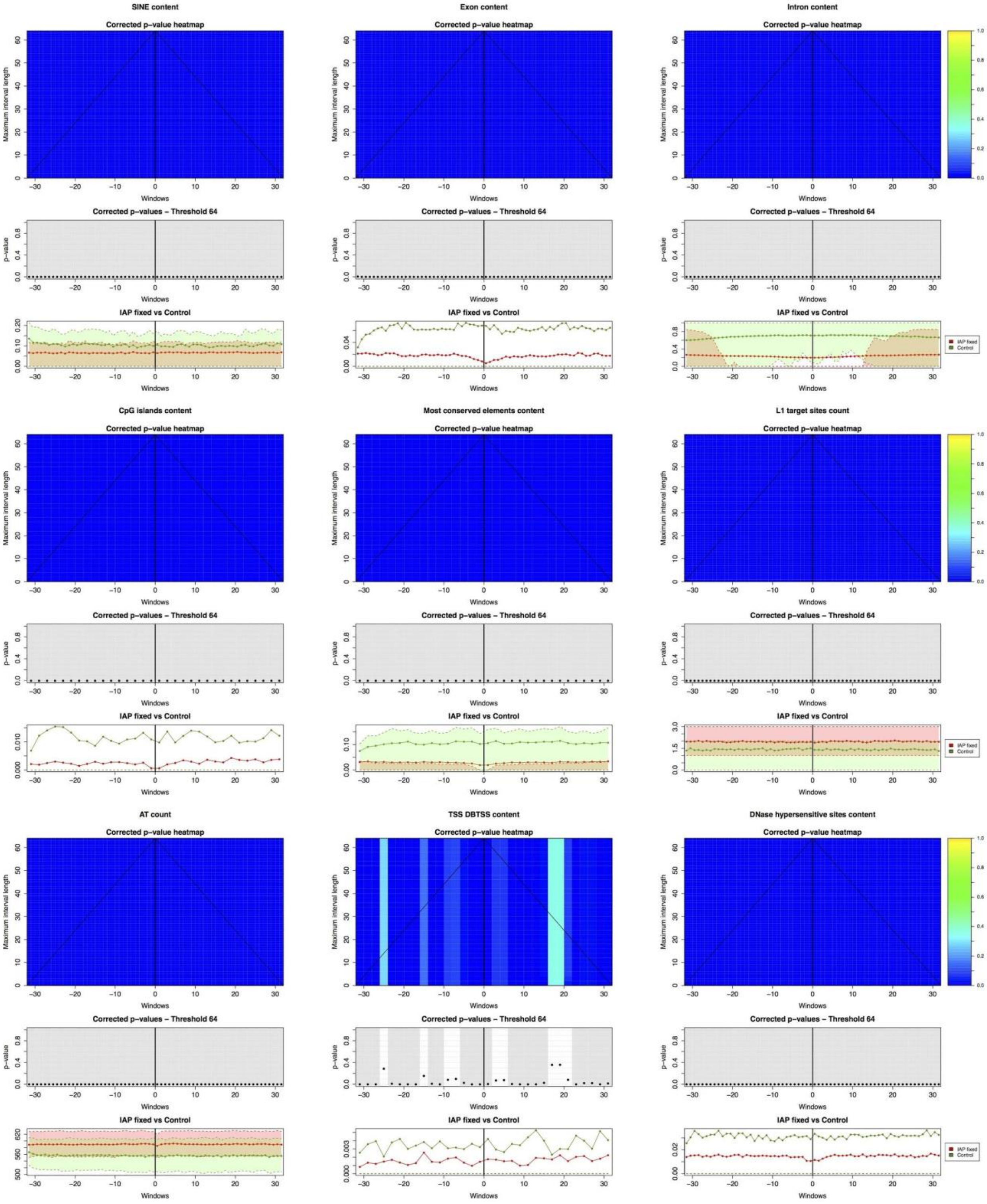
SINE content
Exon content
Intron content
CpG islands content
Most conserved elements content
L1 target sites count
AT count
TSS DBTSS content
DNase hypersensitive sites content
Corrected p−value heatmap
Maximum interval length
Windows
Corrected p−values − Threshold 64
p−value
IAP fixed vs Control
IAP fixed
Control

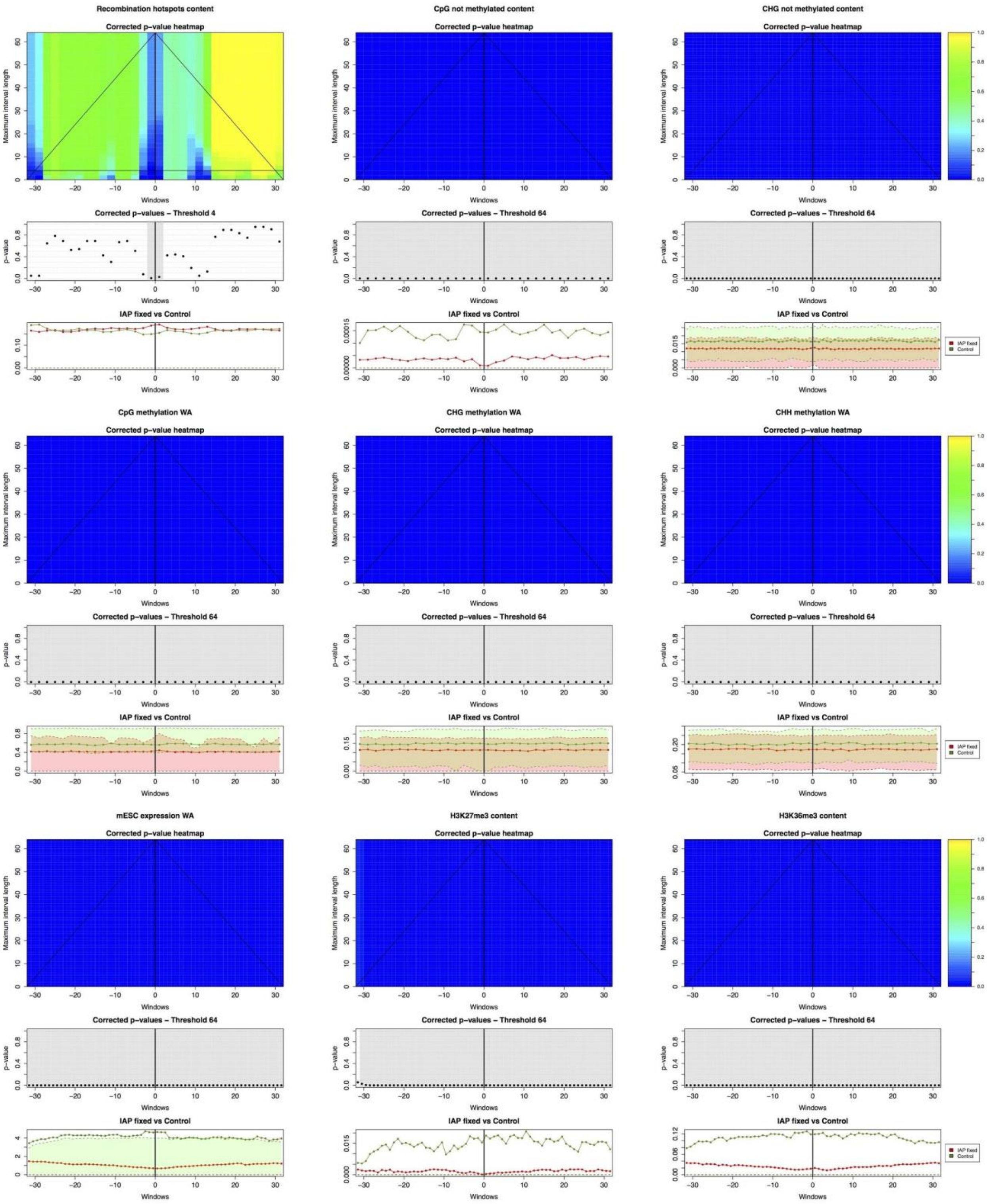
Recombination hotspots content
CpG not methylated content
CHG not methylated content
CpG methylation WA
CHG methylation WA
CHH methylation WA
mESC expression WA
H3K27me3 content
H3K36me3 content
Corrected p−value heatmap
Maximum interval length
Windows
Corrected p−values − Threshold 4
Corrected p−values − Threshold 64
p−value
IAP fixed vs Control
IAP fixed
Control

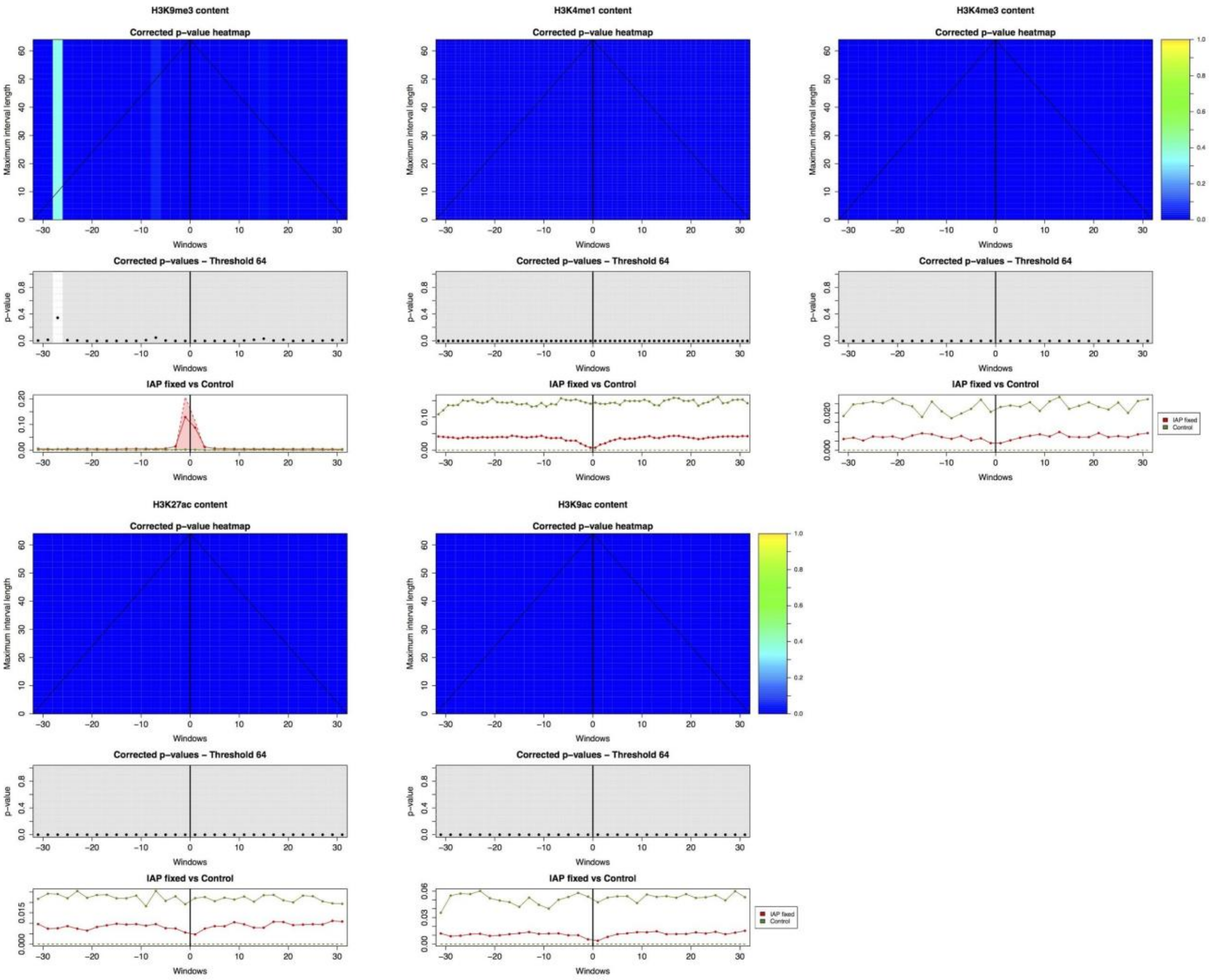

H3K9me3 content
Corrected p−value heatmap
Maximum interval length
Windows
Corrected p−values − Threshold 64
p−value
IAP fixed vs Control
H3K4me1 content
H3K4me3 content
IAP fixed
Control
H3K27ac content
H3K9ac content

B)

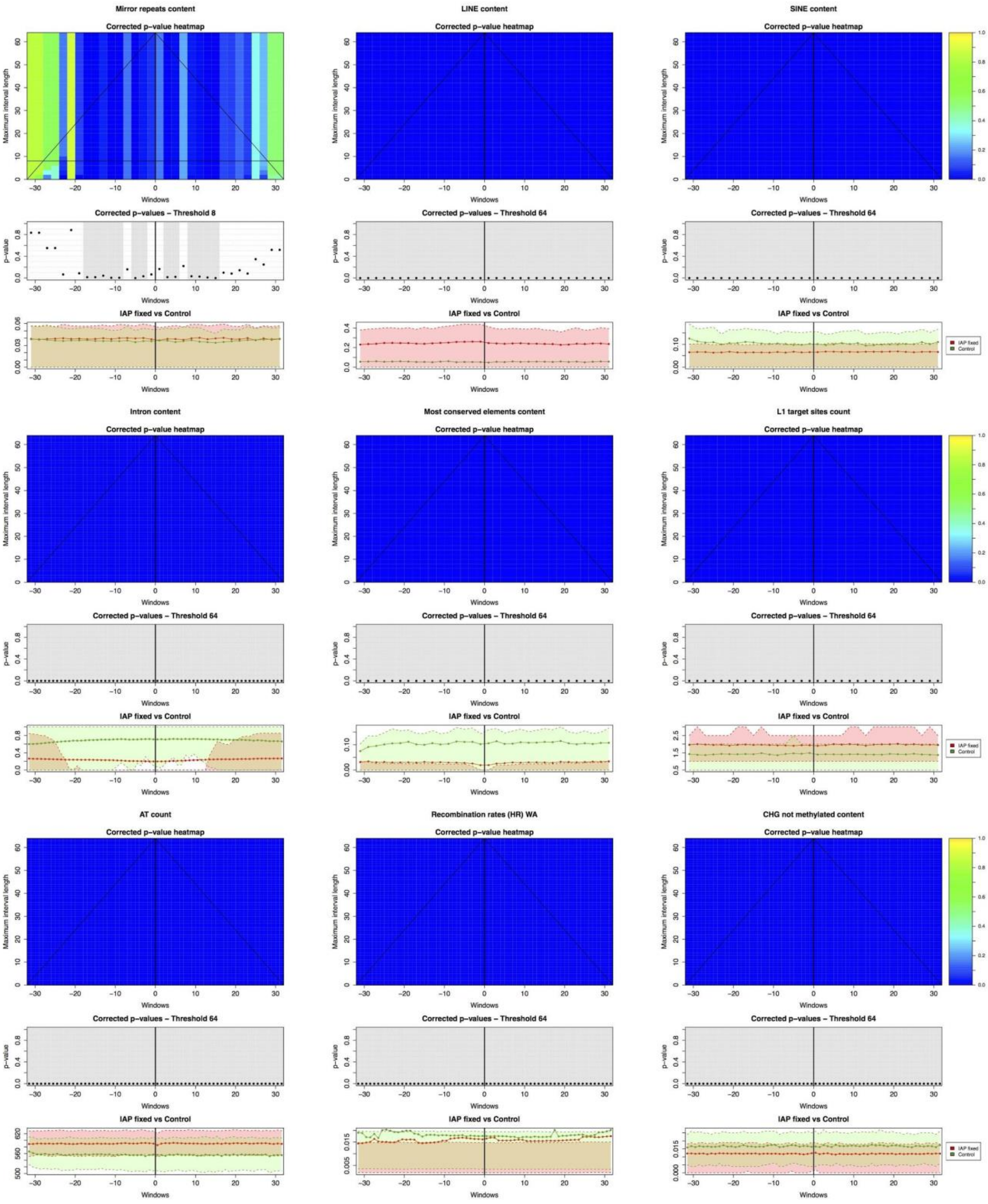
Mirror repeats content
LINE content
SINE content
Corrected p−value heatmap
Maximum interval length
Windows
Corrected p−values – Threshold 8
Corrected p−values – Threshold 64
p−value
IAP fixed vs Control
IAP fixed
Control
Intron content
Most conserved elements content
L1 target sites count
AT count
Recombination rates (HR) WA
CHG not methylated content

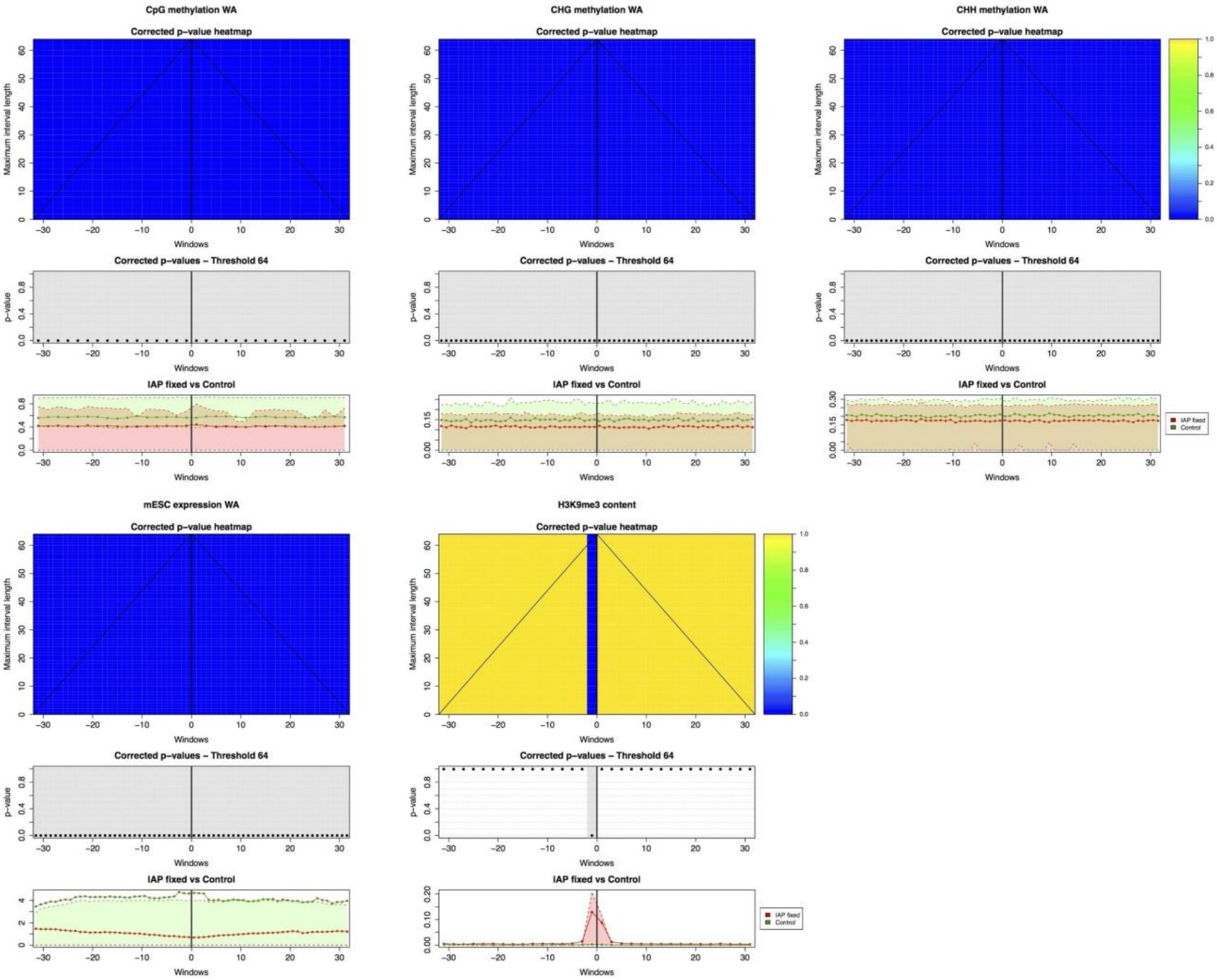
CpG methylation WA
CHG methylation WA
CHH methylation WA
Corrected p−value heatmap
Maximum interval length
Windows
Corrected p−values − Threshold 64
p−value
IAP fixed vs Control
IAP fixed
Control
mESC expression WA
H3K9me3 content

C)

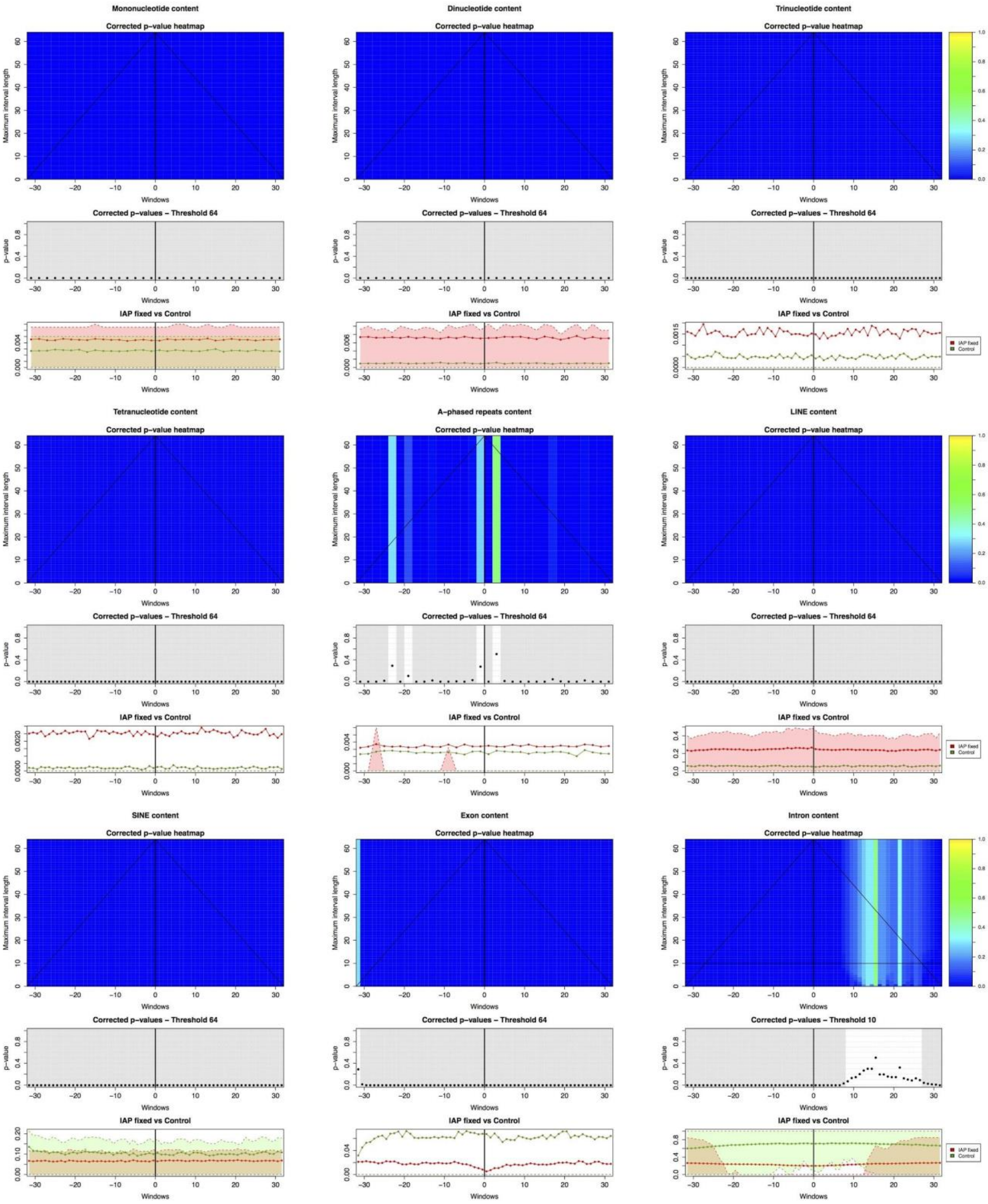

Mononucleotide content
Dinucleotide content
Trinucleotide content
Tetranucleotide content
A–phased repeats content
LINE content
SINE content
Exon content
Intron content
Corrected p–value heatmap
Maximum interval length
Windows
Corrected p–values – Threshold 64
Corrected p–values – Threshold 10
p–value
IAP fixed vs Control
IAP fixed
Control

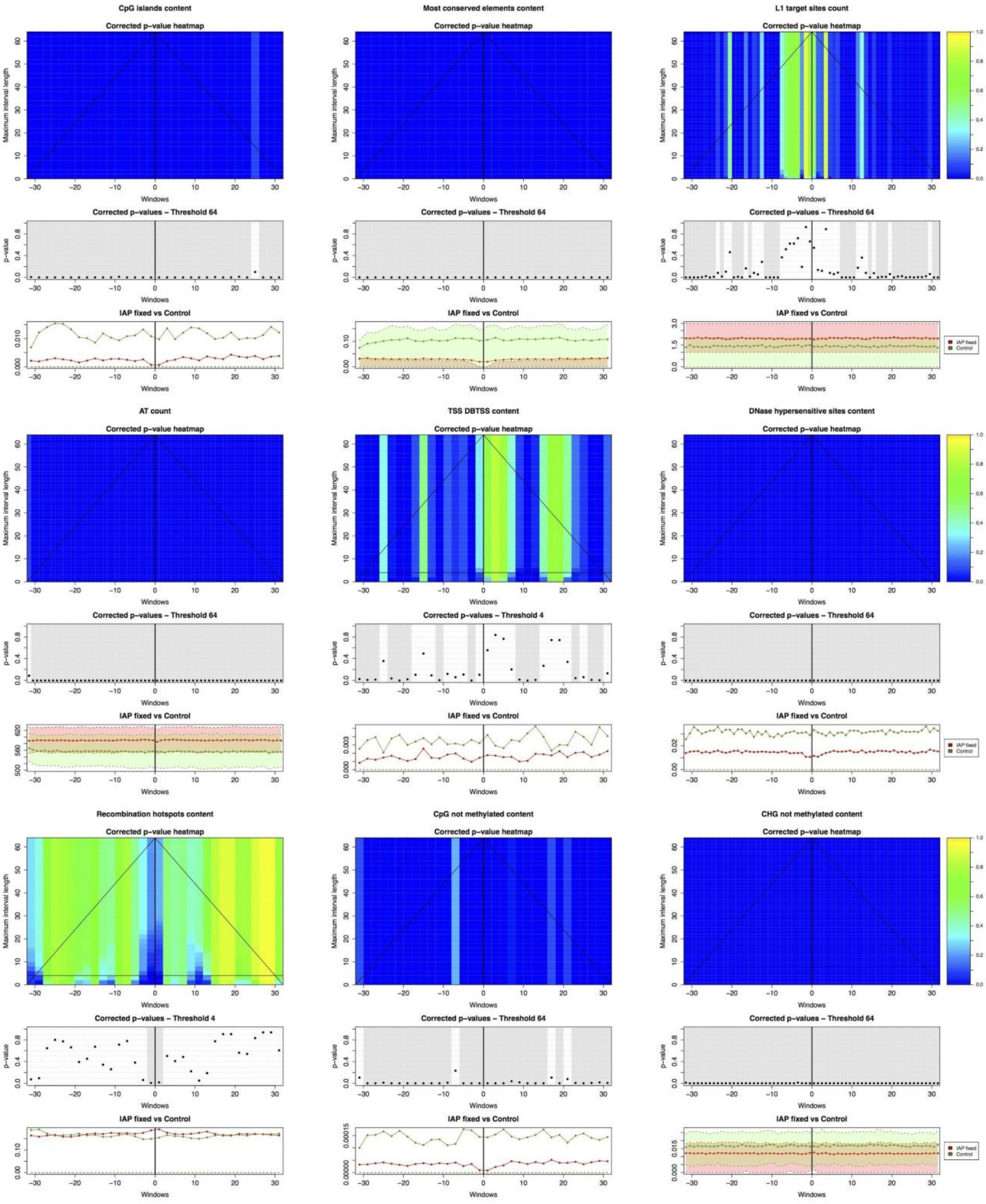
CpG islands content
Most conserved elements content
L1 target sites count
AT count
TSS DBTSS content
DNase hypersensitive sites content
Recombination hotspots content
CpG not methylated content
CHG not methylated content
Corrected p–value heatmap
Maximum interval length
Windows
Corrected p–values – Threshold 64
Corrected p–values – Threshold 4
p–value
IAP fixed vs Control
IAP fixed
Control

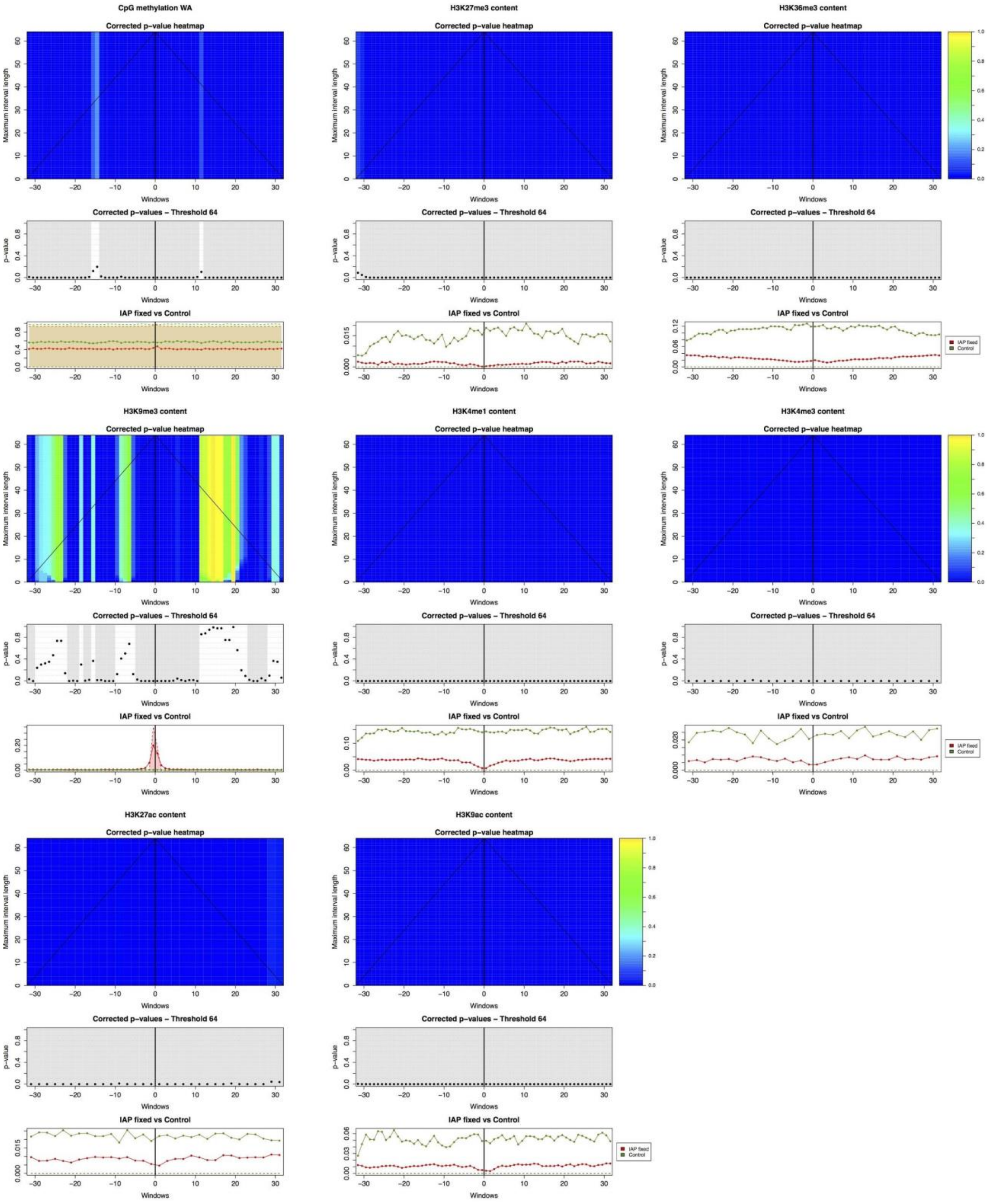
CpG methylation WA
Corrected p−value heatmap
Maximum interval length
Windows
Corrected p−values − Threshold 64
p−value
IAP fixed vs Control
H3K27me3 content
H3K36me3 content
IAP fixed
Control
H3K9me3 content
H3K4me1 content
H3K4me3 content
H3K27ac content
H3K9ac content

Figure J. Significant genomic features for ITP of comparisons polymorphic IAP vs. control with test statistics: A) sample mean difference, B) sample median difference, C) sample variance ratio.

A)

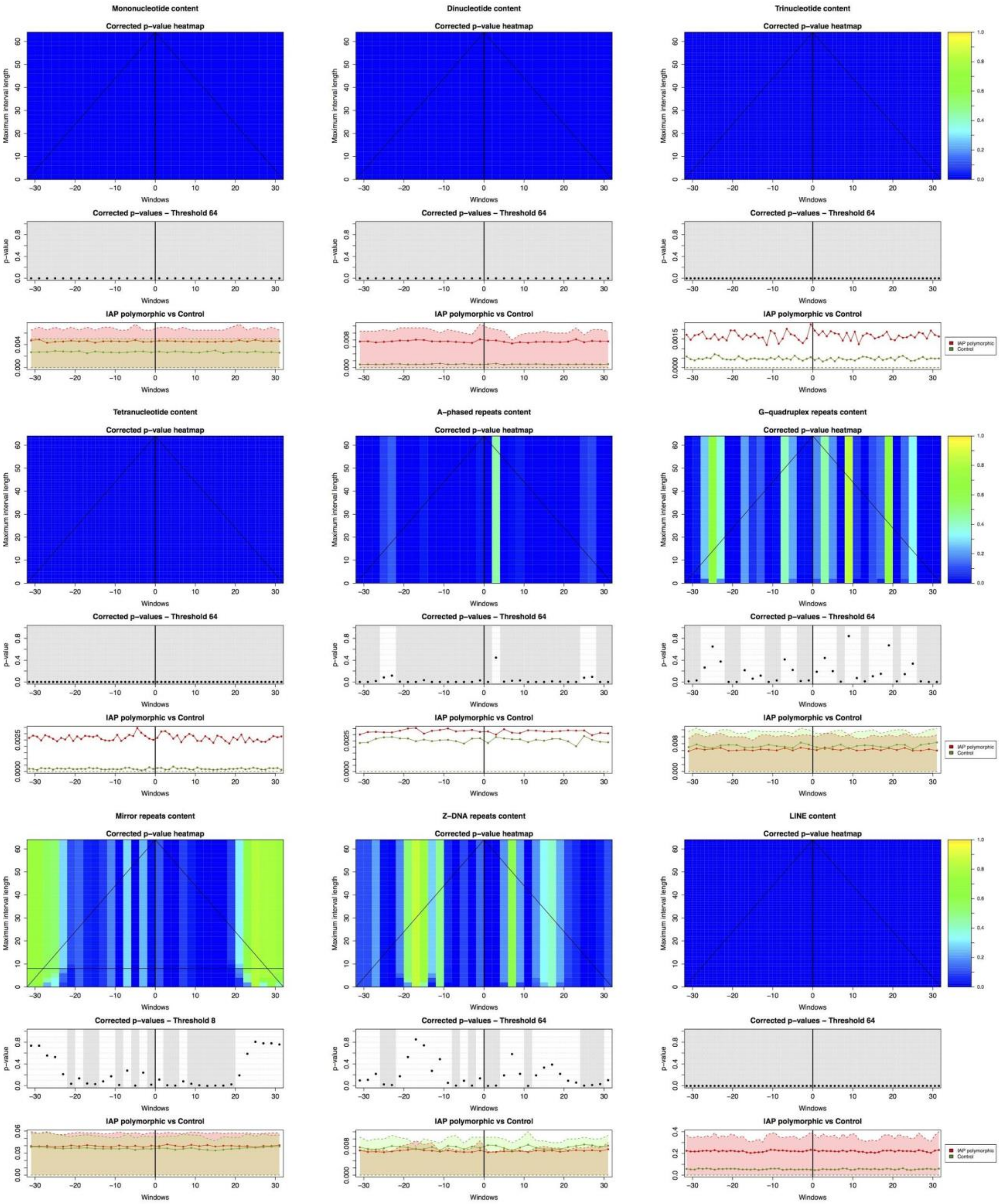

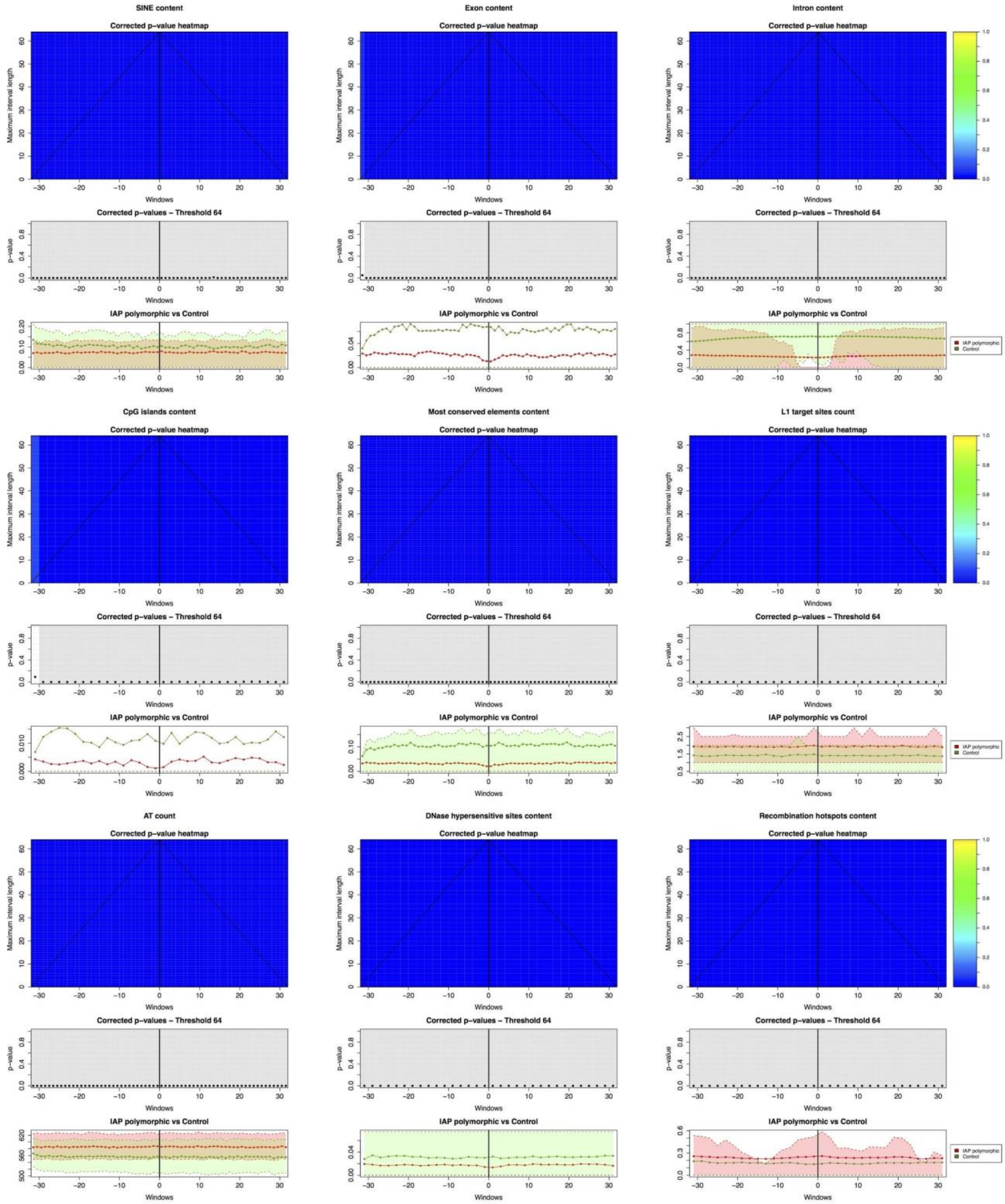
SINE content
Exon content
Intron content
CpG islands content
Most conserved elements content
L1 target sites count
AT count
DNase hypersensitive sites content
Recombination hotspots content
Corrected p−value heatmap
Corrected p−values − Threshold 64
IAP polymorphic vs Control
Maximum interval length
p−value
Windows
IAP polymorphic
Control

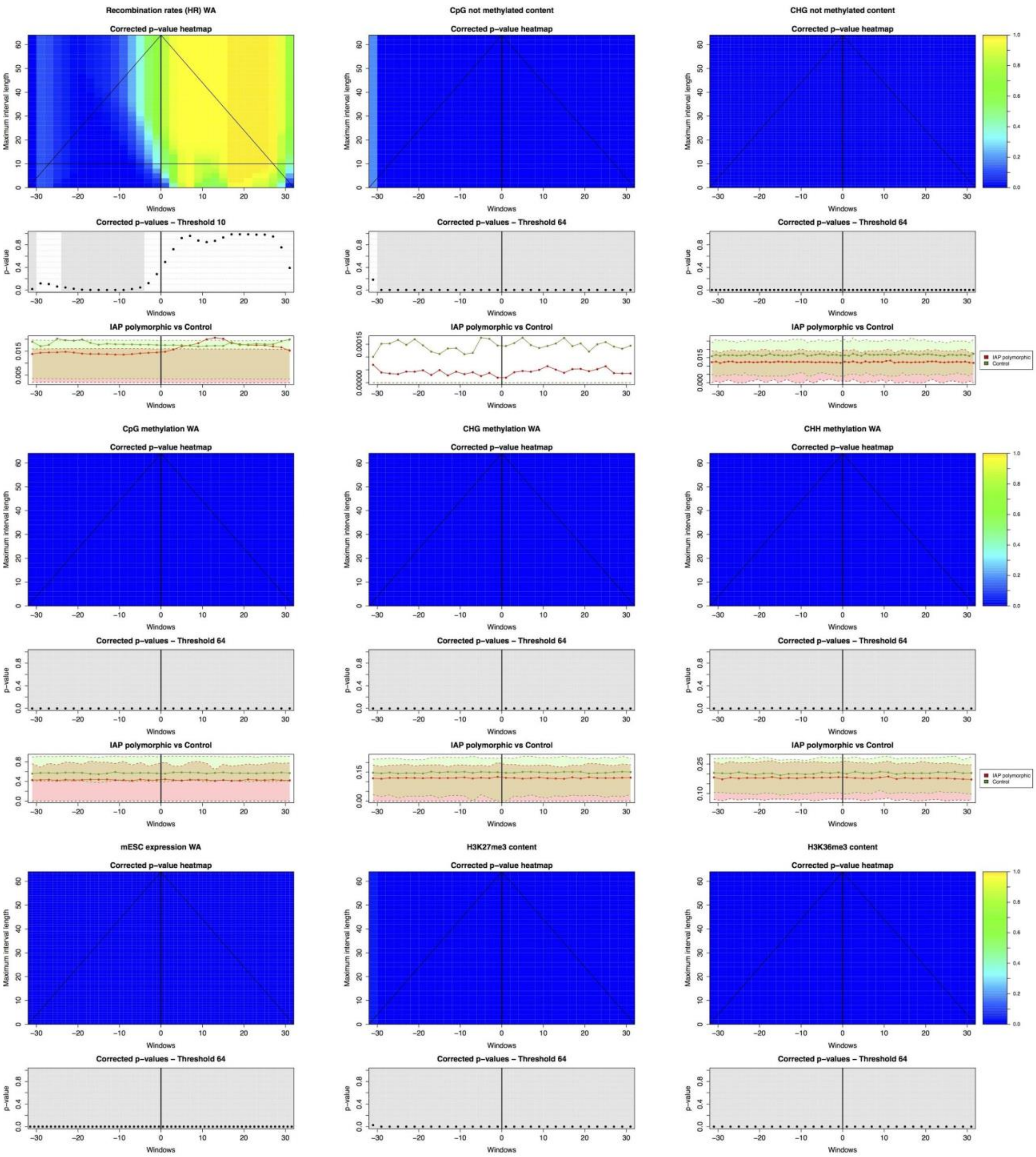

Recombination rates (HR) WA
CpG not methylated content
CHG not methylated content
Corrected p−value heatmap
Maximum interval length
Windows
Corrected p−values − Threshold 10
Corrected p−values − Threshold 64
p−value
IAP polymorphic vs Control
IAP polymorphic
Control
CpG methylation WA
CHG methylation WA
CHH methylation WA
mESC expression WA
H3K27me3 content
H3K36me3 content

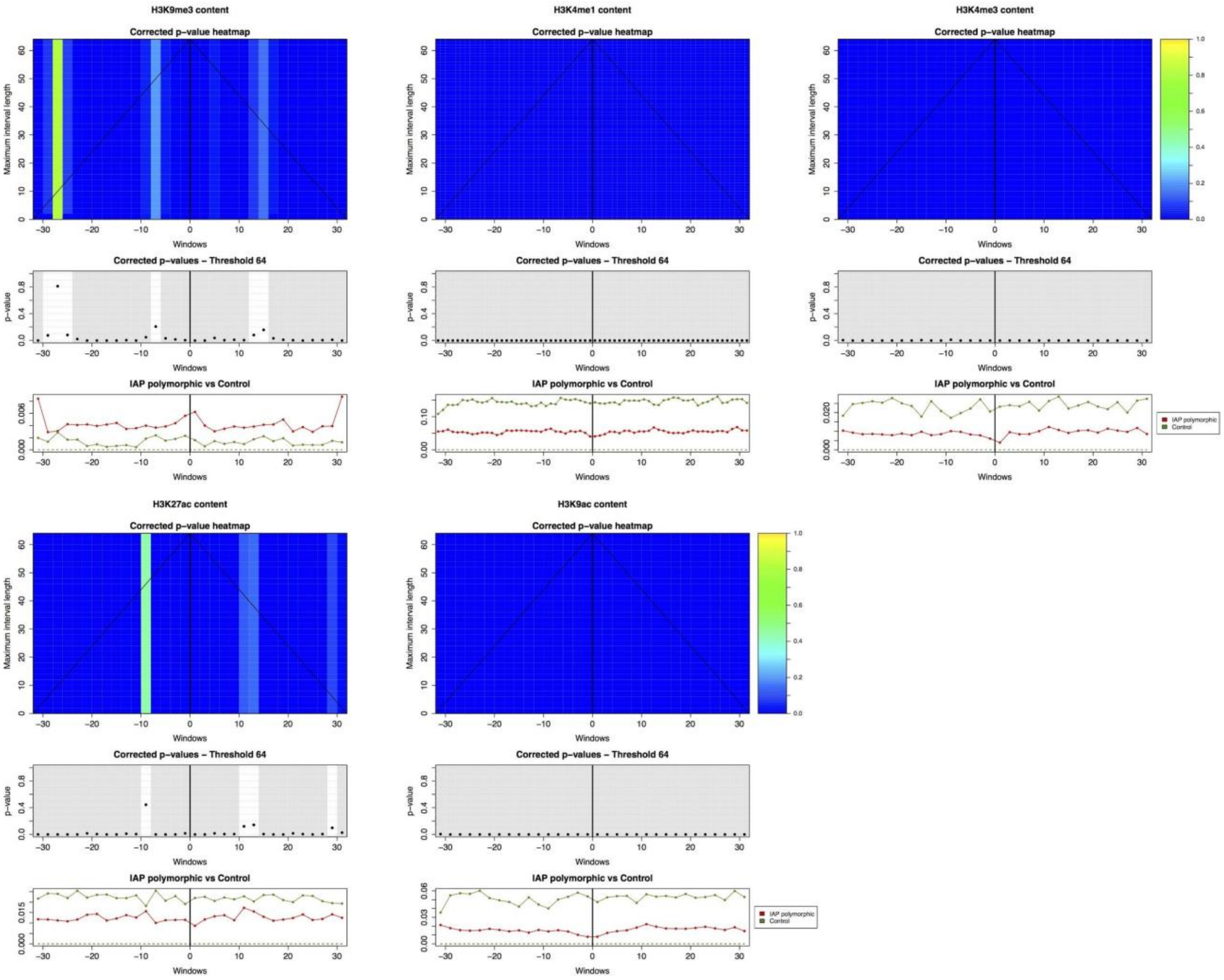

H3K9me3 content
H3K4me1 content
H3K4me3 content
H3K27ac content
H3K9ac content
Corrected p–value heatmap
Maximum interval length
Windows
Corrected p–values – Threshold 64
p–value
IAP polymorphic vs Control
IAP polymorphic
Control

B)

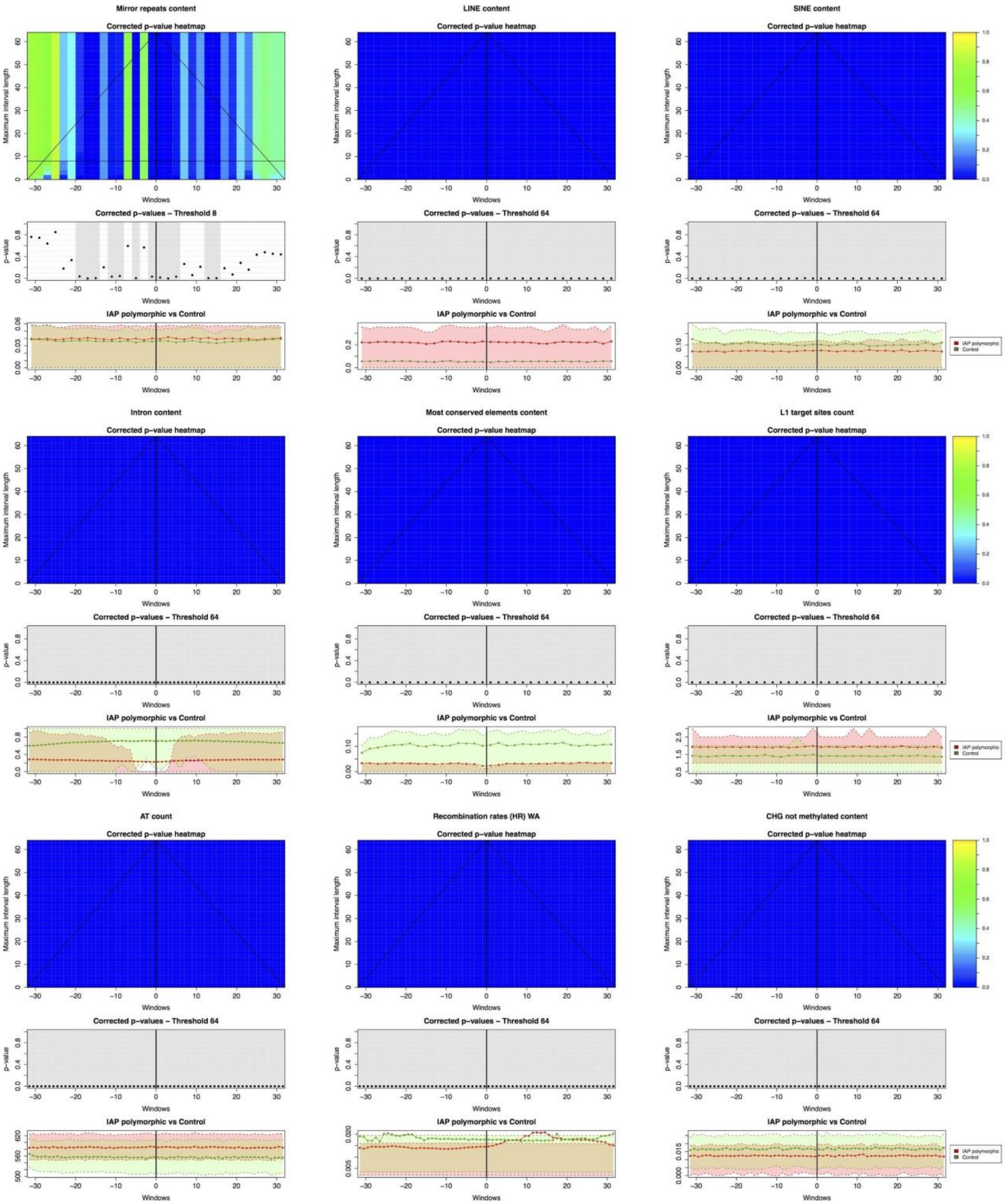
Mirror repeats content
LINE content
SINE content
Corrected p−value heatmap
Maximum interval length
Windows
Corrected p−values − Threshold 8
Corrected p−values − Threshold 64
p−value
IAP polymorphic vs Control
IAP polymorphic
Control
Intron content
Most conserved elements content
L1 target sites count
AT count
Recombination rates (HR) WA
CHG not methylated content

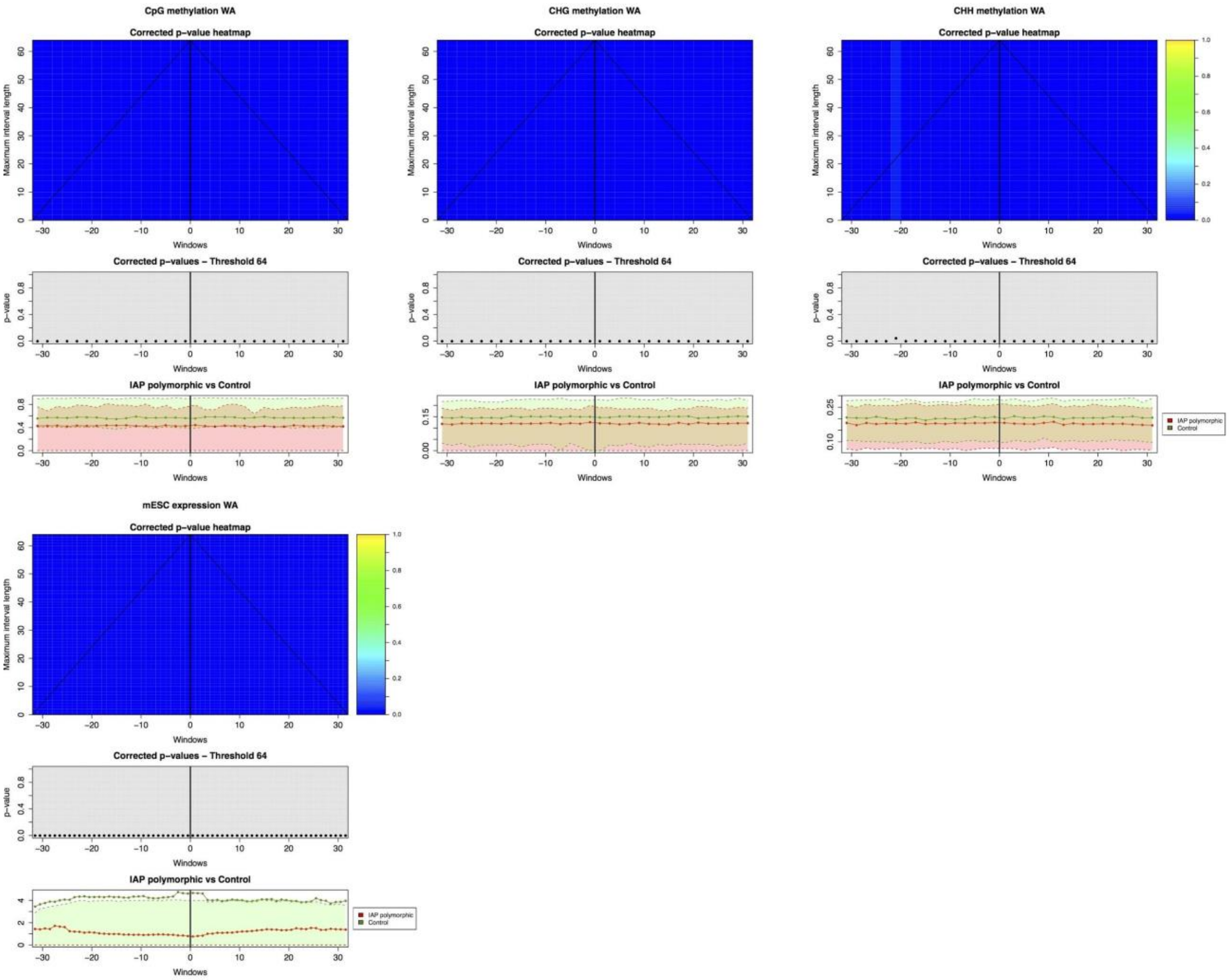
CpG methylation WA
CHG methylation WA
CHH methylation WA
mESC expression WA
Corrected p−value heatmap
Corrected p−values − Threshold 64
IAP polymorphic vs Control
Maximum interval length
p−value
Windows
IAP polymorphic
Control

C)

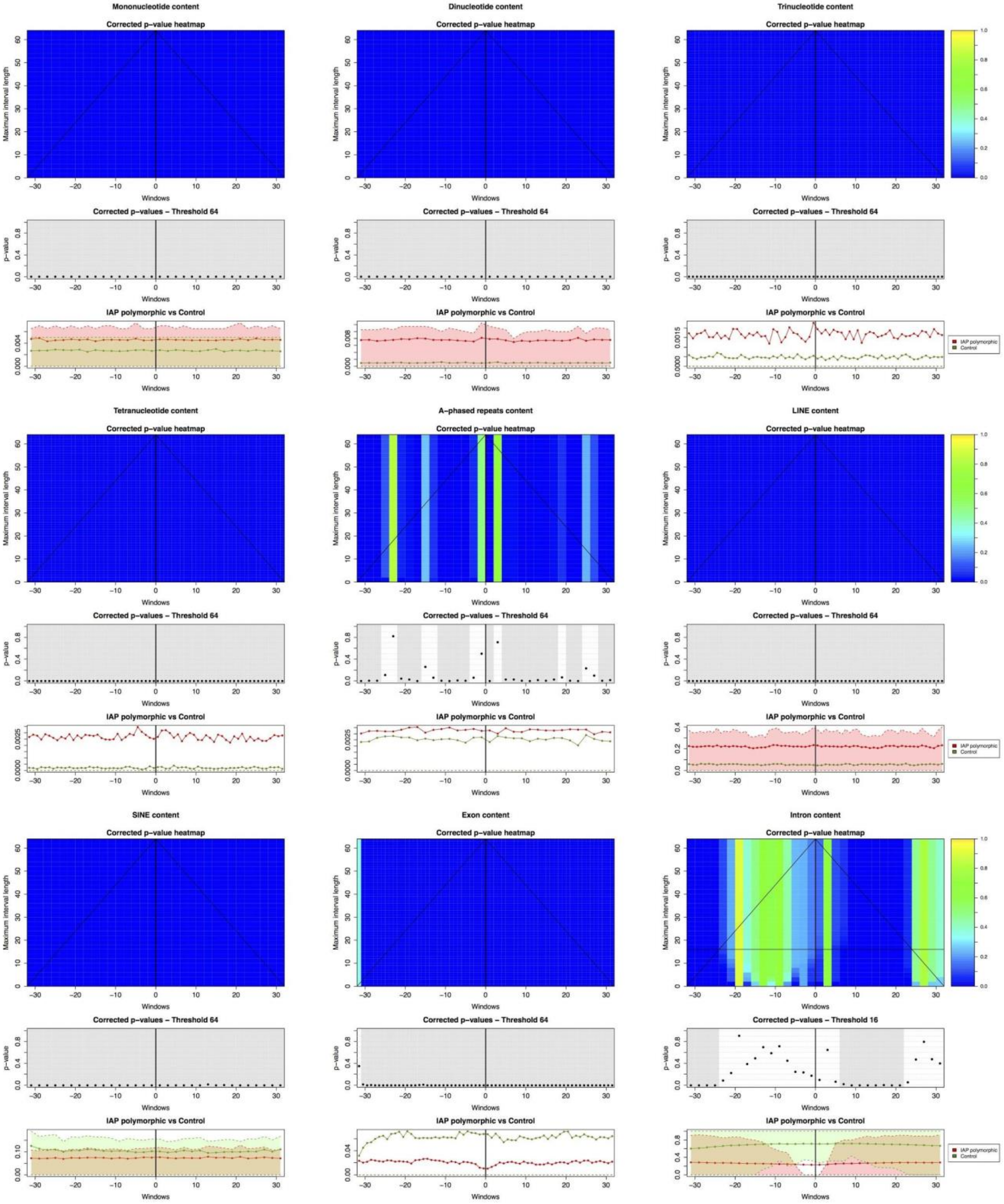
Mononucleotide content
Dinucleotide content
Trinucleotide content
Tetranucleotide content
A–phased repeats content
LINE content
SINE content
Exon content
Intron content
Corrected p–value heatmap
Maximum interval length
Windows
Corrected p–values – Threshold 64
Corrected p–values – Threshold 16
p–value
IAP polymorphic vs Control
IAP polymorphic
Control

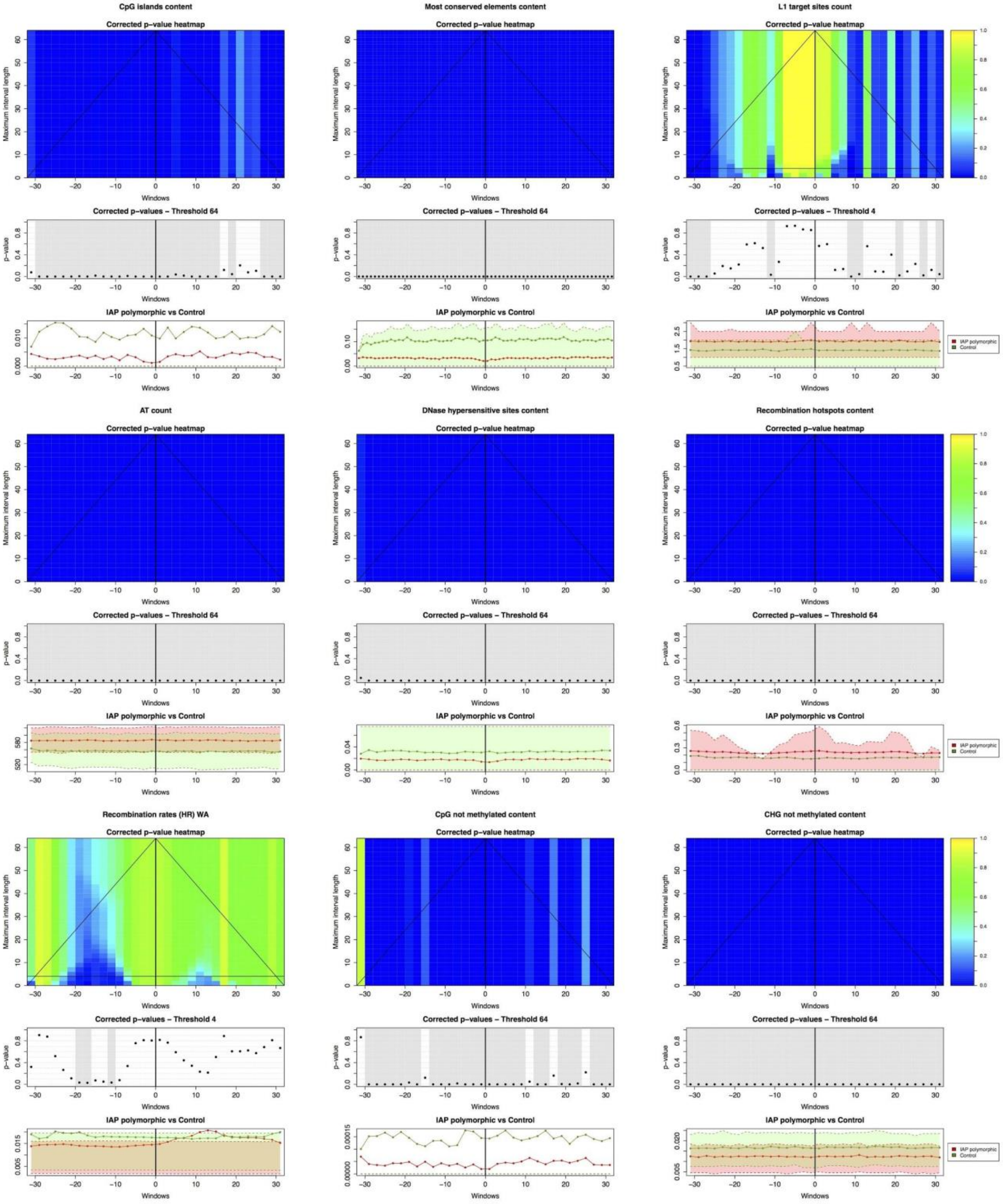
CpG islands content
Most conserved elements content
L1 target sites count
AT count
DNase hypersensitive sites content
Recombination hotspots content
Recombination rates (HR) WA
CpG not methylated content
CHG not methylated content
Corrected p−value heatmap
Maximum interval length
Windows
Corrected p−values − Threshold 64
Corrected p−values − Threshold 4
p−value
IAP polymorphic vs Control
IAP polymorphic
Control

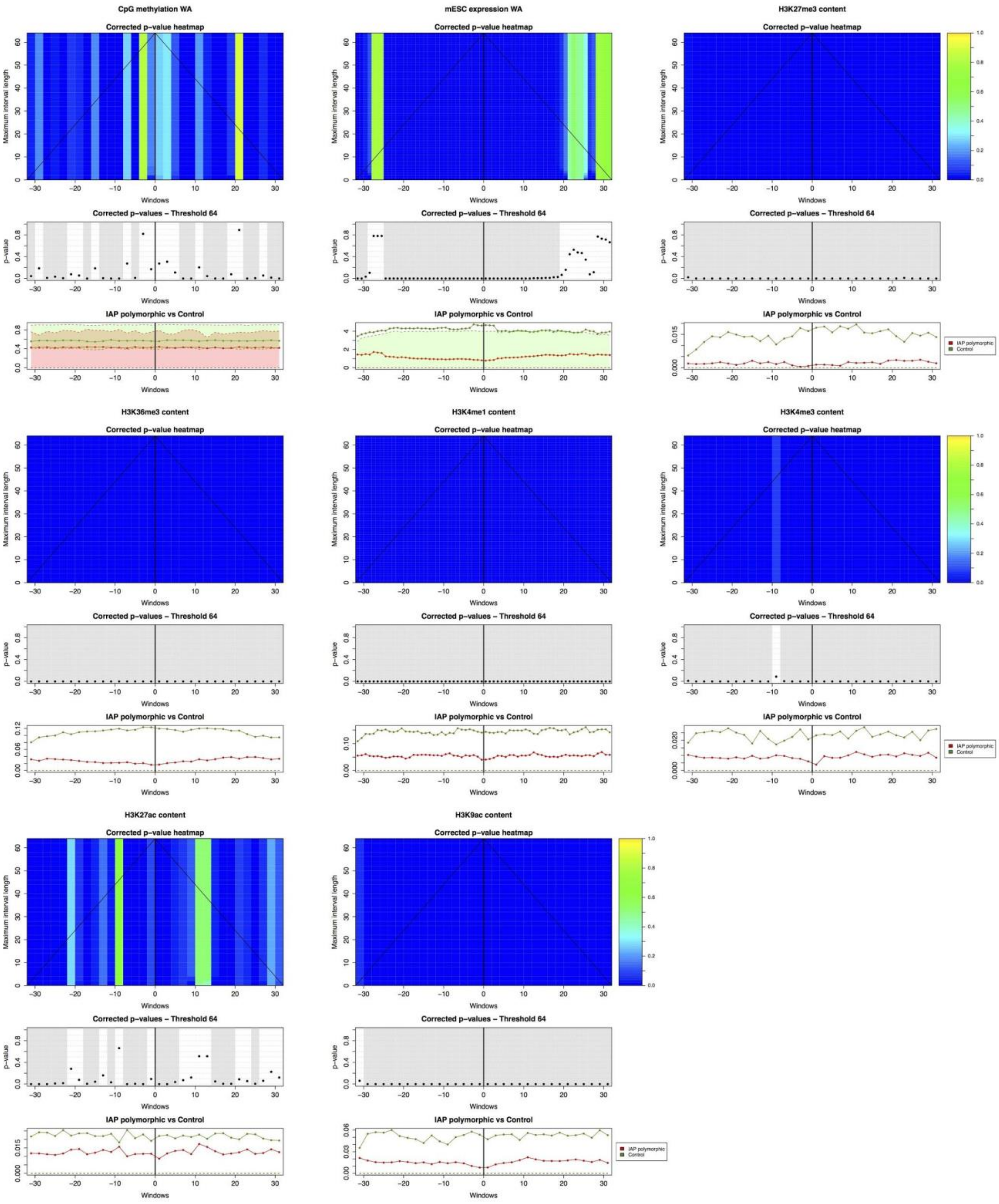
CpG methylation WA
mESC expression WA
H3K27me3 content
H3K36me3 content
H3K4me1 content
H3K4me3 content
H3K27ac content
H3K9ac content
Corrected p−value heatmap
Maximum interval length
Windows
Corrected p−values − Threshold 64
p−value
IAP polymorphic vs Control
IAP polymorphic
Control

Figure K. Significant genomic features for ITP of comparisons fixed vs. polymorphic IAP with test statistics: A) sample mean difference, B) sample median difference, C) sample variance ratio.

A)

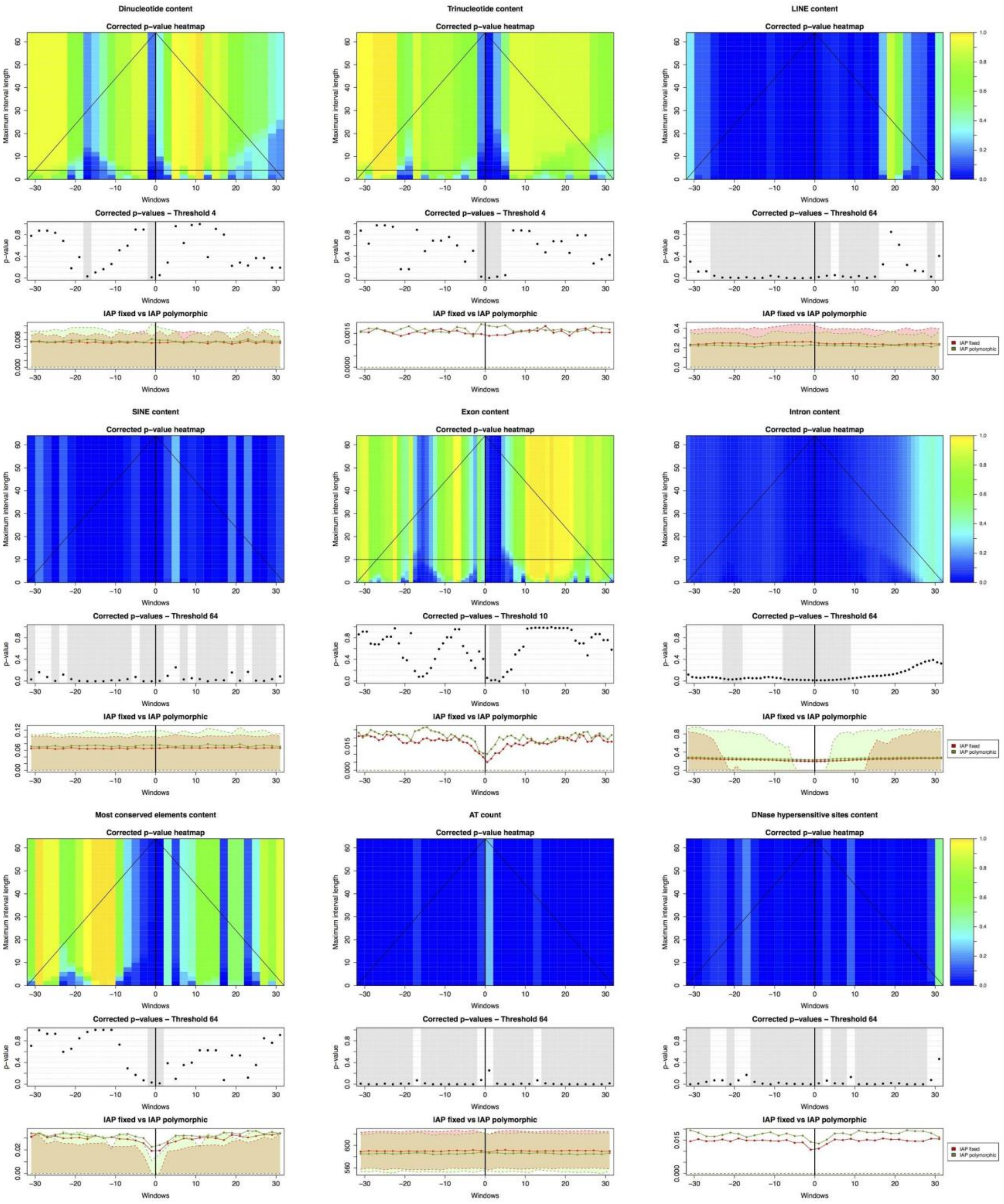

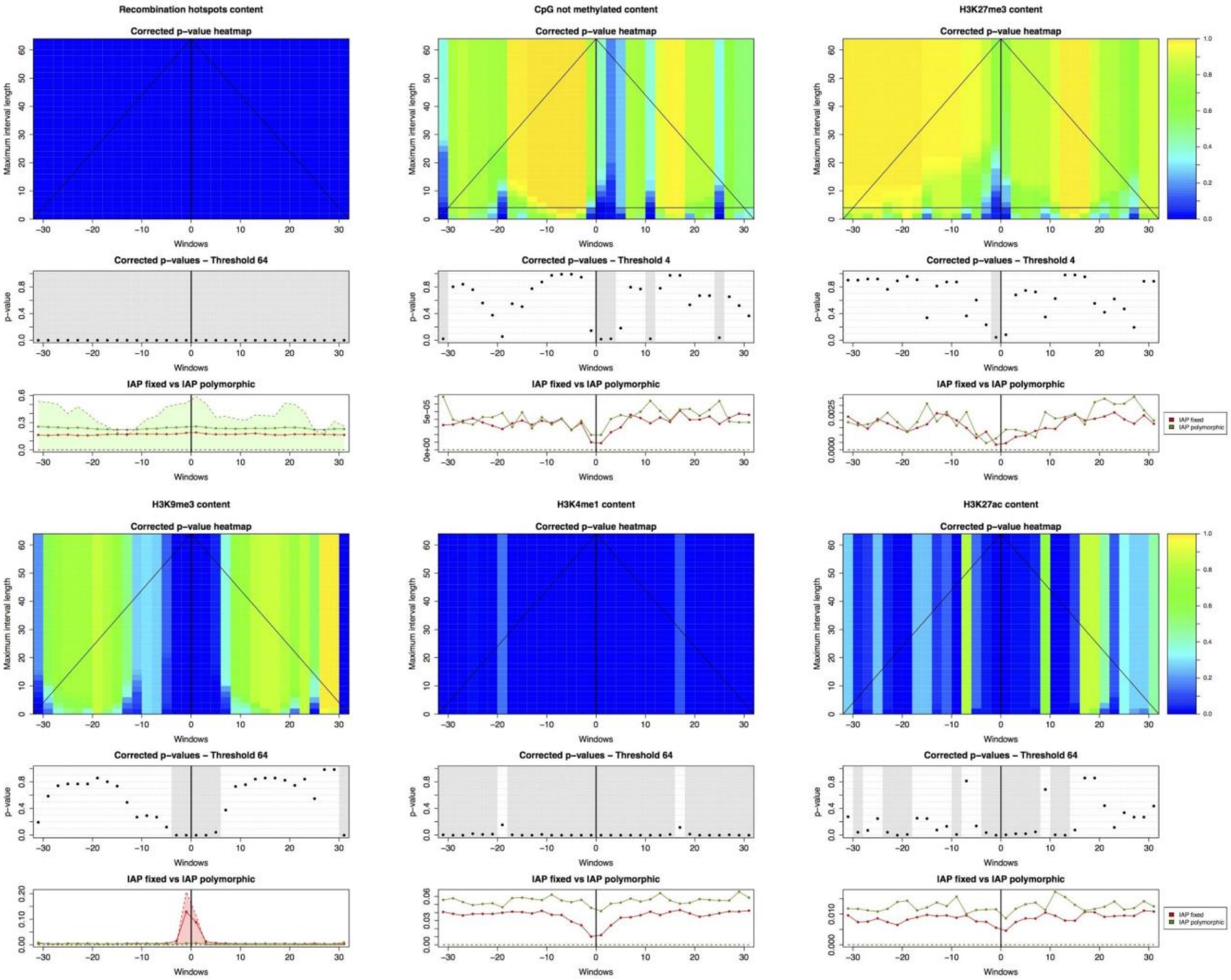
Recombination hotspots content
Corrected p−value heatmap
Maximum interval length
Windows
Corrected p−values − Threshold 64
p−value
IAP fixed vs IAP polymorphic
CpG not methylated content
Corrected p−value heatmap
Corrected p−values − Threshold 4
IAP fixed vs IAP polymorphic
H3K27me3 content
Corrected p−value heatmap
Corrected p−values − Threshold 4
IAP fixed vs IAP polymorphic
IAP fixed
IAP polymorphic
H3K9me3 content
Corrected p−value heatmap
Corrected p−values − Threshold 64
IAP fixed vs IAP polymorphic
H3K4me1 content
Corrected p−value heatmap
Corrected p−values − Threshold 64
IAP fixed vs IAP polymorphic
H3K27ac content
Corrected p−value heatmap
Corrected p−values − Threshold 64
IAP fixed vs IAP polymorphic
IAP fixed
IAP polymorphic

B)

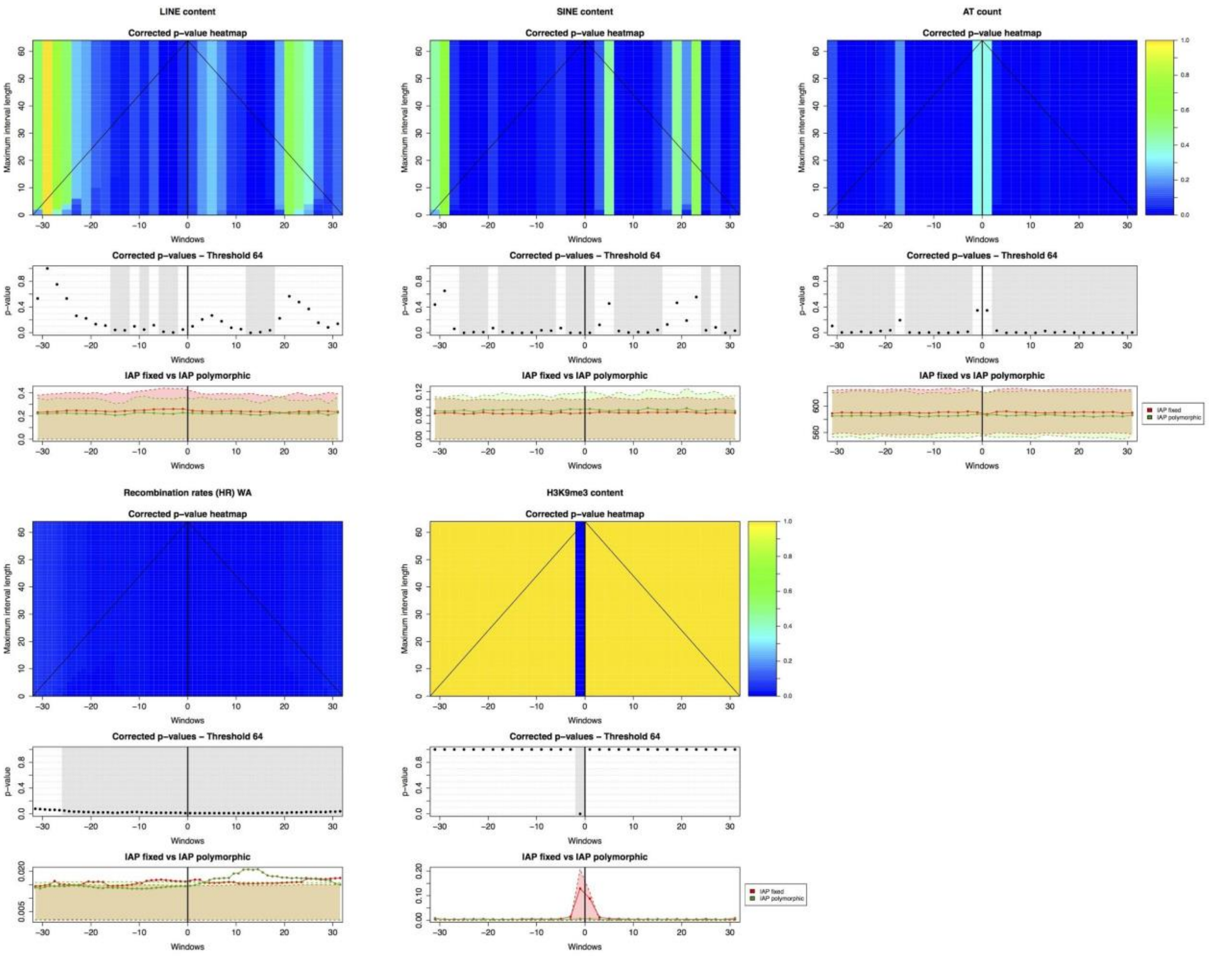
LINE content
SINE content
AT count
Corrected p–value heatmap
Maximum interval length
Windows
Corrected p–values – Threshold 64
p–value
IAP fixed vs IAP polymorphic
IAP fixed
IAP polymorphic
Recombination rates (HR) WA
H3K9me3 content

C)

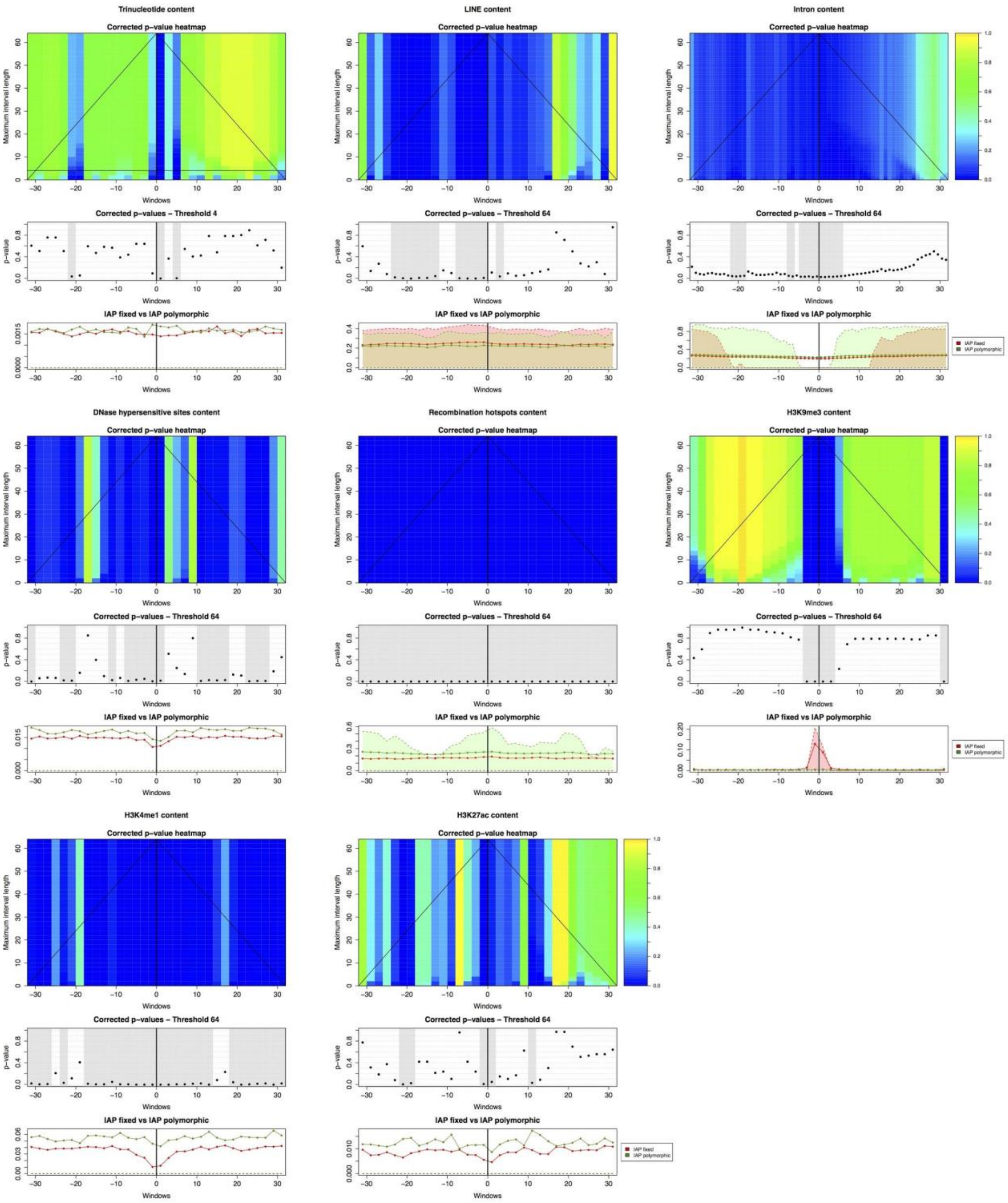
Trinucleotide content
LINE content
Intron content
DNase hypersensitive sites content
Recombination hotspots content
H3K9me3 content
H3K4me1 content
H3K27ac content
Corrected p−value heatmap
Maximum interval length
Windows
Corrected p−values − Threshold 4
Corrected p−values − Threshold 64
p−value
IAP fixed vs IAP polymorphic
IAP fixed
IAP polymorphic

Figure L. Significant genomic features for ITP of comparisons fixed HERV-K vs. control with test statistics: A) sample mean difference, B) sample median difference, C) sample variance ratio.

A)

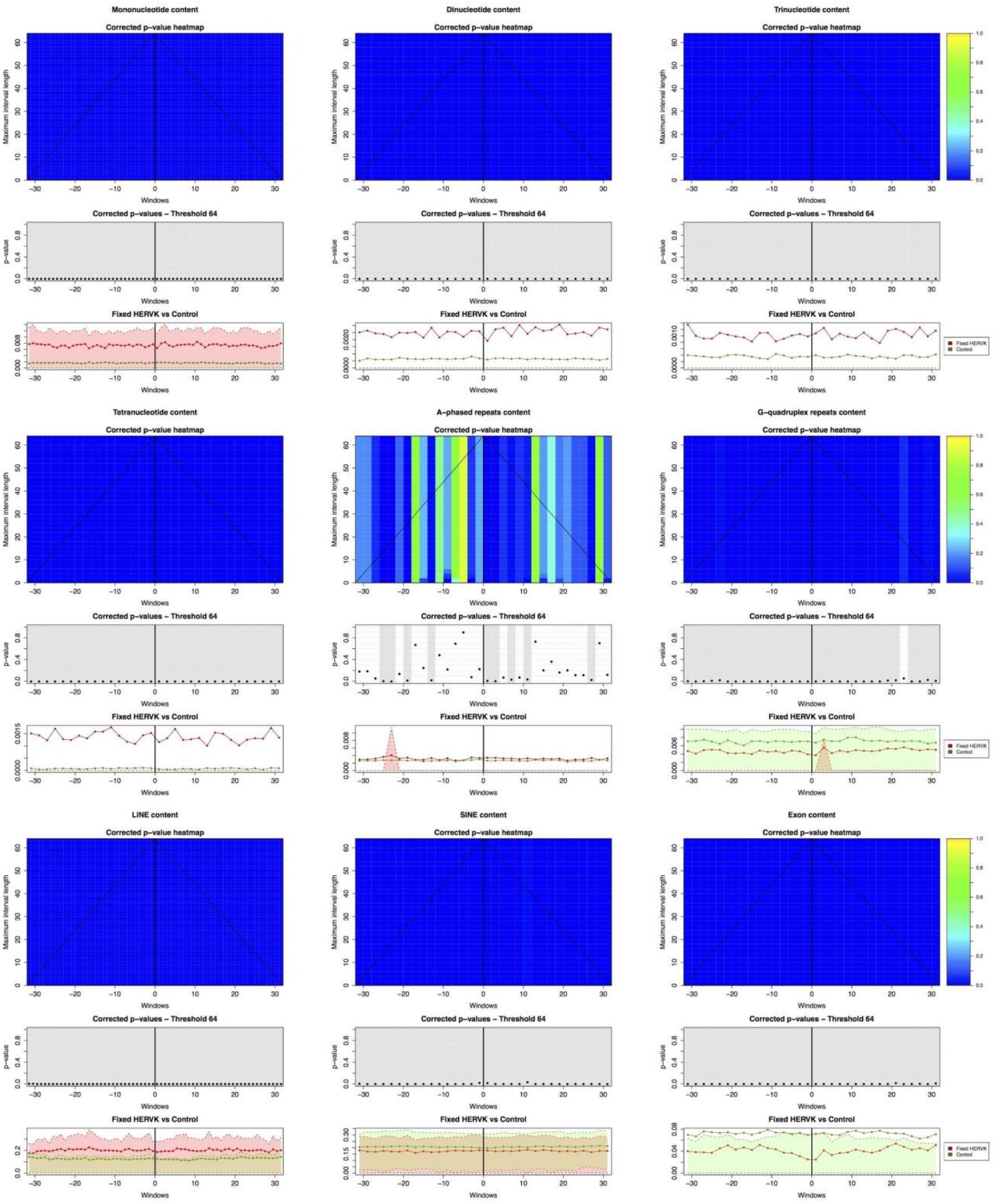

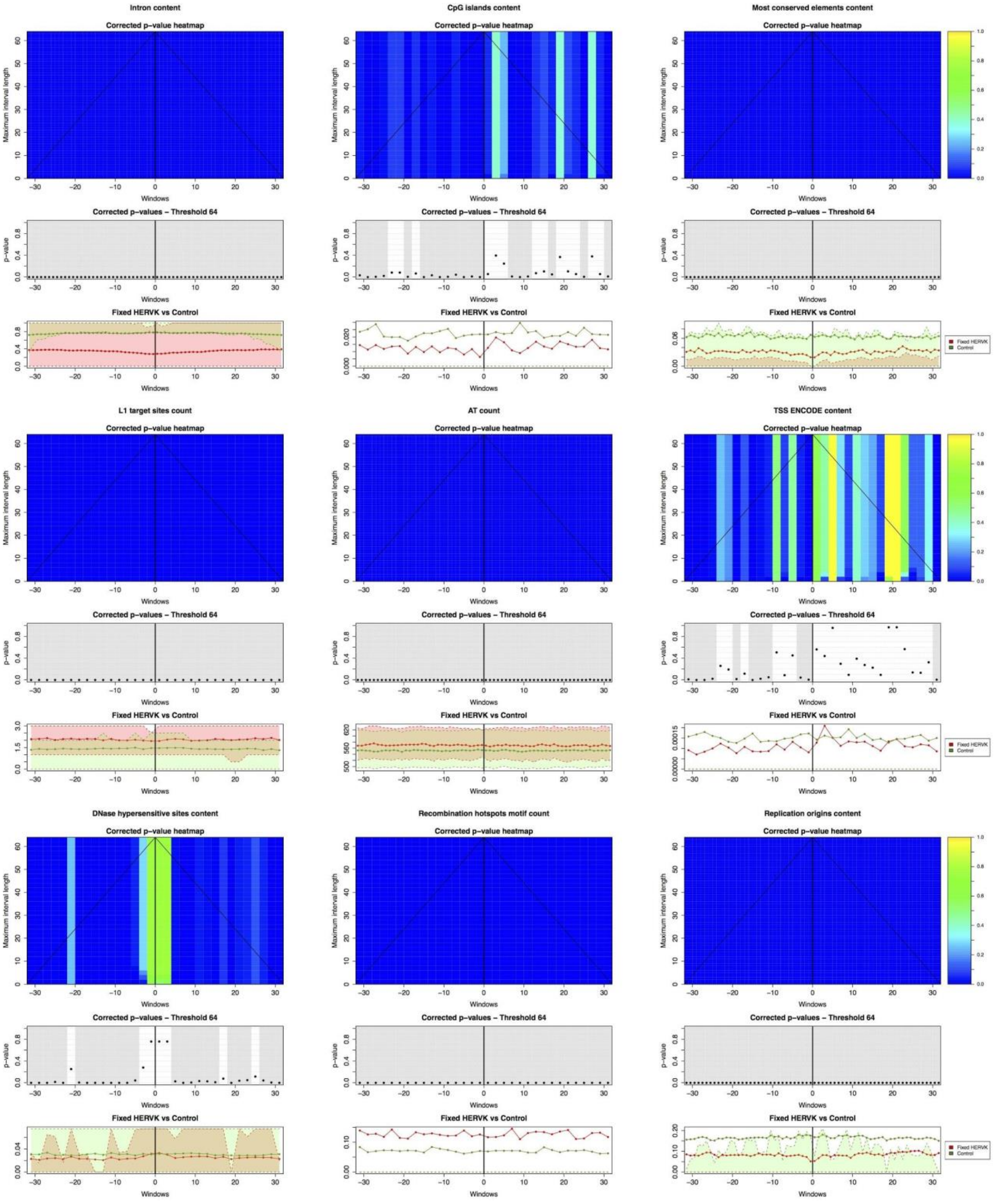

Intron content
CpG islands content
Most conserved elements content
L1 target sites count
AT count
TSS ENCODE content
DNase hypersensitive sites content
Recombination hotspots motif count
Replication origins content
Corrected p−value heatmap
Maximum interval length
Windows
Corrected p−values − Threshold 64
p−value
Fixed HERVK vs Control
Fixed HERVK
Control

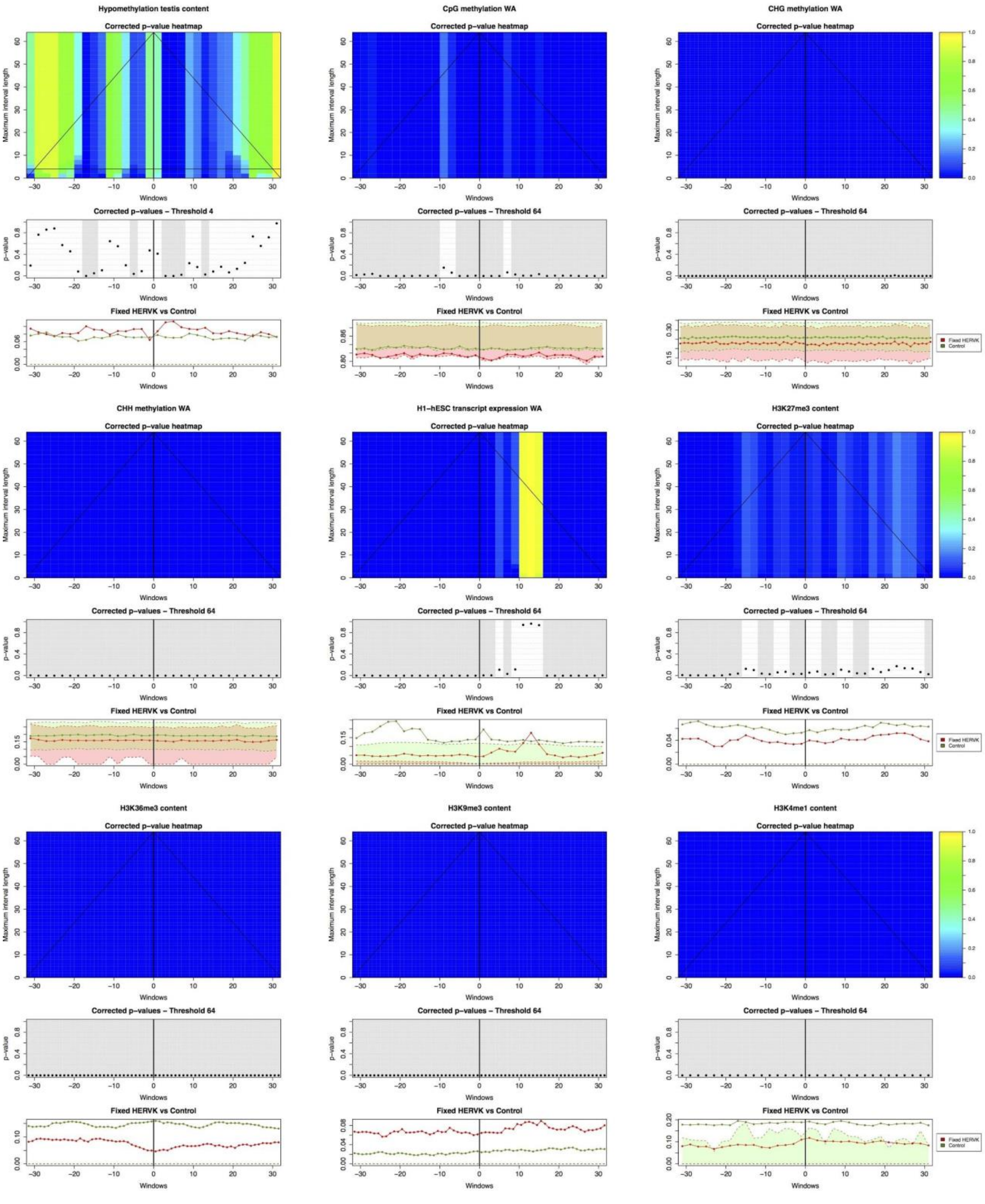
Hypomethylation testis content
CpG methylation WA
CHG methylation WA
CHH methylation WA
H1-hESC transcript expression WA
H3K27me3 content
H3K36me3 content
H3K9me3 content
H3K4me1 content
Corrected p-value heatmap
Corrected p-values – Threshold 4
Corrected p-values – Threshold 64
Fixed HERVK vs Control
Maximum interval length
p-value
Windows
Fixed HERVK
Control

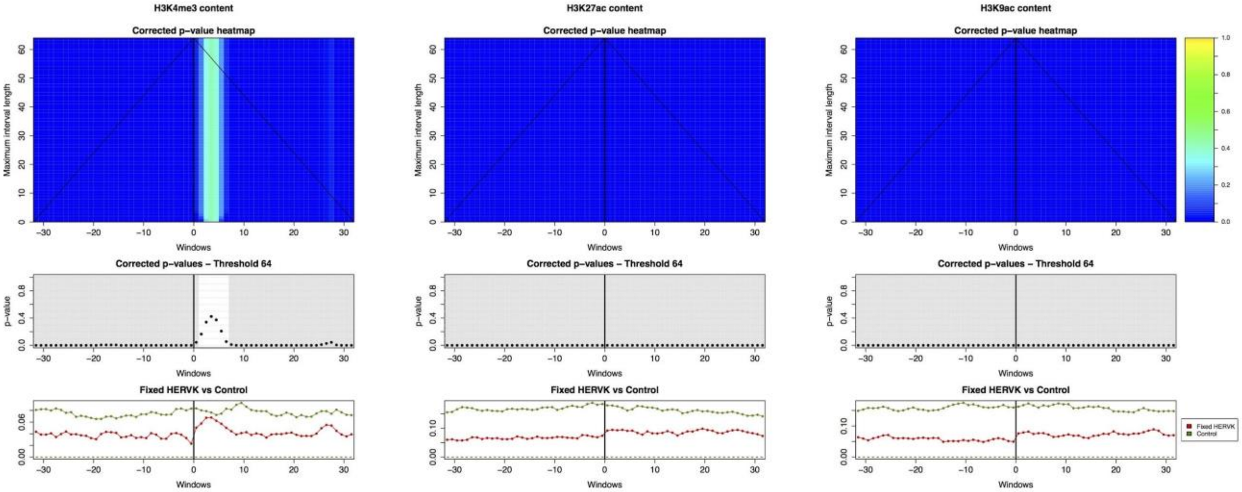

H3K4me3 content
H3K27ac content
H3K9ac content
Corrected p−value heatmap
Maximum interval length
Windows
Corrected p−values − Threshold 64
p−value
Fixed HERVK vs Control
Fixed HERVK
Control

B)

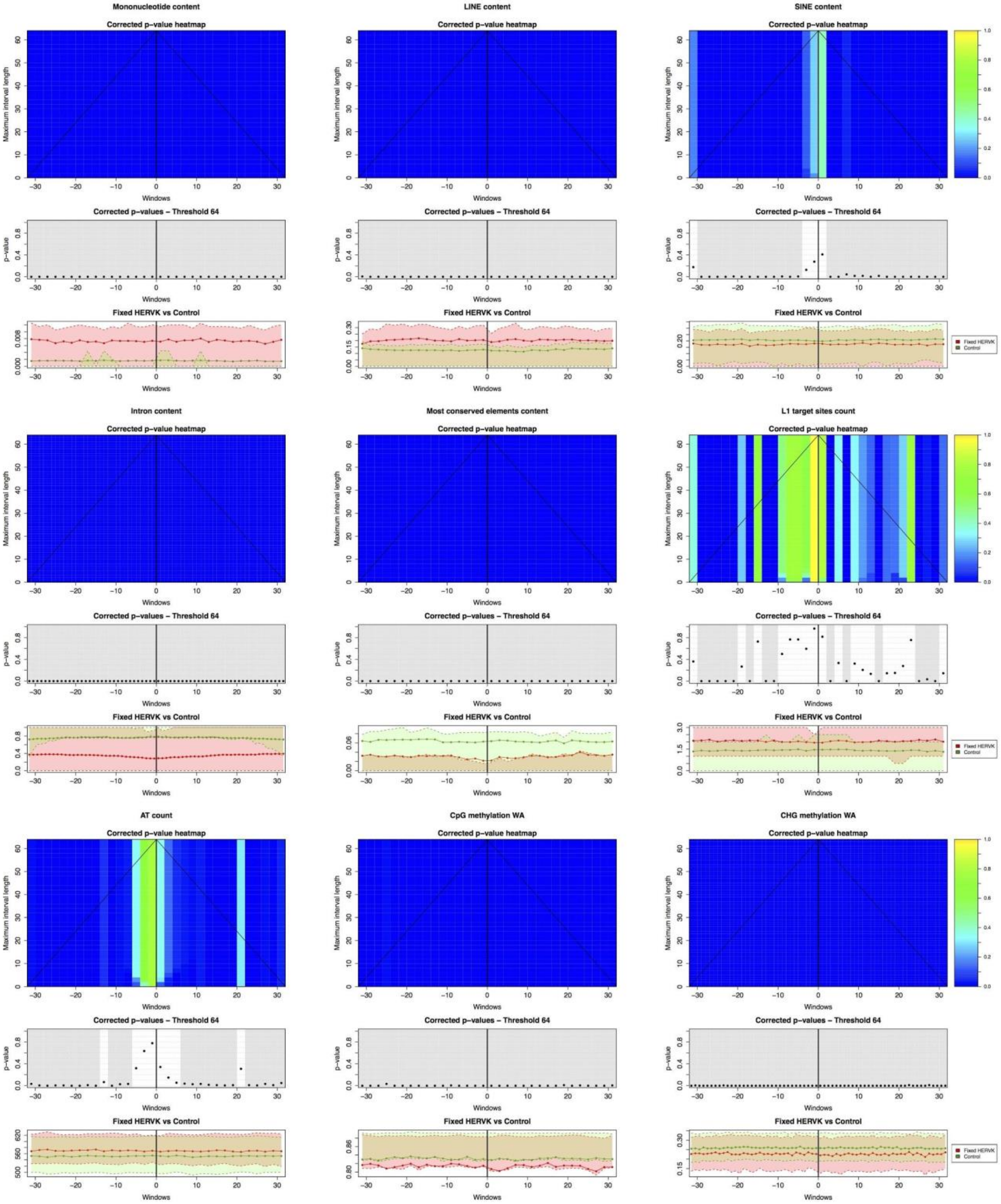
Mononucleotide content
LINE content
SINE content
Intron content
Most conserved elements content
L1 target sites count
AT count
CpG methylation WA
CHG methylation WA
Corrected p−value heatmap
Maximum interval length
Windows
Corrected p−values − Threshold 64
p−value
Fixed HERVK vs Control
Fixed HERVK
Control

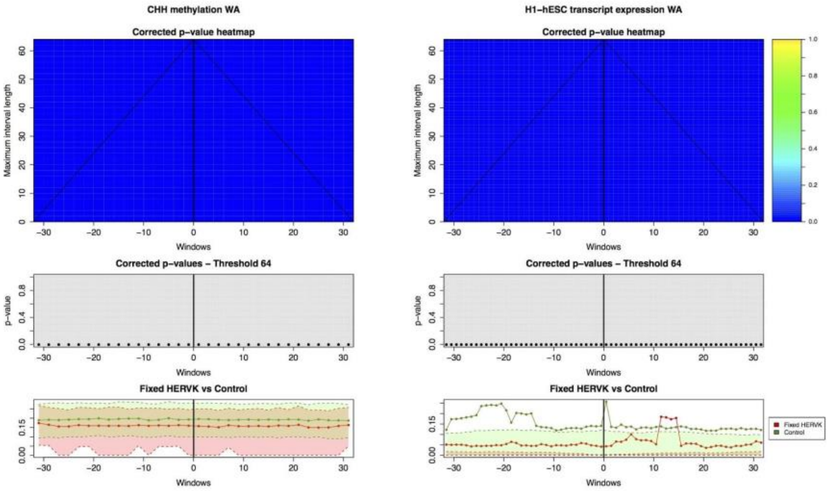

CHH methylation WA
H1−hESC transcript expression WA
Corrected p−value heatmap
Maximum interval length
Windows
Corrected p−values − Threshold 64
p−value
Fixed HERVK vs Control
Fixed HERVK
Control

C)

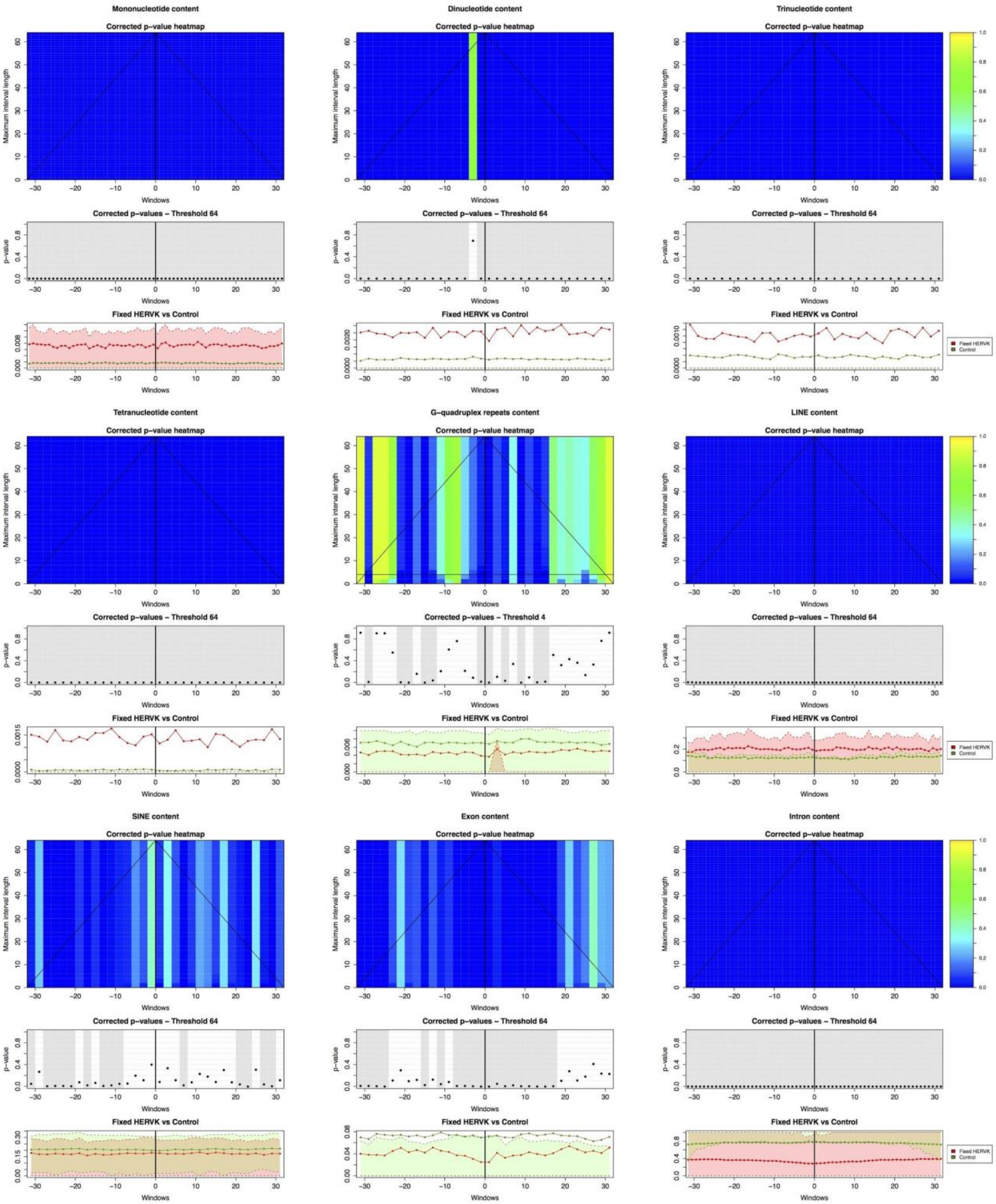
Mononucleotide content
Dinucleotide content
Trinucleotide content
Tetranucleotide content
G-quadruplex repeats content
LINE content
SINE content
Exon content
Intron content
Corrected p-value heatmap
Maximum interval length
Windows
Corrected p-values – Threshold 64
Corrected p-values – Threshold 4
p-value
Fixed HERVK vs Control
Fixed HERVK
Control

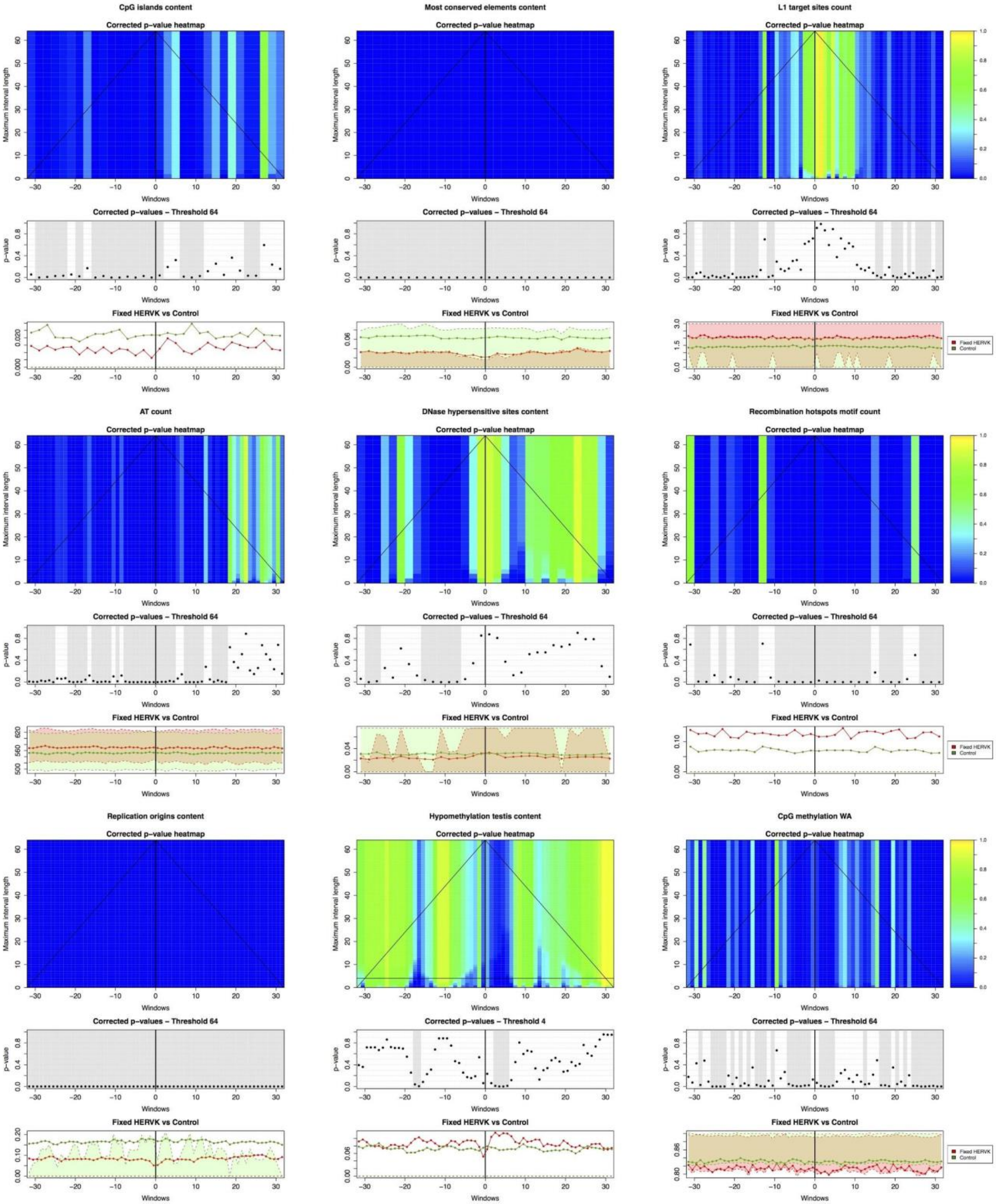
CpG islands content
Most conserved elements content
L1 target sites count
AT count
DNase hypersensitive sites content
Recombination hotspots motif count
Replication origins content
Hypomethylation testis content
CpG methylation WA
Corrected p−value heatmap
Maximum interval length
Windows
Corrected p−values − Threshold 64
Corrected p−values − Threshold 4
p−value
Fixed HERVK vs Control
Fixed HERVK
Control

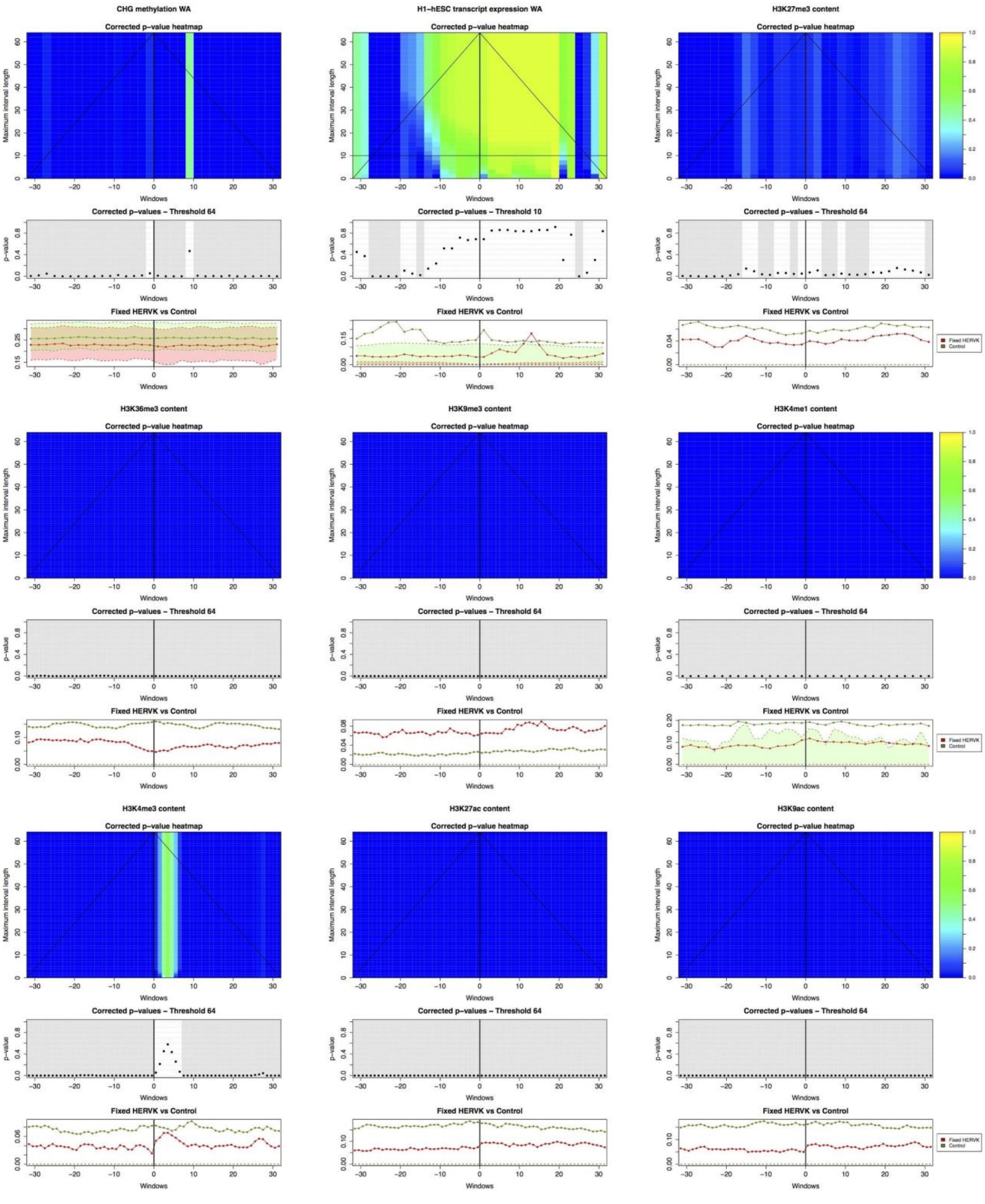
CHG methylation WA
H1−hESC transcript expression WA
H3K27me3 content
H3K36me3 content
H3K9me3 content
H3K4me1 content
H3K4me3 content
H3K27ac content
H3K9ac content
Corrected p−value heatmap
Corrected p−values − Threshold 64
Corrected p−values − Threshold 10
Fixed HERVK vs Control
Maximum interval length
Windows
p−value
Fixed HERVK
Control

Figure M. Significant genomic features for ITP of comparisons *in vitro* HERV-K vs. control with test statistics: A) sample mean difference, B) sample median difference, C) sample variance ratio.

A)

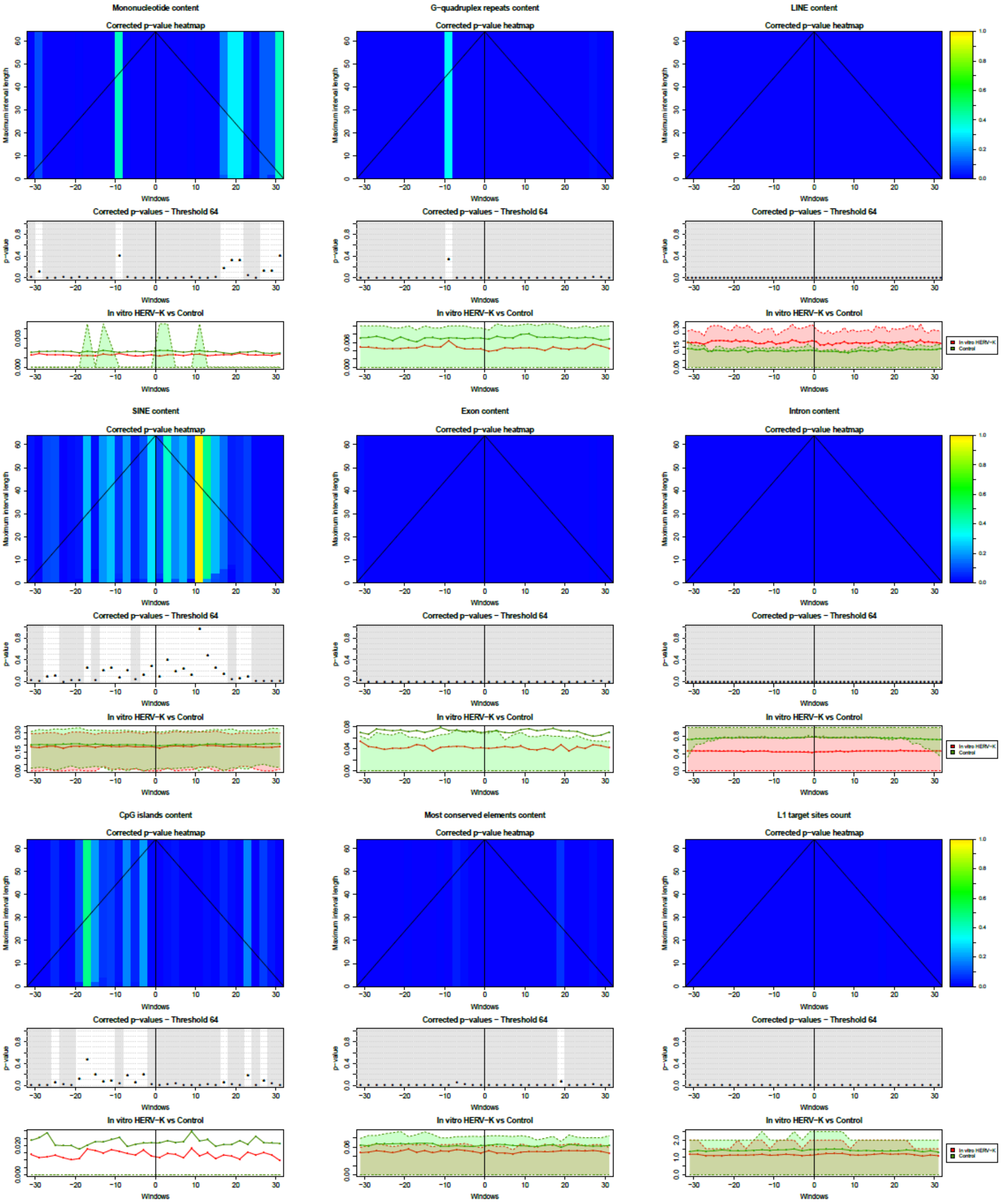

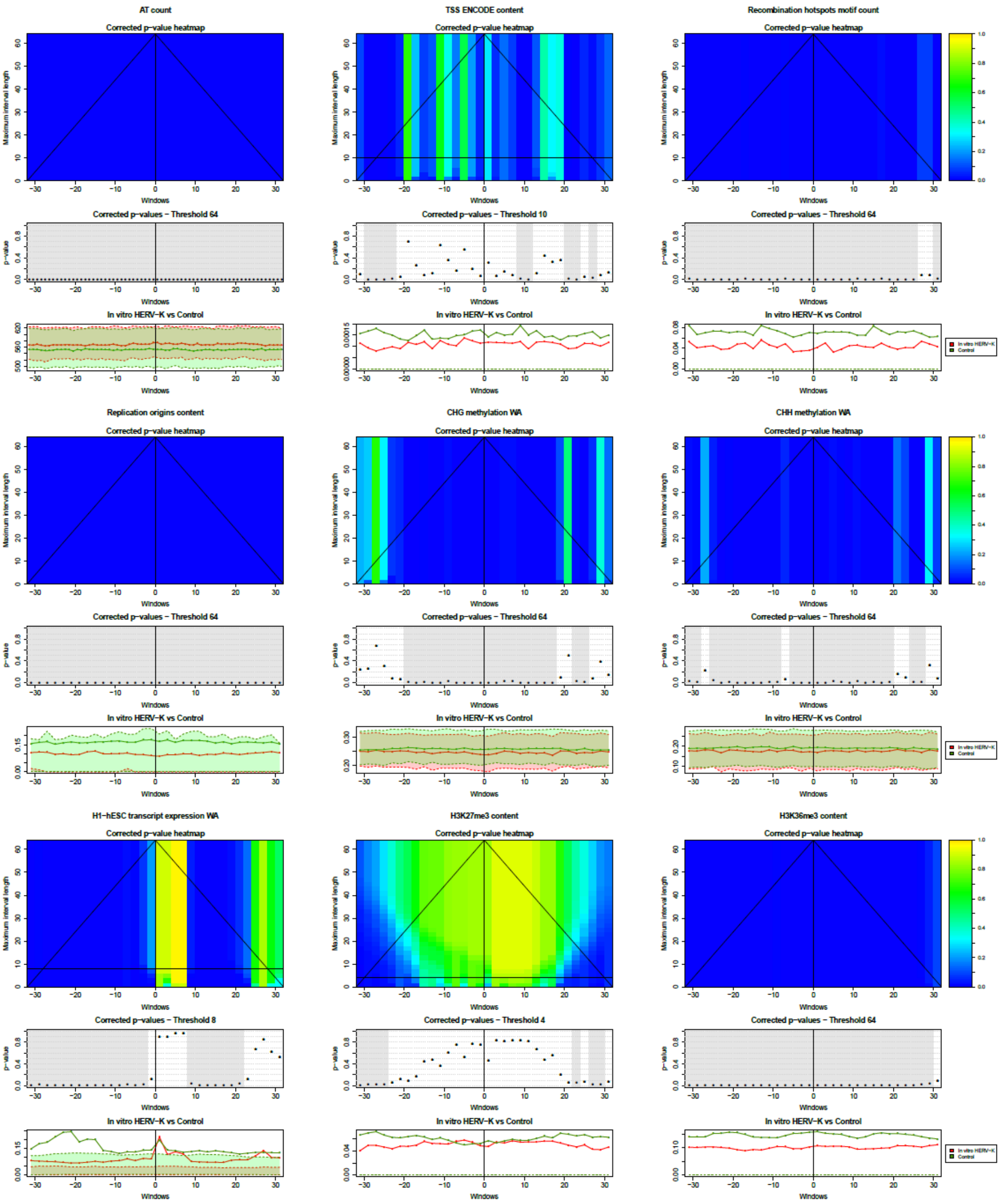
AT count
Corrected p−value heatmap
Corrected p−values − Threshold 64
In vitro HERV−K vs Control
TSS ENCODE content
Corrected p−value heatmap
Corrected p−values − Threshold 10
In vitro HERV−K vs Control
Recombination hotspots motif count
Corrected p−value heatmap
Corrected p−values − Threshold 64
In vitro HERV−K vs Control
Replication origins content
Corrected p−value heatmap
Corrected p−values − Threshold 64
In vitro HERV−K vs Control
CHG methylation WA
Corrected p−value heatmap
Corrected p−values − Threshold 64
In vitro HERV−K vs Control
CHH methylation WA
Corrected p−value heatmap
Corrected p−values − Threshold 64
In vitro HERV−K vs Control
H1−hESC transcript expression WA
Corrected p−value heatmap
Corrected p−values − Threshold 8
In vitro HERV−K vs Control
H3K27me3 content
Corrected p−value heatmap
Corrected p−values − Threshold 4
In vitro HERV−K vs Control
H3K36me3 content
Corrected p−value heatmap
Corrected p−values − Threshold 64
In vitro HERV−K vs Control
Maximum interval length
p−value
Windows

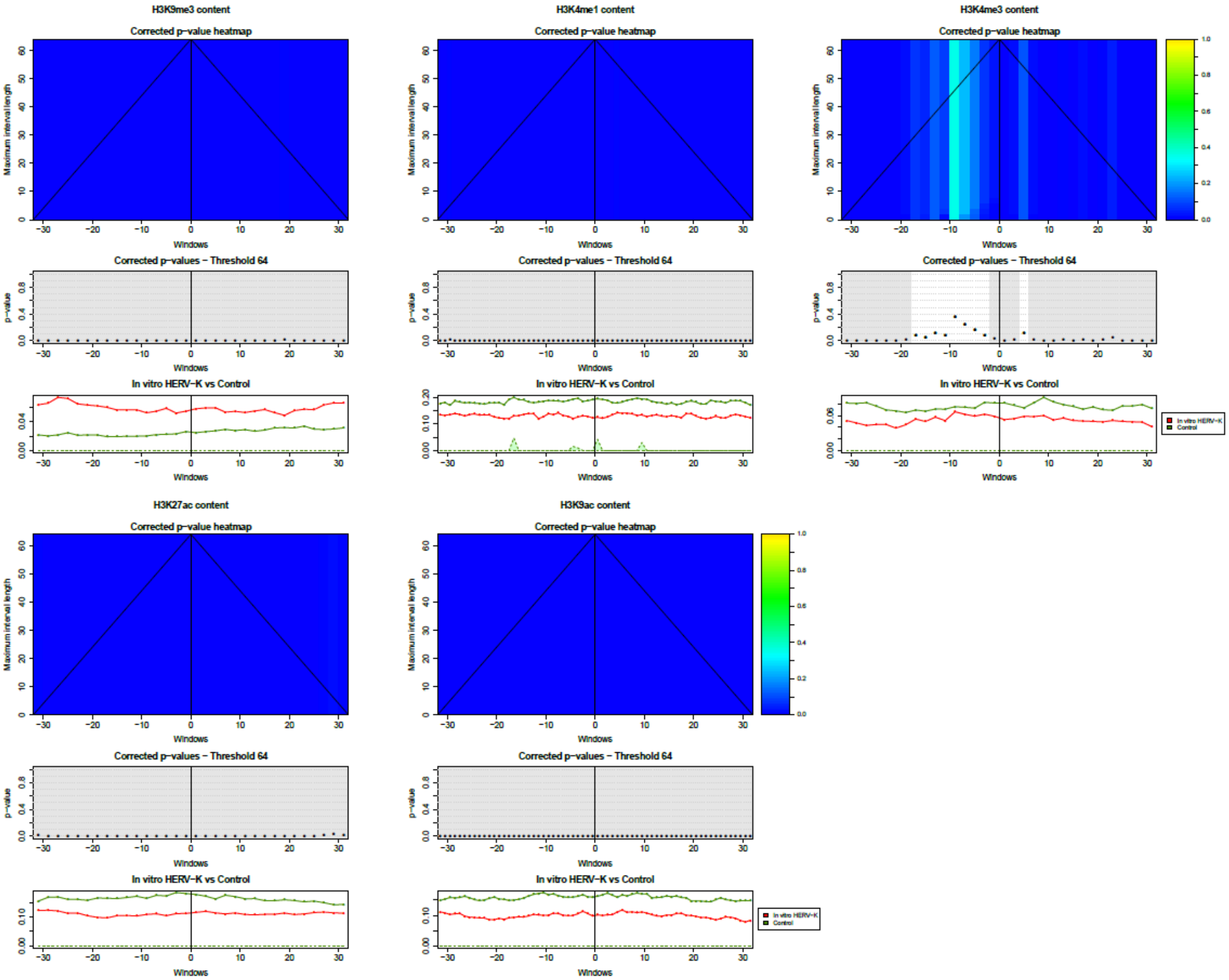
H3K9me3 content
H3K4me1 content
H3K4me3 content
H3K27ac content
H3K9ac content
Corrected p−value heatmap
Maximum interval length
Windows
Corrected p−values − Threshold 64
p−value
In vitro HERV−K vs Control
In vitro HERV−K
Control

B)

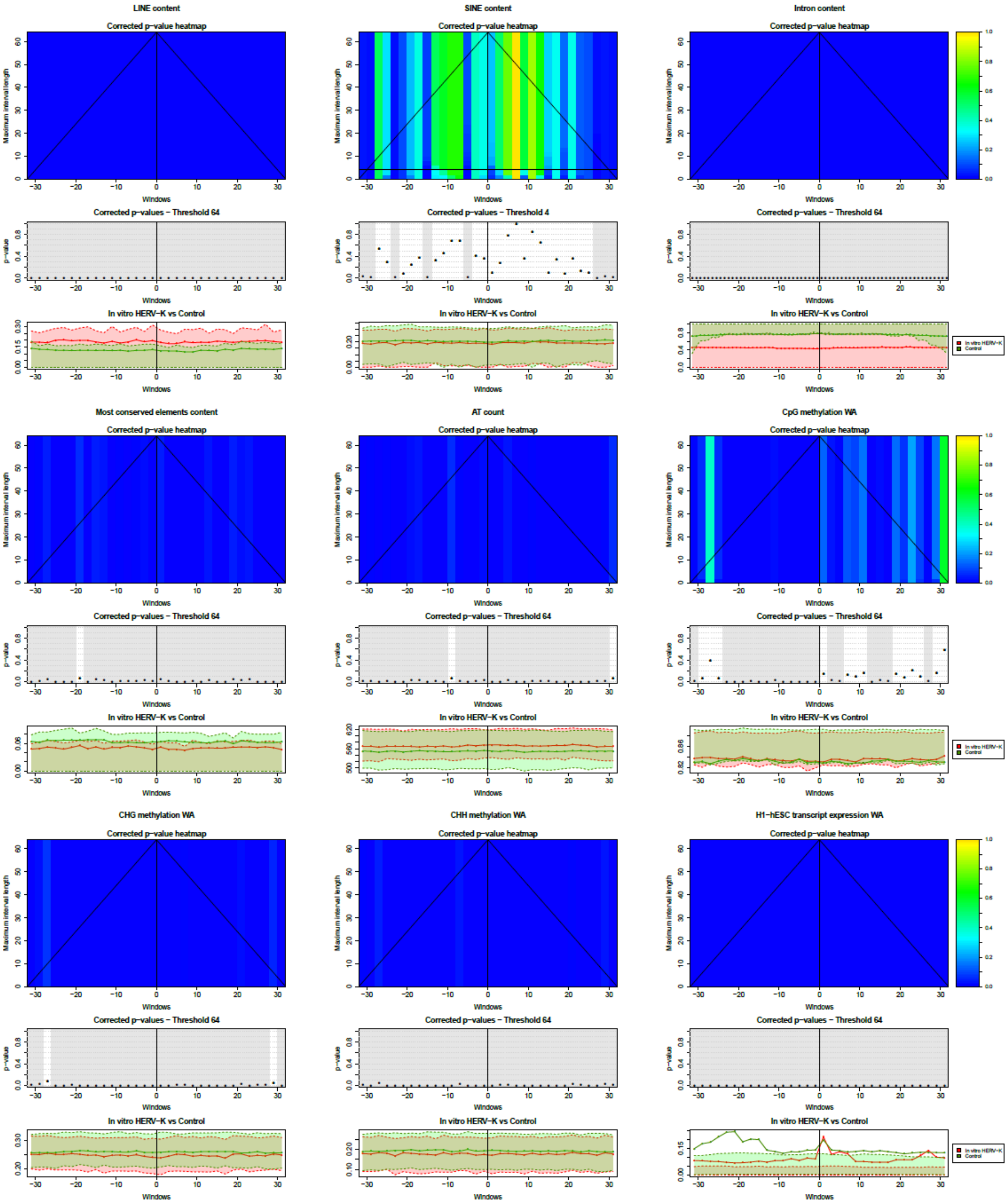
LINE content
SINE content
Intron content
Most conserved elements content
AT count
CpG methylation WA
CHG methylation WA
CHH methylation WA
H1−hESC transcript expression WA
Corrected p−value heatmap
Maximum interval length
Windows
Corrected p−values − Threshold 64
Corrected p−values − Threshold 4
p−value
In vitro HERV−K vs Control
In vitro HERV−K
Control

C)

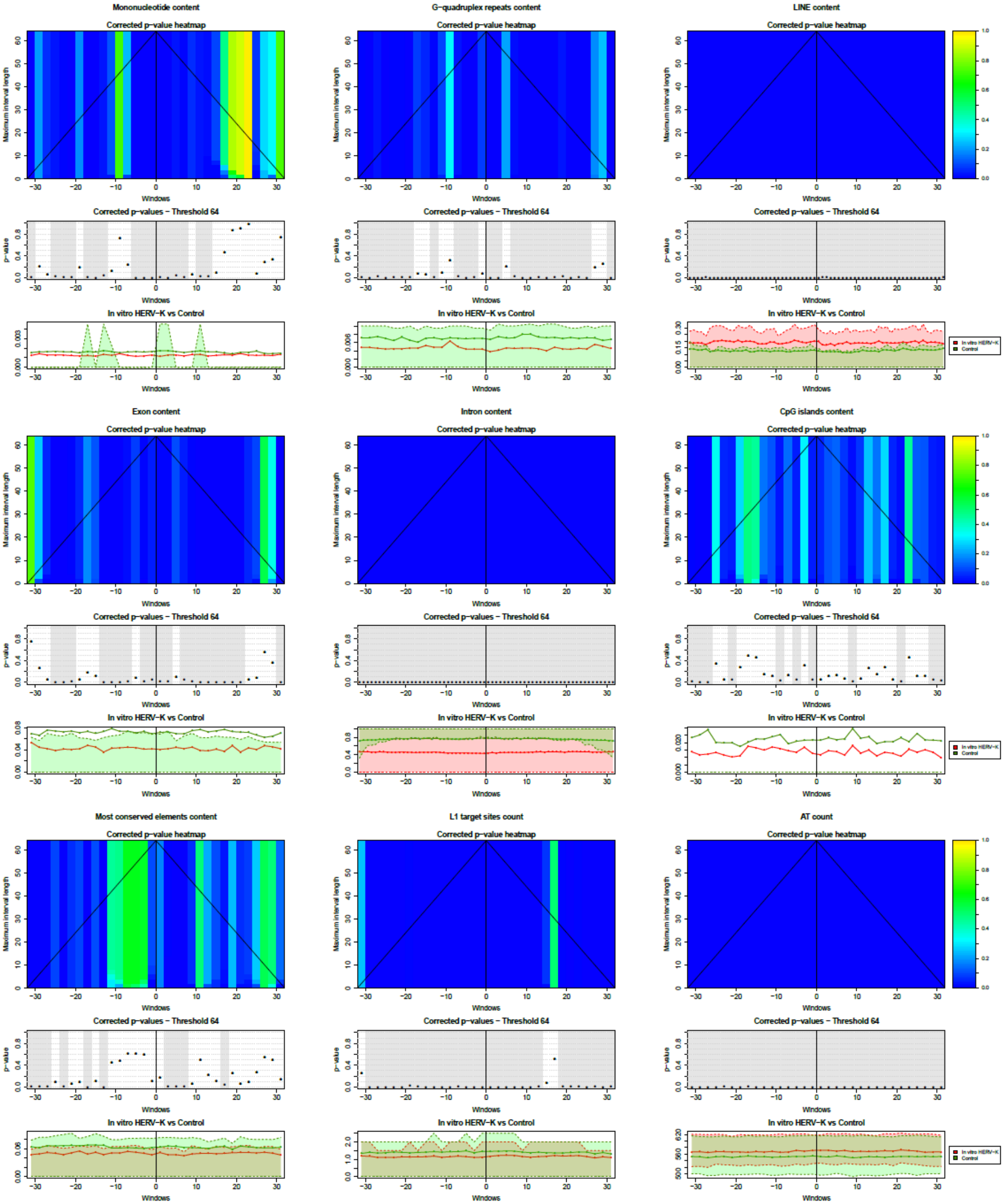
Mononucleotide content
G−quadruplex repeats content
LINE content
Exon content
Intron content
CpG islands content
Most conserved elements content
L1 target sites count
AT count
Corrected p−value heatmap
Corrected p−values − Threshold 64
In vitro HERV−K vs Control
Maximum interval length
Windows
p−value

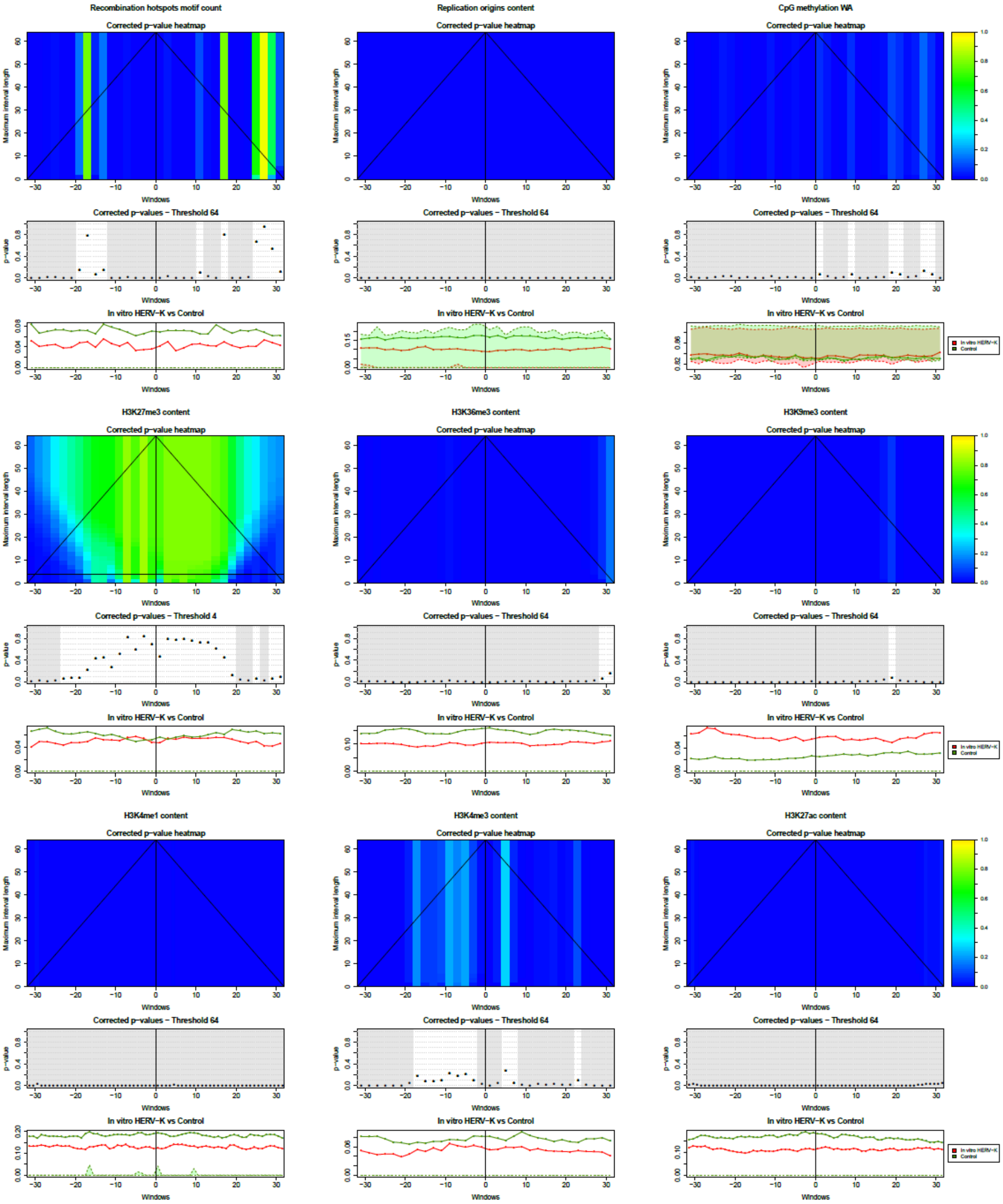

Recombination hotspots motif count
Replication origins content
CpG methylation WA
H3K27me3 content
H3K36me3 content
H3K9me3 content
H3K4me1 content
H3K4me3 content
H3K27ac content
Corrected p−value heatmap
Corrected p−values − Threshold 64
Corrected p−values − Threshold 4
In vitro HERV−K vs Control
Maximum interval length
p−value
Windows
In vitro HERV−K
Control

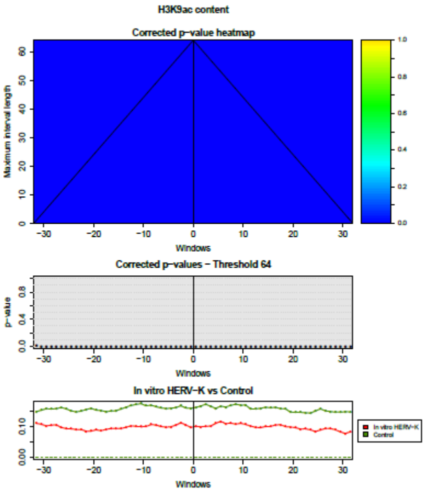

H3K9ac content
Corrected p−value heatmap
Maximum interval length
Windows
Corrected p−values − Threshold 64
p−value
Windows
In vitro HERV−K vs Control
In vitro HERV−K
Control
Windows

Figure N. Significant genomic features for ITP of comparisons fixed vs. *in vitro* HERV-K with test statistics: A) sample mean difference, B) sample median difference, C) sample variance ratio.

A)

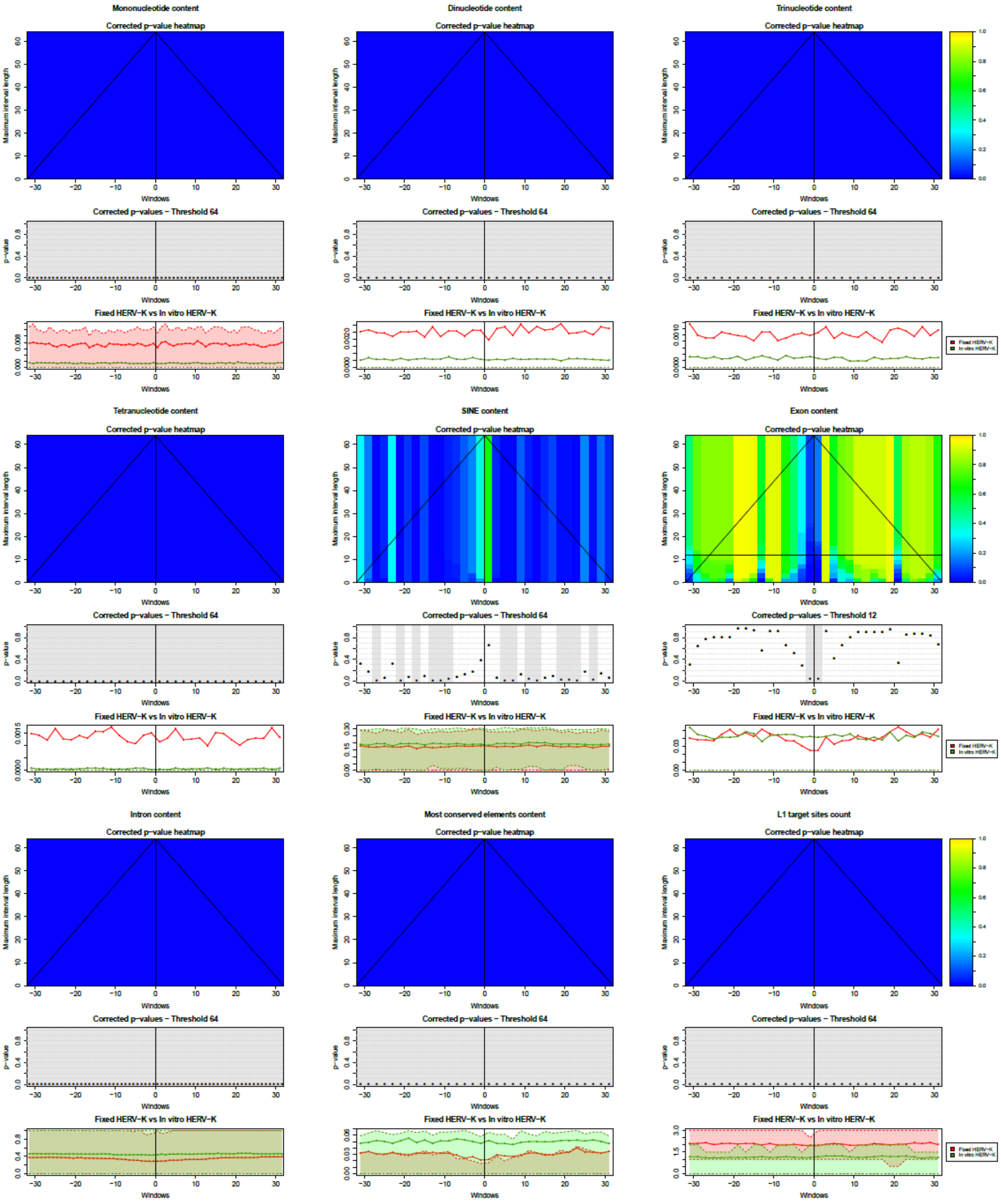

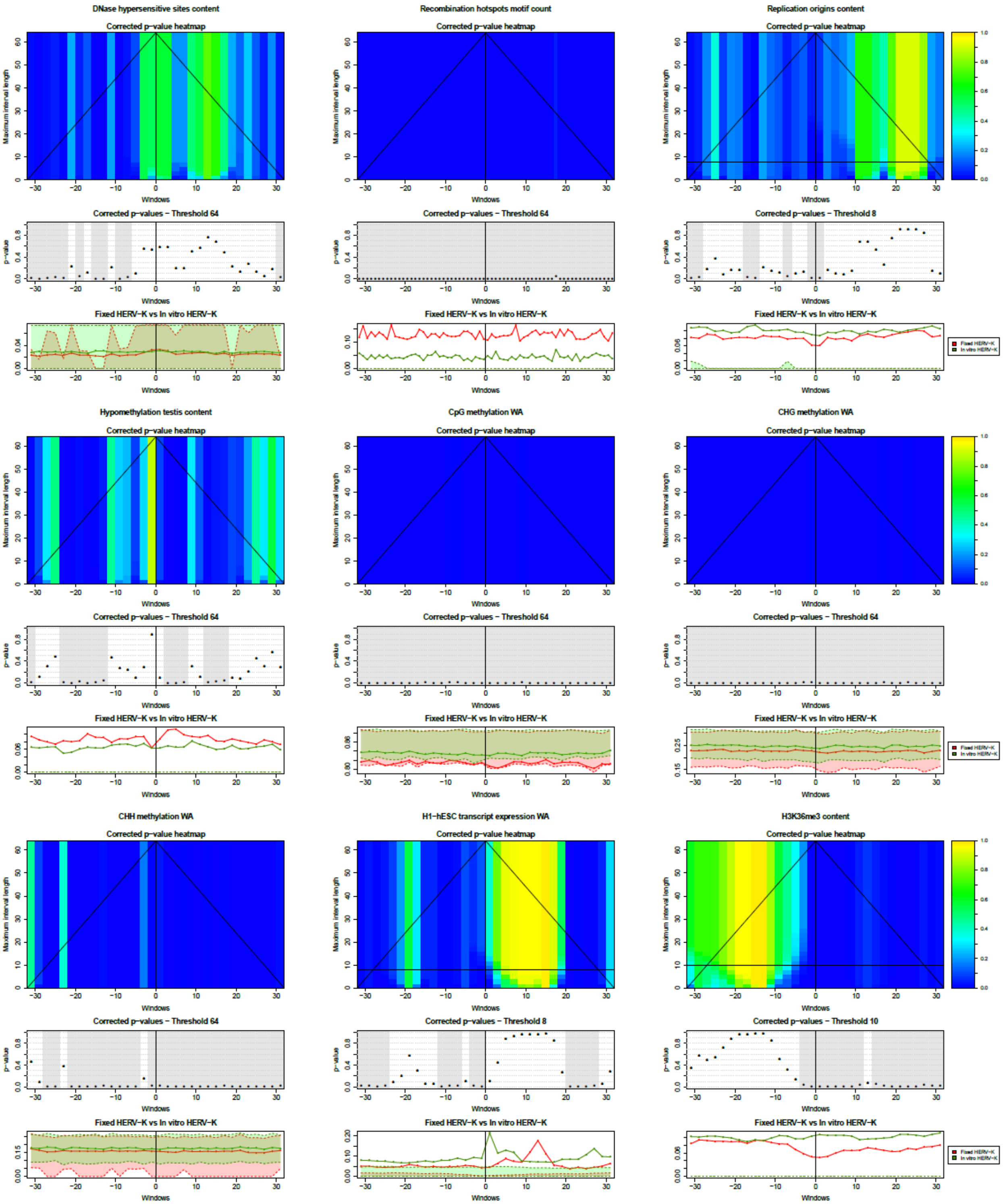
DNase hypersensitive sites content
Recombination hotspots motif count
Replication origins content
Corrected p-value heatmap
Maximum interval length
Windows
Corrected p-values − Threshold 64
Corrected p-values − Threshold 8
p−value
Fixed HERV−K vs In vitro HERV−K
Fixed HERV−K
In vitro HERV−K
Hypomethylation testis content
CpG methylation WA
CHG methylation WA
CHH methylation WA
H1−hESC transcript expression WA
H3K36me3 content
Corrected p-values − Threshold 10

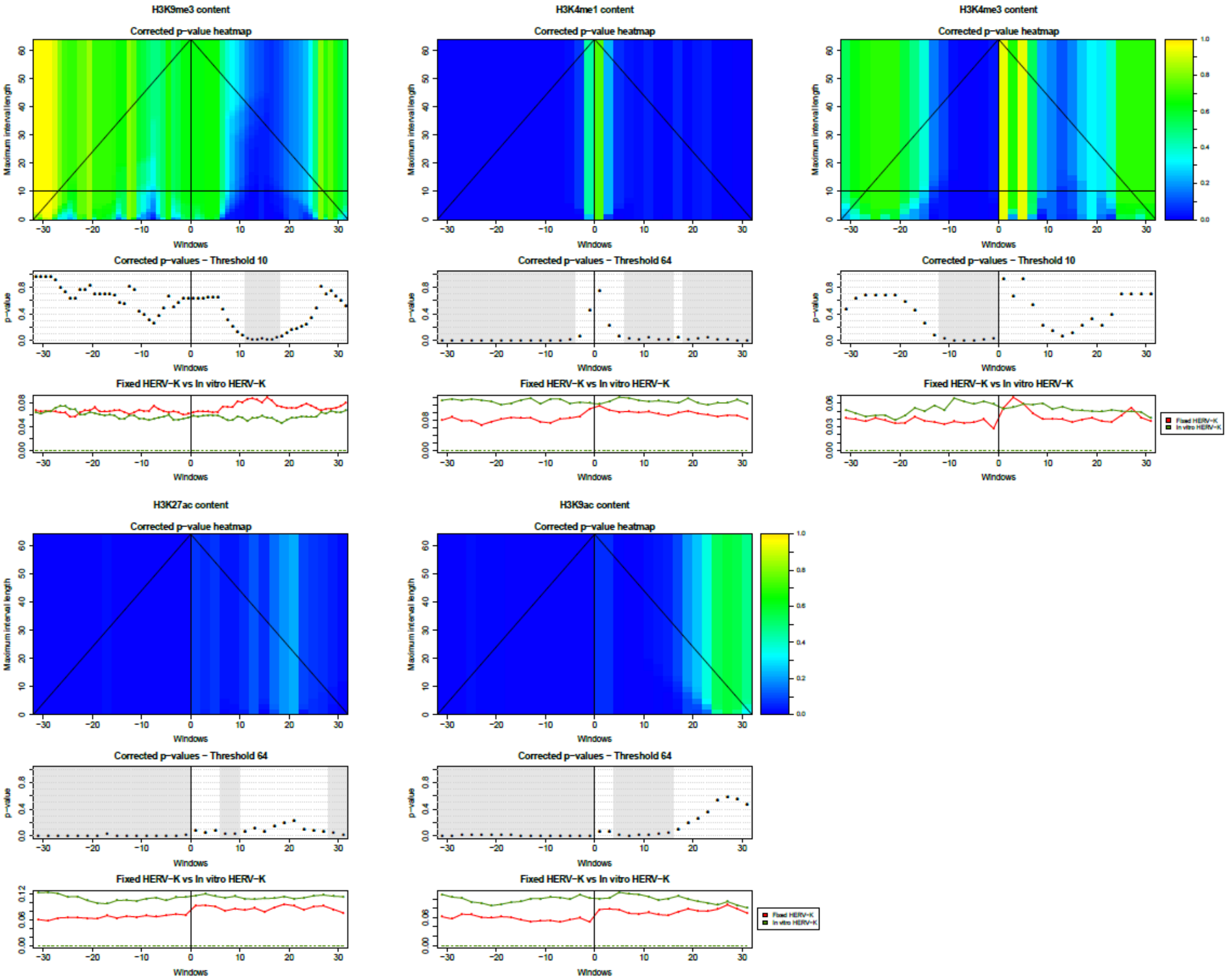
H3K9me3 content
Corrected p−value heatmap
Maximum interval length
Windows
Corrected p−values − Threshold 10
p−value
Fixed HERV−K vs In vitro HERV−K
H3K4me1 content
Corrected p−values − Threshold 64
H3K4me3 content
Fixed HERV−K
In vitro HERV−K
H3K27ac content
H3K9ac content

B)

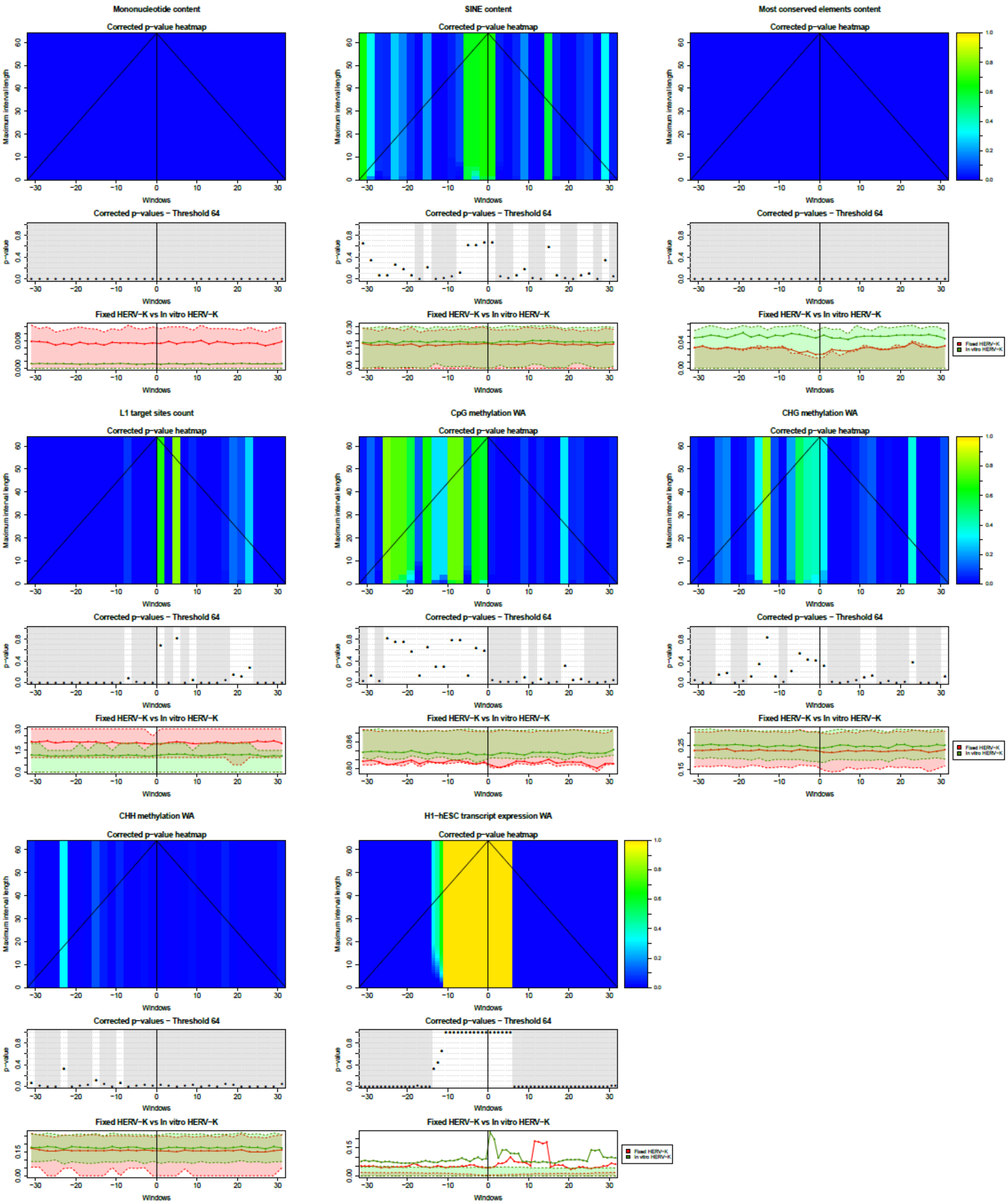

Mononucleotide content
SINE content
Most conserved elements content
L1 target sites count
CpG methylation WA
CHG methylation WA
CHH methylation WA
H1-hESC transcript expression WA
Corrected p-value heatmap
Corrected p-values - Threshold 64
Fixed HERV-K vs In vitro HERV-K
Maximum interval length
p-value
Windows

C)

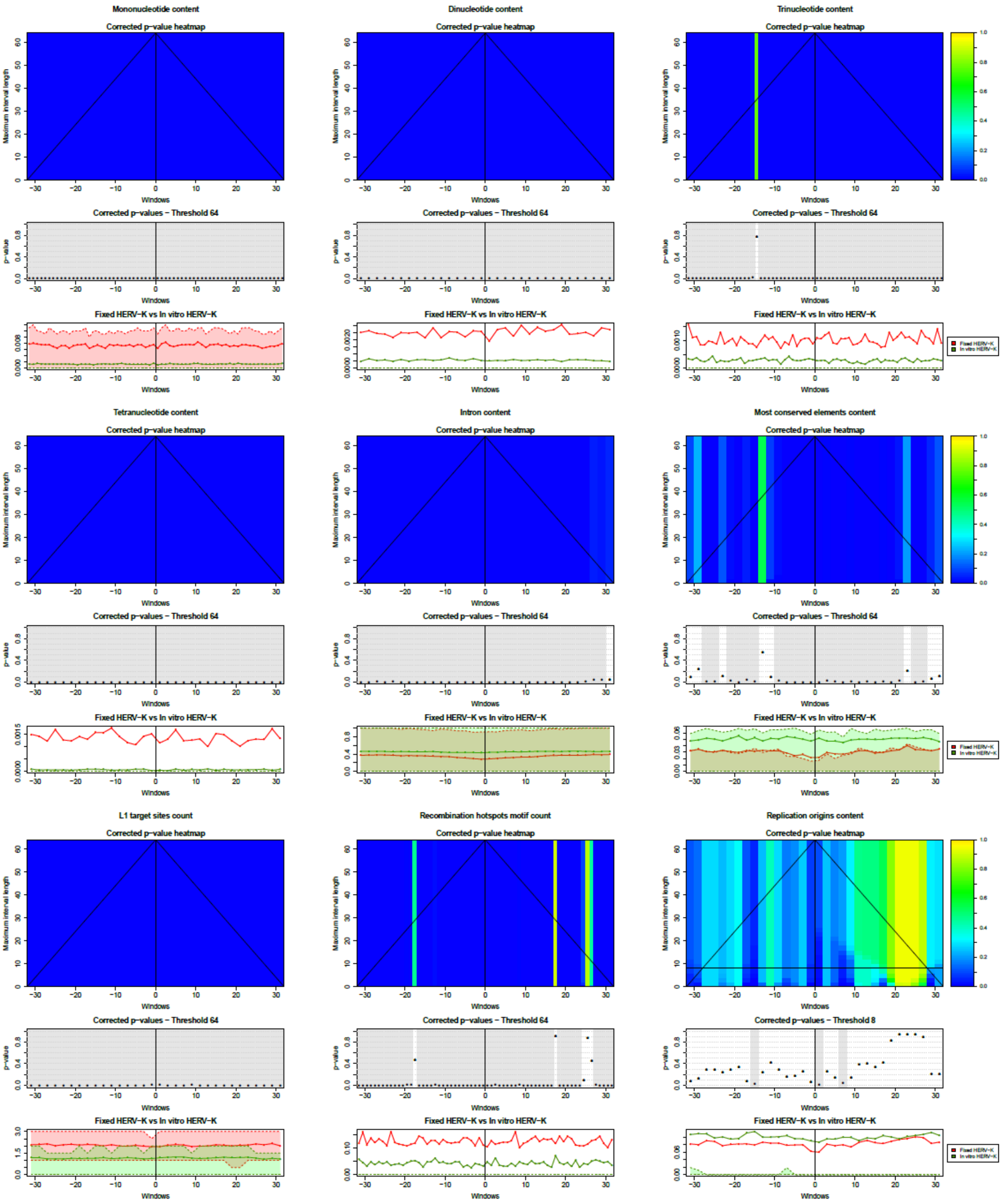
Mononucleotide content
Dinucleotide content
Trinucleotide content
Corrected p-value heatmap
Maximum interval length
Windows
Corrected p-values – Threshold 64
p-value
Fixed HERV-K vs In vitro HERV-K
Tetranucleotide content
Intron content
Most conserved elements content
L1 target sites count
Recombination hotspots motif count
Replication origins content
Corrected p-values – Threshold 8
Fixed HERV-K
In vitro HERV-K

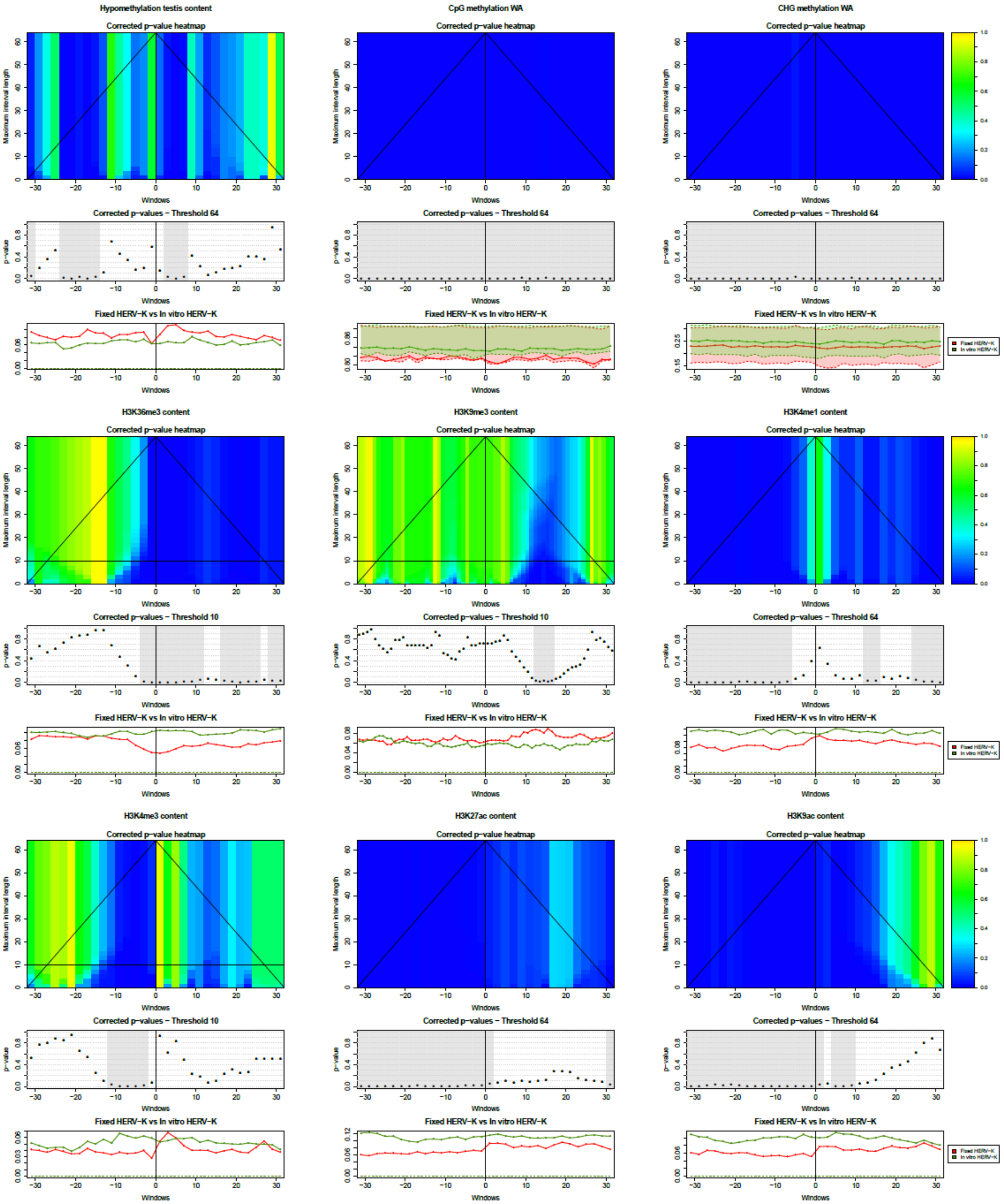
Hypomethylation testis content
CpG methylation WA
CHG methylation WA
H3K36me3 content
H3K9me3 content
H3K4me1 content
H3K4me3 content
H3K27ac content
H3K9ac content
Corrected p−value heatmap
Maximum interval length
Windows
Corrected p−values − Threshold 64
Corrected p−values − Threshold 10
p−value
Fixed HERV−K vs In vitro HERV−K
Fixed HERV−K
In vitro HERV−K

Figure O. Significance (i.e. -log10(corrected p-value)) of genomic features in windows along the flanking regions, obtained from the ITP using the **median** difference as test statistics: (A) fixed ETns vs. controls, (B) polymorphic ETns vs. controls, and (C) fixed vs. polymorphic ETns. In each panel, the horizontal axis represents the 64 1-kb windows. The vertical black line between window -1 kb and 1 kb marks the integration site. The thresholds reported on the left represent the maximum scale at which each feature is significant, ranging from 64 kb (coarsest) to 1 kb (finest). Each row corresponds to one feature and each cell represents one or two contiguous windows, depending on the number of nodes employed in the B-splines (we consider one value for every 1-kb window when using the raw data, and one value every two 1-kb windows when using the piecewise constant smoothed version of the data).

White cells: not significant (p-value >0.05), red cells: significant with higher median in the flanking regions of ETns vs. controls (or in the flanking regions of fixed vs. polymorphic ETns), blue cells: significant with lower median in the flanking regions of ETns vs. controls (or in the flanking regions of fixed vs. polymorphic ETns). Color intensity is proportional to significance (more intense colors correspond to lower corrected p-values).

A)

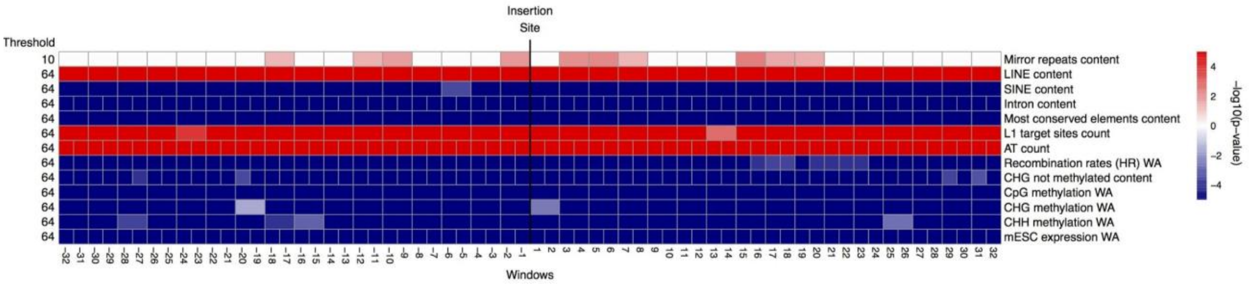

B)

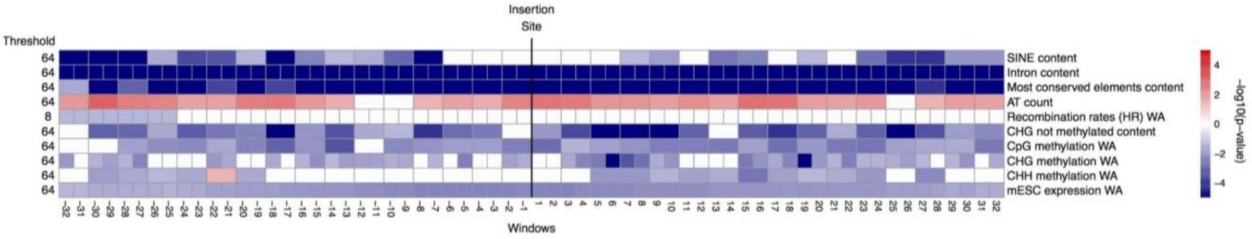

C)

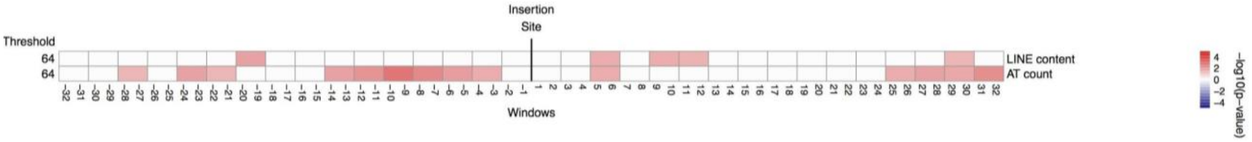

Figure P. Significance (i.e. -log10(corrected p-value)) of genomic features in windows along the flanking regions, obtained from the ITP using the **median** difference as test statistics: (A) fixed IAPs vs. controls, (B) polymorphic IAPs vs. controls, and (C) fixed vs. polymorphic IAPs. In each panel, the horizontal axis represents the 64 1-kb windows. The vertical black line between window -1 kb and 1 kb marks the integration site. The thresholds reported on the left represent the maximum scale at which each feature is significant, ranging from 64 kb (coarsest) to 1 kb (finest). Each row corresponds to one feature and each cell represents one or two contiguous windows, depending on the number of nodes employed in the B-splines (we consider one value for every 1-kb window when using the raw data, and one value every two 1-kb windows when using the piecewise constant smoothed version of the data).

White cells: not significant (p-value >0.05), red cells: significant with higher median in the flanking regions of IAPs vs. controls (or in the flanking regions of fixed vs. polymorphic IAPs), blue cells: significant with lower median in the flanking regions of IAPs vs. controls (or in the flanking regions of fixed vs. polymorphic IAPs). Color intensity is proportional to significance (more intense colors correspond to lower corrected p-values).

A)

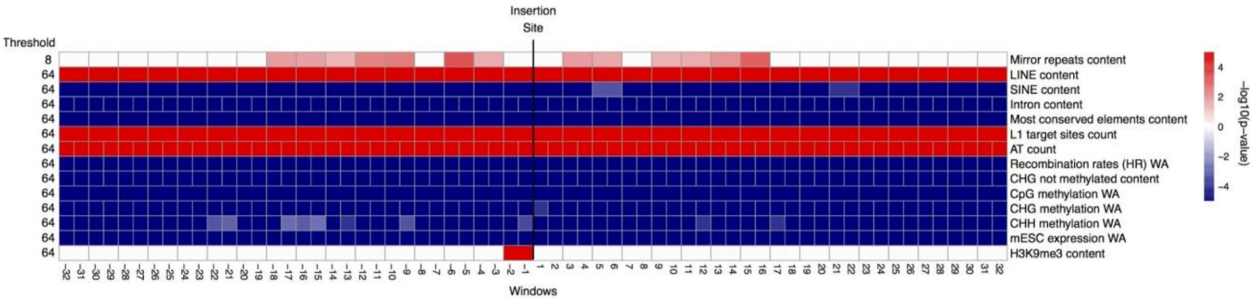

B)

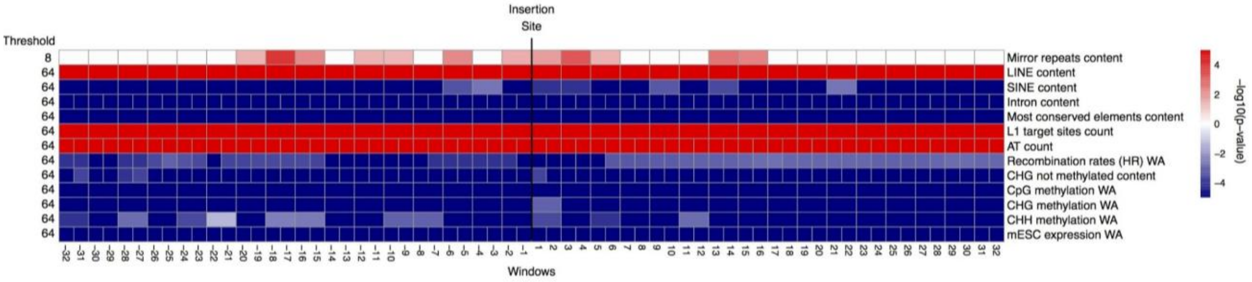

C)

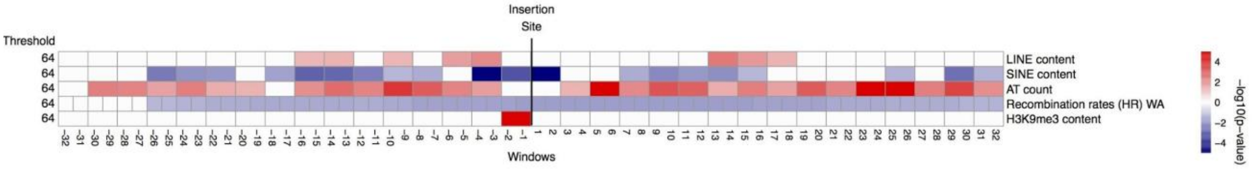

Figure Q. Significance (i.e. -log10(corrected p-value)) of genomic features in windows along the flanking regions, obtained from the ITP using the **median** difference as test statistics: (A) fixed HERV-Ks vs. controls, (B) *in vitro* HERV-Ks vs. controls, and (C) fixed vs. *in vitro* HERV-Ks. In each panel, the horizontal axis represents the 64 1-kb windows. The vertical black line between window -1 kb and 1 kb marks the integration site. The thresholds reported on the left represent the maximum scale at which each feature is significant, ranging from 64 kb (coarsest) to 1 kb (finest). Each row corresponds to one feature and each cell represents one or two contiguous windows, depending on the number of nodes employed in the B-splines (we consider one value for every 1-kb window when using the raw data, and one value every two 1-kb windows when using the piecewise constant smoothed version of the data).

White cells: not significant (p-value >0.05), red cells: significant with higher median in the flanking regions of HERV-Ks vs. controls (or in the flanking regions of fixed vs. *in vitro* HERV-Ks), blue cells: significant with lower median in the flanking regions of HERV-Ks vs. controls (or in the flanking regions of fixed vs. *in vitro* HERV-Ks). Color intensity is proportional to significance (more intense colors correspond to lower corrected p-values).

A)

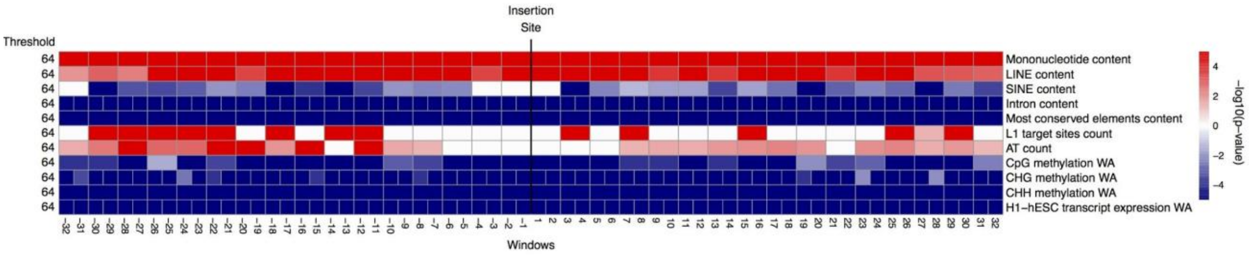

B)

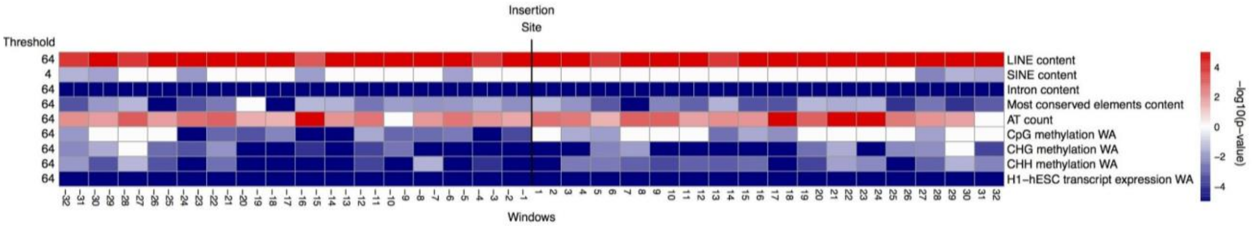

C)

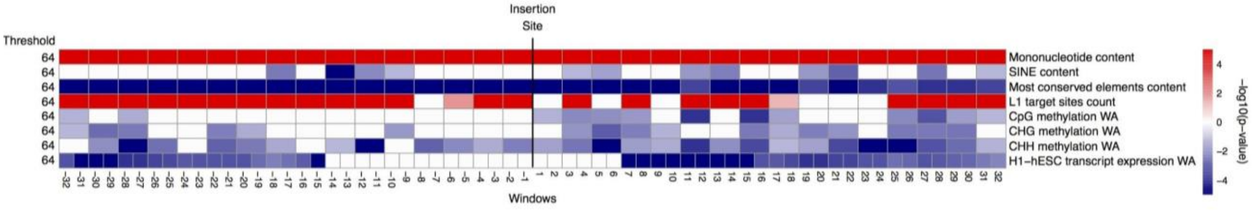

Figure R. Significance (i.e. -log10(corrected p-value)) of genomic features in windows along the flanking regions, obtained from the ITP using the **variance** difference as test statistics: (A) fixed ETns vs. controls, (B) polymorphic ETns vs. controls, and (C) fixed vs. polymorphic ETns. In each panel, the horizontal axis represents the 64 1-kb windows. The vertical black line between window -1 kb and 1 kb marks the integration site. The thresholds reported on the left represent the maximum scale at which each feature is significant, ranging from 64 kb (coarsest) to 1 kb (finest). Each row corresponds to one feature and each cell represents one or two contiguous windows, depending on the number of nodes employed in the B-splines (we consider one value for every 1-kb window when using the raw data, and one value every two 1-kb windows when using the piecewise constant smoothed version of the data).
White cells: not significant (p-value >0.05), red cells: significant with higher variance in the flanking regions of ETns vs. controls (or in the flanking regions of fixed vs. polymorphic ETns), blue cells: significant with lower variance in the flanking regions of ETns vs. controls (or in the flanking regions of fixed vs. polymorphic ETns). Color intensity is proportional to significance (more intense colors correspond to lower corrected p-values).

A)

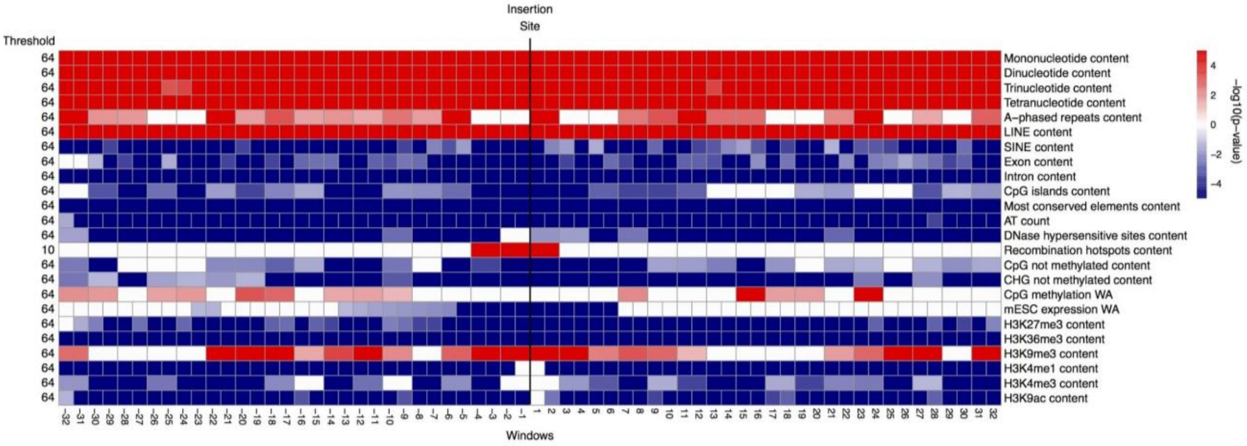

B)

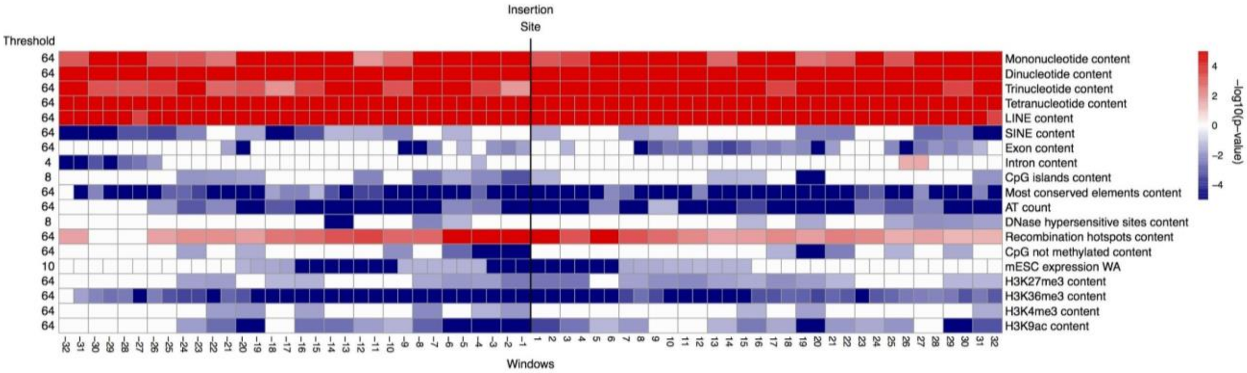

C)

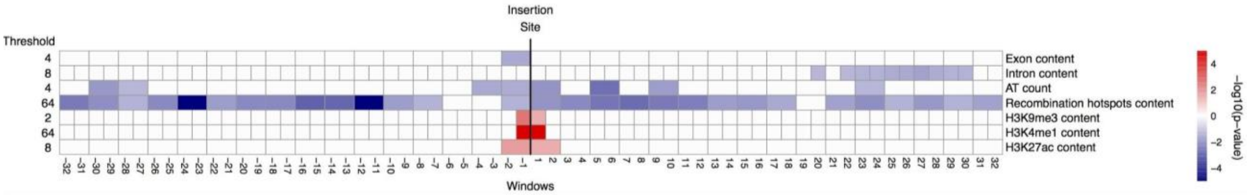
Insertion Site
Threshold
4
8
4
64
2
64
8
Exon content
Intron content
AT count
Recombination hotspots content
H3K9me3 content
H3K4me1 content
H3K27ac content
Windows
−log10(p−value)
4
2
0
−2
−4

Figure S. Significance (i.e. -log10(corrected p-value)) of genomic features in windows along the flanking regions, obtained from the ITP using the **variance** difference as test statistics: (A) fixed IAPs vs. controls, (B) polymorphic IAPs vs. controls, and (C) fixed vs. polymorphic IAPs. In each panel, the horizontal axis represents the 64 1-kb windows. The vertical black line between window -1 kb and 1 kb marks the integration site. The thresholds reported on the left represent the maximum scale at which each feature is significant, ranging from 64 kb (coarsest) to 1 kb (finest). Each row corresponds to one feature and each cell represents one or two contiguous windows, depending on the number of nodes employed in the B-splines (we consider one value for every 1-kb window when using the raw data, and one value every two 1-kb windows when using the piecewise constant smoothed version of the data).

White cells: not significant (p-value >0.05), red cells: significant with higher variance in the flanking regions of IAPs vs. controls (or in the flanking regions of fixed vs. polymorphic IAPs), blue cells: significant with lower variance in the flanking regions of IAPs vs. controls (or in the flanking regions of fixed vs. polymorphic IAPs). Color intensity is proportional to significance (more intense colors correspond to lower corrected p-values).

A)

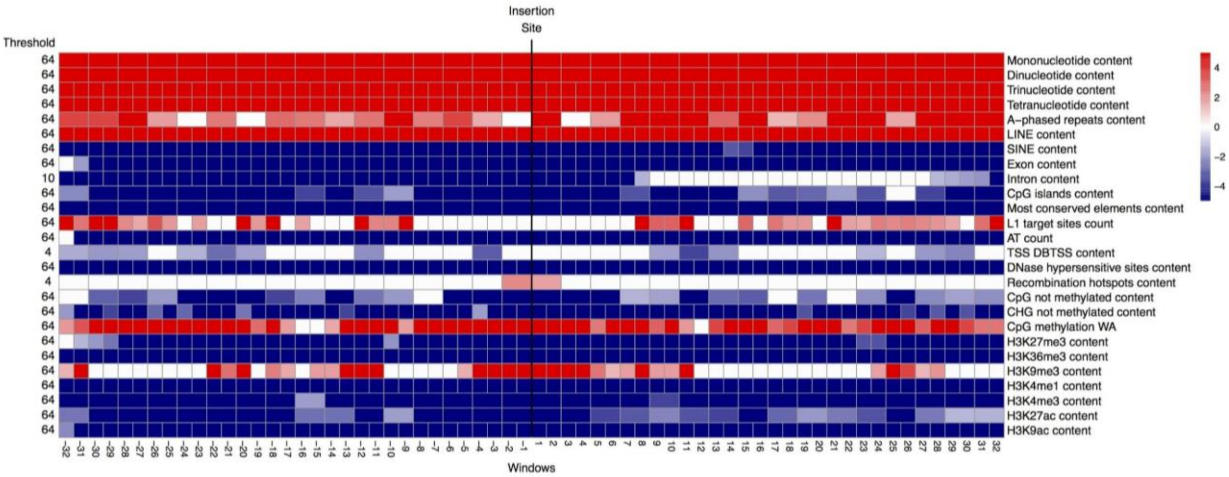

B)

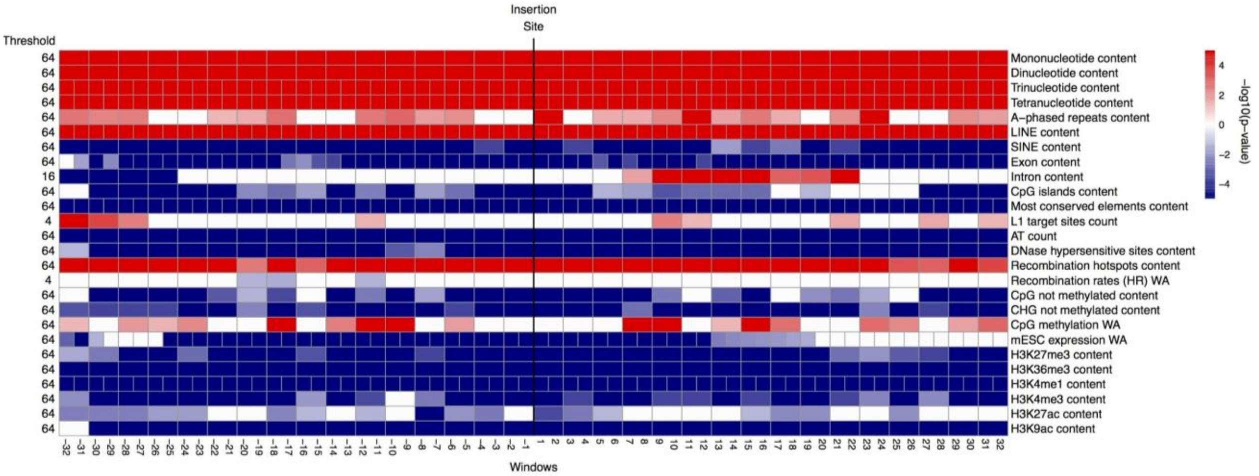

C)

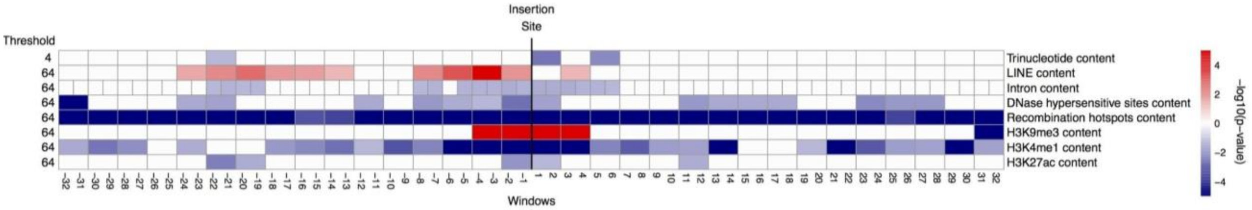
Insertion Site
Threshold
4
64
64
64
64
64
64
64
Trinucleotide content
LINE content
Intron content
DNase hypersensitive sites content
Recombination hotspots content
H3K9me3 content
H3K4me1 content
H3K27ac content
Windows
−log10(p−value)
4
2
0
−2
−4

Figure T. Significance (i.e. -log10(corrected p-value)) of genomic features in windows along the flanking regions, obtained from the ITP using the **variance** difference as test statistics: (A) fixed HERV-Ks vs. controls, (B) *in vitro* HERV-Ks vs. controls, and (C) fixed vs. *in vitro* HERV-Ks. In each panel, the horizontal axis represents the 64 1-kb windows. The vertical black line between window -1 kb and 1 kb marks the integration site. The thresholds reported on the left represent the maximum scale at which each feature is significant, ranging from 64 kb (coarsest) to 1 kb (finest). Each row corresponds to one feature and each cell represents one or two contiguous windows, depending on the number of nodes employed in the B-splines (we consider one value for every 1-kb window when using the raw data, and one value every two 1-kb windows when using the piecewise constant smoothed version of the data).

White cells: not significant (p-value >0.05), red cells: significant with higher variance in the flanking regions of HERV-Ks vs. controls (or in the flanking regions of fixed vs. *in vitro* HERV-Ks), blue cells: significant with lower variance in the flanking regions of HERV-Ks vs. controls (or in the flanking regions of fixed vs. *in vitro* HERV-Ks). Color intensity is proportional to significance (more intense colors correspond to lower corrected p-values).

A)

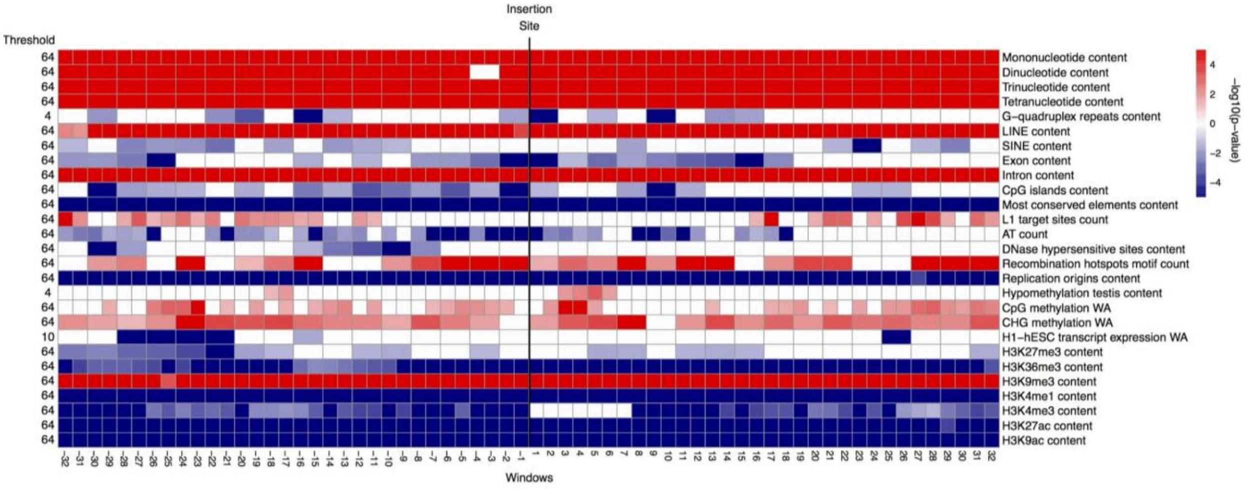

B)

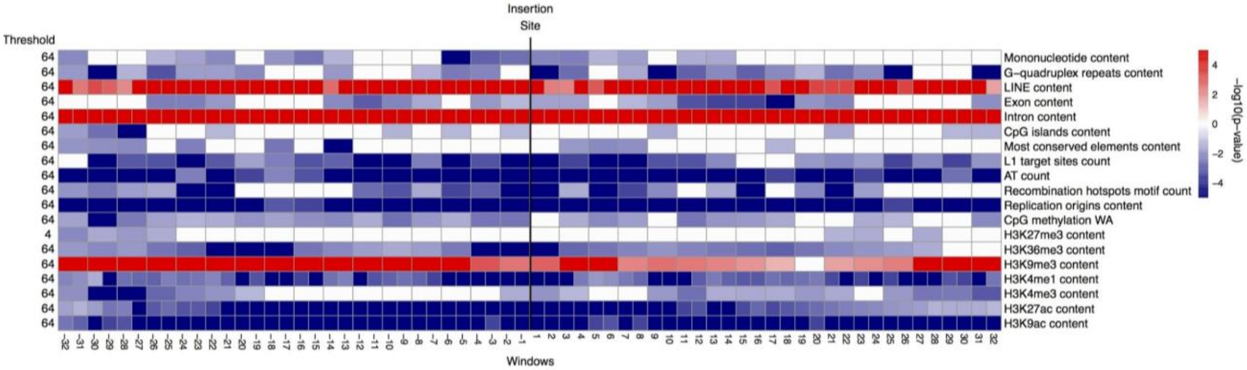

C)

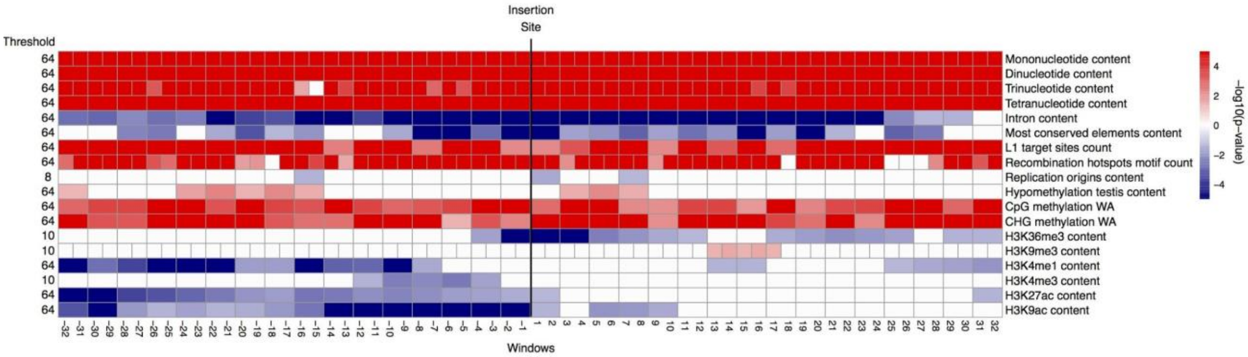
Insertion Site
Threshold
64 Mononucleotide content
64 Dinucleotide content
64 Trinucleotide content
64 Tetranucleotide content
64 Intron content
64 Most conserved elements content
64 L1 target sites count
64 Recombination hotspots motif count
8 Replication origins content
64 Hypomethylation testis content
64 CpG methylation WA
64 CHG methylation WA
10 H3K36me3 content
10 H3K9me3 content
64 H3K4me1 content
10 H3K4me3 content
64 H3K27ac content
64 H3K9ac content
−log10(p−value)
4
2
0
−2
−4
Windows
